# The heritability of vocal tract structures estimated from structural MRI in a large cohort of Dutch twins

**DOI:** 10.1007/s00439-022-02469-2

**Published:** 2022-07-13

**Authors:** Dan Dediu, Emily M. Jennings, Dennis van’t Ent, Scott R. Moisik, Grazia Di Pisa, Janna Schulze, Eco J. C. de Geus, Anouk den Braber, Conor V. Dolan, Dorret I. Boomsma

**Affiliations:** 1 Department of Catalan Philology and General Linguistics, University of Barcelona, Barcelona, Spain; 2 Universitat de Barcelona Institute of Complex Systems (UBICS), Barcelona, Spain; 3 Catalan Institute for Research and Advanced Studies (ICREA), Barcelona, Spain; 4 Faculty of Linguistics, Philology and Phonetics, University of Oxford, Oxford, UK; 5 Department of Biological Psychology, Vrije Universiteit Amsterdam, Amsterdam, The Netherlands; 6 Linguistics and Multilingual Studies, Nanyang Technological University, Singapore, Singapore; 7 Department of Linguistics, Universität Konstanz, Constance, Germany; 8 Independent Researcher, Nijmegen, The Netherlands; 9 Department of Neurology, Alzheimer Center, Neuroscience Amsterdam, Amsterdam UMC, Vrije Universiteit Amsterdam, Amsterdam, The Netherlands

## Abstract

While language is expressed in multiple modalities, including sign, writing, or whistles, speech is arguably the most common. The human vocal tract is capable of producing the bewildering diversity of the 7000 or so currently spoken languages, but relatively little is known about its genetic bases, especially in what concerns normal variation. Here, we capitalize on five cohorts totaling 632 Dutch twins with structural magnetic resonance imaging (MRI) data. Two raters placed clearly defined (semi)landmarks on each MRI scan, from which we derived 146 measures capturing the dimensions and shape of various vocal tract structures, but also aspects of the head and face. We used Genetic Covariance Structure Modeling to estimate the additive genetic, common environmental or non-additive genetic, and unique environmental components, while controlling for various confounds and for any systematic differences between the two raters. We found high heritability, *h*^2^, for aspects of the skull and face, the mandible, the anteroposterior (horizontal) dimension of the vocal tract, and the position of the hyoid bone. These findings extend the existing literature, and open new perspectives for understanding the complex interplay between genetics, environment, and culture that shape our vocal tracts, and which may help explain cross-linguistic differences in phonetics and phonology.

**Supplementary Information:**

The online version contains supplementary material available at 10.1007/s00439-022-02469-2.

## Introduction

The various organs comprising the human vocal tract, such as the tongue, the larynx, the lips, the hard palate, and the jaws (Gick et al. 2013), are essential for the production of speech. Much more is known about the genetics of their developmental abnormalities, affecting, for example, the teeth (Cobourne and Sharpe 2013; Brook et al. 2014; Phan et al. 2016; Lu et al. 2017), the hard palate and the upper lip (Dixon et al. 2011; Leslie and Marazita 2013), the larynx (Birkent et al. 2012), and the tongue (Topouzelis et al. 2011; Hong 2013), than about the genetic underpinnings of their normal variation and the interplay between genetics, environment, and cultural practices shaping them (von Cramon-Taubadel 2011; Šešelj et al. 2015; Švalkauskienė et al. 2015; Richmond et al. 2018; Blasi et al. 2019; Weinberg et al. 2019; Paul et al. 2021).

Our study is one of the first to explicitly address the question of the heritability of the various components of the vocal tract based on data from a large cohort of twins and a comprehensive set of rigorously defined measures based on MRI structural scans. Our study capitalizes on a large mega-sample of five studies, collected across almost 2 decades by the Netherlands Twin Register (NTR; https://tweelingenregister.vu.nl), totaling 632 twins, composed of 290 complete twin pairs and 48 with data for only one twin (all these coming from a single study), distributed among 249 (73.2%) monozygotic pairs and 91 (26.8%) dizygotic pairs. We analyzed 146 phenotypes capturing anatomical aspects of most structures (soft and rigid) of the vocal tract, extracted from 3D structural MRI scans and coded in parallel by two independent raters following the same coding manual. The two expert raters placed landmarks (that identify clearly defined anatomical structures, such as the “nasion”), and semi-landmarks (that trace the contour of a curve or surface). From these, we derived a set of measures (distances, angles, curvatures, ratios, principal component scores, and Procrustes distances) that capture the dimensions and shape of the structures of the vocal tract and their inter-relationships. We then fitted, for each measure separately, a genetic covariance structure modeling (GCSM) that includes the additive genetic component (*A*), the common environmental component (*C*) or the non-additive genetic component (*D*), and the unique environmental circumstances (and the measurement error) (*E*), while controlling for various confounds (sex, age, and intra-cranial volume) and for any systematic differences between the two raters.

The paper is structured as follows: we first describe the data and the methodology, followed by the results, and we end with a discussion and conclusions that contextualize our study in the wider literature of what is currently known about the heritability and genetics of variation of the skull and face, as well as in terms of their significance for understanding the evolution of speech and language, the patterns of normal variation between individuals, and, potentially, the patterns of normal variation between languages.

## Materials and methods

### Participants

**Fig. 1:**
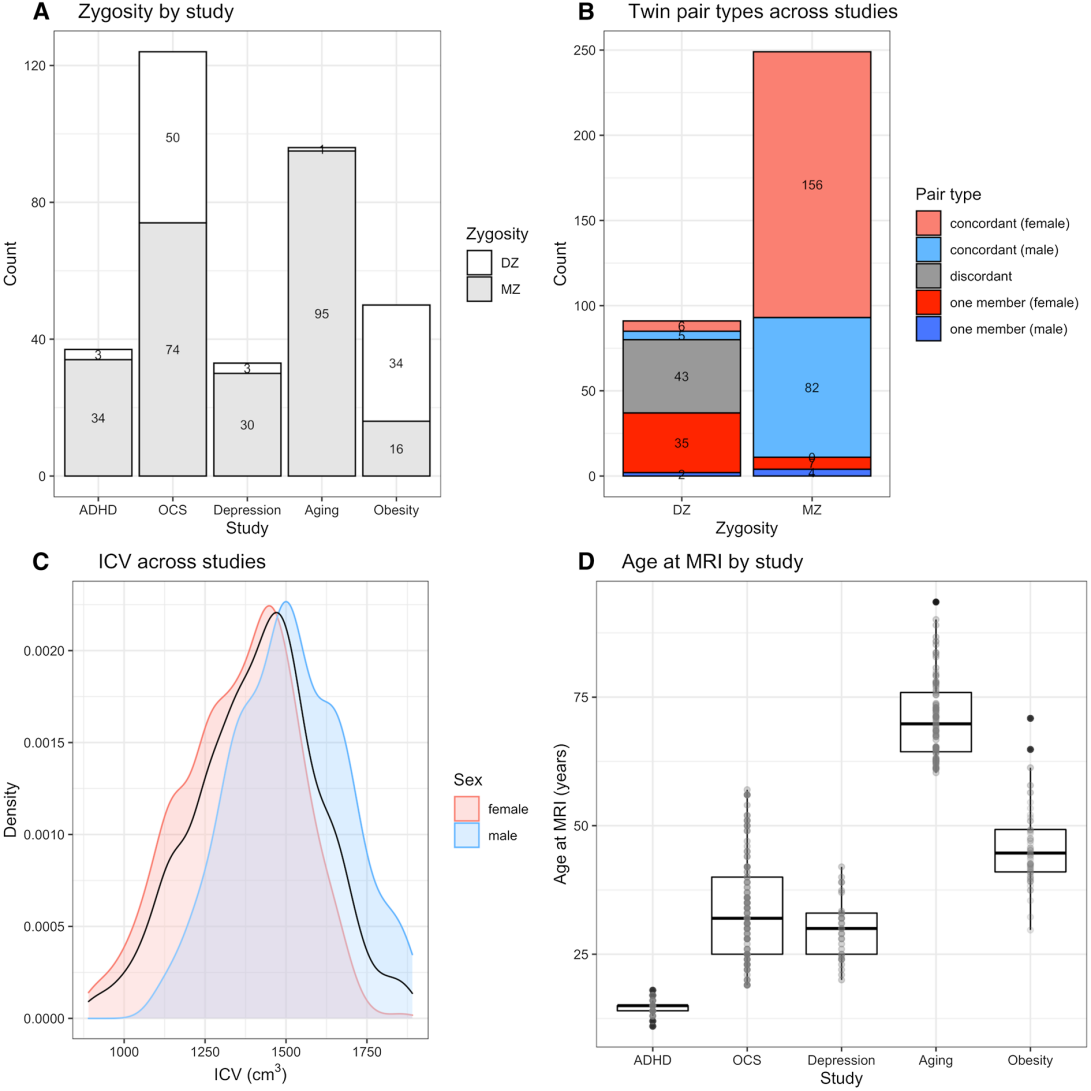
Properties of the mega-sample. **A** Distribution of the twins across the five studies (ADHD, OCS, Depression, Aging and Obesity, on the horizontal axis) combined in the current mega-sample, showing the zygosity of the pairs and their actual counts (stacked bars; gray represents the MZ twins and white the DZ twins). **B** Various types of twin pairs (represented by colors) across studies (for those pairs with only one member included, we show the single member’s sex). **C** Distribution of the intra-cranial volume (ICV) in cm^3^ across all studies as density plots per sex (colored areas and curves) and overall (black curve). **D** Distribution of age (in years) at the time of the MRI for each study (on the horizontal axis) separately as box plots. Generated automatically using R 4.1.3 (https://www.r-project.org/)

T1-weighted MRI data of vocal tract (VT) structures were available from five studies previously conducted by the Netherlands Twin Register (NTR) (van Beijsterveldt et al. 2013; Willemsen et al. 2013; Ligthart et al. 2019). These prior studies were focused on (1) Attention Deficit/Hyperactivity problems (*ADHD*) (van’t Ent et al. 2007), (2) Obsessive Compulsive Symptoms (*OCS*) (den Braber et al. 2010), (3) *Depression* (de Geus et al. 2007), (4) *Aging* (Konijnenberg et al. 2018), and (5) *Obesity* (Doornweerd et al. 2017)—none of these primarily concerning the anatomy of the vocal tract. Figure 1 provides an overview of the participants and the studies. The total sample size *n* is 632 participants (= 2 × 292 [complete twin pairs] + 1 × 48 [single-member twin pairs]). This was composed of 64.7% females (*n*_females_ = 409) and 35.3% males (*n*_males_ = 223). The age range was 11–93.5 years, with a mean of 43.5 and standard deviation of 21 years, but with large variation between the studies (see Fig. 1 panel D). There were 73.2% (*n*_MZ_ = 249) monozygotic (MZ) twin pairs, and 26.8% (*n*_DZ_ = 91) dizygotic (DZ) twin pairs, of which 5.5% (*n*_DZm_ = 5) are concordant-sex male pairs, 6.6% (*n*_DZf_ = 6) are concordant-sex female pairs, 47.3% (*n*_DOS_ = 43) are opposite-sex dizygotic twin (dizygotic opposite sex or DOS) pairs (the remaining 40.7%, *n*_single_ = 37, having one member with missing data), with large variation between studies (see Fig. 1 panels A and B). The twins’ zygosity [monozygotic (MZ) or dizygotic (DZ)] was based on DNA polymorphisms (Odintsova et al. 2018).

### Vocal tract measures

A set of features of the vocal tract that were of interest to understanding inter-individual variation in speech production was identified, resulting in a list of standardized *landmarks* and *semi-landmarks*. Briefly, while landmarks represent fixed, well-defined anatomical points, semi-landmarks are used to describe curves and do not specify fixed points. For example, when describing the midsagittal shape of the hard palate, we may place several semi-landmarks to allow the actual shape to be well approximated (see (Dediu and Moisik 2019) and Table S1 for details). The process of measuring the vocal tract anatomical features of interest involves the following steps: first, a few key landmark points (the tip of the nose, the top, back, left, and right sides of the head) were automatically estimated on the T1 MRI scans by a custom MATLAB script (The Mathworks Inc. 2019) called VTANALYZER. Second, these key landmark points were manually adjusted by two raters with VTANALYZER. In doing so, they used those key points to make rough predictions about the placement of several “first-order” landmark points (e.g., the base of the second cervical (C2) vertebral body, the basion, and the odontoid). Third, these predicted locations of the first-order landmarks were manually adjusted by the same two raters, who then traced the “second-order” semi-landmarks representing various curves (e.g., the maxillary dental arch and the pharynx wall). The two raters were trained in the same way, had access to the same guidelines, software and hardware, were blinded to the twin relationships and zygosity, and landmarked the data independently. However, they were free to discuss problematic cases. Thus, each MRI scan in the dataset resulted in two sets of corresponding landmarks and semi-landmarks (one per rater). Some scans had various degrees of missing data due to indiscernible features in a participant’s scans (e.g., the presence of braces in a patient’s mouth would obliterate the signal in a large region of the anterior part of the vocal tract; please see the Text S1 for details).

The landmarks and semi-landmarks were used to derive 146 *phenotypic measures* (PMs; please see Text S2 for details) of five types: *distances* (the metric distance between two points; e.g., the width of the dental arch between the canines), *angles* (the angle between the two lines connecting three points; e.g., the angle between the nasal cavity floor and inferior right central incisor), *curvatures* (estimated from quadratic regressions; e.g., the curvature of the maxillary dental arch), *ratios* (ratio of two PMs of the same type; e.g., the ratio of intercanine width to intermolar width), and *Procrustes distances* (measures of shape similarity between two sets of corresponding points between an individual and a mean, as obtained after the translation, scaling, and rotation of the sets of corresponding points (Zelditch et al. 2012); e.g., the Procrustes distance between the tracing of an individual’s maxillary dental arch and the mean configuration of the maxillary dental arch, defined by all scans with maxillary dental arch tracing). The majority of the PMs were of type distance (62.3%). The PMs were also grouped by *domain* (the broad anatomical component to which they refer): the *hard palate* (22.6%), the *skull* (19.2%), and the *larynx* (13.7%) being the most numerous. See Table 1 for counts and percents, Table S2 for the detailed list of PMs, and Table 2 for the subset of PMs with notable heritabilities. 18 pairs of PMs were very highly correlated (Pearson’s *r* ≥ 0.90; see Table S3).

**Table 1:** Distribution of the 146 phenotypic measures (PMs) by domain (rows) and type (columns) as counts, with row- and column-wise totals and percentages (rounded to one decimal)

Type/domain	Angle	Curvature	Distance	Procrustes. dist	Ratio	Total
Cervical	0	0	7	0	0	7 (4.8%)
Dentition	4	1	5	1	0	11 (7.5%)
General	0	0	4	2	0	6 (4.1%)
Hard palate	4	5	20	4	0	33 (22.6%)
Hyoid	0	0	5	0	0	5 (3.4%)
Larynx	0	0	20	0	0	20 (13.7%)
Mandible	2	0	12	0	0	14 (9.6%)
Oral	0	0	0	0	12	12 (8.2%)
Pharynx	0	7	1	1	0	9 (6.2%)
Skull	12	0	16	0	0	28 (19.2%)
Soft palate	0	0	1	0	0	1 (0.7%)
Total	22 (15.1%)	13 (8.9%)	91 (62.3%)	8 (5.5%)	12 (8.2%)	146 (100.0%)

Because all of our data were landmarked by two raters independently, we examined the agreement between raters as an indication of which VT PMs were most reliably estimated. Inter-rater reliability was assessed via the intra-class correlations coefficient (ICC), a standardized reliability measure, with values closer to 1.0 indicating stronger agreement, values close to 0.0 indicating randomness, and negative values indicating systematic disagreement. We specifically considered the consistency formula ICC(*C*,1), which measures the consistency among the measurements and is insensitive to bias effects (McGraw and Wong 1996). It is considered that ICC values ≥ 0.75 represent “good” reliability measures, and those ≥ 0.90 are “excellent” (Koo and Li 2016), which restricts the noise in the measurement to one-third or less of the spread among the true scores (Liljequist et al. 2019). Given that the estimates of ICC(*C*,1) are subject to uncertainty, as expressed in their standard errors, we consider those measures for which the lower limit of their 95% confidence interval (95%CI) of the ICC(*C*,1) is ≥ 0.75 to have very high reliability.

The following covariates were included in the genetic analyses to account for their effects on the PMs: age at time of MRI (age), sex, and intra-cranial volume (ICV). Age and sex were included, because there are well-documented anatomical differences in vocal tract structures across age (e.g., vocal fold thickness (Hollien and Shipp 1972), the ossification of the epiglottis (Kahane 1987), the size of the craniofacial structures (Israel 1973; Flügel and Rohen 1991), and the length and volume of the oral cavity (Xue and Hao 2003)) and between the sexes (e.g., vocal fold length Kahane (1978), the ratio of pharynx to mouth cavity length, and laryngeal cavity size (Fant 1966)). ICV, the estimated volume of the cranial cavity (which is outlined by the supratentorial dura mater, or the cerebral contour when the dura mater is not detectable), reaches a maximum around 10 years of age (Pfefferbaum et al. 1994) and remains stable across the lifespan (Blatter et al. 1995). ICV serves as an indication of head size, and is commonly used as a normalization factor during MRI image registration (Eritaia et al. 2000). ICV estimates were obtained through an automated process implemented by FreeSurfer (Fischl 2012; Fischl et al. 2013) version 5.1 (http://freesurfer.net/), a popular MRI processing and visualization software suite. There were seven participants without ICV, for whom we imputed the average ICV for cases of the same sex and age group, with “age group” meaning all twins within 1 year of the age associated with the imputed ICV. Age and ICV were standardized (*z*-scored). Standardized age (satm—from standardized age at measurement), standardized ICV (sICV), and standardized age squared (satm^2^) were defined as fixed effects in the regression analyses; the latter was included, because the effect of age may not be strictly linear.

### Data processing

All analyses were performed on Ubuntu 18.04 and macOS 12 using R (R Core Team 2021) (versions 4.0.5 and 4.1.3; https://www.R-project.org/), RStudio (RStudio Team 2020) (version 2022.02.0; https://www.rstudio.com/), and OpenMX 2 (Boker et al. 2011; Neale et al. 2016) as implemented by the corresponding R package (version 2.19; https://cran.r-project.org/package=OpenMx), using a Macbook Air (2021) laptop with an Apple M1 CPU and 16 Gb RAM, and a desktop machine with an AMD Ryzen 3700X CPU (8 cores with hyperthreading at 4.4 GHz maximum frequency) and 64 Gb RAM. Within both raters’ datasets, there were six duplicate cases: these are three sets of MZ female twins that participated in two studies (the OCS and Aging studies), whose age ranges were 52.0–56.0 years when data were collected in 2005 and 2008 for the OCS study, and 60.9–65.5 years when data were collected in 2014 for the Aging study; we removed the duplicate cases belonging to the Aging study, leaving only their data in the OCS study.

### Testing assumptions, detecting outliers, and “warning scores”

We defined outliers as data points further from the mean by more than 3 standard deviations, and we removed them from the dataset. Linearity and homoscedasticity were assessed by residual versus fitted value plots for each PM regressed on each of the covariates. Whether or not a PM was normally distributed was assessed visually with Q–Q plots. Most of the PMs were linearly related to the covariates, homoscedastic, and normally distributed. While we estimated all valid PMs in all participants with usable data, there are several PMs that should be treated with care given that they violate the assumptions of the parametric methods we use (especially of the GCSM). Therefore, we computed a “warning score” based on (a) the visual inspection of the histograms and Q–Q plots, particularly focusing on high skewness, kurtosis, and signs of bi-modality, (b) if the latent twin correlation estimates (from the phenotypic model—see below) were much larger than 1.0, (c) the comparison of the estimated skewness against the intervals [−1,1] (high skewness) and [−1,−0.5] ∪ [0.5,1] (moderate skewness), (d) the comparison of kurtosis to [2,5], and, (e) the formal Shapiro–Wilk normality test. These “warning scores” can vary between 1 (no or very weak reasons to worry) up to 10 (very strong warning), and are intended to be used to filter or weight the interpretation of the results for each individual measure, with values ≤ 3 probably posing no problems. However, there are 15 measures with scores ≥ 5 raising potentially serious issues (see Table S2).

### The genetic covariance structure model

**Fig. 2:**
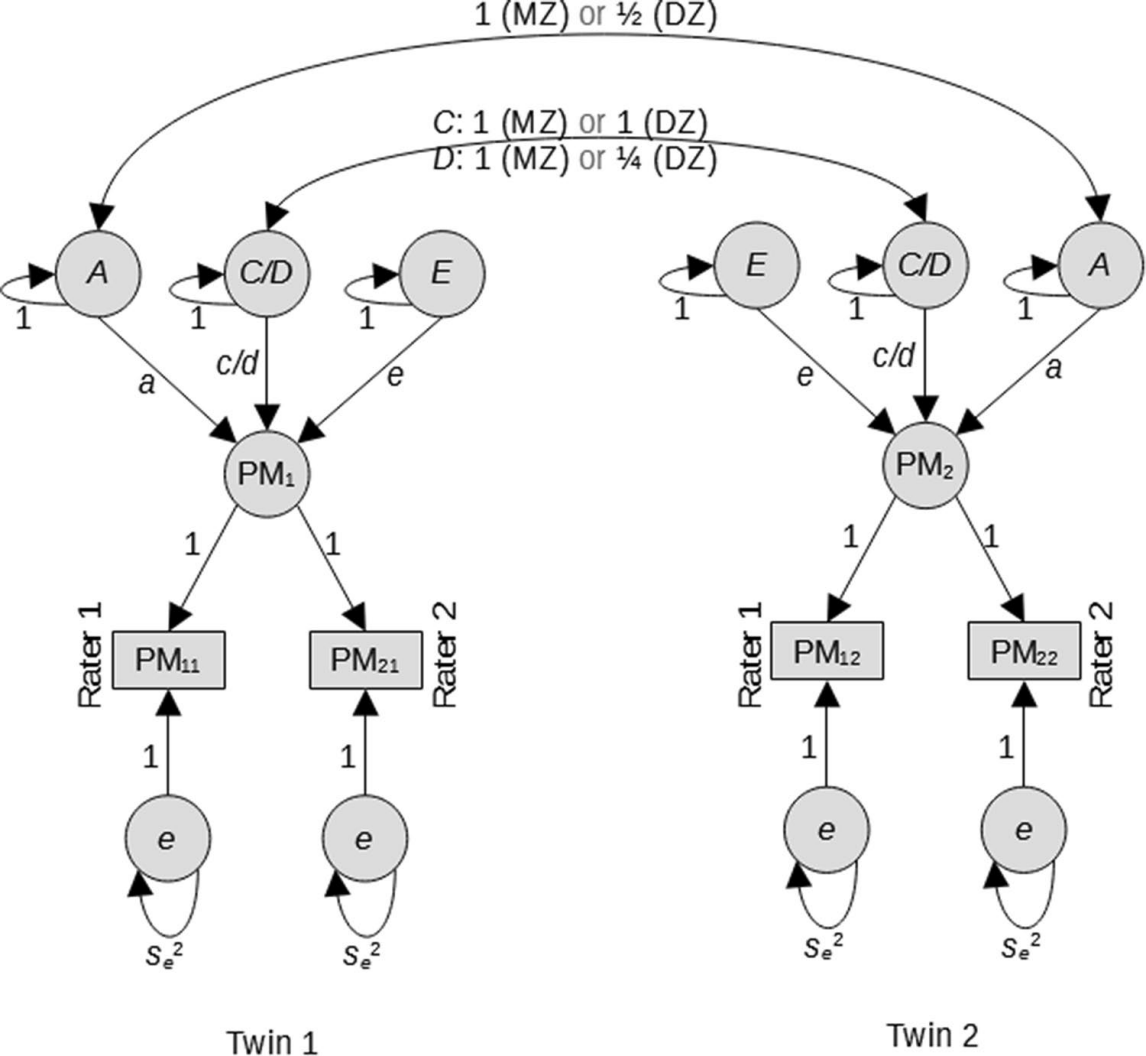
The genetic covariance structure model (GCSM). Given a phenotypic measure PM and a twin pair, we denote as PM_1_ and PM_2_ are the latent values of this measure for the two members of the twin pair, “Twin 1” and “Twin 2”. These are indexed each by the two raters, “Rater 1” and “Rater 2”, producing the four observed values, two per co-twin, denoted as PM_*ij*_, where *i* ∈ {1,2} stands for the rater and *j* ∈ {1,2} for the twin; se^2^ is the variance of the measurement error. The latent measurements PM_1_ and PM_2_ are each influenced by the effects of the additive genotype *A*, the non-shared environment *E*, and of the dominance genetic factor *D* or the shared environment *C*, as appropriate. The correlation between the additive factors *A* of the two twins differs between MZ (1.0) and DZ (1/2) twins, as do the correlations between dominance effects *D* (1.0 for MZ and 1/4 for DZ). The correlation of the shared environment *C* equals 1.0 by definition. Please note that the fixed effects of the covariates are included in the fitted model, but not represented in this figure to avoid cluttering. Drawn manually using LibreOffice Draw 7.2 (https://www.libreoffice.org/)

The two raters coded the same MRI scans independently, following the same training guidelines and using the same platform, but they were free to discuss complex cases. To model the rater effects, we needed to decide if the raters have the same error variance (i.e., the relative magnitude of their errors is the same), which turned out to be the case (see Text S3 and Table S4). Therefore, we modeled the data, as shown in Fig. 2; for a given PM, the measurements of the two raters loaded on the single latent phenotype (i.e., the PM corrected for rater error), with the residual (error) variances of the two raters being equal.

The classical twin design provides a means of estimating the relative contributions of genotype and environment to the variance of the phenotype of interest (Knopik et al. 2016). The design exploits the fact that MZ twins are (nearly) genetically identical, while DZ twins on average share 50% of their segregating alleles to estimate the contributions of additive genetic (*A*) factors, common (or shared) environment (*C*) or dominance (*D*), and twins’ unique (or unshared) environmental circumstances (*E*) to phenotypic variance. The contributions of each factor, i.e., *A*, *C* or *D*, and *E*, to the overall phenotypic variance, *V*, were estimated using maximum-likelihood estimation in genetic covariance structure modeling (GCSM) (Rijsdijk and Sham 2002; Neale and Maes 2004; Franić et al. 2013; Knopik et al. 2016). GCSM was applied to the PMs in two zygosity groups, while simultaneously including the covariates sex, satm, satm^2^, and sICV (see above).

To obtain an estimate of the correlation between MZ and DZ twins, for each PM, we first fitted a constrained two-common factor model (or a “phenotypic model”). In this model, the assessments of each rater pertaining to twin 1 were regressed on a common latent variable (latent PM_1_), as were the assessments pertaining to twin 2 (latent PM_2_). This model included two intercepts, one for the first rater and one for the second rater, which were constrained to be equal over twins. The model includes 4 residual variances (given 2 raters rating the PM in 2 twins), which were modeled as equal. To scale the latent phenotype, the regression coefficients (the factor loadings) were constrained at 1.0, and the common factor covariance matrix was estimated, with the common factor variances constrained to be equal. We fitted this model simultaneously in the MZ and DZ twins, while constraining all parameters to be equal over zygosity, except for the covariance of the common factors (the covariance between latent PM_1_ and latent PM_2_). From the model output, the MZ and DZ correlations (*r*_MZ_, *r*_DZ_) for each PM, corrected for rater error and the covariates, were obtained. When testing a large number of phenotypes affected by measurement error in relatively small samples, we expect that some will produce inadmissible or inconsistent results. Therefore, we did not constrain a priori the PM_1_ – PM_2_ covariance matrix to be positive (semi) definite, so we can detect the PMs that are inconsistent with the assumptions of our model. With these, we observed these correlations to be slightly > 1.0 for 3 (2.1%) PMs (*CS2A*: 1.025, *SNOL*: 1.022, *SNOR*: 1.011) in MZ twins, and for another 1 (0.7%) (*HMSP*: 1.04) in DZ twins, which are small enough to be dismissed as random fluctuations or numeric errors. However, for 2 (1.4%) PMs, the *r*_DZ_ were much larger than 1.0 (*HCCP*: 1.532, *HMDC*: 1.158), suggesting that they violate assumptions of the model and forcing us to assign them a warning score of 8. This phenotypic two-common factor model informed us on the subsequent decomposition of the phenotypic variance into genetic and environmental components (see Fig. 2).

In the subsequent genetic model, we based the decision to fit an ADE (*a*dditive, *d*ominance, and non-shared *e*nvironment) model or an ACE (*a*dditive, *c*ommon environment, and non-shared *e*nvironment) model on the latent PM twin correlations, *r*_MZ_ and *r*_DZ_ (see Fig. 2). To this end, we applied the following common heuristic: we fitted ADE if *r*_MZ_ > 2*r*_DZ_; otherwise, we fitted ACE. (Please note that it is not possible to simultaneously estimate both *C* and *D*, i.e., to fit the full ACDE model, as all four variance components are not identified in a univariate model.) For each of the *A*, *E*, and *C* or *D* (the choice between the latter two denoted in the following as *C/D*), we obtained the point estimates and 95% confidence intervals (95% CIs) of the path coefficients *a*, *e* and *c/d*, and we also tested their individual contribution by performing model comparison (using ΔAIC with a cut-off of 2, and the likelihood ratio test at an *α*-level of 0.05, which turned out to be virtually identical) versus the constrained model with the component fixed to 0 (e.g., free *a* versus *a* fixed to 0.0).

## Results

All the results and plots are available in the accompanying full analysis report, and are summarized in Table S5 and Figs. S4–S15.

### Covariates and predictors

The included covariates are sex, satm (z-scored age at MRI scan), satm^2^ (z-scored squared age at MRI scan), and sICV (z-scored intra-cranial volume; see Fig. 1 for descriptive statistics). There are more females (*n*_females_ = 409; 64.7%) than males (*n*_males_ = 223; 35.3%), and there are differences in the female:male ratio across the five studies from which our sample is drawn (% females: ADHD = 62.2%, OCS = 63.1%, depression = 60%, aging = 57.2%, obesity = 100%). The age at MRI (non-*z*-scored; in years) ranges between 11 and 93.5 (mean 43.5, median 39, sd 21 and iqr 37), distributed similarly between sexes but different between studies (by design). The ICV (non-*z*-scored; in cm^3^) ranges between 890 and 1890.2 (mean 1410.5, median 1425.6, sd 191.4 and iqr 249.2), being, as expected (Pfefferbaum et al. 1994), slightly larger for males (by 156.5 cm^3^ on average).

### Inter-rater agreement

For each PM, we estimated the inter-rater agreement as the intra-class coefficient (ICC) ICC(*C*,1) (McGraw and Wong 1996), as Krippendorff’s *α* (Krippendorff 2004), and as estimated from the GCSM model by 1 − (the standardized rater error variance) = (*A* + (*C* or *D*) + *E*)/var(PM). Preliminary analyses suggest that the first and the last are virtually identical (Pearson’s *r* = 0.99, *p* = 3.24 × 10^–122^; given the more general nature of ICC(*C*,1), this is the one we use here) and are better suited to our data than Krippendorff’s *α*. ICC(*C*,1) varies between 0.07 and 0.98 (mean 0.6, median 0.63, sd 0.23, iqr 0.35) overall (see Table S5 and Fig. S1); the domains with the highest agreement (*F*(10,135) = 13.05, *p* = 9.34 × 10^–16^; all pairwise comparisons significant) are the mandible, the skull, “general”, and the hyoid, while the types with the highest agreement (*F*(4,141) = 19.59, *p* = 7.74 × 10^–13^; all pairwise comparisons significant) are the angles and the distances. There are 37 PMs with the lower bound of the 95%CI of their ICC(*C*,1) ≥ 0.75, and 7 extra PMs that, while including 0.75 in the 95%CI of their ICC(*C*,1), still have an ICC(*C*,1) ≥ 0.75.

### Twin correlations

The phenotypic model allows the estimation of the twin correlations corrected for covariates and rater error. The corrected Pearson’s correlations between the two members of the MZ twin pairs, *r*_MZ_, varied between 0.04 and 1.03 (mean 0.65, median 0.65, sd 0.18, iqr 0.26), while for the DZ twin pairs, *r*_DZ_, they vary between −0.72 and 1.53 (mean 0.38, median 0.39, sd 0.28, iqr 0.28); please see above for details about the 6 correlations over 1.0. For 130 PMs (89%) *r*_MZ_ > *r*_DZ_, and for 53 of these (40.8%), *r*_MZ_ > 2*r*_DZ_. The inter-rater agreement *ICC*(*C*,1) correlates positively with *r*_MZ_ (Pearson’s *r* = 0.54, *p* = 3.11 × 10^–12^), but not with *r*_DZ_ (Pearson’s *r* = 0.16, *p* = 0.056).

### Maximum-likelihood estimates of the variance components

Using the *r*_MZ_ > 2*r*_DZ_ heuristic, there are 93 PMs where *ACE* seems the appropriate model (of which 29 have ICC(*C*,1) ≥ 0.75, of which 24 also exclude 0.75 from their 95% CI), and 53 where *ADE* seems the appropriate model (of which 15 have ICC(*C*,1) ≥ 0.75, of which 13 also exclude 0.75 from their 95%CI). Both the ACE and ADE models include the additive genetic (*A*) and the non-shared environment (*E*) variance components, and while the former also includes the shared environment (*C*) variance component, the latter includes the dominance (*D*) variance component. For each of these variance components, we also obtained their 95%CIs, and we performed formal tests of their significance by means of a likelihood ratio test (with *α*-level 0.05) and by comparing their Akaike Information Criteria (AIC, using the ΔAIC > 2 rule of thumb) (Aho et al. 2014), comparing the model with and the model without the variance component of interest (e.g., for *C*, we compared ACE and AE). These two formal criteria turned out to be virtually equivalent (for *A*, of 146 PMs, they agree on 146 and disagree on 0, and for *E*, of 146 PMs, they agree on 146 and disagree on 0; for *C*, of the 93 PMs with and ACE model, they agree on 92 and disagree on 1, and for *D*, of the 53 PMs with and ADE model, they agree on 53 and disagree on 0); see Table S6.

We are obviously interested in the squared standardized estimates *a*^2^ (narrow-sense heritability, commonly symbolized as *h*^2^), *c*^2^, *d*^2^ and *e*^2^. Comparing these among themselves and with the inter-rater agreement, ICC(*C*,1), we found that (see also Fig. S2 and Table S7) ICC(*C*,1) is positively correlated (all *p* values are Bonferroni-corrected) with *h*^2^ (Pearson’s *r* = 0.42, *p* = 2.83 × 10^–6^), negatively with *e*^2^ (Pearson’s *r* = − 0.64, *p* = 6.87 × 10^–16^), but not with *c*^2^ nor with *d*^2^ (Pearson’s *r* = − 0.16, *p* = 0.607, and Pearson’s *r* = 0.20, *p* = 0.694, respectively).

As Fig. S3 shows, *h*^2^ differs by domain (*F*(10,120) = 6.64, *p* = 3.85 × 10^–8^), being significantly higher for the skull, but not by type (*F*(4,126) = 2.15, *p* = 0.078). *c*^2^ differs by domain (*F*(10,74) = 3.99, *p* = 2.23 × 10^–4^), being higher for “general”, and possibly by type (*F*(4,80) = 3.07, *p* = 0.021). *d*^2^ differs by domain (*F*(7,38) = 3.79, *p* = 0.003), being higher for the mandible and larynx, but not by type (*F*(3,42) = 0.91, *p* = 0.442). Finally, *e*^2^ differs by domain (*F*(10,120) = 16.62, *p* = 1.7 × 10^–18^), being higher for the larynx, oral and the hard palate, and by type (*F*(4,126) = 6.93, *p* = 4.51 × 10^–5^), being higher for ratios, curvatures, and Procrustes distances. It is encouraging to note the lack of systematic differences in *h*^2^, *c*^2^, and *d*^2^ among types of PMs as none was expected a priori, while the higher *e*^2^ for ratios, curvatures, and Procrustes distances was to be expected, being due to their higher (cumulated) measurement errors.

As detailed in Text S4, as an extra check, we re-implemented the GCSM model in lavaan (Rosseel 2012) version 0.6, and we obtained very similar results: for *h*^2^, Pearson’s *r* = 0.82, *p* = 2.08 × 10^–37^, for *c*^2^, Pearson’s *r* = 0.64, *p* = 1.74 × 10^–10^, for *d*^2^, Pearson’s *r* = 0.73, *p* = 8.73 × 10^–9^, and for *e*^2^, Pearson’s *r* = 0.85, *p* = 4.83 × 10^–42^. However, this re-implementation was not identical due to differences between the two software packages OpenMX and lavaan, mainly in that in the lavaan implementation, the covariates were not included in the SEM model, but were regressed out from the PMs previous to fitting it and the decision to fit an *ACE* or an *ADE* model was based on comparing their Akaike’s Information Criteria (AIC), and in how the numerical fitting is done in the two packages. Thus, even more so, their similarity suggests that the results are at least robust.

### Ranking the PMs

While the GCSM does model the inter-rater agreement, it does not do so perfectly, in the sense that a low inter-rater agreement fundamentally induces noise in the estimates, as confirmed by the residual negative correlation between ICC(*C*,1) and the error *e*^2^. Therefore, we decided to include the inter-rater agreement ICC(*C*,1) in our interpretation of the GSEM estimates of narrow-sense heritability, *h*^2^. For a given PM and GCSM component, its estimate can be considered in terms of its (a) statistical significance and (b) actual size; if we also consider the PM’s inter-rater agreement, we have the following cases (with their symbolic notation): is the estimate statistically significantly greater than 0 at the *α*-level 0.05 (denoted as ^*^)? Is this still significant after Holm’s (1979) multiple-testing correction (denoted as ^*c^)? Is the point estimate greater than the 0.20 threshold (denoted as ^>^)? If so, is also the lower limit of its 95% confidence interval greater than 0.20 (denoted as ^>>^)? Finally, is the point estimate of the inter-rater agreement ICC(C,1) greater than 0.75 (denoted as ^+^)? If so, is also the lower limit of its 95% confidence interval greater than this threshold? (denoted as ^++^)? The 0.75 threshold for the inter-rater agreement ICC(*C*,1) was discussed above (Koo and Li 2016; Liljequist et al. 2019), while the 0.20 threshold for narrow-sense heritability simply represents a subjective view that a contribution of additive genetic variance of more than a fifth of the total variance might justify further research into the genetic underpinnings of an anatomical phenotype. There are some logical relationships between these criteria, namely: ^*c^ ⇒ ^*^, ^>>^ ⇒ ^>^, and ^++^ ⇒ ^+^.

**Fig. 3:**
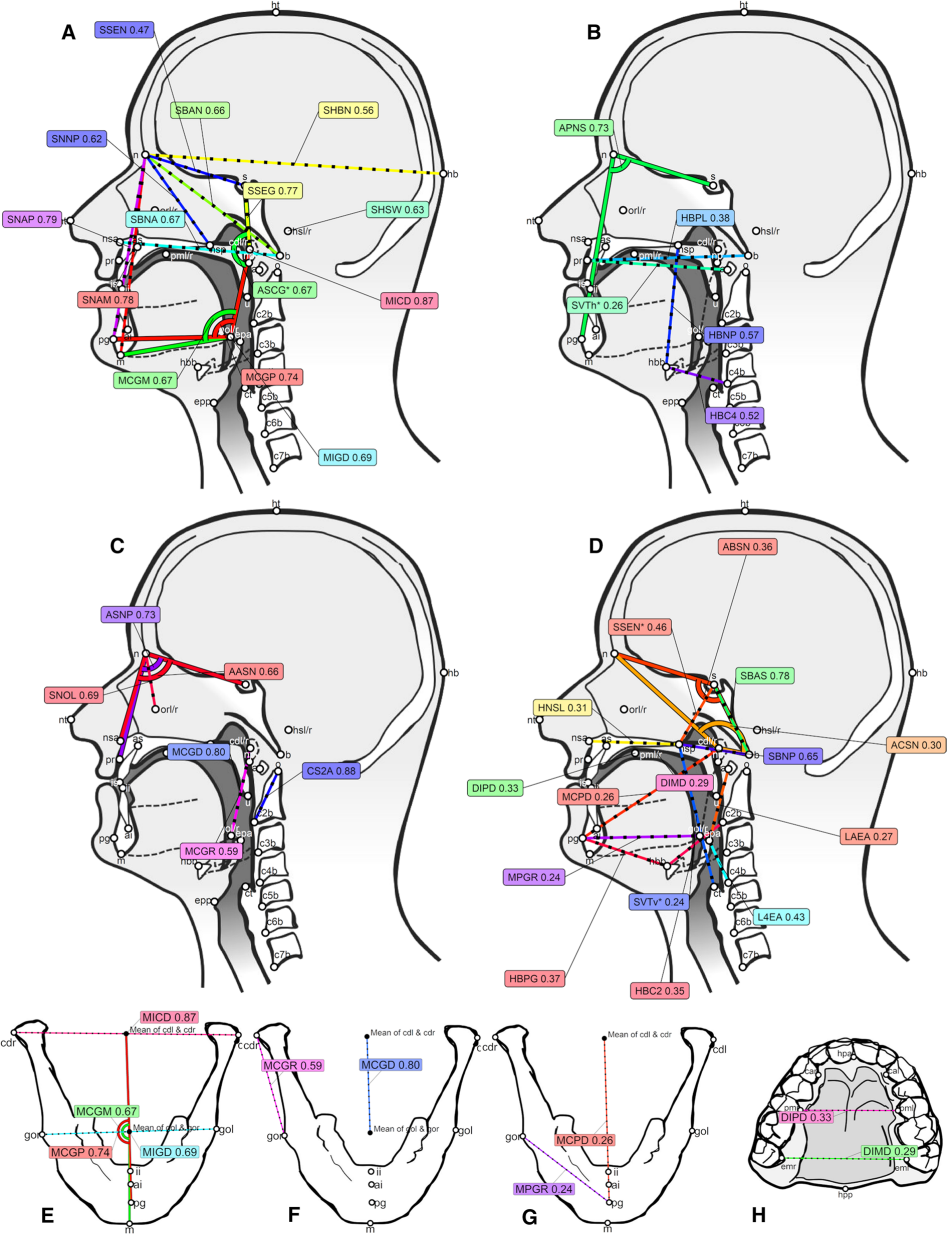
Visual representation of the PMs with evidence for narrow-sense heritability in our data. For full size images, please see the Figs. S4–S15. **A**–**D** Midsagittal view of several measures from various domains that belong, respectively, to class I (very strong evidence; **A**), class II (strong evidence; **B**), class III (moderate evidence; **C**), and class IV (circumstantial evidence; **D**) evidence. **E**–**G** Mandibular view of some mandibular measures in class I (**E**), class III (**F**), and class IV (**G**), respectively (there are no measures of class II in this view). **H** Hard palate view of a dentition measure in class IV (there are no measures of the other classes in this view). Colors help disambiguate the measures. Colored lines with dots represent distances, while solid colored lines with semi-circles represent angles. The decimal numbers after the measure codes are the point estimates of the narrow-sense heritabilities, *h*^2^. We show only the measures in class IV and higher. Please note that *ANSF* (the angle between the line from nasion to sella and the Frankfort Horizontal Plane) is not shown (it belongs to class IV and should have appeared in **D** and **G**), as we did not find a satisfactory way of visually representing it. The PMs are described in Text S2 and Table S2; see also Table 2. Drawn manually based on Figs. S4–S15 using GIMP 2.10 (https://www.gimp.org/)

For a given variance component of interest (*h*^2^, *c*^2^ and *d*^2^), we then combine these criteria to obtain a ranking of the measures from class I (providing the strongest type of evidence for a large and significant component) to class V (which effectively gives no evidence whatsoever for the relevance of this component). Please see Tables S8-S10 for details of this process, but, in brief for *h*^2^, *class I* includes those PMs with very high (≥ 0.75) inter-rater agreement and a statistically significant (after multiple-testing correction) large (≥ 0.20) narrow-sense heritability; *class II* includes those PMs with nominally significant (but that do not survive multiple-testing correction) large (≥ 0.20) narrow-sense heritability with very high (≥ 0.75) inter-rater agreement; *class III* is like class II less the inter-rater agreement; *class IV* is a collection of PMs that might give some suggestive evidence of narrow-sense heritability (≥ 0.20) but without any statistical significance but with some inter-rater agreement; finally, *class V* collects the PMs arguably without support for narrow-sense heritability. We excluded the 15 measures with warning values ≥ 5. Please see Table 2 and Fig. 3 for the results for narrow-sense heritability *h*^2^, and Table S5 and Figs. S4–S15 for all the rankings.

**Table 2:** The 41 PMs with some evidence of narrow-sense heritability *h*^2^ sorted by the strength of this evidence

Measure	Description	Domain	Type	Model	Class	*h*^2^	*p*	*c*^2^ or *d*^2^	ICC(*C*,1)
***MICD***	Width of mandible between the condyles	Mandible	Distance	ACE	I	0.87 (0.51, 0.90)	2.6 × 10^–13^ (3.8 × 10^–11^)	0.01 (0.00, 0.36)	0.98 (0.98, 0.99)
***SNAP***	Height of facial skeleton (using pogonion)	Skull	Distance	ACE	I	0.79 (0.40, 0.88)	9.5 × 10^–7^ (1.3 × 10^–4^)	0.05 (0.00, 0.43)	0.88 (0.86, 0.90)
***SNAM***	Height of facial skeleton (using menton)	Skull	Distance	ACE	I	0.78 (0.40, 0.88)	7.9 × 10^–7^ (1.1 × 10^–4^)	0.06 (0.00, 0.43)	0.90 (0.89, 0.92)
***SSEG***	Height of basicranium relative to mean condyle location	Skull	Distance	ACE	I	0.77 (0.38, 0.83)	5.2 × 10^–6^ (7.2 × 10^–4^)	0.01 (0.00, 0.39)	0.90 (0.88, 0.91)
***MCGP***	Angle of the mandible using the pogonion and the mean locations of the condyles and angles (gonion)	Mandible	Angle	ACE	I	0.74 (0.38, 0.86)	3.1 × 10^–6^ (4.3 × 10^–4^)	0.07 (0.00, 0.44)	0.87 (0.84, 0.88)
***MIGD***	Width of mandible between the angles (gonion)	Mandible	Distance	ACE	I	0.69 (0.39, 0.91)	8.4 × 10^–9^ (1.2 × 10^–6^)	0.19 (0.00, 0.49)	0.95 (0.94, 0.95)
***MCGM***	Angle of the mandible using the menton and the mean locations of the condyles and angles (gonion)	Mandible	Angle	ACE	I	0.67 (0.43, 0.85)	1.7 × 10^–5^ (0)	0.14 (0.00, 0.48)	0.89 (0.87, 0.90)
ASCG*	Angle between the line from the mean condyle location to sella and the line from the mean condyle location to the mean gonion location	Skull	Angle	ACE	I	0.67 (0.41, 0.85)	1.7 × 10^–5^ (0)	0.14 (0.00, 0.48)	0.87 (0.85, 0.89)
***SBNA***	Length of facial skeleton (using anterior nasal spine)	Skull	Distance	ACE	I	0.67 (0.32, 0.87)	5.8 × 10^–6^ (7.9 × 10^–4^)	0.17 (0.00, 0.51)	0.90 (0.88, 0.91)
***SBAN***	Length of anterior basicranium (using nasion)	Skull	Distance	ACE	I	0.66 (0.36, 0.92)	1.7 × 10^–7^ (2.4 × 10^–5^)	0.22 (0.00, 0.52)	0.89 (0.88, 0.91)
***SHSW***	Width of head	Skull	Distance	ACE	I	0.63 (0.28, 0.86)	3.2 × 10^–5^ (0)	0.19 (0.00, 0.53)	0.94 (0.93, 0.95)
***SNNP***	Length of anterior basicranium (using nasion and posterior nasal spine)	Skull	Distance	ACE	I	0.62 (0.28, 0.95)	2.2 × 10^–5^ (0)	0.29 (0.00, 0.63)	0.82 (0.80, 0.85)
***SHBN***	Length of head	Skull	Distance	ACE	I	0.56 (0.31, 0.91)	2 × 10^–9^ (2.9 × 10^–7^)	0.34 (0.00, 0.58)	0.96 (0.95, 0.96)
***SSEN***	Length of anterior basicranium (between sella and nasion)	Skull	Distance	ACE	I	0.47 (0.19, 0.87)	9.5 × 10^–5^ (0.01)	0.40 (0.00, 0.67)	0.90 (0.89, 0.92)
**APNS**	Angle between the line from nasion to pogonion and the line from nasion to sella	Skull	Angle	ACE	II	0.73 (0.25, 0.81)	7.2 × 10^–4^ (0.09)	0.02 (0.00, 0.48)	0.83 (0.80, 0.85)
**HBNP**	Distance between posterior nasal spine and hyoidale	Hyoid	Distance	ACE	II	0.57 (0.11, 0.78)	0.01 (1)	0.13 (0.00, 0.56)	0.84 (0.81, 0.86)
**HBC4**	Distance between C4 body and hyoidale	Hyoid	Distance	ACE	II	0.52 (0.04, 0.72)	0.03 (1)	0.12 (0.00, 0.57)	0.79 (0.76, 0.82)
**HBPL**	Length (anteroposterior distance) of hard palate (lower face)	Hard palate	Distance	ACE	II	0.38 (0.12, 0.78)	0 (0.27)	0.45 (0.08, 0.71)	0.91 (0.89, 0.92)
SVTh*	Length of the horizontal supralaryngeal vocal tract (using the prosthion and the atlas)	General	Distance	ACE	II	0.26 (0.03, 0.62)	0.03 (1)	0.55 (0.20, 0.77)	0.93 (0.91, 0.94)
*CS2A*	Height of C2 from its base to odontoid tip	Cervical	Distance	ACE	III	0.88 (0.36, 1.00)	7.1 × 10^–4^ (0.09)	0.12 (0.00, 0.66)	0.60 (0.55, 0.65)
*MCGD*	Height of ramus of mandible using mean location of condyles and angles (gonion)	Mandible	Distance	ACE	III	0.80 (0.31, 0.91)	7.4 × 10^–4^ (0.1)	0.02 (0.00, 0.47)	0.71 (0.67, 0.75)
*ASNP*	Angle between the line from nasion to sella and the line from nasion to prosthion	Skull	Angle	ACE	III	0.73 (0.26, 0.91)	6.7 × 10^–4^ (0.09)	0.12 (0.00, 0.58)	0.72 (0.68, 0.76)
*SNOL*	Distance from left infraorbital pit to bridge of nose	Skull	Distance	ACE	III	0.69 (0.09, 1.00)	0.02 (1)	0.31 (0.00, 0.90)	0.54 (0.48, 0.59)
*AASN*	Angle between the line from nasion to the anterior nasal spine and the line from nasion to sella	Skull	Angle	ACE	III	0.66 (0.17, 0.92)	0.01 (0.9)	0.18 (0.00, 0.65)	0.63 (0.58, 0.67)
*MCGR*	Height of right ramus of mandible	Mandible	Distance	ACE	III	0.59 (0.05, 0.82)	0.03 (1)	0.12 (0.00, 0.61)	0.68 (0.64, 0.72)
SBAS	Length of middle basicranium (using sella)	Skull	Distance	ADE	IV	0.78 (0.00, 0.86)	0.11 (1)	0.01 (0.00, 0.84)	0.79 (0.76, 0.82)
ANSF	Angle between the line from nasion to sella and the Frankfort horizontal plane	Skull	Angle	ADE	IV	0.73 (0.00, 0.84)	0.22 (1)	0.05 (0.00, 0.83)	0.79 (0.75, 0.81)
SBNP	Distance between basion and posterior nasal spine (using posterior nasal spine)	Skull	Distance	ADE	IV	0.65 (0.00, 0.86)	0.26 (1)	0.16 (0.00, 0.85)	0.80 (0.77, 0.83)
SSEN*	Height of posterior nasal cavity	Skull	Distance	ADE	IV	0.46 (0.00, 0.83)	0.39 (1)	0.32 (0.00, 0.83)	0.90 (0.88, 0.91)
L4EA	Height of larynx (using apex of epiglottis) relative to C4	Larynx	Distance	ACE	IV	0.43 (0.00, 0.69)	0.1 (1)	0.17 (0.00, 0.61)	0.76 (0.72, 0.79)
HBPG	Distance between pogonion (chin) and hyoidale	Hyoid	Distance	ACE	IV	0.37 (0.00, 0.68)	0.15 (1)	0.22 (0.00, 0.62)	0.81 (0.78, 0.83)
ABSN	Angle between the line from sella to basion and the line from sella to nasion	Skull	Angle	ADE	IV	0.36 (0.00, 0.84)	0.5 (1)	0.42 (0.00, 0.84)	0.82 (0.79, 0.84)
HBC2	Distance between C2 body and hyoidale	Hyoid	Distance	ACE	IV	0.35 (0.00, 0.73)	0.2 (1)	0.29 (0.00, 0.67)	0.78 (0.74, 0.81)
DIPD	Width of dental arch between the second premolars	Dentition	Distance	ADE	IV	0.33 (0.00, 0.72)	0.57 (1)	0.32 (0.00, 0.73)	0.80 (0.77, 0.82)
HNSL	Length (anteroposterior distance) of nasal cavity floor	Hard palate	Distance	ACE	IV	0.31 (0.00, 0.78)	0.08 (1)	0.49 (0.02, 0.80)	0.77 (0.74, 0.80)
ACSN	Angle between the line from sella to the mean location of the condyles and the line from sella to the nasion	Skull	Angle	ADE	IV	0.30 (0.00, 0.84)	0.57 (1)	0.50 (0.00, 0.85)	0.87 (0.85, 0.89)
DIMD	Width of dental arch between the second molars	Dentition	Distance	ACE	IV	0.29 (0.00, 0.75)	0.2 (1)	0.40 (0.00, 0.72)	0.77 (0.73, 0.80)
LAEA	Height of larynx (using apex of epiglottis) relative to C1	Larynx	Distance	ACE	IV	0.27 (0.00, 0.61)	0.39 (1)	0.23 (0.00, 0.58)	0.77 (0.73, 0.80)
MCPD	Length of body of mandible using pogonion and mean location of condyles	Mandible	Distance	ADE	IV	0.26 (0.00, 0.86)	0.61 (1)	0.57 (0.00, 0.87)	0.92 (0.90, 0.93)
MPGR	Length of right side of body of mandible	Mandible	Distance	ADE	IV	0.24 (0.00, 0.88)	0.67 (1)	0.60 (0.00, 0.89)	0.84 (0.81, 0.86)
SVTv*	Length of the vertical supralaryngeal vocal tract (using the corniculate tubercle and the posterior nasal spine)	General	Distance	ACE	IV	0.24 (0.00, 0.74)	0.31 (1)	0.43 (0.00, 0.73)	0.80 (0.77, 0.83)

First, the 14 PMs with the strongest evidence of large and statistically significant of heritability (class I) in bold italic, followed by the 5 PMs of class II in bold, the 6 PMs of class III in italic, and the 16 PMs of class IV in regular font (i.e., we drop here the convention of using italics for the PM names). Within each class, the PMs are ordered by the point estimate of *h*^2^. We also show the point estimate and 95% CIs (in parentheses) for *h*^2^ and *c*^2^ or *d*^2^, and the nominal *p* value (and the Holm-corrected *p* value) of *h*^2^. The precise meaning of *c*^2^ or *d*^2^ is disambiguated by the genetic model. Please see the main text for the meaning of the five classes of strength of evidence, Text S2 and Table S2 for the full description of the PMs, as well as Fig. 3 for a visual representation

Focusing on *h*^2^, there are 41 PMs of at least class IV (14 (34.1%) of class I, 5 (12.2%) class II, 6 (14.6%) class III, and 16 (39.0%) class IV), of types angle (9; 22%), distance (32; 78%) and across domains cervical (1; 2.4%), dentition (2; 4.9%), general (2; 4.9%), hard palate (2; 4.9%), hyoid (4; 9.8%), larynx (2; 4.9%), mandible (8; 19.5%), and skull (20; 48.8%)—see Table S11.

## Discussion and conclusions

We found that the inter-rater agreement is positively correlated with narrow-sense heritability, *h*^2^ (= standardized *A*^2^), and negatively with *e*^2^ (= standardized *E*^2^), indicating that achieving high inter-rater agreement is a very important prerequisite for heritability studies. We found that the skull, the hyoid, and the soft palate, and that angles and distances tend to have the highest *h*^2^ estimates. We ranked our PMs, based on their *h*^2^ point estimates, 95% confidence intervals (CIs), and their statistical significance (nominal at *α*-level 0.05 and corrected for multiple testing), and their inter-rater agreement into five classes, going from those that provide the strongest, to those that virtually give no evidence for high heritability in our data: 14 are class I (i.e., give the strongest evidence), 5 are class II, 6 are class III, 16 are class IV, and 90 are class V (i.e., provide no evidence of narrow-sense heritability). As shown in Tables 1 and 2, and Fig. 3, the measures with the strongest evidence for heritability (class I) concern the skull and the mandible, more precisely the shape and width of the mandible (*MCGP* and *MCGM* capturing its angle, and *MICD* and *MIGD* capturing its width) and its articulation with the skull (ASCG*), the overall length and width of the head (*SHBN* and *SHSW*, respectively), the height of the facial skeleton (*SNAM* and *SNAP*), the basicranium and the nasal cavity (*SSEG* capturing its height, and *SBAN*, *SNNP*, and *SSEN* capturing its length at various points), and the horizontal dimension of the lower face/vocal tract (*SBNA*). Strong evidence (class II) exists for the angle of the facial skeleton (*APNS*), the rest position of the hyoid (*HBC4* on the horizontal and *HBNP* on the vertical), and the horizontal dimension of the lower face/vocal tract (*HBPL* and SVTh*). Class III includes the shape and size of the upper face and nasal cavity (*ASNP* and *AASN* are angles, and *SNOL* is a distance), the posterior size of the mandible (*MCGD* and *MCGR*), and the height of the 2nd cervical vertebra (*CS2A*). Finally, very weak evidence (class IV) exists for the shape of the skull and the nasal cavity (*ANSF*, *ABSN* and *ACSN* are angles, SSEN* is a height, and *SBAS* and *SBNP* and lengths), the width of the hard palate (*DIPD* and *DIMD*), the vertical position of the larynx/epiglottis (*L4EA* and *LAEA*), the position of the hyoid (*HBC2* and *HBPG*), and the length of the vertical supralaryngeal vocal tract (SVTv*), as well as the length of the body of the mandible (*MCPD* and *MPGR*) and the dimension of the nasal cavity floor (*HNSL* and *SBNP*). It can be seen that, reassuringly, measures that are similar by definition tend to have similar heritability estimates (e.g., *SNAP* and *SNAM*, and *MCGP* and *MCGM*).

We aim here to interpret our results in the context of the previous literature. Despite their importance for speech, breathing, and eating, not much is currently known about the heritability of various structures of the human vocal tract, but what is known suggests the existence of a genetic influence on the variation in these structures. There are relatively few studies focusing on the vocal tract per se, and most of the information comes from research focusing on the face or on the whole cranium. While interest in the face has recently increased due to the emergence of very large databases and the computational methods capable of mining them (Cha et al. 2018; Böhringer and Jong 2019), the information about the vocal tract is indirect and contextual, as these studies usually focus on the external, visible properties of the face. Moreover, this literature is still evolving, and there is little general agreement between various publications in what concerns the heritability estimates (for recent reviews, please see (Hoskens et al. 2018; Richmond et al. 2018; Weinberg et al. 2019), but this might be due, at least in part, to differences in methodology (what is measured and how, or twin versus father-offspring studies, for example) and to the sometimes very small samples used. Nevertheless, there seem to exist links with pathologies such as cleft palate/lip, helping to identify specific genetic variants (e.g., for nose width and bizygomatic distance; Boehringer et al. (2011), and also studies using normal samples found notable heritabilities (see, among others, (Savoye et al. 1998; Djordjevic et al. 2013, 2016; Tsagkrasoulis et al. 2017; Hoskens et al. 2018)) and several genetic loci (e.g., (Liu et al. 2012; Paternoster et al. 2012; Lee et al. 2017; Cha et al. 2018; Crouch et al. 2018; Indencleef et al. 2018)). While this literature is hard to briefly summarize, when it comes to findings potentially relevant to the vocal tract, it seems that relatively strong genetic influences exist for aspects of nose shape (as well as possibly for maxillary and mandibular prominences and particularly the chin), that the vertical dimensions may have a higher heritability, and that the environment seems to affect more the mandible and the lower face, though different results literally point in different directions, and the heritability estimates range from very small (~ 0.20; please note that these can be equivalently expressed as percents, 20%, but we opted on converting everything to proportions here) to medium (~ 0.30 to 0.60) and high (> 0.70). Our data are relatively consonant with these findings, in that we also found that the face and skull have notable heritabilities, as does the mandible.

There are even fewer studies looking at not-externally visible measures or measures not related to the face, they use very different methodologies and samples, and the picture they paint is also complex (see, for example, (Lundström and McWilliam 1987; Martínez-Abadías et al. 2009; Chi et al. 2014; Šešelj et al. 2015; Švalkauskienė et al. 2015; Šidlauskas et al. 2016)). Again, it seems that some facial measures tend to have the highest heritabilities, while structures of the vocal tract seem to have relatively low-to-moderate heritabilities. For example, Chi et al. (2014) report heritabilities for mandibular length (0.24), mandibular width (0.30), maxillary width (0.47), the distance from the hyoid bone to the retropogonion (0.36; but not for other hyoid distances), and the size of the oropharyngeal space (0.31); Šešelj et al. (2015) report moderate heritabilities for maxillary and mandibular measures; Šidlauskas et al. (2016) report that the heritability of the shape of the mandible is higher than for its size; Švalkauskienė et al. (2015) look at dental arches and find that widths at the back have lower heritabilities than at the front, that the upper jaw has higher heritabilities than the lower, and that length and width seem independent; finally, Martínez-Abadías et al. (2009) found that there are 6 “phenotypic modules” (oro-nasal, molar, orbital, zygomatic-pterygoid, neurocranial vault, and basicranium) with low-to-moderate heritabilities (0.0–0.43). Likewise, various measures of anatomical structures of the vocal tract, such as the teeth (and face) (Hughes et al. 2014), the upper airways (Patel et al. 2008), and the hard palate (Shapiro 1969; Riquelme and Green 1970) may be more similar in MZ than DZ twins. Moreover, various characteristics of the voice that may be affected by anatomy and physiology (e.g., the motor control of the vocal tract), such as the fundamental frequency (Przybyla et al. 1992; van Gysel et al. 2001; Debruyne et al. 2002), seem to be more similar in monozygotic than in dizygotic twins (Forrai and Gordos 1983; Nolan and Oh 2013). Here, our findings extend these earlier reports; in that we focus specifically on not-externally visible aspects of the vocal tract. In particular, we found notable heritabilities for the shape and size of the basicranium and the nasal cavity, the length of the hard palate, the horizontal and vertical dimensions of the vocal tract (but less so for its width and shape), the width, shape, and relative position of the mandible, and the position of the hyoid/larynx.

The effects of environmental factors are of particular interest here, as it is known that biological structures (including the human vocal tract) are very plastic (West-Eberhard 2003), and that, for example, surgical and orthodontic interventions can sometimes have massive remodeling effects even affecting relatively distant structures (e.g., the palatal rugae (Mustafa et al. 2015), the form of the dental arch (Daou et al. 2020)), that digit sucking during childhood changes the shape of the hard palate (Yemitan et al. 2013), as supposedly does tongue activity (at least as suggested by individuals affected by Down Syndrome; Skrinjarić et al. (2004), Klingel et al. (2017)), that the shape of the nasal floor is affected by dentition (Nicholas and Franciscus 2014), and that food-related practices influence, among others, the lower jaw (von Cramon-Taubadel 2011) and the dental occlusion (Blasi et al. 2019). Moreover, the genetic and environmental factors interact in complex ways, and characteristics of our sample may further affect the heritability estimates, which must, therefore, be interpreted with care (Visscher et al. 2008). For example, the high heritability of aspects of the mandible does not necessarily contradict the findings that cross-cultural variation in food consistency affects jaw growth and dental occlusion (von Cramon-Taubadel 2011), while the lack of similar findings for the length and width of the hard palate might be artifacts of dental and orthodontic treatments, particularly popular in the Netherlands, and usually highly clustered within families irrespective of twin status.

Seen in the context of this existing literature, our study adds further evidence that the genetic and environmental (including cultural) factors interact in shaping the vocal tract, highlighting that each structure (and even parts of a structure) has its own constellation of interactions. For example, the resting position of the hyoid/larynx seems to be under unexpectedly strong genetic influences, while various bony components of the vocal tract (such as the dental arches and the hard palate) seem to be highly plastic, changing in shape under the influence of orthodontic interventions, food consistency, and even digit sucking (though other bony structures seem to be quite resilient to such influences, such as the nasal cavity and the mandible). This suggests that future genetic association studies might focus on those structures and measures that show high heritability, but it will also be extremely interesting to understand the (epi)genetic mechanisms involved in the changes in shape in response to various environmental factors, so obvious in some structures of the vocal tract.

While it is very difficult to translate these anatomical findings into their effects on voice idiosyncrasies and speech articulation, our results may offer intriguing windows into the complex relationships between genes, environment, cultural practices, and language (Dediu 2015; Dediu et al. 2017). Recent work has shown that the precise shape of vocal tract structures may affect the speech of individuals (either as pathological productions (Kummer 2014), or as idiosyncrasies and normal variation (Dediu and Moisik 2019)), and may even result in differences between languages (Moisik and Dediu 2017). The nasal cavity may affect the production of nasal consonants (such as “m” and “n”) and nasalised vowels (such as “on” in French), but little is currently known about the influence of its detailed anatomy on speech. A smaller nasopharynx seems to be associated with a high incidence of Chronic Otitis Media (COM) in children (Maw et al. 1991), and it has been suggested that a high incidence in COM among Australian Aborigene children, resulting in widespread partial hearing loss among them, explains certain typologically rare properties of the Australian languages, such as an absence of fricatives and the presence of many place of articulation distinctions (Butcher 2018). The jaws and dentition are probably implicated in the production of labiodental sounds (such as “f” and “v” in English) through their effects on bite (Blasi et al. 2019; Everett and Chen 2021). While this effect was established at the cross-cultural level, and is driven by in vivo changes during development (and beyond) due to variation in the mechanical properties of food (“soft” versus “hard”, broadly representative for the agricultural and the hunter-gathering subsistence strategies, respectively), it is interesting to also consider the genetic factors subtending variation in their shape, dimensions, and inter-relationships, and their responses to the properties of food. However, by far the best studied are the effects of the hard palate dimensions and shape on speech production: for example, these affect the general articulatory variability during speech (Brunner et al. 2009), the production of vowels (Dediu et al. 2019), and of the North American English “r” (Dediu and Moisik 2019), and the alveolar ridge may affect clicks (Moisik and Dediu 2017). Most of these examples start from inter-individual variation, but assume that this is patterned between populations, and that these patterns are relatively stable at the scale of language change, i.e., for several generations (Dediu et al. 2017, 2019). Such patterning and stability can be due to persistent environmental and cultural factors (such as climate or subsistence strategies), but genetics is also expected to contribute.

It is important to stress that our mega-sample rests on the availability of MRI data acquired from twin pairs in five studies, none of which was specifically designed to investigate the anatomy of the vocal tract. In fact, the realization that the already collected MRI structural scans contained high-quality data for the lower part of the head in a majority of their participants was a real surprise. The landmarking of these data by two raters allowed us to quantify the inter-rater agreement for each of the 146 unique primary measures (or PMs), and we found that the domains of the mandible, the skull, the “general” and the hyoid, and the measures of type distance and angle tend to have the highest agreements. Moreover, we identified 15 measures that violate the assumptions of the parametric models we use to a degree that required their removal from the final results.

As mentioned above, our mega-sample is composed of participants from several MRI projects, with about 65% females overall. Age, and birth cohort, which covers a large spectrum (11–93.5 years) does not differ between the sexes, but varies widely between studies. Head size (proxied by the intra-cranial volume or ICV) ranges between 890 and 1890.2 cm^3^, which shows the expected difference between sexes, and also somewhat between studies (probably due to differences in composition with respect to age and sex ratio). After controlling for the effects of these covariates (age, sex, and intra-cranial volume) for each PM, we fitted a genetic covariance structure modeling (or GCSM) to the data of the two raters simultaneously, that includes the additive genetic influences (*A*), the common environmental circumstances (*C*), non-additive genetic or dominance influences (*D*), and the unique environmental effects (and measurement error) (*E*). Our mega-sample contains relatively few DZ twin pairs (26.8%), of which very few are sex-concordant (12.1%), and there is a large discrepancy across studies (three have very few or virtually no DZ twins). While we do pool these studies together and employ advanced statistical models, this unbalanced design, due to the nature of the pre-existing studies, could still affect our estimates of the variance components, probably not in terms of their point estimates (the estimated central tendencies are unbiased), but in terms of the spread of their uncertainty (they have wider confidence intervals and larger standard errors). Moreover, this probably reduces our power to detect statistically significant variance components, but this only makes our exploratory study more conservative.

Our mega-sample may have certain characteristics that make the direct generalization of our results difficult, it being, on the one hand, quite uniform in some respects (e.g., medical care, nutrition), while, on the other (e.g., orthodontic treatment), being clustered within families, potentially leading to, respectively, artificially inflated or deflated heritability estimates (Visscher et al. 2008). Even so, our heritability estimates are broadly in line and consistent with the previous literature concerning the face and the skull. Thus, while our results cannot emphatically be directly generalized to other groups and contexts, and neither to explaining between-group variation, they do suggest that there is a genetic component influencing to varying degrees different aspects of the vocal tract, but, critically, that these genetic influences must be understood in the context of their complex interplay with environmental factors and cultural practices, the latter probably particularly important for the development and life trajectory of the human vocal tract.

## Supplementary Information

Below is the link to the electronic supplementary material.

Supplementary file1 (PDF 1853 kb)

Supplementary file2 (XZ 31532 kb)

## Data Availability

The computer code for performing the analyses (R, OpenMX, lavaan and Rmarkdown) and the full results (HTML) are available in the GitHub repository https://github.com/ddediu/vt-heritability under a GPLv3 license. The MATLAB code of VTANALYZER is available in the GitHub repository https://github.com/ScottMoisik/VTANALYZER under an MIT license. The primary data are available upon request from the Netherlands Twin Register (https://tweelingenregister.vu.nl/information_for_researchers/working-with-ntr-data).
