# Supplementary material for: The heritability of vocal tract structures estimated from structural MRI in a large cohort of Dutch twins
Source: Hum Genet. 2022 Jul 13;141(12):1905–23. doi: 10.1007/s00439-022-02469-2 (PMC9672028; doi:10.1007/s00439-022-02469-2)
Supplement: Supplementary file 1 — Supplementary file1 (PDF 1853 kb) [file 439_2022_2469_MOESM1_ESM.pdf]

Supplementary Information for: The heritability of vocal tract structures estimated from structural MRI in a large cohort of Dutch twins

Dan Dediu,[1,2,3,*,†] Emily M. Jennings,[4,5,†] Dennis van 't Ent,[5] Scott R. Moisik,[6] Grazia Di Pisa,[7] Janna Schulze,[8] Eco J.C. de Geus,[5] Anouk den Braber,[5,9] Conor V. Dolan,[5] Dorret I. Boomsma[5]

[1] Department of Catalan Philology and General Linguistics, University of Barcelona, Barcelona, Spain

[2] Universitat de Barcelona Institute of Complex Systems (UBICS), Barcelona, Spain

[3] Catalan Institute for Research and Advanced Studies (ICREA), Barcelona, Spain

[4] Faculty of Linguistics, Philology and Phonetics, University of Oxford, Oxford, UK

[5] Department of Biological Psychology, Vrije Universiteit Amsterdam, The Netherlands

[6] Linguistics and Multilingual Studies, Nanyang Technological University, Singapore

[7] Department of Linguistics, Universität Konstanz, Konstanz, Germany

[8] Independent researcher

[9] Department of Neurology & Alzheimer Center, Neuroscience Amsterdam, Amsterdam UMC, Vrije Universiteit Amsterdam, The Netherlands

[†] These authors contributed equally

[*] Corresponding author: Dan Dediu

Correspondence: dan.dediu@icrea.cat

Table of contents

Supplementary texts

- Text S1: Sources of missing data
- Text S2: Description of the Phenotypic Measures (PMs)
- Text S3: Modeling rater agreements and disagreements
- Text S4: Re-implementation in `lavaan`

Supplementary figures

- Fig. S1. Inter-rater agreement
- Fig. S2. Pairwise scatterplots between the estimates of the variance components and inter-rater agreement
- Fig. S3. The distribution of h^2, c^2, d^2 and e^2
- Fig. S4. Midsagittal view of h^2 *class I* PMs
- Fig. S5. Midsagittal view of h^2 *class II* PMs
- Fig. S6. Midsagittal view of h^2 *class III* PMs
- Fig. S7. Midsagittal view of h^2 *class IV* PMs
- Fig. S8. Mandible view of h^2 *class I* PMs
- Fig. S9. Mandible view of h^2 *class II* PMs
- Fig. S10. Mandible view of h^2 *class III* PMs
- Fig. S11. Mandible view of h^2 *class IV* PMs
- Fig. S12. Hard palate view of h^2 *class I* PMs
- Fig. S13. Hard palate view of h^2 *class II* PMs
- Fig. S14. Hard palate view of h^2 *class III* PMs
- Fig. S15. Hard palate view of h^2 *class IV* PMs

Supplementary tables

- Table S1. The list of landmarks and semilandmarks
- Table S2. The list of all phenotypic measures (PMs)
- Table S3. Pairs of very highly correlated PMs
- Table S4. The number of PMs supporting the 2r vs the 1r models
- Table S5. The full results
- Table S6. Relationship between the ΔAIC with cut-off 2 and the likelihood ratio test (LRT) at α-level 0.05
- Table S7. Correlations between the estimates of interest
- Table S8. Classes of strength of evidence for narrow-sense heritability h^2
- Table S9. Classes of strength of evidence for c^2
- Table S10.Classes of strength of evidence for d^2
- Table S11. The distribution of the PMs of h^2 class at least *IV*

Supplementary references

Other supplementary materials for this manuscript include the separate full analysis report (a compressed `HTML` document). The computer code for performing the analyses (`R`, `OpenMX`, `lavaan` and `Rmarkdown`) and the full results (`HTML`), are available in the `GitHub` repository https://github.com/ddediu/vt-heritability under a `GPLv3` license. The `MATLAB` code of `VTANALYZER` is available in the `GitHub` repository https://github.com/ScottMoisik/VTANALYZER under an `MIT` license. The primary data is available upon request from the Netherlands Twin Register (https://tweelingenregister.vu.nl/information_for_researchers/working-with-ntr-data).

Supplementary texts.

Text S1: Sources of missing data.

There are multiple reasons for which various landmarks and semilandmarks might be missing from particular MRI scans, an important one being signal disruption from braces, alongside the actual coverage of the scanning region. The latter affects many measurements (especially those involving the cervical spine). Looking at missing landmark counts, we see the following patterns (given as % of total landmarking sessions, where a session is either of the two raters' landmarking of one scan):

- *Cervical spine*: there is an increasing cascade from C2 to C7, as follows: C2 (absent from 1.4% of the sessions), C3 (10.5%), C4 (29.2%), C5 (69.5%), C6 (93.2%) and C7 (99.6%);
- *Dentition*: missing landmarks cascade from the back to the front, with paired landmarks (left/right) showing similar absence rates, suggesting the effects of braces and of the anterior extension of the scanning region: second molars (absent from 19.3% of the sessions), second premolars (25.1%), canines absent (25.6%), and incisors (24.8%);
- *Non-dentition anterior landmarks*: these might have been impacted by braces, like the anterior dentition landmarks, or the scanning region being set too posterior to capture some protruding structures of the face or even too superior to capture landmarks such as the menton; it is telling that the nasion (the frontal and nasal bone meeting point), however, is absent from only 0.2% of the sessions, reflecting the constraints of MRI brain scanning: menton (absent from 18.7% of sessions), anterior nasal spine (11.3%), and pogonion (14.5%);
- *Larynx and hyoid*: like for the landmarks of the cervical vertebrae, lower landmarks have more severe absence rates:: apex of the epiglottis (absent from 5.4% of the sessions), hyoid (17.7%), corniculate tubercle (37.7%), and petiole of the epiglottis (48.0%).

Please note that, by definition, the landmarks necessary for `VTANALZER` to work (head top, head back, nose tip, head side (right), and head side (left)) are present in all sessions. Furthermore, it can be seen that, in general, landmarks closer to the brain have very low absence rates (sella, basion, odontoid, condyles, nasal spine posterior, uvula, gonion, and inferior border of the eye orbits), again reflecting the design of the original studies.

Text S2: Description of the Phenotypic Measures (PMs).

The phenotypic measures (PMs) listed in **Table S2** were derived from the (semi)landmarks listed in **Table S1** using an automated method implemented using `MATLAB` and the support of freely available scripts for performing various geometric operations across the various measures Schwarz (2022). The objective in designing the PMs was to provide a comprehensive coverage of the vocal tract dimensions and shape, in as much as the landmark set would allow, with Howells (1973) providing general inspiration, and with many of the measurement definitions drawn directly from several sources. Cervical spine measurements *CS3H*, *CS4H*, *CS5H*, *CS6H*, *CS7H*, and *CS2A* are based on Grave et al. (1999). Measurements *HBC3*, *PNNP*, *HNSL*, *SPUN*, *DICD*, *DIPD*, *DIMD* are based on Alfwaress et al. (2015), with *DICD*, *DIPD*, and *DIMD* also following Reenen and Allen (1987). Measurements for the skull, including several distances (*SBAS*, *SBAN*, *SBAP*, *SNNA*, *SNNP*, *SNAM*, and *SSEN*) and angles (*ABSN*, *ACSN*, *AASN*, *AANP*, *APNS*, *APNP*, *ASCG*, *ASCG*, *ANSF*, *ANNF*, and *ALNS*) are based on Solow et al. (1982). Finally, we also drew inspiration from Nishimura et al. (2006) in defining the measurements characterizing the horizontal and vertical lengths of the vocal tract, *SVTh*, *SVTh**, *SVTv*, *SVT**. The other measures are based on our own design or represent simple extensions of measures listed here using slightly different landmarks (e.g., we added *HBC2* and *HBC4* as extensions of *HBC3* noted above).

Text S3: Modeling rater agreements and disagreements.

The two raters coded the same MRI scans independently, following the same training guidelines and using the same platform. However, they did discuss complex cases between them. To model the rater effects, we need to decide if the raters have the same error variance. While the fully unconstrained model fitted to the data would arguably be the least *a priori* biased, this model includes many parameters to estimate, which is potentially problematic given our sample size. Before seeing the data, based only on the organization of the training and of the landmarking process, supplemented by personal observations and debriefing interviews with the raters during and after the landmarking, the most constrained model (i.e., same latent means and error variances) would be favored. After having obtained the data (here, the estimates of the PMs for each of the two raters and not the actual (semi)landmarks), we first examined

the correlations and the differences between the two raters for the corresponding participants and measures, and we found that the two raters tend to agree on the means of the measurements and to have medium to high correlations, suggesting that the latent means might indeed be equal. Finally, for each PM, we fitted two GCSM models in `OpenMX` (see below for details), one allowing different error variances for the two raters (the "2r" model) and another constraining the error variances to be equal (the "1r" model). These models were compared using both Akaike's Information Criterion (AIC; corrected with the "Parameters Penalty", which uses the number of free parameters in the model rather than the degrees of freedom) and the likelihood ratio test. Crucially, we used the overall results across the measures to decide between which one of the 1r or the 2r models to apply uniformly to all measures, therefore not inflating the number of multiple tests performed between measures. We found that none of the measures supported the 2r model, with the two models being equivalent for a majority of the measures, and the few remaining ones supporting the 1r model (see **Table S3**). Taking all these together and preferring simplicity, we decided to implement the 1r model throughout (see **Figure 2** in the paper).

Text S4: Re-implementation in `lavaan`.

As an extra check, we re-implemented the GCSM model in `lavaan` (Rosseel 2012); for full details, please see the **full `HTML` report**. However, this re-implementation differs from the one in `OpenMX` mainly in that the covariates were not included in the GCSM model, but were regressed out from the PMs previous to fitting the GCSM model. Moreover, we implemented both the ACE and the ADE models, and we chose between them using Akaike's Information Criterion, AIC, and not by comparing the latent phenotypic correlations r_{MZ} ans $2r_{DZ}$. `lavaan` returns the standardized estimates with standard errors, 95% confidence intervals, and *p*-values, but for the *A*, *C* and *D* components, we also performed model comparison, using AIC and the likelihood ratio test for nested models, between the "full" model and the "reduced" model without the component of interest. With these, we obtained, for all the PMs, the fitted model (ACE or ADE) and the standardized point estimates, standard errors, 95%CIs, *p*-values and (for *A*, *C* and *D*) the ΔAIC and *p*-value of the LR test, which we compared with their corresponding main `OpenMX` estimates.

First, even if the choice between the ACE and ADE genetic models are based on different approaches, for 127 (87.0%) PMs, the chosen genetic model is the same, but for virtually all the remaining PMs, the `lavaan` ACE and ADE models are virtually indistinguishable (and that for some, even the `OpenMX` latent r_{MZ} and $2r_{\mathrm{DZ}}$ are also very close), suggesting that the differences between the genetic models are very superficial.

Second, there is a strong positive correlation between the narrow-sense heritability estimates from `OpenMX` and `lavaan` across all PMs (Pearson's $r = 0.82$, $p = 2.08\times10^{-37}$ and Spearman's $\rho = 0.82$, $p = 2.39\times10^{-36}$), and the PMs for which the two pick different genetic models (ACE vs ADE) do not seem to be particularly divergent. The linear regressions of one on the other have a high adjusted R^2 of 67.7%, and are very close to identity:

lavaan = 0.07 (±0.04, $p = 1.56\times10^{-4}$) + 0.80*OpenMX* (±0.09, $p = 2.08\times10^{-37}$)

and

OpenMX = 0.03 (±0.04, $p = 0.076$) + 0.85*lavaan* (±0.10, $p = 2.08\times10^{-37}$)

Therefore, these two different implementations support each other, but given the limitations of our `lavaan` implementation, we used the `OpenMX` results throughout.

Supplementary figures.

Fig. S1. Inter-rater agreement.

Fig. S1. Inter-rater agreement, *ICC*(*C*,1), by *domain* (left) and *type* (right), ordered by increasing median agreement from top to bottom. We shows that actual points as well as the boxplots. Generated automatically using `R` 4.1.3 (https://www.r-project.org/).

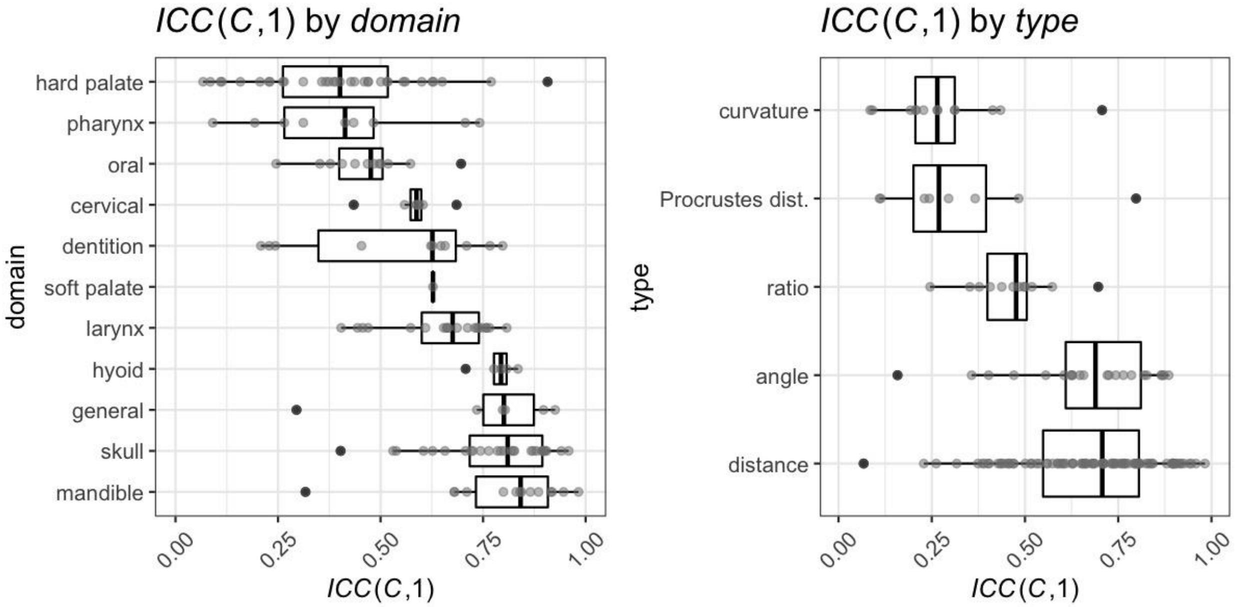

Fig. S2. Pairwise scatterplots between the estimates of the variance components and inter-rater agreement.

Fig. S2. Pairwise scatterplots between the estimates of the variance components (h^2, c^2, d^2 and e^2) and inter-rater agreement, *ICC*(*C*,1), showing the actual points as well as the linear (blue) and loess (red) fits (with 95% confidence intervals as the gray areas). Generated automatically using `R` 4.1.3 (https://www.r-project.org/).

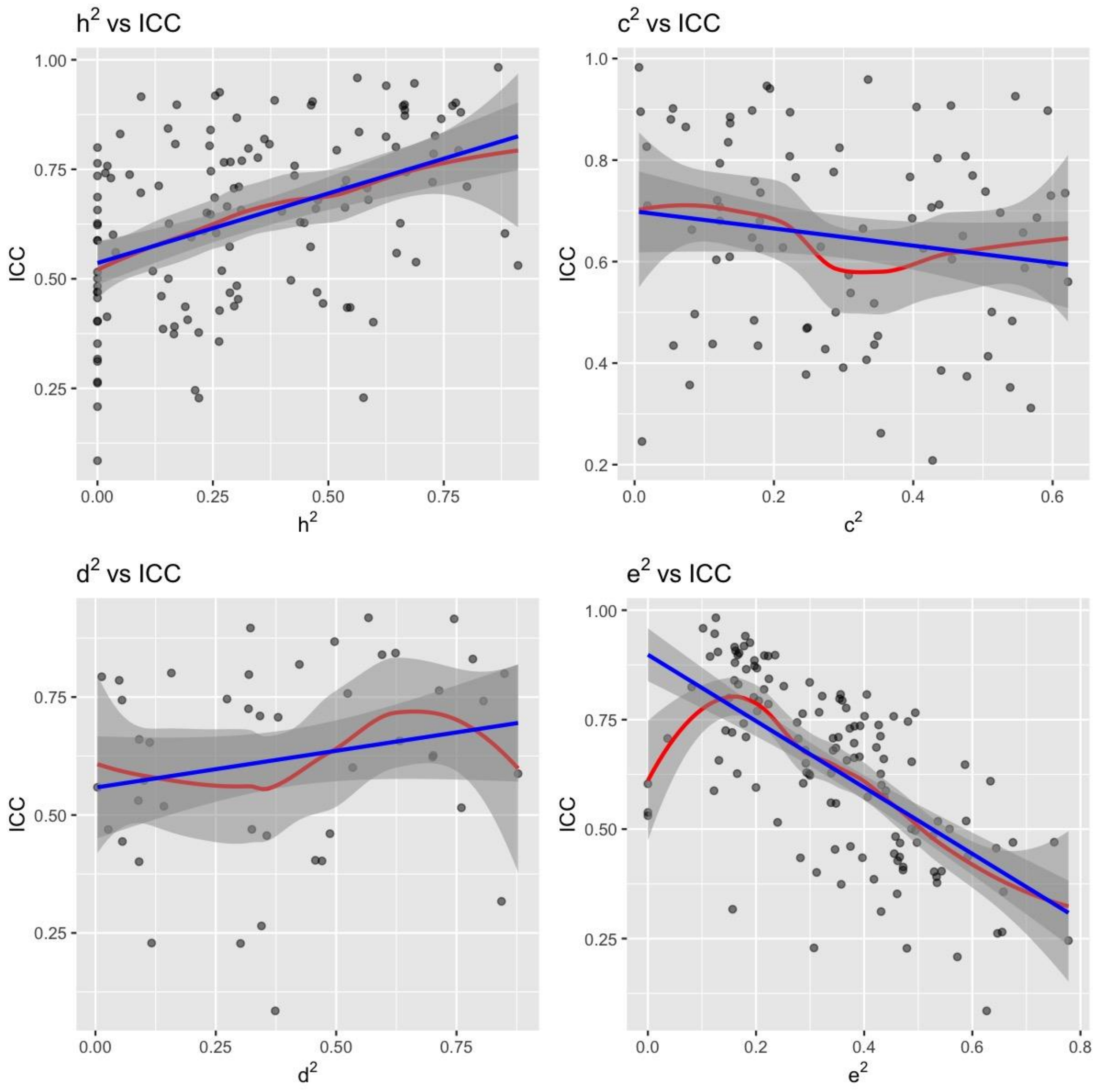

Fig. S3. The distribution of h^2, c^2, d^2 and e^2.

Fig. S3. The distribution of h^2, c^2, d^2 and e^2 across *domains* (left-hand side panels) and *types* (right-hand side panels), showing the actual point estimates for each PM in each domain/type as well as the corresponding boxplots, and the important values of 0.0, 0.2 and 1.0. Generated automatically using `R` 4.1.3 (https://www.r-project.org/).

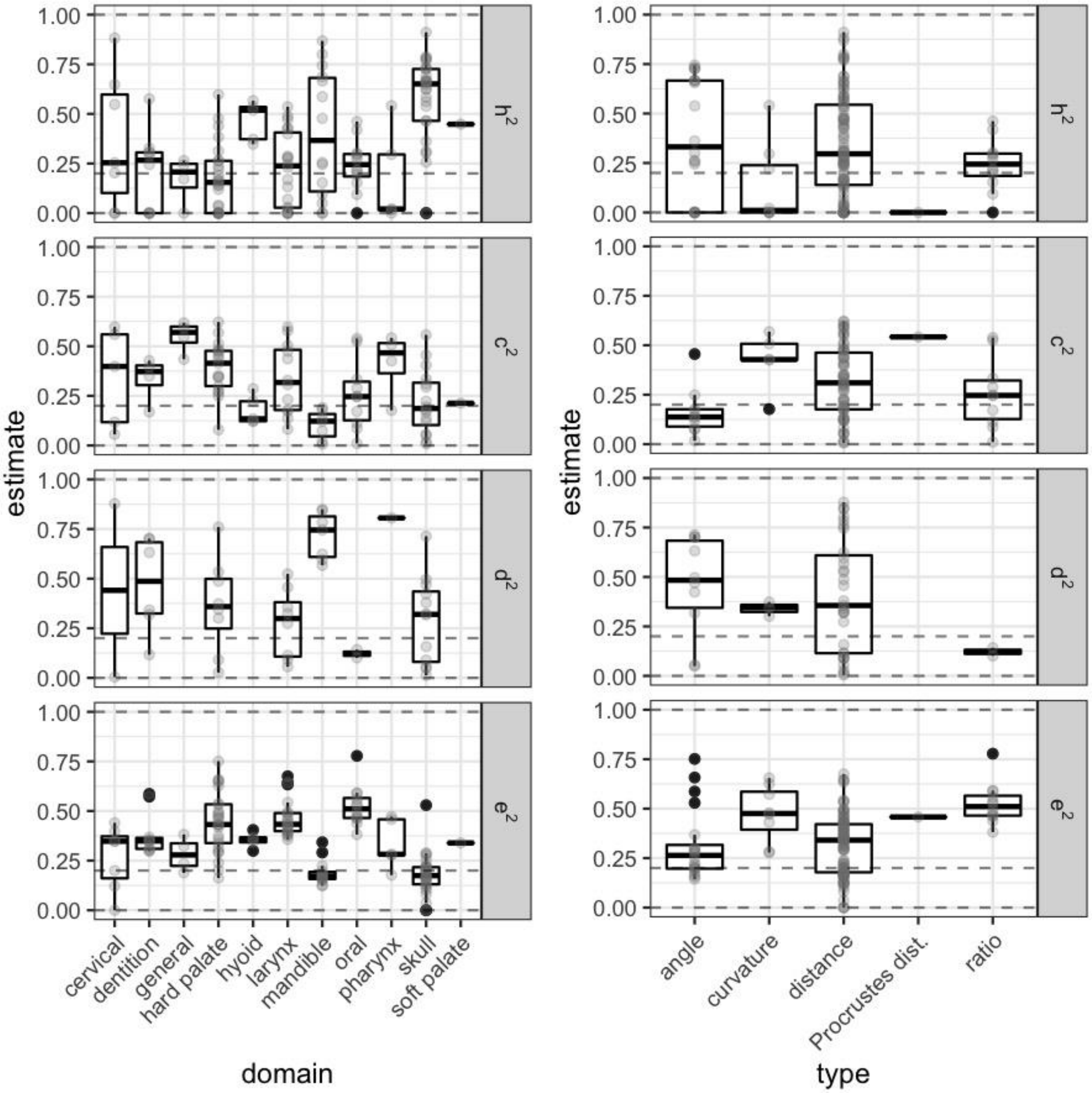

Fig. S4. Midsagittal view of h^2 *class I* PMs.

Fig. S4. Midsagittal view of h^2 *class I* PMs (i.e., PMs with very strong evidence for high narrow-sense heritability coupled with high inter-rater agreement). This is **Panel A** of **Figure 3** in the main text. Please see **Figure 3** in the main text for conventions.

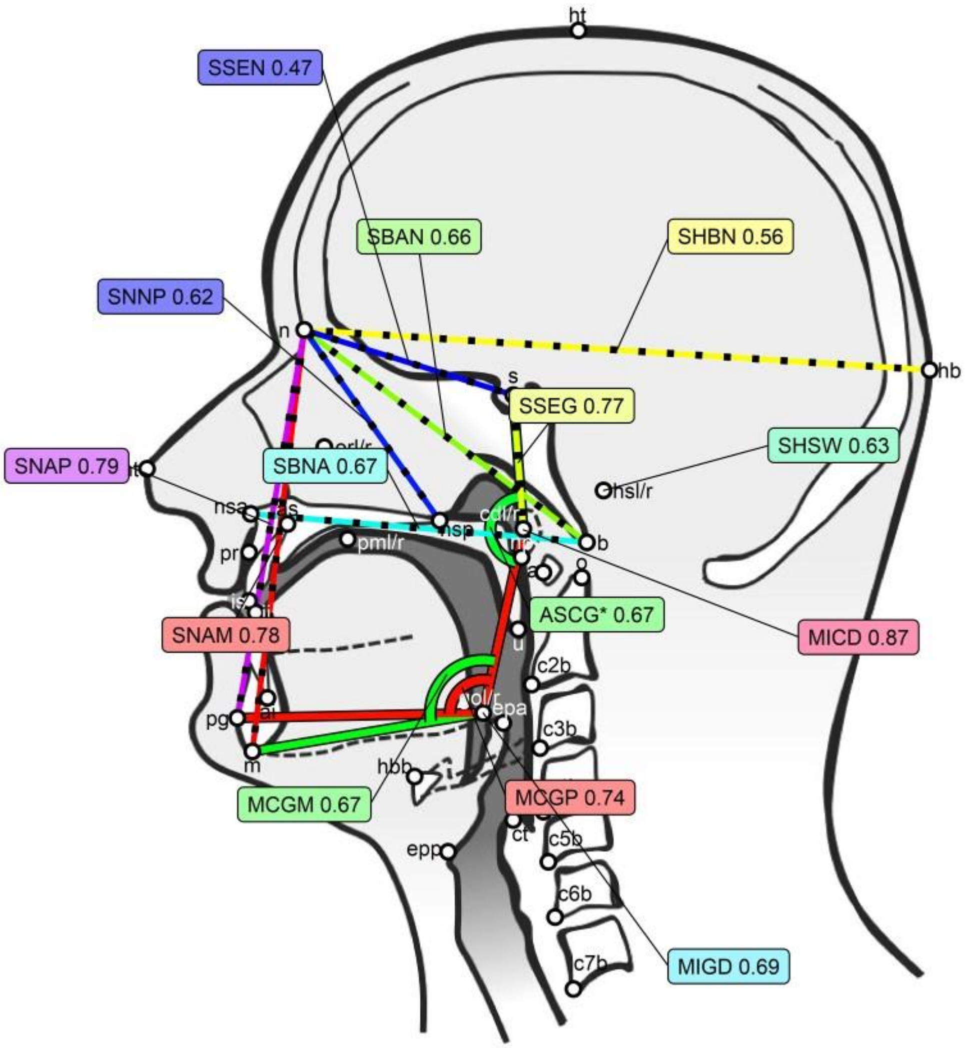

Fig. S5. Midsagittal view of h^2 *class II* PMs.

Fig. S5. Midsagittal view of h^2 *class II* PMs (i.e., PMs with relatively high narrow-sense heritability, coupled with good inter-rater agreement). This is **Panel B** of **Figure 3** in the main text. Please see **Figure 3** in the main text for conventions and **Text S2** and **Table S2** for the information about the PMs.

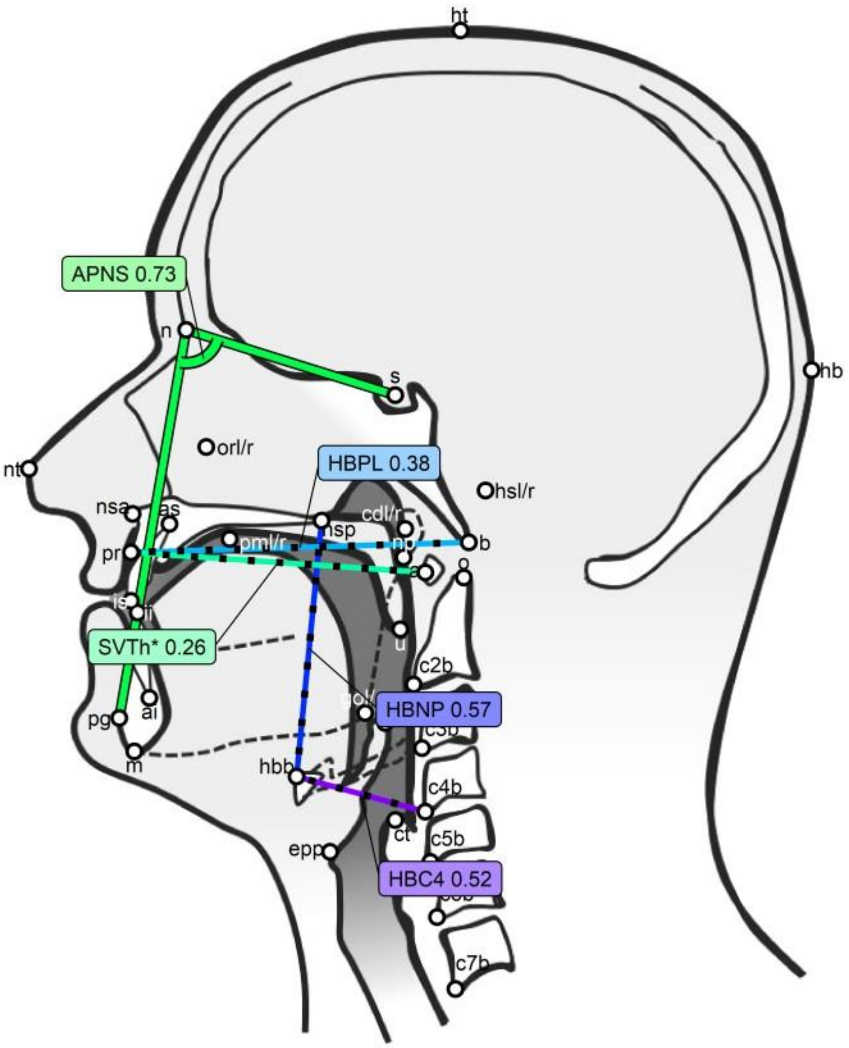

Fig. S6. Midsagittal view of h^2 *class III* PMs.

Fig. S6. Midsagittal view of h^2 *class III* PMs (i.e., PMs with relatively high narrow-sense heritability, but lacking inter-rater agreement). This is **Panel C** of **Figure 3** in the main text. Please see **Figure 3** in the main text for conventions and **Text S2** and **Table S2** for the information about the PMs.

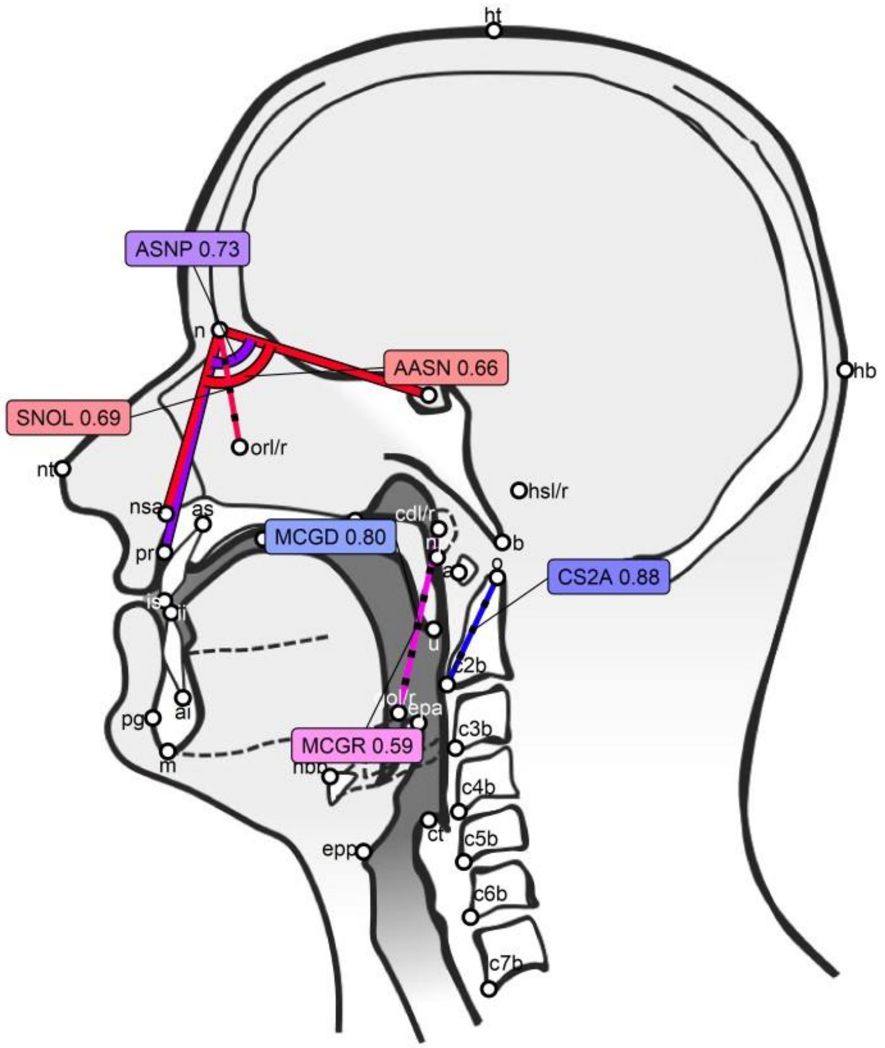

Fig. S7. Midsagittal view of h^2 *class IV* PMs.

Fig. S7. Midsagittal view of h^2 *class IV* PMs (i.e., PMs with circumstantial evidence of narrow-sense heritability, irrespective of inter-rater agreement). Please note that *ANSF* is not shown. This is **Panel D** of **Figure 3** in the main text. Please see **Figure 3** in the main text for conventions and **Text S2** and **Table S2** for the information about the PMs.

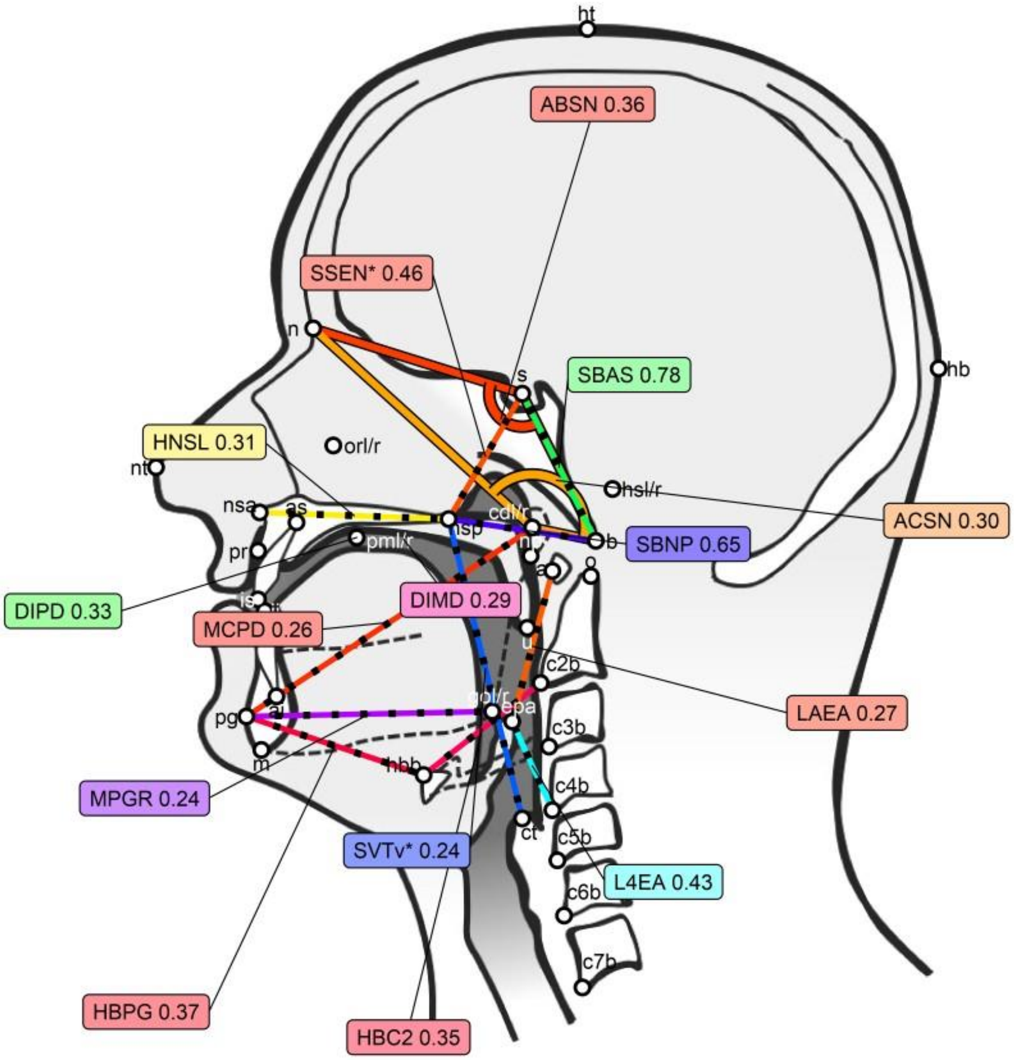

Fig. S8. Mandible view of h^2 *class I* PMs.

Fig. S8. Mandible view of h^2 *class I* PMs (i.e., PMs with very strong evidence for high narrow-sense heritability coupled with high inter-rater agreement). This is **Panel E** of **Figure 3** in the main text. Please see **Figure 3** in the main text for conventions and **Text S2** and **Table S2** for the information about the PMs.

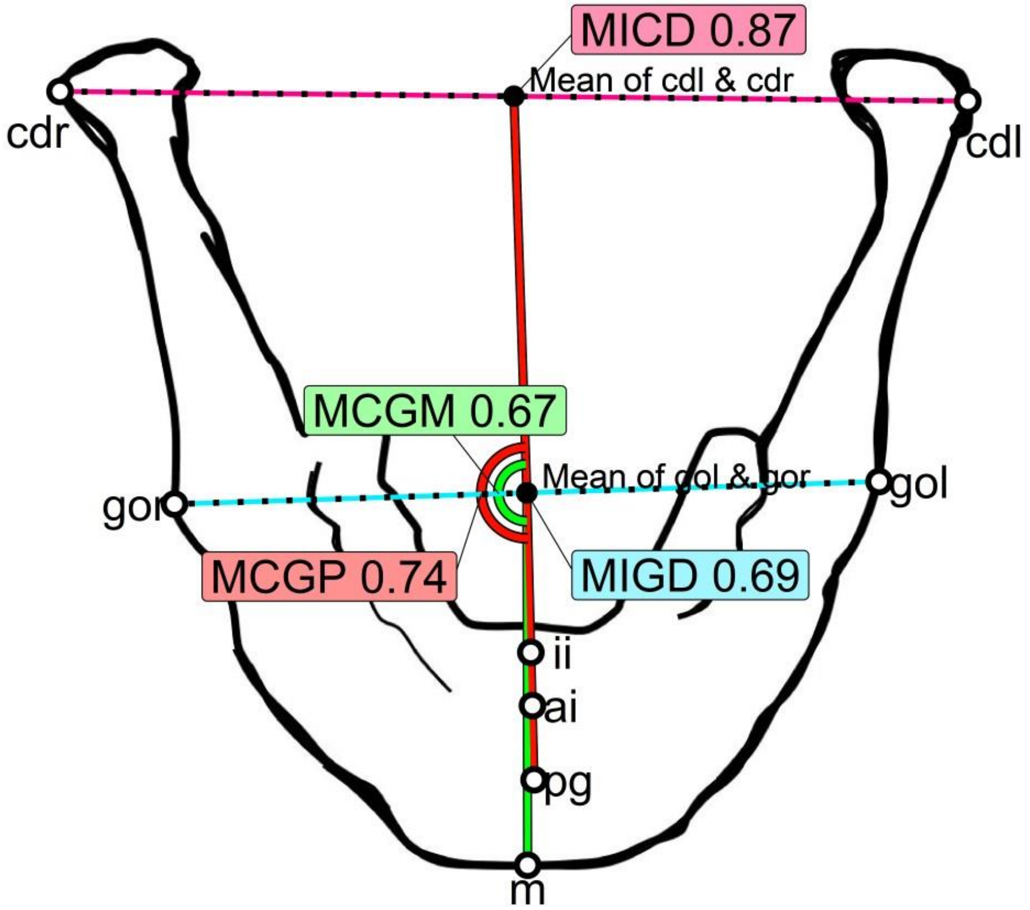

Fig. S9. Mandible view of h^2 *class II* PMs.

Fig. S9. Mandible view of h^2 *class II* PMs (i.e., PMs with relatively high narrow-sense heritability, coupled with good inter-rater agreement). This is not shown in **Figure 3** in the main text. Please see **Figure 3** in the main text for conventions and **Text S2** and **Table S2** for the information about the PMs.

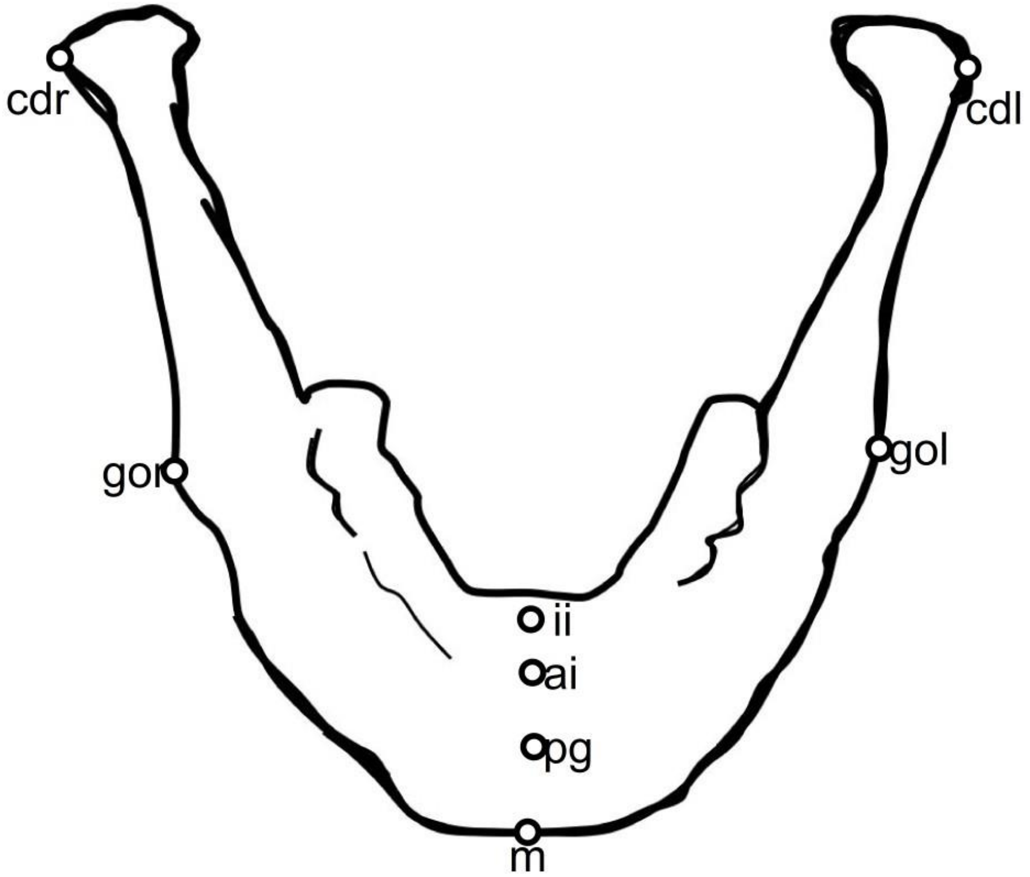

Fig. S10. Mandible view of h^2 *class III* PMs.

Fig. S10. Mandible view of h^2 *class III* PMs (i.e., PMs with relatively high narrow-sense heritability, but lacking inter-rater agreement). This is **Panel F** of **Figure 3** in the main text. Please see **Figure 3** in the main text for conventions and **Text S2** and **Table S2** for the information about the PMs.

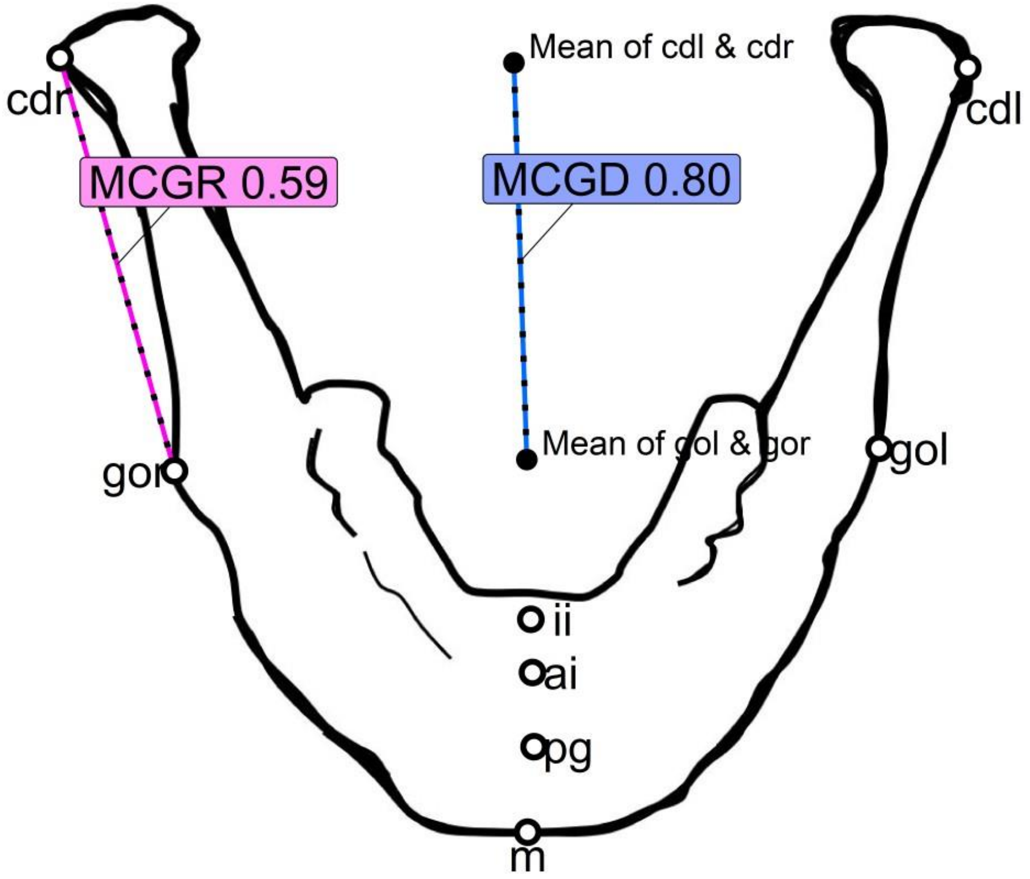

Fig. S11. Mandible view of *h*² *class IV* PMs.

Fig. S11. Mandible view of h^2 *class IV* PMs (i.e., PMs with circumstantial evidence of narrow-sense heritability, irrespective of inter-rater agreement). This is **Panel G** of **Figure 3** in the main text. Please see **Figure 3** in the main text for conventions and **Text S2** and **Table S2** for the information about the PMs.

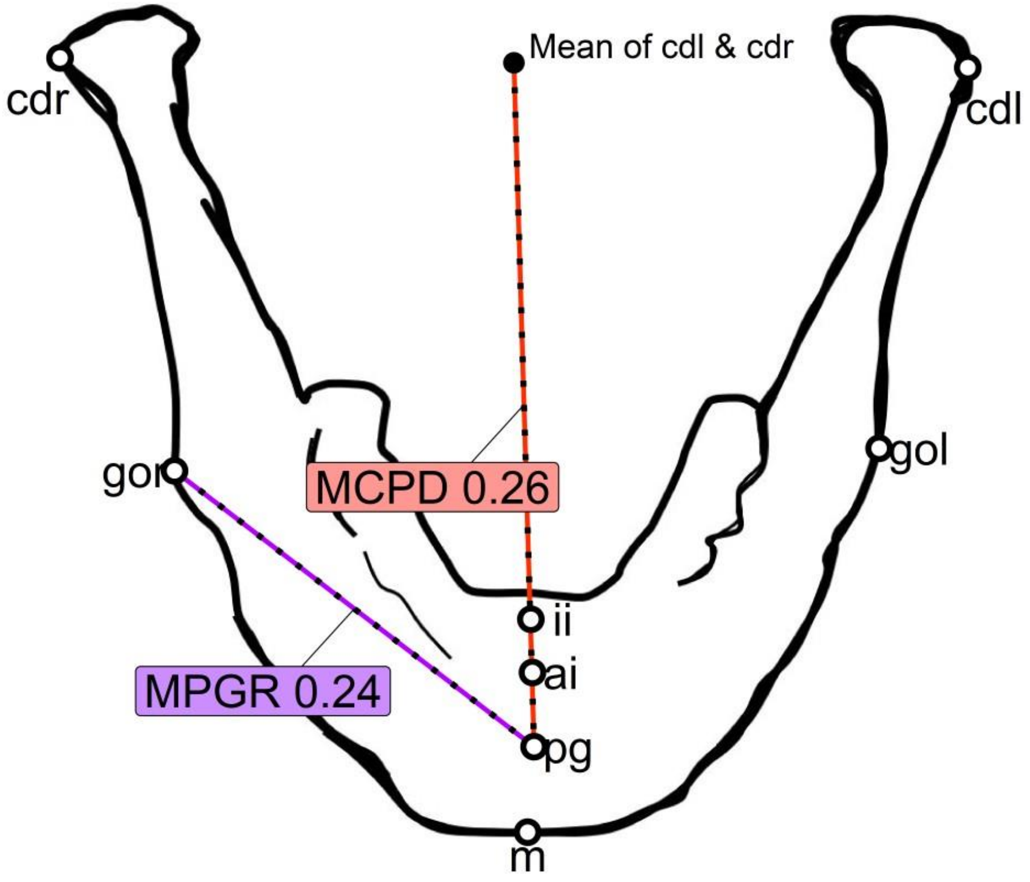

Fig. S12. Hard palate view of h^2 *class I* PMs.

Fig. S12. Hard palate view of h^2 *class I* PMs (i.e., PMs with very strong evidence for high narrow-sense heritability coupled with high inter-rater agreement).This is not shown in **Figure 3** in the main text. Please see **Figure 3** in the main text for conventions and **Text S2** and **Table S2** for the information about the PMs.

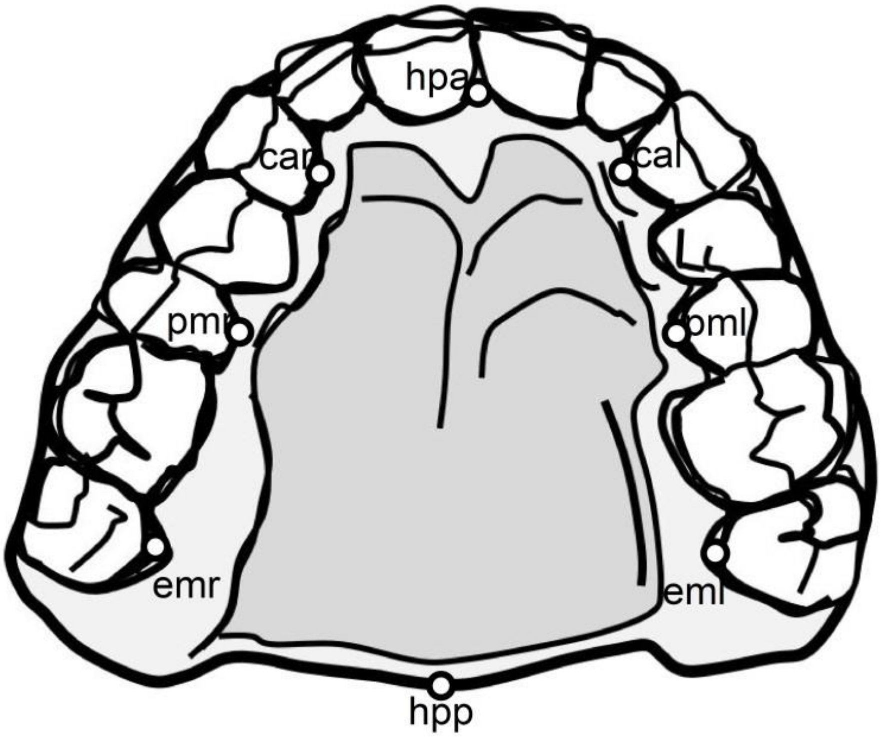

Fig. S13. Hard palate view of h^2 *class II* PMs.

Fig. S13. Hard palate view of h^2 *class II* PMs (i.e., PMs with relatively high narrow-sense heritability, coupled with good inter-rater agreement). This is not shown in **Figure 3** in the main text. Please see **Figure 3** in the main text for conventions and **Text S2** and **Table S2** for the information about the PMs.

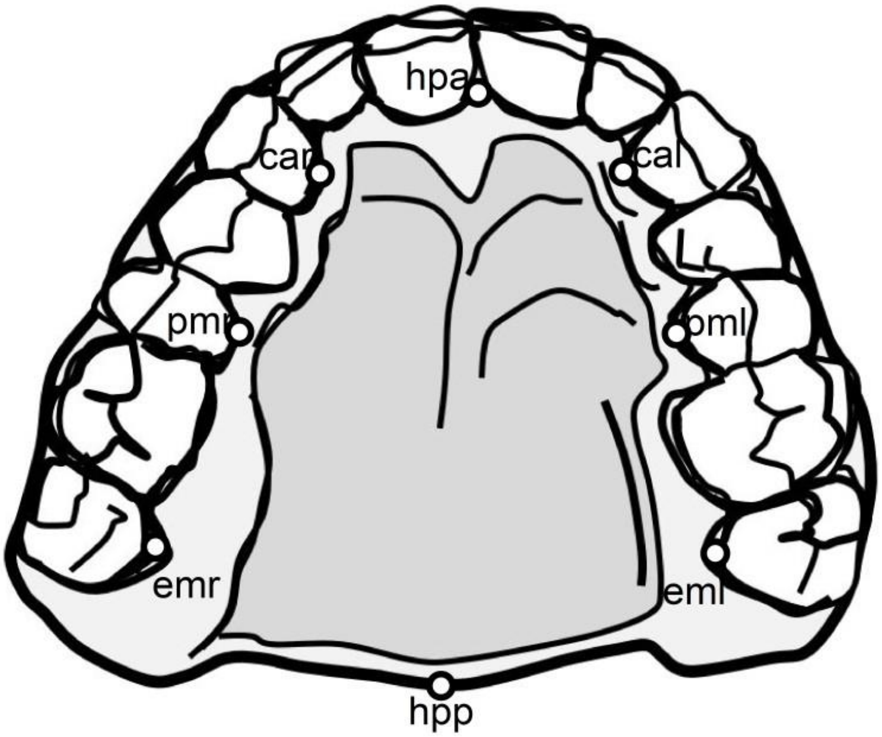

Fig. S14. Hard palate view of h^2 *class III* PMs.

Fig. S14. Hard palate view of h^2 *class III* PMs (i.e., PMs with relatively high narrow-sense heritability, but lacking inter-rater agreement). This is not shown in **Figure 3** in the main text. Please see **Figure 3** in the main text for conventions and **Text S2** and **Table S2** for the information about the PMs.

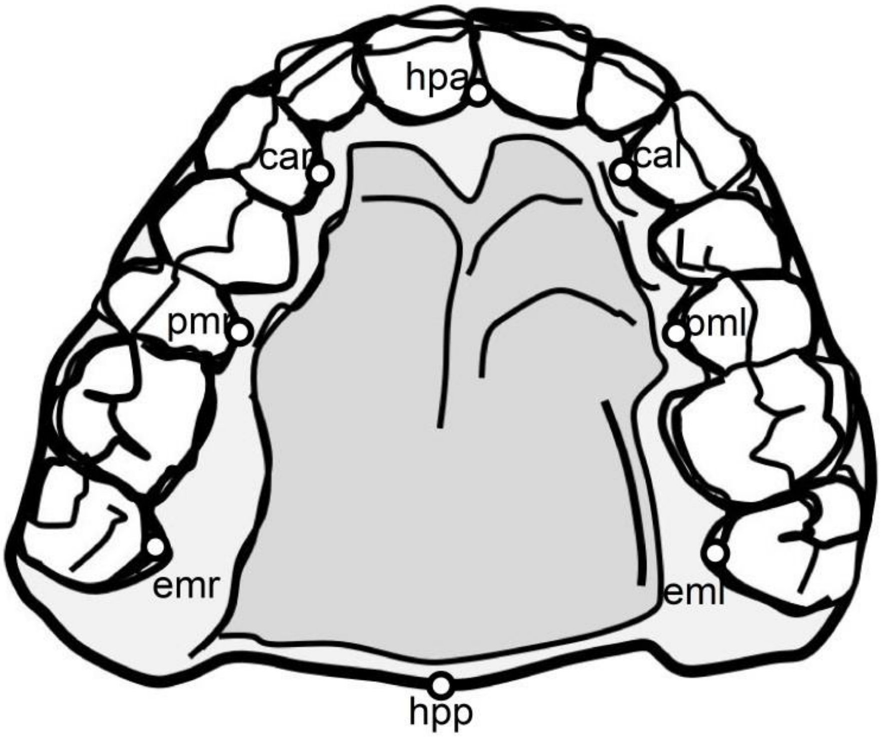

Fig. S15. Hard palate view of h^2 *class IV* PMs.

Fig. S15. Hard palate view of h^2 *class IV* PMs (i.e., PMs with circumstantial evidence of narrow-sense heritability, irrespective of inter-rater agreement). Please note that *ANSF* is not shown. This is **Panel H** of **Figure 3** in the main text. Please see **Figure 3** in the main text for conventions and **Text S2** and **Table S2** for the information about the PMs.

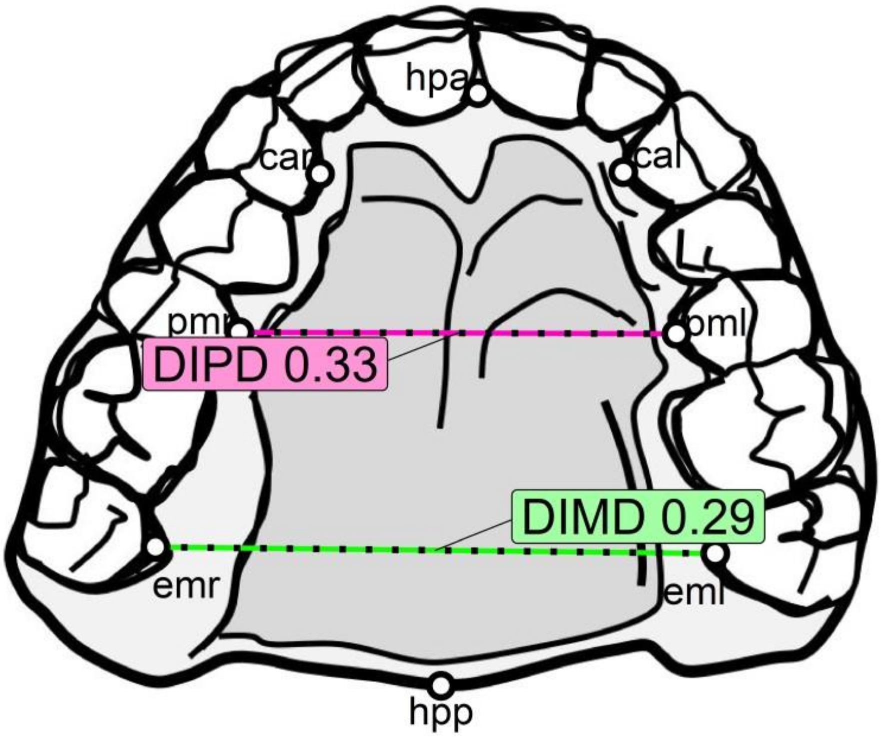

Supplementary tables

Table S1. The list of landmarks and semilandmarks.

Table S1. The list of landmarks and semilandmarks used to derive the phenotypic measures (PMs) described in **Table S2**. Shows the full name (and its abbreviation), if it is a landmark or a set of semilandmarks forming a curve, its full description, as well as various notes, comments and clarifications.

Name (abbr)	Semi	Description	Notes
head top (*ht*)	No	A point on the top of the head situated above the basion and in the midsagittal plane.	
head back (*hb*)	No	A point on the back of the head in the midsagittal plane directly posterior to the highest point of the cerebellum.	
nose tip (*nt*)	No	A point on the most anterior part of the nose.	
head side left (*hsl*)	No	A location on the left side of the head, deep within the pinna which marks the highest point of the external auditory meatus.	This point is comparable to the porion.
head side right (*hsr*)	No	A location on the right side of the head, deep within the pinna which marks the highest point of the external auditory meatus.	This point is comparable to the porion.
basion (*b*)	No	Most inferior posterior point in the midsagittal plane on the anterior rim of the foramen magnum.	Immediately superior and anterior to the odontoid and posterior and superior to the atlas.
odontoid (*o*)	No	Tip of the odontoid process of C2 in the midsagittal plane.	This landmark is very close to the basion.
atlas (*a*)	No	Most anterior point in the midsagittal plane of the anterior arch of the atlas (C1).	It is a point on the tip of the anterior tubercle.
C2 base (*c2b*)	No	Most anterior and inferior point of the body of C2 (axis) in the midsagittal plane.	
C3 base (*c3b*)	No	Most anterior and inferior point of the body of C3 in the midsagittal plane.	
C4 base (*c4b*)	No	Most anterior and inferior point of the body of C4 in the midsagittal plane.	This might not be visible for all participants.
C5 base (*c5b*)	No	Most anterior and inferior point of the body of C5 in the midsagittal plane.	This might not be visible for all participants.
sella (*s*)	No	The center of the sella turcica, bony seat of the pituitary gland.	The point should be positioned in the midsagittal plane and located directly below the pituitary infundibulum (stalk).

Name (abbr)	Semi	Description	Notes
nasion (*n*)	No	Anterior limit of the naso-frontal suture in the midsagittal plane. The bony part of where the top of the bridge of the nose connects with the bony part of the brow.	
nasal spine posterior (*nsp*)	No	Posterior most point of the hard palate in the midsagittal plane at the junction of the soft palate and nasal septum (vomer bone).	
nasal spine anterior (*nsa*)	No	Apex of the anterior projection of the maxilla above the incisive bone.	
hard palate posterior (*hpp*)	No	A point on the midsagittal palatal contour defined by posterior aspects of M2 in the coronal plane.	This landmark should be close to the visible junction between the soft and hard palates, and it should be approximately directly below the vomer-palatine junction. Defines the posterior landmark for the hard palate midsagittal semilandmarks.
hard palate anterior (*hpa*)	No	Defines the anterior landmark for the hard palate midsagittal semilandmarks.	This point is located at the lingual gingival margin of the interdental space between the central incisors.
uvula (*u*)	No	The apex of the uvula.	May be easiest to locate in the axial plane.
prosthion (*pr*)	No	A location above the central incisors on the alveolar bone of the maxilla located in the midsagittal plane.	Also known as the alveolar point.
pogonion (*pg*)	No	A point on the most prominent part of the mandibular symphysis in the midsagittal plane.	This landmark is always on the anterior aspect of the bone in comparison to the gnathion, which is found on the inferior aspect.
menton (*m*)	No	A point on the lower border of the mandibular symphysis in the midsagittal plane.	This landmark is always on the inferior aspect of the bone in comparison to the pogonion, which is found on the anterior aspect.
apex inferior (*ai*)	No	Root apex of the right-sided mandibular central incisor.	Right side only. Can be found by starting in the midsagittal plane and then searching laterally to the right for the sagittal plane which contains the best image of this incisor.
apex superior (*as*)	No	Root apex of the right-sided maxillary central incisor.	Right side only. Can be found by starting in the midsagittal plane and then searching laterally to the right for the sagittal plane which contains the best image of this incisor.
incision inferior (*ii*)	No	A point on the incisive edge of the right-sided mandibular central incisor.	Right side only. Can be found by starting in the midsagittal plane and then searching laterally to the right for the sagittal plane which contains the best image of this incisor.
incision superior (*is*)	No	A point on the incisive edge of the right-sided maxillary central incisor.	Right side only. Can be found by starting in the midsagittal plane and then searching laterally to the right for the sagittal plane which contains the best image of this incisor.

Name (abbr)	Semi	Description	Notes
gonion left (*gol*)	No	A point on the most lateral, posterior, and inferior part of the angle of the left side of the mandible.	This can be found by moving through the sagittal planes and observing the mandible. Place the landmark on the point of greatest curvature if possible.
gonion right (*gor*)	No	A point on the most lateral, posterior, and inferior part of the angle of the right side of the mandible.	This can be found by moving through the sagittal planes and observing the mandible. Place the landmark on the point of greatest curvature if possible.
condylion left (*cdl*)	No	A point on the most lateral surface of the left mandibular condyle.	Locate it by first searching in the sagittal view along the mandible at a part where the teeth are visible and then scanning laterally until the condyle can be identified with certainty. Keep searching laterally until the condyle disappears from view and mark its location.
condylion right (*cdr*)	No	A point on the most lateral surface of the right mandibular condyle.	Locate it by first searching in the sagittal view along the mandible at a part where the teeth are visible and then scanning laterally until the condyle can be identified with certainty. Keep searching laterally until the condyle disappears from view and mark its location.
canine left (*cal*)	No	A centrally located point on the gingival margin of the lingual surface of the left-sided maxillary canine (C).	Defines the start landmark of the hard palate coronal canine semilandmarks.
canine right (*car*)	No	A centrally located point on the gingival margin of the lingual surface of the right-sided maxillary canine (C).	Defines the end landmark of the hard palate coronal canine semilandmarks.
second premolar left (*pml*)	No	A centrally located point on the gingival margin of the lingual surface of the left-sided maxillary second pre-molar (PM2).	Defines the start landmark of the hard palate coronal second pre-molar semilandmarks.
second premolar right (*pmr*)	No	A centrally located point on the gingival margin of the lingual surface of the right-sided maxillary second pre-molar (PM2).	Defines the end landmark of the hard palate coronal second pre-molar semilandmarks.
endomolare left (*eml*)	No	A centrally located point on the gingival margin of the lingual surface of the left second molar.	Defines the start landmark for the hard palate coronal M2 semilandmarks and the start landmark of the maxillary dental arch semilandmarks.
endomolare right (*emr*)	No	A centrally located point on the gingival margin of the lingual surface of the right second molar.	Defines the end landmark for the hard palate coronal M2 semilandmarks and the end landmark of the maxillary dental arch semilandmarks.
corniculate tubercle (*ct*)	No	A point on the posterior and superior-most surface of the corniculate tubercles in the midsagittal plane.	May not be visible. Defines the end landmark of the pharynx wall semilandmarks.
nasopharynx (*np*)	No	A point on the posterior pharyngeal wall within the nasopharynx that intersects a line drawn between the atlas and nasal spine posterior landmarks.	Defines the start landmark of the pharynx wall semilandmarks.

Name (abbr)	Semi	Description	Notes
epiglottis apex (*epa*)	No	A point on the apex of the epiglottis in the midsagittal plane.	
epiglottis petiole (*epp*)	No	A point on the petiole of the epiglottis in the midsagittal plane.	May not always be visible.
orbitale left (*orl*)	No	The deepest point on the infraorbital margin.	May not be visible if the anonymized area over the eyes is too large. Used to define the Frankfort Horizontal.
orbitale right (*orr*)	No	The deepest point on the infraorbital margin.	May not be visible if the anonymized area over the eyes is too large. Used to define the Frankfort Horizontal.
hard palate midsagittal (*hpms*)	Yes	A curve formed from semilandmarks defined between hard palate posterior and hard palate anterior. Comprises 20 sample points. The individual semilandmark points are constrained to move only in the y-direction (axial freedom). The points are concentrated in the region of the alveolar ridge.	Care must be taken to locate the thin mucosal covering of the hard palate which defines the contour that is traced with the semilandmarks. It can be helpful to look in parallel slices to see how the mucosa looks in relation to the tongue, which often is pressed against the hard palate.
hard palate coronal 2nd molar (*hpc-m2*)	Yes	A curve formed from semilandmarks defined between endomolare left and endomolare right. Comprises 12 sample points. The individual semilandmark points are constrained to move only in the y-direction (axial freedom).	Care must be taken to locate the thin mucosal covering of the hard palate which defines the contour that is traced with the semilandmarks. It can be helpful to look in parallel slices to see how the mucosa looks in relation to the tongue, which often is pressed against the hard palate.
hard palate coronal 2nd premolar (*hpc-pm2*)	Yes	A curve formed from semilandmarks defined between second premolar left and second premolar right. Comprises 15 sample points. The individual semilandmark points are constrained to move only in the y-direction (axial freedom).	Care must be taken to locate the thin mucosal covering of the hard palate which defines the contour that is traced with the semilandmarks. It can be helpful to look in parallel slices to see how the mucosa looks in relation to the tongue, which often is pressed against the hard palate.
hard palate coronal canine (*hpc-c*)	Yes	A curve formed from semilandmarks defined between endomolare left and endomolare right. Comprises 15 sample points. The individual semilandmark points are constrained to move only in the y-direction (axial freedom).	Care must be taken to locate the thin mucosal covering of the hard palate which defines the contour that is traced with the semilandmark. It can be helpful to look in parallel slices to see how the mucosa looks in relation to the tongue, which often is pressed against the hard palate.
maxillary dental arch (*mda*)	Yes	A curve formed from semilandmarks defined between endomolare left and endomolare right. Comprises 15 sample points. The individual semilandmark points are constrained to move only in the y-direction (coronal freedom).	Care must be taken to locate the thin mucosal covering of the hard palate which defines the contour that is traced with the semilandmark. It can be helpful to look in parallel slices to see how the mucosa looks in relation to the tongue, which often is pressed against the hard palate.
pharynx wall (*pw*)	Yes	A curve formed from semilandmarks defined between corniculate tubercle and nasopharynx. Comprises 20	

Name (abbr)	Semi	Description	Notes
		sample points. The individual semilandmark points are constrained to move only in the x-direction (coronal freedom).	

Table S2. The list of all phenotypic measures (PMs).

Table S2. The list of all phenotypic measures (PMs) used here, with their short four-character names (the star * helps deduplicate otherwise identical short names, such as *SVTv* and *SVTv**), their anatomical domain and type, their full description (which makes reference to the landmarks and semilandmarks defined in **Table S1**), and their full names. Please note that 4 PMs turned out to be perfectly duplicated (i.e., they measured exactly the same things twice) and were subsequently removed from the analysis: column *Remove* shows "Yes" for the 4 exact duplicated PMs that were not considered (and shows, in parentheses, the corresponding PM that was kept). Column *Warning* shows the warning score for those measures with a score ≥ 3, as well as the main reasons for the score (these derive from manual and automatic checks and may seem rendudant as in, for example, "seems skewed" and "moderately skewed", the first derived subjectively from looking at the PM's histogram and QQ-plot, the second being based on the actual skewness of the distribution against [-1,1] for high skewness, and against [-1,-0.5] ∪ [0.5,1] for moderate skewness). Scores ≥ 5 represent serious interpretation warnings and were. in fact, dropped from the final results. The table is ordered by domain, type and short name. The short names of the PMs considered in the analysis are in ***bold italic***.

Name	Domain	Type	Description	Full name	Remove	Warning score
AANP	skull	angle	Angle between the line from nasion to the anterior nasal spine and the line from nasion to prosthion	SKULLANGLE_AANP_NASIONNSA_NASIONPROSTHION_ANGLE		3 (Seems skewed, moderately skewed)
AASN	skull	angle	Angle between the line from nasion to the anterior nasal spine and the line from nasion to sella	SKULLANGLE_AASN_NASIONNSA_NASIONSELLA_ANGLE		
ABSN	skull	angle	Angle between the line from sella to basion and the line from sella to nasion	SKULLANGLE_ABSN_SELLABASION_SELLANASION_ANGLE		
ACSN	skull	angle	Angle between the line from sella to the mean location of the condyles and the line from sella to the nasion	SKULLANGLE_ACSN_SELLACONYLIONMEAN_SELLANASION_ANGLE		
ALNS	skull	angle	Angle between line from nasion to sella and the nasal cavity floor	SKULLANGLE_ALNS_NASIONSELLALINE_NSANSPLINE_ANGLE		
ANNF	skull	angle	Angle between the floor of the nasal cavity and the Frankfort horizontal plane	SKULLANGLE_ANNF_NSANSP_SELLAFRANFORTPLANEPERP_ANGLE		
ANSF	skull	angle	Angle between the line from nasion to sella and the Frankfort horizontal plane	SKULLANGLE_ANSF_SELLANASION_SELLAFRANFORTPLANEPERP_ANGLE		
APNP	skull	angle	Angle between the line from nasion to pogonion and the line from nasion to prosthion	SKULLANGLE_APNP_NASIONPOGONION_NASIONPROSTHION_ANGLE		
APNS	skull	angle	Angle between the line from nasion to pogonion and the line from nasion to sella	SKULLANGLE_APNS_NASIONPOGONION_NASIONSELLA_ANGLE		

Name	Domain	Type	Description	Full name	Remove	Warning score
APSN	skull	angle	Angle between the line from sella to the posterior nasal spine and the line from sella to nasion	SKULLANGLE_APSN_SELLANSP_SELLANASION_ANGLE		
ASCG	skull	angle	Angle between the line from the mean gonion location to the mean condyle location and the line from the mean gonion location to menton	SKULLANGLE_ASCG_GONIONMEANCONDYLIONMEAN_GONIONMEANMENTON_A	Yes (*MCGM*)	
*ASCG**	skull	angle	Angle between the line from the mean condyle location to sella and the line from the mean condyle location to the mean gonion location	SKULLANGLE_ASCG_CONDMEANSELLA_CONDMEANGONIONMEAN_ANGLE		
ASNP	skull	angle	Angle between the line from nasion to sella and the line from nasion to prosthion	SKULLANGLE_ASNP_NASIONSELLA_NASIONPROSTHION_ANGLE		
CS2A	cervical	distance	Height of C2 from its base to odontoid tip	CERVICALSPINE_CS2A_C2BASE_ODONTOID_DISTANCE		
CS2H	cervical	distance	Height of C2 from its base to atlas (C1)	CERVICALSPINE_CS2H_ATLAS_C2BASE_DISTANCE		
CS3H	cervical	distance	Height of C3 from its base to C2 base	CERVICALSPINE_CS3H_C2BASE_C3BASE_DISTANCE		
CS4H	cervical	distance	Height of C4 from its base to C3 base	CERVICALSPINE_CS4H_C3BASE_C4BASE_DISTANCE		
CS5H	cervical	distance	Height of C5 from its base to C4 base	CERVICALSPINE_CS5H_C4BASE_C5BASE_DISTANCE		
CS6H	cervical	distance	Height of C6 from its base to C5 base	CERVICALSPINE_CS6H_C5BASE_C6BASE_DISTANCE		
CS7H	cervical	distance	Height of C7 from its base to C6 base	CERVICALSPINE_CS7H_C6BASE_C7BASE_DISTANCE		
DAII	dentition	angle	Angle between the nasal cavity floor and inferior right central incisor	DENITION_DAII_INCISIONINFAPEXINFLINE_NSANSPLINE_ANGLE		
DAIM	dentition	angle	Angle between the mandible body line (defined using the menton and mean gonion locations) and inferior right central incisor	DENITION_DAIM_INCISIONINFAPEXINFLINE_MENTONGONIONMEANLINE_		
DAIP	dentition	angle	Angle between the mandible body line (defined using the pogonion and mean gonion locations) and inferior right central incisor	DENITION_DAIP_INCINFAPEXINFLINE_POGGONMEANLINE_ANGLE		

Name	Domain	Type	Description	Full name	Remove	Warning score
DAIS	dentition	angle	Angle between the nasal cavity floor and superior right central incisor	DENITION_DAIS_INCISIONSUPAPEXSUPLINE_NSANSPLINE_ANGLE		
DDAP	dentition	Procrustes dist.	Procrustes distance of maxillary dental arch tracing	DENTITION_DDAP_DENTALARCHTRACE_MATLABPROCDISTANCE		5 (Seems skewed, moderately skewed, leptokurtic)
DICD	dentition	distance	Width of dental arch between the canines	DENITION_DICD_INTERCANINE_DISTANCE		
DIIL	dentition	distance	Distance between apex inferior and incisor inferior	DENITION_DIIL_APEXINFERIOR_INCISIONINFERIOR_DISTANCE		
DIMD	dentition	distance	Width of dental arch between the second molars	DENITION_DIMD_INTERM2_DISTANCE		
DIPD	dentition	distance	Width of dental arch between the second premolars	DENITION_DIPD_INTERPM2_DISTANCE		
DMAC	dentition	curvature	Curvature of the maxillary dental arch	DENITION_DMAC_MAXILLARYDENTALARCH_COMPLETETRACE_MEANCURVA		
DSIL	dentition	distance	Distance between apex superior and incisor superior	DENITION_DSIL_APEXSUPERIOR_INCISIONSUPERIOR_DISTANCE		
GAPD	general	Procrustes dist.	Procrustes distance of all available landmarks registered against the mean configuration defined by scans which contain all landmarks	GENERAL_GAPD_ALLPOINTSET_MATLABPROCDISTANCE		8 (Seems skewed, highly skewed, leptokurtic)
GRPD	general	Procrustes dist.	Procrustes distance of common points (excludes points not found in all scans)	GENERAL_GRPD_REGISTRATIONPOINTSET_MATLABPROCDISTANCE		8 (Seems skewed, highly skewed, leptokurtic)
HACL	hard palate	distance	Length of hard palate from canines to its anterior most point	HARDPALATE_HACL_HPA_INTERCANINE_DISTANCE		
HACP	hard palate	angle	Angle between the line from the intermolar projection (on midsagittal hard palate trace) to the anterior hard palate and the occlusal plane (containing pr, cal, car, pml, pmr, eml, emr)	HARDPALATE_HACP_CANPM2PROJ_OCCLUSALPLANE_POSTALVSLOPE_ANGLE		

Name	Domain	Type	Description	Full name	Remove	Warning score
HAML	hard palate	distance	Length of hard palate from second molars to its anterior most point	HARDPALATE_HAML_HPA_INTERM2_DISTANCE		
HAPL	hard palate	distance	Length of hard palate from second premolars to its anterior most point	HARDPALATE_HAPL_HPA_INTERPM2_DISTANCE		
HARC	hard palate	angle	Angle between the line from the intercanine projection to the anterior hard palate and the occlusal plane (containing pr, cal, car, pml, pmr, eml, emr)	HARDPALATE_HARC_HPAINTERCANINEPROJ_OCCLUSALPLANE_ALVRIDGE_		
HARF	hard palate	angle	Angle between the line from the interpremolar projection (on midsagittal hard palate trace) to the anterior hard palate and the occlusal plane (containing pr, cal, car, pml, pmr, eml, emr)	HARDPALATE_HARF_FRONTSEGSLOPE_OCCLUSALPLANE_ALVRIDGE_ANGLE		8 (Seems skewed, highly skewed)
HBC2	hyoid	distance	Distance between C2 body and hyoidale	HYOIDBONE_HBC2_C2BASE_HYOIDALE_DISTANCE		3 (moderately skewed)
HBC3	hyoid	distance	Distance between C3 body and hyoidale	HYOIDBONE_HBC3_C3BASE_HYOIDALE_DISTANCE		
HBC4	hyoid	distance	Distance between C4 body and hyoidale	HYOIDBONE_HBC4_C4BASE_HYOIDALE_DISTANCE		
HBNP	hyoid	distance	Distance between posterior nasal spine and hyoidale	HYOIDBONE_HBNP_NSP_HYOIDALE_DISTANCE		
HBPG	hyoid	distance	Distance between pogonion (chin) and hyoidale	HYOIDBONE_HBPG_POGONION_HYOIDALE_DISTANCE		
HBPL	hard palate	distance	Length (anteroposterior distance) of hard palate (lower face)	HARDPALATE_HBPL_BASION_PROSTHION_DISTANCE		
HCCC	hard palate	curvature	Curvature of coronal hard palate profile at the canines	HARDPALATE_HCCC_CORONALCANINE_COMPLETETRACE_MEANCURVATURE		
HCCP	hard palate	Procrustes dist.	Procrustes distance of canine coronal hard palate tracing	HARDPALATE_HCCP_CORONALCANINETRACE_MATLABPROCDISTANCE		8 (Latent rDZ correlation much larger than 1.0, moderately skewed)

Name	Domain	Type	Description	Full name	Remove	Warning score
HCMP	hard palate	Procrustes dist.	Procrustes distance of second molar coronal hard palate tracing	HARDPALATE_HCMP_CORONALM2TRACE_MATLABPROCDISTANCE		8 (Seems skewed, highly skewed, leptokurtic)
HCPP	hard palate	Procrustes dist.	Procrustes distance of second premolar coronal hard palate tracing	HARDPALATE_HCPP_CORONALPM2TRACE_MATLABPROCDISTANCE		5 (Seems skewed, leptokurtic)
HCTH	hard palate	distance	Height of coronal hard palate profile at the canines	HARDPALATE_HCTH_CORONALCANINETRACE_HEIGHT		
HDMP	hard palate	angle	Angle between line from the intermolar projection to the interpremolar projection (both of these on the midsagittal hard palate trace) and the occlusal plane (containing pr, cal, car, pml, pmr, eml, emr)	HARDPALATE_HDMP_M2PM2PROJ_OCCLUSALPLANE_PALATEDOME_ANGLE		8 (Sugests high kurtosis, highly skewed, leptokurtic)
HHPL	hard palate	distance	Length (anteroposterior distance) of hard palate	HARDPALATE_HHPL_HPA_HPP_DISTANCE		
HICH	hard palate	distance	Height of hard palate at midpoint between the canines	HARDPALATE_HICH_INTERCANINE_HEIGHT		
HICL	hard palate	distance	Length of hard palate from canines to incisors	HARDPALATE_HICL_INTERCANINE_INCISIONSUPERIOR_DISTANCE		
*HICL**	hard palate	distance	Length of hard palate from canines to prosthion	HARDPALATE_HICL_INTERCANINE_PROSTHION_DISTANCE		
HIMH	hard palate	distance	Height of hard palate at midpoint between the second molars	HARDPALATE_HIMH_INTERM2_HEIGHT		
HIML	hard palate	distance	Length of hard palate from second molars to incisors	HARDPALATE_HIML_INTERM2_INCISIONSUPERIOR_DISTANCE		
*HIML**	hard palate	distance	Length of hard palate from second molars to prosthion	HARDPALATE_HIML_INTERM2_PROSTHION_DISTANCE		
HIPH	hard palate	distance	Height of hard palate at midpoint between the second premolars	HARDPALATE_HIPH_INTERPM2_HEIGHT		
HIPL	hard palate	distance	Length of hard palate from second premolars to incisors	HARDPALATE_HIPL_INTERPM2_INCISIONSUPERIOR_DISTANCE		
*HIPL**	hard palate	distance	Length of hard palate from second premolars to prosthion	HARDPALATE_HIPL_INTERPM2_PROSTHION_DISTANCE		

Name	Domain	Type	Description	Full name	Remove	Warning score
HM2C	hard palate	curvature	Curvature of coronal hard palate profile at the second molars	HARDPALATE_HM2C_CORONALM2_COMPLETETRACE_MEANCURVATURE		
HMAC	hard palate	curvature	Curvature of alveolar ridge of midsagittal hard palate tracing (indices 16 to 20)	HARDPALATE_HMAC_MIDSAGITTAL_ALVRIDGEINDS15TO19_MEANCURVATURE		3 (Seems skewed, moderately skewed)
HMDC	hard palate	curvature	Curvature of dome of midsagittal hard palate tracing (indices 1 to 15)	HARDPALATE_HMDC_MIDSAGITTAL_DOMEINDICES1TO15_MEANCURVATURE		8 (Latent rDZ correlation much larger than 1.0)
HMSP	hard palate	Procrustes dist.	Procrustes distance of midsagittal hard palate tracing	HARDPALATE_HMSP_MIDSAGITTALTRACE_MATLABPROCDISTANCE		8 (Seems skewed, highly skewed, leptokurtic)
HMTC	hard palate	distance	Curvature of total midsagittal hard palate tracing	HARDPALATE_HMTC_MIDSAGITTAL_COMPLETETRACE_MEANCURVATURE		5 (Seems bimodal, moderately skewed, leptokurtic)
HMTH	hard palate	distance	Height of coronal hard palate profile at the second molars	HARDPALATE_HMTH_CORONALM2TRACE_HEIGHT		
HNAP	hard palate	distance	Height of hard palate on its external anterior surface	HARDPALATE_HNAP_NSA_PROSTHION_DISTANCE		
HNSL	hard palate	distance	Length (anteroposterior distance) of nasal cavity floor	HARDPALATE_HNSL_NSA_NSP_DISTANCE		
HP2C	hard palate	curvature	Curvature of coronal hard palate profile at the second premolars	HARDPALATE_HP2C_CORONALPM2_COMPLETETRACE_MEANCURVATURE		
HPTH	hard palate	distance	Height of coronal hard palate profile at the second premolars	HARDPALATE_HPTH_CORONALPM2TRACE_HEIGHT		
L2CT	larynx	distance	Height of larynx (using corniculate tubercle) relative to C2	LARYNX_L2CT_C2BASE_CORNICULATETUBERCLE_DISTANCE		
L2EA	larynx	distance	Height of larynx (using apex of epiglottis) relative to C2	LARYNX_L2EA_C2BASE_EPIGLOTTISAPEX_DISTANCE		3 (Seems skewed,

Name	Domain	Type	Description	Full name	Remove	Warning score
						moderately skewed)
L2EP	larynx	distance	Height of larynx (using petiole of epiglottis) relative to C2	LARYNX_L2EP_C2BASE_EPIGLOTTISPETIOLE_DISTANCE		
L3CT	larynx	distance	Height of larynx (using corniculate tubercle) relative to C3	LARYNX_L3CT_C3BASE_CORNICULATETUBERCLE_DISTANCE		
L3EA	larynx	distance	Height of larynx (using apex of epiglottis) relative to C3	LARYNX_L3EA_C3BASE_EPIGLOTTISAPEX_DISTANCE		3 (moderately skewed)
L3EP	larynx	distance	Height of larynx (using petiole of epiglottis) relative to C3	LARYNX_L3EP_C3BASE_EPIGLOTTISPETIOLE_DISTANCE		
L4CT	larynx	distance	Height of larynx (using corniculate tubercle) relative to C4	LARYNX_L4CT_C4BASE_CORNICULATETUBERCLE_DISTANCE		
L4EA	larynx	distance	Height of larynx (using apex of epiglottis) relative to C4	LARYNX_L4EA_C4BASE_EPIGLOTTISAPEX_DISTANCE		
L4EP	larynx	distance	Height of larynx (using petiole of epiglottis) relative to C4	LARYNX_L4EP_C4BASE_EPIGLOTTISPETIOLE_DISTANCE		
L5CT	larynx	distance	Height of larynx (using corniculate tubercle) relative to C5	LARYNX_L5CT_C5BASE_CORNICULATETUBERCLE_DISTANCE		
L5EA	larynx	distance	Height of larynx (using apex of epiglottis) relative to C5	LARYNX_L5EA_C5BASE_EPIGLOTTISAPEX_DISTANCE		
L5EP	larynx	distance	Height of larynx (using petiole of epiglottis) relative to C5	LARYNX_L5EP_C5BASE_EPIGLOTTISPETIOLE_DISTANCE		
LACT	larynx	distance	Height of larynx (using corniculate tubercle) relative to C1	LARYNX_LACT_ATLAS_CORNICULATETUBERCLE_DISTANCE		
LAEA	larynx	distance	Height of larynx (using apex of epiglottis) relative to C1	LARYNX_LAEA_ATLAS_EPIGLOTTISAPEX_DISTANCE		
LAEP	larynx	distance	Height of larynx (using petiole of epiglottis) relative to C1	LARYNX_LAEP_ATLAS_EPIGLOTTISPETIOLE_DISTANCE		
LEPL	larynx	distance	Height of epiglottis from petiole to apex	LARYNX_LEPL_EPIGLOTTISAPEX_EPIGLOTTISPETIOLE_DISTANCE		
LHCT	larynx	distance	Height of larynx (using corniculate tubercle) relative to hyoidale	LARYNX_LHCT_HYOIDALE_CORNICULATETUBERCLE_DISTANCE		
LHEA	larynx	distance	Height of larynx (using apex of epiglottis) relative to hyoidale	LARYNX_LHEA_HYOIDALE_EPIGLOTTISAPEX_DISTANCE		

Name	Domain	Type	Description	Full name	Remove	Warning score
LHEP	larynx	distance	Height of larynx (using petiole of epiglottis) relative to hyoidale	LARYNX_LHEP_HYOIDALE_EPIGLOTTISPETIOLE_DISTANCE		
LNCT	larynx	distance	Height of larynx (using corniculate tubercle) relative to posterior nasal spine	LARYNX_LNCT_NSP_CORNICULATETUBERCLE_DISTANCE	Yes (*SVTv**)	
LNEA	larynx	distance	Height of larynx (using apex of epiglottis) relative to posterior nasal spine	LARYNX_LNEA_NSP_EPIGLOTTISAPEX_DISTANCE		
LNEP	larynx	distance	Height of larynx (using petiole of epiglottis) relative to posterior nasal spine	LARYNX_LNEP_NSP_EPIGLOTTISPETIOLE_DISTANCE	Yes (*SVTv*)	
MCGD	mandible	distance	Height of ramus of mandible using mean location of condyles and angles (gonion)	MANDIBLE_MCGD_CONDYLIONMEAN_GONIONMEAN_DISTANCE		
MCGL	mandible	distance	Height of left ramus of mandible	MANDIBLE_MCGL_CONDYLIONLEFT_GONIONLEFT_DISTANCE		
MCGM	mandible	angle	Angle of the mandible using the menton and the mean locations of the condyles and angles (gonion)	MANDIBLE_MCGM_GONIONMEANCONYLIONMEAN_GONIONMEANMENTON_ANGLE		
MCGP	mandible	angle	Angle of the mandible using the pogonion and the mean locations of the condyles and angles (gonion)	MANDIBLE_MCGP_GONIONMEANCONYLIONMEAN_GONIONMEANPOGONION_A		
MCGR	mandible	distance	Height of right ramus of mandible	MANDIBLE_MCGR_CONDYLIONRIGHT_GONIONRIGHT_DISTANCE		
MCMD	mandible	distance	Length of body of mandible using menton and mean location of condyles	MANDIBLE_MCMD_CONDYLIONMEAN_MENTON_DISTANCE		
MCPD	mandible	distance	Length of body of mandible using pogonion and mean location of condyles	MANDIBLE_MCPD_CONDYLIONMEAN_POGONION_DISTANCE		
MGMD	mandible	distance	Length of body of mandible using menton and mean location of angles (gonion)	MANDIBLE_MGMD_GONIONMEAN_MENTON_DISTANCE		
MGPD	mandible	distance	Length of body of mandible using pogonion and mean location of angles (gonion)	MANDIBLE_MGPD_GONIONMEAN_POGONION_DISTANCE		
MICD	mandible	distance	Width of mandible between the condyles	MANDIBLE_MICD_INTERCONDYLION_DISTANCE		
MIGD	mandible	distance	Width of mandible between the angles (gonion)	MANDIBLE_MIGD_INTERGONIAL_DISTANCE		
MMPG	mandible	distance	Height of chin	MANDIBLE_MMPG_MENTON_POGONION_DISTANCE		

Name	Domain	Type	Description	Full name	Remove	Warning score
MPGL	mandible	distance	Length of left side of body of mandible	MANDIBLE_MPGL_POGONION_GONIONLEFT_DISTANCE		
MPGR	mandible	distance	Length of right side of body of mandible	MANDIBLE_MPGR_POGONION_GONIONRIGHT_DISTANCE		
OCHL	oral	ratio	Ratio between intercanine height of hard palate and intercanine distance to the anterior-most point of the hard palate (i.e., a hard palate height:length ratio)	ORALCAVITY_OCHL_INTERCANHEIGHT_INTERCANHPADIST_HLRATIO		
OCHW	oral	ratio	Ratio between intercanine height of hard palate and intercanine width (i.e., a hard palate height:width ratio)	ORALCAVITY_OCHW_INTERCANHEIGHT_INTERCANDIST_HWRATIO		
OCLW	oral	ratio	Ratio between intercanine distance to the anterior-most point of the hard palate and intercanine width (i.e., a hard palate length:width ratio)	ORALCAVITY_OCLW_INTERCANDIST_INTERCANHPADIST_WLRATIO		3 (Seems skewed, moderately skewed)
OCMR	oral	ratio	Ratio of intercanine width to intermolar width	ORALCAVITY_OCMR_INTERCANDIST_INTERM2DIST_RATIO		
OCPR	oral	ratio	Ratio of intercanine width to interpremolar width	ORALCAVITY_OCPR_INTERCANDIST_INTERPM2DIST_RATIO		
OMHL	oral	ratio	Ratio between intermolar height of hard palate and intermolar distance to the anterior-most point of the hard palate (i.e., a hard palate height:length ratio)	ORALCAVITY_OMHL_INTERM2HEIGHT_INTERM2HPADIST_HLRATIO		
OMHW	oral	ratio	Ratio between intermolar height of hard palate and intermolar width (i.e., a hard palate height:width ratio)	ORALCAVITY_OMHW_INTERM2HEIGHT_INTERM2DIST_HWRATIO		
OMLW	oral	ratio	Ratio between intermolar distance to the anterior-most point of the hard palate and intermolar width of hard palate (i.e., a hard palate length:width ratio)	ORALCAVITY_OMLW_INTERM2DIST_INTERM2HPADIST_WLRATIO		
OPHL	oral	ratio	Ratio between interpremolar height of hard palate and interpremolar distance to the anterior-most point of the hard palate (i.e., a hard palate height:length ratio)	ORALCAVITY_OPHL_INTERPM2HEIGHT_INTERPM2HPADIST_HLRATIO		

Name	Domain	Type	Description	Full name	Remove	Warning score
OPHW	oral	ratio	Ratio between interpremolar height of hard palate and interpremolar width (i.e., a hard palate height:width ratio)	ORALCAVITY_OPHW_INTERPM2HEIGHT_INTERPM2DIST_HWRATIO		
OPLW	oral	ratio	Ratio between interpremolar distance to the anterior-most point of the hard palate and interpremolar width (i.e., a hard palate length:width ratio)	ORALCAVITY_OPLW_INTERPM2DIST_INTERPM2HPADIST_WLRATIO		
OPMR	oral	ratio	Ratio of interpremolar width to intermolar width	ORALCAVITY_OPMR_INTERPM2DIST_INTERM2DIST_RATIO		
PNNP	pharynx	distance	Length (anteroposterior distance) of nasopharynx	PHARYNX_PNNP_NSP_NASOPHARYNX_DISTANCE		
PPW2	pharynx	curvature	Curvature of pharynx wall down to projection of C2	PHARYNX_PPW2_PHARYNXWALL_TOC2PROJECTION_MEANCURVATURE		8 (Sugests high kurtosis, leptokurtic)
PPW3	pharynx	curvature	Curvature of pharynx wall down to projection of C3	PHARYNX_PPW3_PHARYNXWALL_TOC3PROJECTION_MEANCURVATURE		8 (Sugests high kurtosis, moderately skewed, leptokurtic)
PPW4	pharynx	curvature	Curvature of pharynx wall down to projection of C4	PHARYNX_PPW4_PHARYNXWALL_TOC4PROJECTION_MEANCURVATURE		6 (Sugests high kurtosis, leptokurtic)
PPW5	pharynx	curvature	Curvature of pharynx wall down to projection of C5	PHARYNX_PPW5_PHARYNXWALL_TOC5PROJECTION_MEANCURVATURE		3 (Sugests high kurtosis)
PPW6	pharynx	curvature	Curvature of pharynx wall down to projection of C6	PHARYNX_PPW6_PHARYNXWALL_TOC6PROJECTION_MEANCURVATURE		
PPW7	pharynx	curvature	Curvature of pharynx wall down to projection of C7	PHARYNX_PPW7_PHARYNXWALL_TOC7PROJECTION_MEANCURVATURE		
PPWC	pharynx	curvature	Curvature of pharynx wall complete trace	PHARYNX_PPWC_PHARYNXWALL_COMPLETETRACE_MEANCURVATURE		8 (Sugests high kurtosis, moderately

Name	Domain	Type	Description	Full name	Remove	Warning score
						skewed, leptokurtic)
PWTP	pharynx	Procrustes dist.	Procrustes distance of pharyngeal wall tracing	PHARYNX_PWTP_MIDSAGITTAL TRACE_MATLABPROCDISTANCE		
SBAN	skull	distance	Length of anterior basicranium (using nasion)	SKULL_SBAN_BASION_NASION_ DISTANCE		
SBAP	skull	distance	Length of facial skeleton (using prosthion)	SKULL_SBAP_BASION_PROSTHIO N_DISTANCE	Yes (*HBPL*)	
SBAS	skull	distance	Length of middle basicranium (using sella)	SKULL_SBAS_BASION_SELLA_DI STANCE		
SBNA	skull	distance	Length of facial skeleton (using anterior nasal spine)	SKULL_SBNA_BASION_NSA_DIST ANCE		
SBNP	skull	distance	Distance between basion and posterior nasal spine (using posterior nasal spine)	SKULL_SBNP_BASION_NSP_DIST ANCE		
SHBN	skull	distance	Length of head	SKULL_SHBN_HEADBACK_NASI ON_DISTANCE		
SHSW	skull	distance	Width of head	SKULL_SHSW_HEADSIDELEFT_H EADSIDERIGHT_DISTANCE		
SIOD	skull	distance	Distance between the eyes (infraorbital distance)	SKULL_SIOD_INTERORBITALE_D ISTANCE		
SNAM	skull	distance	Height of facial skeleton (using menton)	SKULL_SNAM_NASION_MENTON _DISTANCE		
SNAP	skull	distance	Height of facial skeleton (using pogonion)	SKULL_SNAP_NASION_POGONIO N_DISTANCE		
SNNA	skull	distance	Height of facial skeleton (using anterior nasal spine)	SKULL_SNNA_NASION_NSA_DIS TANCE		
SNNP	skull	distance	Length of anterior basicranium (using nasion and posterior nasal spine)	SKULL_SNNP_NASION_NSP_DIST ANCE		
SNOL	skull	distance	Distance from left infraorbital pit to bridge of nose	SKULL_SNOL_NASION_ORBITAL ELEFT_DISTANCE		
SNOR	skull	distance	Distance from right infraorbital pit to bridge of nose	SKULL_SNOR_NASION_ORBITAL ERIGHT_DISTANCE		
SPUN	soft palate	distance	Length of soft palate from posterior nasal spine to uvula tip	SOFTPALATE_SPUN_UVULA_NSP _DISTANCE		

Name	Domain	Type	Description	Full name	Remove	Warning score
SSEG	skull	distance	Height of basicranium relative to mean condyle location	SKULL_SSEG_SELLA_CONDYLIONMEAN_DISTANCE		
SSEN	skull	distance	Length of anterior basicranium (between sella and nasion)	SKULL_SSEN_SELLA_NASION_DISTANCE		
*SSEN**	skull	distance	Height of posterior nasal cavity	SKULL_SSEN_SELLA_NSP_DISTANCE		
SVTh	general	distance	Length of the horizontal supralaryngeal vocal tract (using the anterior-most point of the hard palate and the atlas)	GENERAL_SVTH_ATLAS_HPA_DISTANCE		
*SVTh**	general	distance	Length of the horizontal supralaryngeal vocal tract (using the prosthion and the atlas)	GENERAL_SVTH_ATLAS_PROSTHION_DISTANCE		
SVTv	general	distance	Length of the vertical supralaryngeal vocal tract (using the petiole of the epiglottis and and the posterior nasal spine)	GENERAL_SVTV_NSP_EPIGLOTTISPETIOLE_DISTANCE		
*SVTv**	general	distance	Length of the vertical supralaryngeal vocal tract (using the corniculate tubercle and the posterior nasal spine)	GENERAL_SVTV_NSP_CORNICULATETUBERCLE_DISTANCE		

Table S3. Pairs of very highly correlated PMs.

Table S3. Pairs of very highly correlated (Pearson's r ≥ 0.90) PMs ordered by their correlation. PM1 and PM2 are the names of the measures; *r* is Pearson's correlation estimate; Descriptions give the actual descriptions of the two measures (in the order PM1, PM2) separated by a semicolon (;). See **Text S2** and **Table S2** for the information about the PMs.

PM1	PM2	*r*	Descriptions
HICH	*OCHW*	0.98	*HICH*: Height of hard palate at midpoint between the canines; *OCHW*: Ratio between intercanine height of hard palate and intercanine width (i.e., a hard palate height:width ratio)
DAIM	*DAIP*	0.98	*DAIM*: Angle between the mandible body line (defined using the menton and mean gonion locations) and inferior right central incisor; *DAIP*: Angle between the mandible body line (defined using the pogonion and mean gonion locations) and inferior right central incisor
SNAM	*SNAP*	0.97	*SNAM*: Height of facial skeleton (using menton); *SNAP*: Height of facial skeleton (using pogonion)
MCGM	*MCGP*	0.96	*MCGM*: Angle of the mandible using the menton and the mean locations of the condyles and angles (gonion); *MCGP*: Angle of the mandible using the pogonion and the mean locations of the condyles and angles (gonion)
MCMD	*MCPD*	0.96	*MCMD*: Length of body of mandible using menton and mean location of condyles; *MCPD*: Length of body of mandible using pogonion and mean location of condyles
L2CT	*L3CT*	0.96	*L2CT*: Height of larynx (using corniculate tubercle) relative to C2; *L3CT*: Height of larynx (using corniculate tubercle) relative to C3
MGMD	*MGPD*	0.95	*MGMD*: Length of body of mandible using menton and mean location of angles (gonion); *MGPD*: Length of body of mandible using pogonion and mean location of angles (gonion)
L2EP	*L3EP*	0.95	*L2EP*: Height of larynx (using petiole of epiglottis) relative to C2; *L3EP*: Height of larynx (using petiole of epiglottis) relative to C3
MGPD	*MPGR*	0.94	*MGPD*: Length of body of mandible using pogonion and mean location of angles (gonion); *MPGR*: Length of right side of body of mandible
L4EA	*L5EA*	0.93	*L4EA*: Height of larynx (using apex of epiglottis) relative to C4; *L5EA*: Height of larynx (using apex of epiglottis) relative to C5
MCGD	*MCGL*	0.93	*MCGD*: Height of ramus of mandible using mean location of condyles and angles (gonion); *MCGL*: Height of left ramus of mandible
MGPD	*MPGL*	0.92	*MGPD*: Length of body of mandible using pogonion and mean location of angles (gonion); *MPGL*: Length of left side of body of mandible
L2CT	*LACT*	0.92	*L2CT*: Height of larynx (using corniculate tubercle) relative to C2; *LACT*: Height of larynx (using corniculate tubercle) relative to C1
HBPL	*SVTh**	0.92	*HBPL*: Length (anteroposterior distance) of hard palate (lower face); *SVTh**: Length of the horizontal supralaryngeal vocal tract (using the prosthion and the atlas)
HIMH	*OMHW*	0.92	*HIMH*: Height of hard palate at midpoint between the second molars; *OMHW*: Ratio between intermolar height of hard palate and intermolar width (i.e., a hard palate height:width ratio)

**PM1**	**PM2**	***r***	**Descriptions**
L2EP	*LAEP*	0.91	*L2EP*: Height of larynx (using petiole of epiglottis) relative to C2; *LAEP*: Height of larynx (using petiole of epiglottis) relative to C1
MCGD	*MCGR*	0.91	*MCGD*: Height of ramus of mandible using mean location of condyles and angles (gonion); *MCGR*: Height of right ramus of mandible
HAML	*HAPL*	0.90	*HAML*: Length of hard palate from second molars to its anterior most point; *HAPL*: Length of hard palate from second premolars to its anterior most point

Table S4. The number of PMs supporting the 2r vs the 1r models.

Table S4. The number of PMs supporting the 2r (i.e., different error variances between raters) vs the 1r (i.e., equal error variances) models. We used three ways of quantifying, for a given PM, which of the two models is better supported (or if they are deemed equivalent): looking at the direction and the difference in AIC points (ΔAIC) and comparing it against 2 (a rather weak difference between models) or against 10 (a very strong difference), and looking for a significant (at α-level 0.05) likelihood ratio test. For each of these methods (the rows) we give the number (and %) of PMs that support the 2r, neither, or the 1r model (the columns). *N.B.*, both 2r and 1r assume equal latent means between raters.

**Method**	**2r supported**	**2r and 1r equivalent**	**1r supported**
$\lvert\Delta AIC\rvert > 10$	0 (0.0%)	144 (98.6%)	2 (1.4%)
$\lvert\Delta AIC\rvert > 2$	0 (0.0%)	136 (93.2%)	10 (6.8%)
LRT at α-level 0.05	0 (0.0%)	136 (93.2%)	10 (6.8%)

Table S5. The full results.

Table S5. The full results. For each PM, we give the narrow sense heritability h^2, the shared environment c^2 or dominance d^2 components, and the non-shared environment and error e^2; for each component we give: the point estimate (the 95% confidence interval in parentheses), the model comparison likelihood ratio test nominal *p*-value (and the Holm-corrected *p*-value in parentheses). We also give the inter-rater agreement *ICC*(*C*,1) as a point estimate (and with 95% confidence interval). For h^2 and c^2/d^2, we give the class to which the PM belongs in terms of the strength of evidence in favour of narrow sense heritability, shared environment or dominance (ordered decreasingly from the most convincing in *class I* to virtually no evidence in *class V*). The genetic model fitted (ACE or ADE) helps interpret the 'c^2 or d^2' column. The table is ordered by decreasing h^2 class and estimate. See **Text S2** and **Table S2** for the information about the PMs.

Name	Model	h^2	Class	c^2 or d^2	Class	e^2	*ICC*(*C*,1)
SSEN	ACE	0.47 (0.19, 0.87), p=9.46×10^{-5} (0.013)	I	0.40 (0.00, 0.67), p=0.049 (1)	II	0.13 (0.09, 0.17), p=1.18×10^{-24} (1.34×10^{-22})	0.90 (0.89, 0.92)
SHBN	ACE	0.56 (0.31, 0.91), p=1.99×10^{-9} (2.88×10^{-7})	I	0.34 (0.00, 0.58), p=0.068 (1)	IV	0.10 (0.08, 0.13), p=4.26×10^{-44} (6.04×10^{-42})	0.96 (0.95, 0.96)
SNNP	ACE	0.62 (0.28, 0.95), p=2.2×10^{-5} (0.003)	I	0.29 (0.00, 0.63), p=0.219 (1)	IV	0.08 (0.04, 0.13), p=7.28×10^{-5} (0.003)	0.82 (0.80, 0.85)
SHSW	ACE	0.63 (0.28, 0.86), p=3.15×10^{-5} (0.004)	I	0.19 (0.00, 0.53), p=0.396 (1)	V	0.18 (0.14, 0.24), p=2.37×10^{-37} (3.22×10^{-35})	0.94 (0.93, 0.95)
SBAN	ACE	0.66 (0.36, 0.92), p=1.69×10^{-7} (2.41×10^{-5})	I	0.22 (0.00, 0.52), p=0.256 (1)	IV	0.11 (0.08, 0.16), p=2.04×10^{-15} (1.79×10^{-13})	0.89 (0.88, 0.91)
SBNA	ACE	0.67 (0.32, 0.87), p=5.76×10^{-6} (7.94×10^{-4})	I	0.17 (0.00, 0.51), p=0.443 (1)	V	0.17 (0.12, 0.22), p=8.04×10^{-30} (1×10^{-27})	0.90 (0.88, 0.91)
*ASCG**	ACE	0.67 (0.41, 0.85), p=1.74×10^{-5} (0.002)	I	0.14 (0.00, 0.48), p=0.536 (1)	V	0.20 (0.15, 0.26), p=1.91×10^{-41} (2.68×10^{-39})	0.87 (0.85, 0.89)
MCGM	ACE	0.67 (0.43, 0.85), p=1.74×10^{-5} (0.002)	I	0.14 (0.00, 0.48), p=0.536 (1)	V	0.20 (0.15, 0.26), p=1.91×10^{-41} (2.68×10^{-39})	0.89 (0.87, 0.90)
MIGD	ACE	0.69 (0.39, 0.91), p=8.41×10^{-9} (1.21×10^{-6})	I	0.19 (0.00, 0.49), p=0.33 (1)	V	0.12 (0.09, 0.17), p=1.39×10^{-28} (1.72×10^{-26})	0.95 (0.94, 0.95)
MCGP	ACE	0.74 (0.38, 0.86), p=3.07×10^{-6} (4.29×10^{-4})	I	0.07 (0.00, 0.44), p=0.747 (1)	V	0.18 (0.14, 0.24), p=1.13×10^{-30} (1.44×10^{-28})	0.87 (0.84, 0.88)
SSEG	ACE	0.77 (0.38, 0.83), p=5.21×10^{-6} (7.24×10^{-4})	I	0.01 (0.00, 0.39), p=0.969 (1)	V	0.22 (0.17, 0.29), p=3×10^{-48} (4.29×10^{-46})	0.90 (0.88, 0.91)
SNAM	ACE	0.78 (0.40, 0.88), p=7.92×10^{-7} (1.12×10^{-4})	I	0.06 (0.00, 0.43), p=0.812 (1)	V	0.17 (0.12, 0.22), p=2.02×10^{-28} (2.45×10^{-26})	0.90 (0.89, 0.92)
SNAP	ACE	0.79 (0.40, 0.88), p=9.46×10^{-7} (1.33×10^{-4})	I	0.05 (0.00, 0.43), p=0.819 (1)	V	0.16 (0.12, 0.22), p=2.17×10^{-20} (2.34×10^{-18})	0.88 (0.86, 0.90)

Name	Model	h^2	Class	c^2 or d^2	Class	e^2	$ICC(C,1)$
MICD	ACE	0.87 (0.51, 0.90), $p=2.58\times10^{-13}$ (3.76×10^{-11})	I	0.01 (0.00, 0.36), p=0.977 (1)	V	0.13 (0.10, 0.16), $p=7.58\times10^{-105}$ (1.11×10^{-102})	0.98 (0.98, 0.99)
*SVTh**	ACE	0.26 (0.03, 0.62), p=0.028 (1)	II	0.55 (0.20, 0.77), p=0.005 (0.774)	II	0.19 (0.15, 0.24), $p=3.59\times10^{-57}$ (5.21×10^{-55})	0.93 (0.91, 0.94)
HBPL	ACE	0.38 (0.12, 0.78), p=0.002 (0.267)	II	0.45 (0.08, 0.71), p=0.027 (1)	II	0.16 (0.12, 0.21), $p=4.12\times10^{-35}$ (5.4×10^{-33})	0.91 (0.89, 0.92)
HBC4	ACE	0.52 (0.04, 0.72), p=0.032 (1)	II	0.12 (0.00, 0.57), p=0.659 (1)	V	0.36 (0.28, 0.46), $p=2.33\times10^{-38}$ (3.19×10^{-36})	0.79 (0.76, 0.82)
HBNP	ACE	0.57 (0.11, 0.78), p=0.013 (1)	II	0.13 (0.00, 0.56), p=0.601 (1)	V	0.30 (0.22, 0.40), $p=3.3\times10^{-20}$ (3.53×10^{-18})	0.84 (0.81, 0.86)
APNS	ACE	0.73 (0.25, 0.81), $p=7.16\times10^{-4}$ (0.093)	II	0.02 (0.00, 0.48), p=0.952 (1)	V	0.25 (0.19, 0.33), $p=2.28\times10^{-31}$ (2.92×10^{-29})	0.83 (0.80, 0.85)
MCGR	ACE	0.59 (0.05, 0.82), p=0.032 (1)	III	0.12 (0.00, 0.61), p=0.667 (1)	V	0.29 (0.18, 0.42), $p=2.07\times10^{-8}$ (1.22×10^{-6})	0.68 (0.64, 0.72)
AASN	ACE	0.66 (0.17, 0.92), p=0.007 (0.896)	III	0.18 (0.00, 0.65), p=0.553 (1)	V	0.17 (0.08, 0.26), $p=6.16\times10^{-5}$ (0.002)	0.63 (0.58, 0.67)
SNOL	ACE	0.69 (0.09, 1.00), p=0.023 (1)	III	0.31 (0.00, 0.90), p=0.43 (1)	IV	0.00 (0.00, 0.11), p=1 (1)	0.54 (0.48, 0.59)
ASNP	ACE	0.73 (0.26, 0.91), $p=6.67\times10^{-4}$ (0.088)	III	0.12 (0.00, 0.58), p=0.694 (1)	V	0.16 (0.09, 0.23), $p=4\times10^{-8}$ (2.28×10^{-6})	0.72 (0.68, 0.76)
MCGD	ACE	0.80 (0.31, 0.91), $p=7.37\times10^{-4}$ (0.095)	III	0.02 (0.00, 0.47), p=0.943 (1)	V	0.18 (0.09, 0.29), $p=4.32\times10^{-5}$ (0.002)	0.71 (0.67, 0.75)
CS2A	ACE	0.88 (0.36, 1.00), $p=7.13\times10^{-4}$ (0.093)	III	0.12 (0.00, 0.66), p=0.75 (1)	V	0.00 (0.00, 0.08), p=1 (1)	0.60 (0.55, 0.65)
*SVTv**	ACE	0.24 (0.00, 0.74), p=0.307 (1)	IV	0.43 (0.00, 0.73), p=0.118 (1)	IV	0.32 (0.23, 0.43), $p=1.95\times10^{-18}$ (1.95×10^{-16})	0.80 (0.77, 0.83)
MPGR	ADE	0.24 (0.00, 0.88), p=0.668 (1)	IV	0.60 (0.00, 0.89), p=0.251 (1)	IV	0.16 (0.11, 0.23), $p=2.06\times10^{-13}$ (1.69×10^{-11})	0.84 (0.81, 0.86)
MCPD	ADE	0.26 (0.00, 0.86), p=0.615 (1)	IV	0.57 (0.00, 0.87), p=0.219 (1)	IV	0.18 (0.13, 0.23), $p=7.04\times10^{-33}$ (9.08×10^{-31})	0.92 (0.90, 0.93)
LAEA	ACE	0.27 (0.00, 0.61), p=0.394 (1)	IV	0.23 (0.00, 0.58), p=0.509 (1)	IV	0.49 (0.38, 0.62), $p=3.45\times10^{-35}$ (4.56×10^{-33})	0.77 (0.73, 0.80)
DIMD	ACE	0.29 (0.00, 0.75), p=0.197 (1)	IV	0.40 (0.00, 0.72), p=0.133 (1)	IV	0.32 (0.23, 0.42), $p=1.09\times10^{-22}$ (1.23×10^{-20})	0.77 (0.73, 0.80)

**Name**	**Model**	$\boldsymbol{h^2}$	**Class**	c^2 or d^2	**Class**	e^2	*ICC*(*C*,1)
ACSN	ADE	0.30 (0.00, 0.84), *p*=0.574 (1)	IV	0.50 (0.00, 0.85), *p*=0.31 (1)	IV	0.20 (0.15, 0.26), $p=1.07\times10^{-35}$ (1.43×10^{-33})	0.87 (0.85, 0.89)
HNSL	ACE	0.31 (0.00, 0.78), *p*=0.085 (1)	IV	0.49 (0.02, 0.80), *p*=0.043 (1)	II	0.20 (0.14, 0.28), $p=7.74\times10^{-13}$ (6.27×10^{-11})	0.77 (0.74, 0.80)
DIPD	ADE	0.33 (0.00, 0.72), *p*=0.568 (1)	IV	0.32 (0.00, 0.73), *p*=0.56 (1)	IV	0.35 (0.27, 0.46), $p=9.52\times10^{-36}$ (1.28×10^{-33})	0.80 (0.77, 0.82)
HBC2	ACE	0.35 (0.00, 0.73), *p*=0.196 (1)	IV	0.29 (0.00, 0.67), *p*=0.325 (1)	IV	0.37 (0.26, 0.49), $p=2.41\times10^{-17}$ (2.34×10^{-15})	0.78 (0.74, 0.81)
ABSN	ADE	0.36 (0.00, 0.84), *p*=0.495 (1)	IV	0.42 (0.00, 0.84), *p*=0.386 (1)	IV	0.21 (0.16, 0.29), $p=8.5\times10^{-26}$ (9.95×10^{-24})	0.82 (0.79, 0.84)
HBPG	ACE	0.37 (0.00, 0.68), *p*=0.147 (1)	IV	0.22 (0.00, 0.62), *p*=0.45 (1)	IV	0.40 (0.31, 0.51), $p=5.8\times10^{-44}$ (8.18×10^{-42})	0.81 (0.78, 0.83)
L4EA	ACE	0.43 (0.00, 0.69), *p*=0.099 (1)	IV	0.17 (0.00, 0.61), *p*=0.55 (1)	V	0.40 (0.31, 0.51), $p=3.83\times10^{-34}$ (4.98×10^{-32})	0.76 (0.72, 0.79)
*SSEN**	ADE	0.46 (0.00, 0.83), *p*=0.393 (1)	IV	0.32 (0.00, 0.83), *p*=0.515 (1)	IV	0.22 (0.16, 0.28), $p=6.98\times10^{-37}$ (9.43×10^{-35})	0.90 (0.88, 0.91)
SBNP	ADE	0.65 (0.00, 0.86), *p*=0.257 (1)	IV	0.16 (0.00, 0.85), *p*=0.761 (1)	V	0.20 (0.14, 0.27), $p=1.89\times10^{-18}$ (1.91×10^{-16})	0.80 (0.77, 0.83)
ANSF	ADE	0.73 (0.00, 0.84), *p*=0.219 (1)	IV	0.05 (0.00, 0.83), *p*=0.928 (1)	V	0.22 (0.16, 0.30), $p=2.97\times10^{-19}$ (3.06×10^{-17})	0.79 (0.75, 0.81)
SBAS	ADE	0.78 (0.00, 0.86), *p*=0.112 (1)	IV	0.01 (0.00, 0.84), *p*=0.977 (1)	V	0.20 (0.14, 0.28), $p=2.39\times10^{-15}$ (2.08×10^{-13})	0.79 (0.76, 0.82)
CS4H	ACE	0.00 (0.00, 0.32), *p*=1 (1)	V	0.56 (0.40, 0.70), *p*=0.008 (1)	II	0.44 (0.30, 0.60), $p=2.3\times10^{-9}$ (1.41×10^{-7})	0.59 (0.53, 0.64)
DAIM	ADE	0.00 (0.00, 0.72), *p*=1 (1)	V	0.70 (0.58, 0.81), *p*=0.06 (1)	IV	0.30 (0.19, 0.42), $p=2.24\times10^{-10}$ (1.45×10^{-8})	0.63 (0.58, 0.67)
HACL	ACE	0.00 (0.00, 0.67), *p*=1 (1)	V	0.35 (0.00, 0.55), *p*=0.593 (1)	IV	0.65 (0.36, 0.93), $p=5.96\times10^{-4}$ (0.017)	0.26 (0.19, 0.33)
SVTv	ACE	0.00 (0.00, 0.36), *p*=1 (1)	V	0.62 (0.25, 0.72), *p*=0.007 (0.944)	II	0.38 (0.28, 0.50), $p=1.33\times10^{-15}$ (1.2×10^{-13})	0.73 (0.70, 0.77)
HPTH	ADE	0.00 (0.00, 0.85), *p*=1 (1)	V	0.76 (0.62, 0.76), *p*=0.271 (1)	IV	0.24 (0.14, 0.38), $p=1.78\times10^{-4}$ (0.006)	0.52 (0.46, 0.57)
MGMD	ADE	0.00 (0.00, 0.65), *p*=1 (1)	V	0.85 (0.18, 0.90), *p*=0.018 (1)	II	0.15 (0.10, 0.22), $p=1.7\times10^{-10}$ (1.15×10^{-8})	0.80 (0.77, 0.83)

Name	Model	h^2	Class	c^2 or d^2	Class	e^2	*ICC*(*C*,1)
DAIP	ADE	0.00 (0.00, 0.73), p=1 (1)	V	0.70 (0.00, 0.80), p=0.068 (1)	IV	0.30 (0.20, 0.42), p=1.89×10^{-10} (1.26×10^{-8})	0.62 (0.57, 0.67)
APNP	ADE	0.00 (0.00, 0.75), p=1 (1)	V	0.71 (0.00, 0.79), p=0.112 (1)	IV	0.29 (0.21, 0.37), p=1.66×10^{-25} (1.93×10^{-23})	0.76 (0.73, 0.79)
HHPL	ACE	0.00 (0.00, 0.58), p=1 (1)	V	0.51 (0.00, 0.66), p=0.07 (1)	IV	0.49 (0.34, 0.65), p=7.91×10^{-11} (5.54×10^{-9})	0.50 (0.44, 0.56)
L3CT	ACE	0.00 (0.00, 0.52), p=1 (1)	V	0.58 (0.06, 0.68), p=0.031 (1)	II	0.42 (0.32, 0.54), p=2×10^{-21} (2.22×10^{-19})	0.69 (0.64, 0.73)
LHEP	ADE	0.00 (0.00, 0.50), p=1 (1)	V	0.32 (0.00, 0.51), p=0.706 (1)	IV	0.68 (0.49, 0.87), p=5.46×10^{-12} (4.15×10^{-10})	0.47 (0.41, 0.53)
HCCC	ACE	0.00 (0.00, 0.79), p=1 (1)	V	0.57 (0.00, 0.85), p=0.216 (1)	IV	0.43 (0.15, 0.69), p=0.005 (0.11)	0.31 (0.24, 0.38)
HP2C	ADE	0.00 (0.00, 0.62), p=1 (1)	V	0.34 (0.00, 0.66), p=0.542 (1)	IV	0.66 (0.34, 0.97), p=2.96×10^{-4} (0.009)	0.26 (0.19, 0.34)
MMPG	ADE	0.00 (0.00, 1.00), p=1 (1)	V	0.84 (0.00, 1.00), p=0.226 (1)	IV	0.16 (0.00, 0.40), p=0.238 (1)	0.32 (0.24, 0.39)
LEPL	ADE	0.00 (0.00, 0.63), p=1 (1)	V	0.46 (0.00, 0.68), p=0.353 (1)	IV	0.54 (0.32, 0.78), p=7.07×10^{-6} (3.04×10^{-4})	0.40 (0.34, 0.47)
DAII	ADE	0.00 (0.00, 0.37), p=1 (1)	V	0.63 (0.19, 0.74), p=0.014 (1)	II	0.37 (0.26, 0.49), p=2.42×10^{-18} (2.4×10^{-16})	0.66 (0.61, 0.70)
OCHL	ACE	0.00 (0.00, 0.76), p=1 (1)	V	0.54 (0.00, 0.77), p=0.252 (1)	IV	0.46 (0.23, 0.69), p=4.69×10^{-4} (0.014)	0.35 (0.28, 0.42)
L4CT	ADE	0.00 (0.00, 0.49), p=1 (1)	V	0.36 (0.00, 0.55), p=0.29 (1)	IV	0.64 (0.45, 0.86), p=3.02×10^{-12} (2.33×10^{-10})	0.46 (0.39, 0.52)
HMAC	ADE	0.00 (0.00, 1.00), p=1 (1)	V	0.37 (0.00, 1.00), p=0.582 (1)	IV	0.63 (0.00, 1.00), p=0.223 (1)	0.08 (0.01, 0.16)
CS2H	ADE	0.00 (0.00, 0.94), p=1 (1)	V	0.88 (0.00, 0.94), p=0.157 (1)	IV	0.12 (0.05, 0.24), p=0.017 (0.262)	0.59 (0.53, 0.64)
AANP	ADE	0.00 (0.00, 0.63), p=1 (1)	V	0.47 (0.00, 0.67), p=0.349 (1)	IV	0.53 (0.33, 0.75), p=1×10^{-6} (4.92×10^{-5})	0.40 (0.33, 0.47)
HACP	ACE	0.00 (0.00, 0.42), p=1 (1)	V	0.25 (0.00, 0.43), p=0.479 (1)	IV	0.75 (0.57, 0.93), p=7.67×10^{-16} (6.98×10^{-14})	0.47 (0.41, 0.53)
PWTP	ACE	0.00 (0.00, 0.70), p=1 (1)	V	0.54 (0.00, 0.71), p=0.238 (1)	IV	0.46 (0.29, 0.64), p=1.42×10^{-6} (6.52×10^{-5})	0.48 (0.42, 0.54)

Name	Model	h^2	Class	c^2 or d^2	Class	e^2	*ICC*(*C*,1)
DMAC	ACE	0.00 (0.00, 0.85), p=1 (1)	V	0.43 (0.00, 0.82), p=0.587 (1)	IV	0.57 (0.17, 0.91), p=0.015 (0.237)	0.21 (0.13, 0.28)
PNNP	ADE	0.02 (0.00, 0.85), p=0.975 (1)	V	0.81 (0.00, 0.88), p=0.11 (1)	IV	0.18 (0.12, 0.25), $p=9.61\times10^{-12}$ (7.11×10^{-10})	0.74 (0.70, 0.77)
PPW5	ACE	0.02 (0.00, 0.74), p=0.967 (1)	V	0.51 (0.00, 0.73), p=0.286 (1)	IV	0.47 (0.24, 0.69), $p=1.34\times10^{-4}$ (0.005)	0.41 (0.35, 0.48)
L3EA	ADE	0.02 (0.00, 0.63), p=0.974 (1)	V	0.52 (0.00, 0.65), p=0.406 (1)	IV	0.45 (0.35, 0.58), $p=9.75\times10^{-40}$ (1.35×10^{-37})	0.76 (0.72, 0.79)
L2CT	ACE	0.03 (0.00, 0.61), p=0.908 (1)	V	0.60 (0.04, 0.72), p=0.038 (1)	II	0.37 (0.27, 0.48), $p=1.41\times10^{-20}$ (1.54×10^{-18})	0.73 (0.69, 0.76)
HIPL	ADE	0.03 (0.00, 0.68), p=0.964 (1)	V	0.53 (0.00, 0.69), p=0.468 (1)	IV	0.43 (0.31, 0.58), $p=2.55\times10^{-14}$ (2.17×10^{-12})	0.60 (0.55, 0.65)
HAML	ACE	0.04 (0.00, 0.75), p=0.906 (1)	V	0.62 (0.00, 0.78), p=0.085 (1)	IV	0.34 (0.20, 0.48), $p=4.38\times10^{-7}$ (2.3×10^{-5})	0.56 (0.50, 0.61)
MGPD	ADE	0.05 (0.00, 0.87), p=0.933 (1)	V	0.78 (0.00, 0.88), p=0.141 (1)	IV	0.17 (0.12, 0.23), $p=5.11\times10^{-16}$ (4.7×10^{-14})	0.83 (0.80, 0.85)
LAEP	ACE	0.07 (0.00, 0.65), p=0.807 (1)	V	0.50 (0.00, 0.67), p=0.106 (1)	IV	0.43 (0.32, 0.55), $p=1.68\times10^{-21}$ (1.88×10^{-19})	0.74 (0.70, 0.77)
OMLW	ACE	0.09 (0.00, 0.68), p=0.728 (1)	V	0.52 (0.00, 0.71), p=0.074 (1)	IV	0.38 (0.27, 0.51), $p=3.7\times10^{-16}$ (3.44×10^{-14})	0.70 (0.65, 0.73)
MCMD	ADE	0.09 (0.00, 0.86), p=0.852 (1)	V	0.75 (0.00, 0.88), p=0.105 (1)	IV	0.16 (0.12, 0.21), $p=2.44\times10^{-28}$ (2.93×10^{-26})	0.92 (0.90, 0.93)
HAPL	ACE	0.12 (0.00, 0.61), p=0.784 (1)	V	0.34 (0.00, 0.60), p=0.442 (1)	IV	0.54 (0.38, 0.70), $p=8.73\times10^{-13}$ (6.98×10^{-11})	0.52 (0.46, 0.57)
L5CT	ACE	0.13 (0.00, 0.65), p=0.607 (1)	V	0.44 (0.00, 0.65), p=0.108 (1)	IV	0.43 (0.32, 0.56), $p=3.27\times10^{-25}$ (3.76×10^{-23})	0.71 (0.67, 0.75)
HMTH	ADE	0.14 (0.00, 0.77), p=0.864 (1)	V	0.49 (0.00, 0.79), p=0.544 (1)	IV	0.37 (0.21, 0.56), $p=7.69\times10^{-6}$ (3.23×10^{-4})	0.46 (0.40, 0.52)
*HICL**	ACE	0.14 (0.00, 0.80), p=0.804 (1)	V	0.44 (0.00, 0.78), p=0.443 (1)	IV	0.42 (0.20, 0.64), $p=5.96\times10^{-4}$ (0.017)	0.39 (0.32, 0.45)
MPGL	ADE	0.15 (0.00, 0.83), p=0.798 (1)	V	0.62 (0.00, 0.84), p=0.261 (1)	IV	0.22 (0.16, 0.30), $p=3.36\times10^{-20}$ (3.56×10^{-18})	0.84 (0.82, 0.86)
OPLW	ACE	0.15 (0.00, 0.61), p=0.71 (1)	V	0.29 (0.00, 0.58), p=0.477 (1)	IV	0.56 (0.39, 0.74), $p=8.07\times10^{-12}$ (6.05×10^{-10})	0.50 (0.44, 0.56)

Name	Model	h^2	Class	c^2 or d^2	Class	e^2	*ICC*(*C*,1)
*HIPL**	ACE	0.15 (0.00, 0.68), *p*=0.635 (1)	V	0.41 (0.00, 0.67), *p*=0.241 (1)	IV	0.43 (0.31, 0.57), *p*=3.3×10^{-16} (3.1×10^{-14})	0.63 (0.58, 0.67)
HNAP	ACE	0.17 (0.00, 0.39), *p*=0.747 (1)	V	0.48 (0.00, 0.82), *p*=0.363 (1)	IV	0.36 (0.15, 0.57), *p*=0.001 (0.031)	0.37 (0.30, 0.44)
HICH	ACE	0.17 (0.00, 0.68), *p*=0.712 (1)	V	0.30 (0.00, 0.63), *p*=0.479 (1)	IV	0.53 (0.32, 0.75), *p*=1.36×10^{-6} (6.37×10^{-5})	0.39 (0.32, 0.46)
LACT	ACE	0.17 (0.00, 0.69), *p*=0.463 (1)	V	0.47 (0.00, 0.71), *p*=0.078 (1)	IV	0.36 (0.27, 0.46), *p*=5.12×10^{-27} (6.05×10^{-25})	0.81 (0.78, 0.83)
SVTh	ACE	0.17 (0.00, 0.53), *p*=0.227 (1)	V	0.59 (0.22, 0.79), *p*=0.005 (0.703)	II	0.24 (0.18, 0.30), *p*=3.54×10^{-50} (5.09×10^{-48})	0.90 (0.88, 0.91)
HCTH	ACE	0.19 (0.00, 0.73), *p*=0.713 (1)	V	0.34 (0.00, 0.70), *p*=0.507 (1)	IV	0.47 (0.27, 0.67), *p*=1.13×10^{-5} (4.65×10^{-4})	0.44 (0.37, 0.50)
OCHW	ACE	0.20 (0.00, 0.72), *p*=0.65 (1)	V	0.33 (0.00, 0.68), *p*=0.417 (1)	IV	0.47 (0.27, 0.68), *p*=5.06×10^{-6} (2.23×10^{-4})	0.41 (0.34, 0.47)
CS3H	ACE	0.20 (0.00, 0.89), *p*=0.536 (1)	V	0.60 (0.00, 0.90), *p*=0.117 (1)	IV	0.20 (0.07, 0.34), *p*=0.003 (0.074)	0.60 (0.54, 0.64)
OCLW	ACE	0.21 (0.00, 0.50), *p*=0.753 (1)	V	0.01 (0.00, 0.01), *p*=0.986 (1)	V	0.78 (0.50, 1.00), *p*=1.91×10^{-6} (8.61×10^{-5})	0.25 (0.17, 0.32)
OPHL	ACE	0.22 (0.00, 0.67), *p*=0.699 (1)	V	0.25 (0.00, 0.64), *p*=0.663 (1)	IV	0.53 (0.33, 0.74), *p*=8.95×10^{-7} (4.56×10^{-5})	0.38 (0.31, 0.44)
HM2C	ADE	0.22 (0.00, 0.91), *p*=0.88 (1)	V	0.30 (0.00, 0.88), *p*=0.839 (1)	IV	0.48 (0.07, 0.84), *p*=0.028 (0.387)	0.23 (0.15, 0.30)
LHEA	ACE	0.23 (0.00, 0.52), *p*=0.63 (1)	V	0.14 (0.00, 0.50), *p*=0.775 (1)	V	0.63 (0.48, 0.80), *p*=2.73×10^{-17} (2.62×10^{-15})	0.61 (0.56, 0.66)
*HIML**	ACE	0.24 (0.00, 0.78), *p*=0.375 (1)	V	0.47 (0.00, 0.77), *p*=0.125 (1)	IV	0.29 (0.19, 0.41), *p*=4.34×10^{-10} (2.78×10^{-8})	0.65 (0.60, 0.69)
DAIS	ACE	0.24 (0.00, 0.54), *p*=0.555 (1)	V	0.17 (0.00, 0.52), *p*=0.699 (1)	V	0.59 (0.46, 0.72), *p*=1.24×10^{-30} (1.56×10^{-28})	0.65 (0.60, 0.69)
LNEA	ADE	0.25 (0.00, 0.63), *p*=0.705 (1)	V	0.27 (0.00, 0.63), *p*=0.668 (1)	IV	0.48 (0.37, 0.62), *p*=1.62×10^{-28} (1.99×10^{-26})	0.75 (0.71, 0.78)
CS7H	ACE	0.25 (0.00, 0.74), *p*=0.372 (1)	V	0.40 (0.00, 0.72), *p*=0.229 (1)	IV	0.35 (0.25, 0.47), *p*=1.43×10^{-16} (1.35×10^{-14})	0.69 (0.64, 0.72)
ALNS	ACE	0.26 (0.00, 0.80), *p*=0.399 (1)	V	0.46 (0.00, 0.78), *p*=0.221 (1)	IV	0.29 (0.18, 0.41), *p*=2.06×10^{-9} (1.27×10^{-7})	0.60 (0.55, 0.65)

Name	Model	h^2	Class	c^2 or d^2	Class	e^2	*ICC*(*C*,1)
HARC	ACE	0.26 (0.00, 0.59), p=0.613 (1)	V	0.08 (0.00, 0.52), p=0.868 (1)	V	0.66 (0.41, 0.90), p=4.35×10^{-7} (2.3×10^{-5})	0.36 (0.29, 0.42)
HIPH	ACE	0.26 (0.00, 0.72), p=0.576 (1)	V	0.27 (0.00, 0.68), p=0.565 (1)	IV	0.46 (0.28, 0.65), p=1.03×10^{-6} (4.92×10^{-5})	0.43 (0.36, 0.49)
OCPR	ADE	0.27 (0.00, 0.56), p=0.766 (1)	V	0.14 (0.00, 0.57), p=0.874 (1)	V	0.59 (0.43, 0.76), p=1.35×10^{-15} (1.2×10^{-13})	0.52 (0.46, 0.57)
L2EP	ACE	0.28 (0.00, 0.71), p=0.359 (1)	V	0.33 (0.00, 0.68), p=0.326 (1)	IV	0.39 (0.28, 0.52), p=2.6×10^{-15} (2.23×10^{-13})	0.67 (0.62, 0.71)
L3EP	ACE	0.29 (0.00, 0.72), p=0.401 (1)	V	0.31 (0.00, 0.68), p=0.391 (1)	IV	0.41 (0.28, 0.56), p=3.66×10^{-11} (2.6×10^{-9})	0.57 (0.52, 0.62)
OCMR	ACE	0.29 (0.00, 0.70), p=0.492 (1)	V	0.25 (0.00, 0.65), p=0.55 (1)	IV	0.47 (0.30, 0.66), p=1.96×10^{-8} (1.17×10^{-6})	0.47 (0.40, 0.53)
PPW7	ACE	0.30 (0.00, 0.80), p=0.249 (1)	V	0.43 (0.00, 0.77), p=0.17 (1)	IV	0.28 (0.19, 0.39), p=1.4×10^{-11} (1.02×10^{-9})	0.71 (0.67, 0.74)
OMHL	ACE	0.30 (0.00, 0.60), p=0.531 (1)	V	0.11 (0.00, 0.55), p=0.805 (1)	V	0.59 (0.40, 0.79), p=1.84×10^{-9} (1.16×10^{-7})	0.44 (0.37, 0.50)
OMHW	ACE	0.30 (0.00, 0.63), p=0.501 (1)	V	0.17 (0.00, 0.59), p=0.704 (1)	V	0.53 (0.37, 0.70), p=1.75×10^{-11} (1.26×10^{-9})	0.48 (0.42, 0.54)
DSIL	ACE	0.30 (0.00, 0.82), p=0.47 (1)	V	0.35 (0.00, 0.78), p=0.419 (1)	IV	0.35 (0.18, 0.53), p=1.08×10^{-4} (0.004)	0.45 (0.39, 0.51)
DICD	ADE	0.31 (0.00, 0.74), p=0.663 (1)	V	0.34 (0.00, 0.75), p=0.612 (1)	IV	0.35 (0.25, 0.47), p=3.7×10^{-19} (3.77×10^{-17})	0.71 (0.67, 0.75)
SNNA	ACE	0.31 (0.00, 0.92), p=0.212 (1)	V	0.56 (0.00, 0.91), p=0.09 (1)	IV	0.13 (0.04, 0.24), p=0.005 (0.117)	0.66 (0.61, 0.70)
L2EA	ADE	0.40 (0.00, 0.63), p=0.631 (1)	V	0.11 (0.00, 0.63), p=0.889 (1)	V	0.49 (0.37, 0.63), p=4.14×10^{-20} (4.31×10^{-18})	0.65 (0.61, 0.70)
OPHW	ACE	0.42 (0.00, 0.66), p=0.31 (1)	V	0.09 (0.00, 0.60), p=0.833 (1)	V	0.49 (0.34, 0.67), p=1.6×10^{-10} (1.1×10^{-8})	0.50 (0.43, 0.55)
L5EA	ACE	0.43 (0.00, 0.70), p=0.114 (1)	V	0.18 (0.00, 0.63), p=0.551 (1)	V	0.39 (0.30, 0.51), p=1.63×10^{-28} (1.99×10^{-26})	0.74 (0.70, 0.77)
HIML	ACE	0.44 (0.00, 0.81), p=0.137 (1)	V	0.27 (0.00, 0.73), p=0.413 (1)	IV	0.29 (0.18, 0.43), p=2.81×10^{-8} (1.63×10^{-6})	0.63 (0.58, 0.67)
SPUN	ACE	0.45 (0.00, 0.77), p=0.135 (1)	V	0.21 (0.00, 0.69), p=0.52 (1)	IV	0.34 (0.23, 0.47), p=2.96×10^{-12} (2.31×10^{-10})	0.63 (0.58, 0.67)

Name	Model	h^2	Class	c^2 or d^2	Class	e^2	*ICC*(*C*,1)
OPMR	ADE	0.46 (0.00, 0.69), p=0.571 (1)	V	0.10 (0.00, 0.69), p=0.899 (1)	V	0.44 (0.31, 0.58), p=1.21×10^{-13} (1×10^{-11})	0.57 (0.52, 0.62)
L5EP	ADE	0.47 (0.00, 0.67), p=0.485 (1)	V	0.09 (0.00, 0.67), p=0.891 (1)	V	0.44 (0.32, 0.57), p=2.4×10^{-21} (2.64×10^{-19})	0.66 (0.61, 0.70)
HIMH	ADE	0.48 (0.00, 0.67), p=0.584 (1)	V	0.03 (0.00, 0.68), p=0.975 (1)	V	0.50 (0.32, 0.69), p=4.02×10^{-8} (2.28×10^{-6})	0.47 (0.41, 0.53)
MCGL	ACE	0.48 (0.00, 0.78), p=0.087 (1)	V	0.18 (0.00, 0.65), p=0.526 (1)	V	0.34 (0.22, 0.48), p=2.17×10^{-10} (1.43×10^{-8})	0.68 (0.64, 0.72)
L4EP	ADE	0.49 (0.00, 0.72), p=0.607 (1)	V	0.06 (0.00, 0.72), p=0.951 (1)	V	0.46 (0.28, 0.65), p=9.34×10^{-7} (4.67×10^{-5})	0.44 (0.38, 0.50)
HBC3	ACE	0.54 (0.00, 0.76), p=0.064 (1)	V	0.12 (0.00, 0.64), p=0.696 (1)	V	0.34 (0.24, 0.47), p=4.87×10^{-14} (4.09×10^{-12})	0.71 (0.67, 0.74)
LHCT	ACE	0.54 (0.00, 0.74), p=0.133 (1)	V	0.08 (0.00, 0.65), p=0.834 (1)	V	0.38 (0.26, 0.52), p=1.5×10^{-12} (1.19×10^{-10})	0.66 (0.62, 0.70)
APSN	ADE	0.54 (0.00, 0.91), p=0.376 (1)	V	0.32 (0.00, 0.91), p=0.566 (1)	IV	0.14 (0.09, 0.21), p=7.09×10^{-8} (3.9×10^{-6})	0.73 (0.69, 0.76)
PPW6	ACE	0.54 (0.00, 0.92), p=0.255 (1)	V	0.18 (0.00, 0.82), p=0.71 (1)	V	0.28 (0.08, 0.49), p=0.008 (0.162)	0.43 (0.37, 0.50)
CS5H	ACE	0.55 (0.00, 0.79), p=0.251 (1)	V	0.06 (0.00, 0.70), p=0.907 (1)	V	0.40 (0.22, 0.59), p=3.86×10^{-5} (0.002)	0.43 (0.37, 0.50)
DIIL	ADE	0.58 (0.00, 1.00), p=0.688 (1)	V	0.12 (0.00, 1.00), p=0.936 (1)	V	0.31 (0.00, 0.65), p=0.123 (1)	0.23 (0.15, 0.30)
SIOD	ADE	0.58 (0.00, 1.00), p=0.344 (1)	V	0.38 (0.00, 1.00), p=0.502 (1)	IV	0.04 (0.00, 0.11), p=0.315 (1)	0.71 (0.67, 0.74)
HICL	ADE	0.60 (0.00, 0.87), p=0.578 (1)	V	0.09 (0.00, 0.87), p=0.931 (1)	V	0.31 (0.12, 0.50), p=0.002 (0.041)	0.40 (0.33, 0.46)
CS6H	ADE	0.65 (0.00, 0.78), p=0.445 (1)	V	0.00 (0.00, 0.78), p=0.996 (1)	V	0.35 (0.21, 0.50), p=1.04×10^{-7} (5.63×10^{-6})	0.56 (0.50, 0.61)
ANNF	ADE	0.67 (0.00, 0.80), p=0.303 (1)	V	0.06 (0.00, 0.79), p=0.927 (1)	V	0.28 (0.20, 0.37), p=3.63×10^{-20} (3.81×10^{-18})	0.74 (0.71, 0.78)
SNOR	ADE	0.91 (0.00, 1.00), p=0.29 (1)	V	0.09 (0.00, 1.00), p=0.911 (1)	V	0.00 (0.00, 0.13), p=1 (1)	0.53 (0.47, 0.58)

Table S6. Relationship between the ΔAIC with cut-off 2 and the likelihood ratio test (LRT) at α-level 0.05.

Table S6. Relationship between the ΔAIC with cut-off 2 and the likelihood ratio test (LRT) at α-level 0.05. For each of variance components (rows), we give the number of PMs (also as %) for which the two criteria agree that the component is significantly different from 0 (column 'TT'), for which they disagree with ΔAIC judging it significantly different from 0 but LRT not (column 'TF'), for which they disagree with ΔAIC judging it not significantly different from 0 but LRT does (column 'FT'), and for which they agree in judging it not significantly different from 0 (column 'FF'). The only disagreement is for c^2 for PM *SSEN*, where ΔAIC = 1.86 and LRT's $p = 0.049$; given the limited nature of this single case, it can be concluded that the two criteria are virtually identical for our data.

Component	TT	TF	FT	FF
h^2	25 (17%)	0 (0%)	0 (0%)	121 (83%)
c^2	9 (10%)	0 (0%)	1 (1%)	83 (89%)
d^2	2 (4%)	0 (0%)	0 (0%)	51 (96%)
e^2	133 (91%)	0 (0%)	0 (0%)	13 (9%)

Table S7. Correlations between the estimates of interest.

Table S7. Correlations between the estimates of interest, including with a Bonferroni correction.

Var 1	Var 2	Pearson's *r*	*p* (adjusted *p*)
ICC(*C*,1)	h^2	0.42	7.07×10^{-7} (2.83×10^{-6})
ICC(*C*,1)	c^2	-0.16	0.152 (0.607)
ICC(*C*,1)	d^2	0.20	0.174 (0.694)
ICC(*C*,1)	e^2	-0.64	1.72×10^{-16} (6.87×10^{-16})

Table S8. Classes of strength of evidence for narrow-sense heritability h^2.

Table S8. Classes of strength of evidence for narrow-sense heritability h^2, with the number of PMs in each group. *Class I* gives the strongest evidence, while *class V* gives virtually no evidence. The criteria are defined in the main text, but briefly are: h^{2*c} = statistically significant h^2 after multiple testing correction; h^{2*} = nominally statistically significant h^2; $h^{2>>}$ = the 95%CI of h^2 is above 0.20; $h^{2>}$ = h^2 is above 0.20; ICC^{++} = the 95%CI of *ICC*(*C*,1) is above 0.75; ICC^{++} = *ICC*(*C*,1) is above 0.75.

Class	Count	h^{2*c}	h^{2*}	$h^{2>>}$	$h^{2>}$	ICC^{++}	ICC^{+}
I	13	Yes	Yes	Yes	Yes	Yes	Yes
I	1	Yes	Yes	No	Yes	Yes	Yes
II	1	No	Yes	Yes	Yes	Yes	Yes
III	3	No	Yes	Yes	Yes	No	No
II	4	No	Yes	No	Yes	Yes	Yes
III	3	No	Yes	No	Yes	No	No
IV	11	No	No	No	Yes	Yes	Yes
IV	5	No	No	No	Yes	No	Yes
V	43	No	No	No	Yes	No	No
V	6	No	No	No	No	Yes	Yes
V	2	No	No	No	No	No	Yes
V	39	No	No	No	No	No	No

Table S9. Classes of strength of evidence for c^2.

Table S9. Classes of strength of evidence for c^2, with the number of PMs in each group. *Class I* gives the strongest evidence (not available here), while *class V* gives virtually no evidence. The criteria are defined in the main text, but briefly are: c^{2*c} = statistically significant c^2 after multiple testing correction; c^{2*} = nominally statistically significant c^2; $c^{2>>}$ = the 95%CI of c^2 is above 0.20; $c^{2>}$ = c^2 is above 0.20.

Class	Count	c^{2*c}	c^{2*}	$c^{2>>}$	$c^{2>}$
II	3	No	Yes	Yes	Yes
II	6	No	Yes	No	Yes
IV	44	No	No	No	Yes
V	32	No	No	No	No

Table S10.Classes of strength of evidence for d^2.

Table S10. Classes of strength of evidence for d^2, with the number of PMs in each group. *Class I* gives the strongest evidence (not available here), while *class V* gives virtually no evidence. The criteria are defined in the main text, but briefly are: d^{2*c} = statistically significant d^2 after multiple testing correction; d^{2*} = nominally statistically significant d^2; $d^{2>>}$ = the 95%CI of d^2 is above 0.20; $d^{2>}$ = d^2 is above 0.20.

Class	Count	d^{2*c}	d^{2*}	$d^{2>>}$	$d^{2>}$
II	2	No	Yes	No	Yes
IV	2	No	No	Yes	Yes
IV	28	No	No	No	Yes
V	14	No	No	No	No

Table S11. The distribution of the PMs of h^2 class at least *IV*.

Table S11. The distribution of the 41 PMs of h^2 class at least *IV* across classes overall (2nd column), domains (next 8 columns) and types (last 2 columns). The percentages are column-wise).

Class	Overall	cervical	dentition	general	hard palate	hyoid	larynx	mandible	skull	angle	distance
I	14 (34.1%)	0 (0.0%)	0 (0.0%)	0 (0.0%)	0 (0.0%)	0 (0.0%)	0 (0.0%)	4 (50.0%)	10 (50.0%)	3 (33.3%)	11 (34.4%)
II	5 (12.2%)	0 (0.0%)	0 (0.0%)	1 (50.0%)	1 (50.0%)	2 (50.0%)	0 (0.0%)	0 (0.0%)	1 (5.0%)	1 (11.1%)	4 (12.5%)
III	6 (14.6%)	1 (100.0%)	0 (0.0%)	0 (0.0%)	0 (0.0%)	0 (0.0%)	0 (0.0%)	2 (25.0%)	3 (15.0%)	2 (22.2%)	4 (12.5%)
IV	16 (39.0%)	0 (0.0%)	2 (100.0%)	1 (50.0%)	1 (50.0%)	2 (50.0%)	2 (100.0%)	2 (25.0%)	6 (30.0%)	3 (33.3%)	13 (40.6%)

Supplementary references

Alfwaress F, Maaitah EA, Al-Khateeb S, Zama ZA (2015) The relationship of vocal tract dimensions and substitution of the palatal approximant /j/ for the alveolar trill /r/. International Journal of Speech-Language Pathology 17:518–526. https://doi.org/10.3109/17549507.2015.1024165

D'Errico J (2022) distance2curve. MATLAB Central File Exchange

Grave B, Brown T, Townsend G (1999) Comparison of cervicovertebral dimensions in Australian Aborigines and Caucasians. The European Journal of Orthodontics 21:127–135. https://doi.org/10.1093/ejo/21.2.127

Howells WW (1973) Cranial variation in Man: A study by multivariate analysis of patterns of difference among recent human populations. Papers of the Peabody Museum of Archaeology and Ethnology 67:1–259

Kroon D-J (2022) 2D line curvature and normals. MATLAB Central File Exchange

Legland D (2022) geom2d. MATLAB Central File Exchange

Leygue A (2022) Plane fit. MATLAB Central File Exchange

Nishimura T, Mikami A, Suzuki J, Matsuzawa T (2006) Descent of the hyoid in chimpanzees: Evolution of face flattening and speech. J Hum Evol 51:244–254. https://doi.org/10.1016/j.jhevol.2006.03.005

Reenen JF van, Allen DW (1987) The palatal vault of the Bushman (San), Vassekela and Himba. The Journal of the Dental Association of South Africa = Die Tydskrif van die Tandheelkundige Vereniging van Suid-Afrika 42:489–492

Rosseel Y (2012) Lavaan: An R Package for Structural Equation Modeling. Journal of Statistical Software 48:1–36

Schwarz D (2022) Fast and robust curve intersections. MATLAB Central File Exchange

Solow B, Barrett MJ, Brown T (1982) Craniocervical morphology and posture in Australian Aboriginals. American Journal of Physical Anthropology 59:33–45
