# Supplementary material for: The heritability of vocal tract structures estimated from structural MRI in a large cohort of Dutch twins
Source: Hum Genet. 2022 Jul 13;141(12):1905–23. doi: 10.1007/s00439-022-02469-2 (PMC9672028; doi:10.1007/s00439-022-02469-2)
Supplement: Supplementary file 2 — Supplementary file2 (XZ 31532 kb) [file 439_2022_2469_MOESM2_ESM.xz › ./full_analysis_report.html]

 

 

 

 
 
 


 


 The heritability of vocal tract structures estimated from structural MRI in a large cohort of Dutch twins 

 
 
 
 
 
 
 
 
 
 
 
 
 
 
 
 

 

 
 


 


 

 

 


 


 

 


 


 
 
 
 
 
 

 


 


 The heritability of vocal tract structures
estimated from structural MRI in a large cohort of Dutch twins 
 Full analysis report 
 Dan Dediu [ ddediu@gmail.com ] and Emily Jennings, with input from
Conor V. Dolan 
 2022-06-03 11:28:46 

 


 
 Runing the analysis 
 Please note that for running the analysis, the file
 ../data/input/measures.csv  must first be obtained from the
 Netherlands
Twin Register . This file cannot be made public, as it contains
potentially identifying information about the participants.
Nevertheless, we provide the file
 ../data/input/measures-STUB.csv , which is an empty stub, in
order to show the structure of this missing file and to allow the
understanding of what the code does. Moreover, all the other files are
provided, as is all the code and the compiled full analysis report (this
very document) that complements the published paper and its
supplementary materials. 
 
 
 The data and methods 
 
 The “raw” data 
 The “ raw ” data represents the measures taken by each
of the two raters on each of the MRI scans of the individual twins in
the study. There are, in total, 150 measures, each measured
independently by 2 raters in 677 participants (N.B., some participants
and measurements were removed due to missing data). However, some of
these measurements have obvious outliers that would affect the analysis
– their identification and removal produced the “cleaned” data, as
described below. It is this “cleaned” data that will be used in the
following analyses. 
 
 
 The “cleaned” data 
 The data cleaning and preparation are contained in the accompanying
 R  script   1_data_preparation.R  
and is briefly described below: 
 
 the 39 cases with  unknown zygosity  (MZ/DZ) were
removed, 
 followed by the identification and removal of  duplicate
cases : 6 participants with same  FISNumber  number that
participated in two different studies ( OCD  and  EMIF ) →
we kept the  OCD  data, 
 for each phenotypic measure separately, we replaced all values
farther than 3 standard deviations from the mean with missing data
(i.e., all values  x  such that | x  -
 mean ( x )| >  sd ( x ) became
 NA ). 
 
 
 
 Descriptives 
 Total number of participants: 632, of which 409 females (64.7%) and
223 males (35.3%). 
 
 
 
 
 
 
 
 
 
 
 
 
   
 ADHD 
 OCS 
 Depression 
 Aging 
 Obesity 
 Sum 
 
 
 
 
  female  
 92 
 304 
 78 
 214 
 130 
 818 
 
 
  male  
 56 
 178 
 52 
 160 
 0 
 446 
 
 
  Sum  
 148 
 482 
 130 
 374 
 130 
 1264 
 
 
 
 
 
 
 
 
 
 
 
 
 
 
 
   
 ADHD 
 OCS 
 Depression 
 Aging 
 Obesity 
 Sum 
 
 
 
 
  female  
 62.16 
 63.07 
 60 
 57.22 
 100 
 342.5 
 
 
  male  
 37.84 
 36.93 
 40 
 42.78 
 0 
 157.5 
 
 
  Sum  
 100 
 100 
 100 
 100 
 100 
 500 
 
 
 
 
 Pearson’s Chi-squared test:  tmp  
 
 
 
 
 
 
 
 Test statistic 
 df 
 P value 
 
 
 
 
 82.34 
 4 
 5.554e-17 * * * 
 
 
 
 Total number of unique twin pairs: 338, of which 48 have only one
twin member with complete data (all from the  Obesity  study),
and 2 are duplicated among studies ( Aging  and  OCD );
so, in fact we have 2 × 290 [complete twin pairs] + 1 × 48
[single-member twin pairs] + 2 × 2 [duplicated complete twin pairs] =
632. 
 There are 249 MZ twin pairs (73.2%), and 91 DZ twin pairs (26.8%), of
which 5 are concordant-sex male DZ pairs (5.5%), 6 are concordant-sex
female DZ pairs (6.6%), and 43 are discordant-sex DZ pairs (47.3%), with
the remaining 37 having only one non-missing pair member (40.7%). 
 Age: min=11, max=93.5, mean=43.46, median=39, sd=20.95 and
IQR=37.05. 
 
 Welch Two Sample t-test:  Age_MRI  by
 Sex  (continued below) 
 
 
 
 
 
 
 
 
 Test statistic 
 df 
 P value 
 Alternative hypothesis 
 
 
 
 
 1.542 
 878.2 
 0.1234 
 two.sided 
 
 
 
 
 
 
 
 
 
 
 mean in group female 
 mean in group male 
 
 
 
 
 44.14 
 42.21 
 
 
 
 
 Analysis of Variance Model 
 
 
 
 
 
 
 
 
 
 
   
 Df 
 Sum Sq 
 Mean Sq 
 F value 
 Pr(>F) 
 
 
 
 
  Study  
 4 
 469255 
 117314 
 1732 
 0 
 
 
  Residuals  
 1259 
 85281 
 67.74 
 NA 
 NA 
 
 
 
 
 
 
 
 
 
 
 
 
 
   
 diff 
 lwr 
 upr 
 p adj 
 
 
 
 
  OCS-ADHD  
 19.22 
 17.11 
 21.33 
 0 
 
 
  Depression-ADHD  
 15.02 
 12.32 
 17.72 
 0 
 
 
  Aging-ADHD  
 55.99 
 53.8 
 58.17 
 0 
 
 
  Obesity-ADHD  
 31.76 
 29.06 
 34.46 
 0 
 
 
  Depression-OCS  
 -4.2 
 -6.422 
 -1.978 
 2.798e-06 
 
 
  Aging-OCS  
 36.77 
 35.22 
 38.32 
 0 
 
 
  Obesity-OCS  
 12.54 
 10.32 
 14.77 
 0 
 
 
  Aging-Depression  
 40.97 
 38.68 
 43.26 
 0 
 
 
  Obesity-Depression  
 16.74 
 13.95 
 19.53 
 0 
 
 
  Obesity-Aging  
 -24.22 
 -26.51 
 -21.94 
 0 
 
 
 
 ICV (cm 3 ): min=890.019, max=1890.21, mean=1410.48,
median=1425.59, sd=191.36 and IQR=249.22. 
 
 Welch Two Sample t-test:  ICV * 0.001  by
 Sex  (continued below) 
 
 
 
 
 
 
 
 
 Test statistic 
 df 
 P value 
 Alternative hypothesis 
 
 
 
 
 -15.15 
 927.6 
 1.774e-46 * * * 
 two.sided 
 
 
 
 
 
 
 
 
 
 
 mean in group female 
 mean in group male 
 
 
 
 
 1355 
 1512 
 
 
 
 
 Analysis of Variance Model 
 
 
 
 
 
 
 
 
 
 
   
 Df 
 Sum Sq 
 Mean Sq 
 F value 
 Pr(>F) 
 
 
 
 
  Study  
 4 
 11507819 
 2876955 
 104.6 
 6.948e-77 
 
 
  Residuals  
 1245 
 34228689 
 27493 
 NA 
 NA 
 
 
 
 
 
 
 
 
 
 
 
 
 
   
 diff 
 lwr 
 upr 
 p adj 
 
 
 
 
  OCS-ADHD  
 -281.9 
 -324.5 
 -239.3 
 7.949e-14 
 
 
  Depression-ADHD  
 -86.71 
 -142.1 
 -31.32 
 0.0001981 
 
 
  Aging-ADHD  
 -149.1 
 -193.1 
 -105.1 
 1.286e-13 
 
 
  Obesity-ADHD  
 -125.9 
 -180.4 
 -71.48 
 3.673e-09 
 
 
  Depression-OCS  
 195.2 
 149.3 
 241.1 
 8.06e-14 
 
 
  Aging-OCS  
 132.8 
 101.5 
 164.2 
 8.127e-14 
 
 
  Obesity-OCS  
 156 
 111.2 
 200.8 
 1.255e-13 
 
 
  Aging-Depression  
 -62.36 
 -109.6 
 -15.1 
 0.002993 
 
 
  Obesity-Depression  
 -39.22 
 -96.31 
 17.88 
 0.3306 
 
 
  Obesity-Aging  
 23.14 
 -23.01 
 69.29 
 0.6472 
 
 
 
 
 
 
  Figure 1.    Descriptives for the paper. 
 
 
 
 
 
 The measures 
 There are 150 measures (bt please note that some were exact
duplicates and were subsequently removed from the analysis – please see
below), each measured independently by 2 raters in 632 participants. 
 
 
  Table 1.    Details about the phenotypes,
including their short name (the  code ; codes with a star
* represent deduplicated codes), the anatomical  domain 
and  type , their  description  and
 full names . 
 
 
 
 
code
 
 
domain
 
 
type
 
 
description
 
 
full name
 
 
 
 
 
 
CS2A
 
 
cervical
 
 
distance
 
 
Height of C2 from its base to odontoid tip
 
 
CERVICALSPINE_CS2A_C2BASE_ODONTOID_DISTANCE
 
 
 
 
CS2H
 
 
cervical
 
 
distance
 
 
Height of C2 from its base to atlas (C1)
 
 
CERVICALSPINE_CS2H_ATLAS_C2BASE_DISTANCE
 
 
 
 
CS3H
 
 
cervical
 
 
distance
 
 
Height of C3 from its base to C2 base
 
 
CERVICALSPINE_CS3H_C2BASE_C3BASE_DISTANCE
 
 
 
 
CS4H
 
 
cervical
 
 
distance
 
 
Height of C4 from its base to C3 base
 
 
CERVICALSPINE_CS4H_C3BASE_C4BASE_DISTANCE
 
 
 
 
CS5H
 
 
cervical
 
 
distance
 
 
Height of C5 from its base to C4 base
 
 
CERVICALSPINE_CS5H_C4BASE_C5BASE_DISTANCE
 
 
 
 
CS6H
 
 
cervical
 
 
distance
 
 
Height of C6 from its base to C5 base
 
 
CERVICALSPINE_CS6H_C5BASE_C6BASE_DISTANCE
 
 
 
 
CS7H
 
 
cervical
 
 
distance
 
 
Height of C7 from its base to C6 base
 
 
CERVICALSPINE_CS7H_C6BASE_C7BASE_DISTANCE
 
 
 
 
DAII
 
 
dentition
 
 
angle
 
 
Angle between the nasal cavity floor and inferior right central incisor
 
 
DENITION_DAII_INCISIONINFAPEXINFLINE_NSANSPLINE_ANGLE
 
 
 
 
DAIM
 
 
dentition
 
 
angle
 
 
Angle between the mandible body line (defined using the menton and mean
gonion locations) and inferior right central incisor
 
 
DENITION_DAIM_INCISIONINFAPEXINFLINE_MENTONGONIONMEANLINE_
 
 
 
 
DAIP
 
 
dentition
 
 
angle
 
 
Angle between the mandible body line (defined using the pogonion and
mean gonion locations) and inferior right central incisor
 
 
DENITION_DAIP_INCINFAPEXINFLINE_POGGONMEANLINE_ANGLE
 
 
 
 
DAIS
 
 
dentition
 
 
angle
 
 
Angle between the nasal cavity floor and superior right central incisor
 
 
DENITION_DAIS_INCISIONSUPAPEXSUPLINE_NSANSPLINE_ANGLE
 
 
 
 
DMAC
 
 
dentition
 
 
curvature
 
 
Curvature of the maxillary dental arch
 
 
DENITION_DMAC_MAXILLARYDENTALARCH_COMPLETETRACE_MEANCURVA
 
 
 
 
DICD
 
 
dentition
 
 
distance
 
 
Width of dental arch between the canines
 
 
DENITION_DICD_INTERCANINE_DISTANCE
 
 
 
 
DIIL
 
 
dentition
 
 
distance
 
 
Distance between apex inferior and incisor inferior
 
 
DENITION_DIIL_APEXINFERIOR_INCISIONINFERIOR_DISTANCE
 
 
 
 
DIMD
 
 
dentition
 
 
distance
 
 
Width of dental arch between the second molars
 
 
DENITION_DIMD_INTERM2_DISTANCE
 
 
 
 
DIPD
 
 
dentition
 
 
distance
 
 
Width of dental arch between the second premolars
 
 
DENITION_DIPD_INTERPM2_DISTANCE
 
 
 
 
DSIL
 
 
dentition
 
 
distance
 
 
Distance between apex superior and incisor superior
 
 
DENITION_DSIL_APEXSUPERIOR_INCISIONSUPERIOR_DISTANCE
 
 
 
 
DDAP
 
 
dentition
 
 
Procrustes dist.
 
 
Procrustes distance of maxillary dental arch tracing
 
 
DENTITION_DDAP_DENTALARCHTRACE_MATLABPROCDISTANCE
 
 
 
 
SVTh
 
 
general
 
 
distance
 
 
Length of the horizontal supralaryngeal vocal tract (using the
anterior-most point of the hard palate and the atlas)
 
 
GENERAL_SVTH_ATLAS_HPA_DISTANCE
 
 
 
 
SVTh*
 
 
general
 
 
distance
 
 
Length of the horizontal supralaryngeal vocal tract (using the prosthion
and the atlas)
 
 
GENERAL_SVTH_ATLAS_PROSTHION_DISTANCE
 
 
 
 
SVTv
 
 
general
 
 
distance
 
 
Length of the vertical supralaryngeal vocal tract (using the petiole of
the epiglottis and and the posterior nasal spine)
 
 
GENERAL_SVTV_NSP_EPIGLOTTISPETIOLE_DISTANCE
 
 
 
 
SVTv*
 
 
general
 
 
distance
 
 
Length of the vertical supralaryngeal vocal tract (using the corniculate
tubercle and the posterior nasal spine)
 
 
GENERAL_SVTV_NSP_CORNICULATETUBERCLE_DISTANCE
 
 
 
 
GAPD
 
 
general
 
 
Procrustes dist.
 
 
Procrustes distance of all available landmarks registered against the
mean configuration defined by scans which contain all landmarks
 
 
GENERAL_GAPD_ALLPOINTSET_MATLABPROCDISTANCE
 
 
 
 
GRPD
 
 
general
 
 
Procrustes dist.
 
 
Procrustes distance of common points (excludes points not found in all
scans)
 
 
GENERAL_GRPD_REGISTRATIONPOINTSET_MATLABPROCDISTANCE
 
 
 
 
HACP
 
 
hard palate
 
 
angle
 
 
Angle between the line from the intermolar projection (on midsagittal
hard palate trace) to the anterior hard palate and the occlusal plane
(containing pr, cal, car, pml, pmr, eml, emr)
 
 
HARDPALATE_HACP_CANPM2PROJ_OCCLUSALPLANE_POSTALVSLOPE_ANGLE
 
 
 
 
HARC
 
 
hard palate
 
 
angle
 
 
Angle between the line from the intercanine projection to the anterior
hard palate and the occlusal plane (containing pr, cal, car, pml, pmr,
eml, emr)
 
 
HARDPALATE_HARC_HPAINTERCANINEPROJ_OCCLUSALPLANE_ALVRIDGE_
 
 
 
 
HARF
 
 
hard palate
 
 
angle
 
 
Angle between the line from the interpremolar projection (on midsagittal
hard palate trace) to the anterior hard palate and the occlusal plane
(containing pr, cal, car, pml, pmr, eml, emr)
 
 
HARDPALATE_HARF_FRONTSEGSLOPE_OCCLUSALPLANE_ALVRIDGE_ANGLE
 
 
 
 
HDMP
 
 
hard palate
 
 
angle
 
 
Angle between line from the intermolar projection to the interpremolar
projection (both of these on the midsagittal hard palate trace) and the
occlusal plane (containing pr, cal, car, pml, pmr, eml, emr)
 
 
HARDPALATE_HDMP_M2PM2PROJ_OCCLUSALPLANE_PALATEDOME_ANGLE
 
 
 
 
HCCC
 
 
hard palate
 
 
curvature
 
 
Curvature of coronal hard palate profile at the canines
 
 
HARDPALATE_HCCC_CORONALCANINE_COMPLETETRACE_MEANCURVATURE
 
 
 
 
HM2C
 
 
hard palate
 
 
curvature
 
 
Curvature of coronal hard palate profile at the second molars
 
 
HARDPALATE_HM2C_CORONALM2_COMPLETETRACE_MEANCURVATURE
 
 
 
 
HMAC
 
 
hard palate
 
 
curvature
 
 
Curvature of alveolar ridge of midsagittal hard palate tracing (indices
16 to 20)
 
 
HARDPALATE_HMAC_MIDSAGITTAL_ALVRIDGEINDS15TO19_MEANCURVATURE
 
 
 
 
HMDC
 
 
hard palate
 
 
curvature
 
 
Curvature of dome of midsagittal hard palate tracing (indices 1 to 15)
 
 
HARDPALATE_HMDC_MIDSAGITTAL_DOMEINDICES1TO15_MEANCURVATURE
 
 
 
 
HP2C
 
 
hard palate
 
 
curvature
 
 
Curvature of coronal hard palate profile at the second premolars
 
 
HARDPALATE_HP2C_CORONALPM2_COMPLETETRACE_MEANCURVATURE
 
 
 
 
HACL
 
 
hard palate
 
 
distance
 
 
Length of hard palate from canines to its anterior most point
 
 
HARDPALATE_HACL_HPA_INTERCANINE_DISTANCE
 
 
 
 
HAML
 
 
hard palate
 
 
distance
 
 
Length of hard palate from second molars to its anterior most point
 
 
HARDPALATE_HAML_HPA_INTERM2_DISTANCE
 
 
 
 
HAPL
 
 
hard palate
 
 
distance
 
 
Length of hard palate from second premolars to its anterior most point
 
 
HARDPALATE_HAPL_HPA_INTERPM2_DISTANCE
 
 
 
 
HBPL
 
 
hard palate
 
 
distance
 
 
Length (anteroposterior distance) of hard palate (lower face)
 
 
HARDPALATE_HBPL_BASION_PROSTHION_DISTANCE
 
 
 
 
HCTH
 
 
hard palate
 
 
distance
 
 
Height of coronal hard palate profile at the canines
 
 
HARDPALATE_HCTH_CORONALCANINETRACE_HEIGHT
 
 
 
 
HHPL
 
 
hard palate
 
 
distance
 
 
Length (anteroposterior distance) of hard palate
 
 
HARDPALATE_HHPL_HPA_HPP_DISTANCE
 
 
 
 
HICH
 
 
hard palate
 
 
distance
 
 
Height of hard palate at midpoint between the canines
 
 
HARDPALATE_HICH_INTERCANINE_HEIGHT
 
 
 
 
HICL
 
 
hard palate
 
 
distance
 
 
Length of hard palate from canines to incisors
 
 
HARDPALATE_HICL_INTERCANINE_INCISIONSUPERIOR_DISTANCE
 
 
 
 
HICL*
 
 
hard palate
 
 
distance
 
 
Length of hard palate from canines to prosthion
 
 
HARDPALATE_HICL_INTERCANINE_PROSTHION_DISTANCE
 
 
 
 
HIMH
 
 
hard palate
 
 
distance
 
 
Height of hard palate at midpoint between the second molars
 
 
HARDPALATE_HIMH_INTERM2_HEIGHT
 
 
 
 
HIML
 
 
hard palate
 
 
distance
 
 
Length of hard palate from second molars to incisors
 
 
HARDPALATE_HIML_INTERM2_INCISIONSUPERIOR_DISTANCE
 
 
 
 
HIML*
 
 
hard palate
 
 
distance
 
 
Length of hard palate from second molars to prosthion
 
 
HARDPALATE_HIML_INTERM2_PROSTHION_DISTANCE
 
 
 
 
HIPH
 
 
hard palate
 
 
distance
 
 
Height of hard palate at midpoint between the second premolars
 
 
HARDPALATE_HIPH_INTERPM2_HEIGHT
 
 
 
 
HIPL
 
 
hard palate
 
 
distance
 
 
Length of hard palate from second premolars to incisors
 
 
HARDPALATE_HIPL_INTERPM2_INCISIONSUPERIOR_DISTANCE
 
 
 
 
HIPL*
 
 
hard palate
 
 
distance
 
 
Length of hard palate from second premolars to prosthion
 
 
HARDPALATE_HIPL_INTERPM2_PROSTHION_DISTANCE
 
 
 
 
HMTC
 
 
hard palate
 
 
distance
 
 
Curvature of total midsagittal hard palate tracing
 
 
HARDPALATE_HMTC_MIDSAGITTAL_COMPLETETRACE_MEANCURVATURE
 
 
 
 
HMTH
 
 
hard palate
 
 
distance
 
 
Height of coronal hard palate profile at the second molars
 
 
HARDPALATE_HMTH_CORONALM2TRACE_HEIGHT
 
 
 
 
HNAP
 
 
hard palate
 
 
distance
 
 
Height of hard palate on its external anterior surface
 
 
HARDPALATE_HNAP_NSA_PROSTHION_DISTANCE
 
 
 
 
HNSL
 
 
hard palate
 
 
distance
 
 
Length (anteroposterior distance) of nasal cavity floor
 
 
HARDPALATE_HNSL_NSA_NSP_DISTANCE
 
 
 
 
HPTH
 
 
hard palate
 
 
distance
 
 
Height of coronal hard palate profile at the second premolars
 
 
HARDPALATE_HPTH_CORONALPM2TRACE_HEIGHT
 
 
 
 
HCCP
 
 
hard palate
 
 
Procrustes dist.
 
 
Procrustes distance of canine coronal hard palate tracing
 
 
HARDPALATE_HCCP_CORONALCANINETRACE_MATLABPROCDISTANCE
 
 
 
 
HCMP
 
 
hard palate
 
 
Procrustes dist.
 
 
Procrustes distance of second molar coronal hard palate tracing
 
 
HARDPALATE_HCMP_CORONALM2TRACE_MATLABPROCDISTANCE
 
 
 
 
HCPP
 
 
hard palate
 
 
Procrustes dist.
 
 
Procrustes distance of second premolar coronal hard palate tracing
 
 
HARDPALATE_HCPP_CORONALPM2TRACE_MATLABPROCDISTANCE
 
 
 
 
HMSP
 
 
hard palate
 
 
Procrustes dist.
 
 
Procrustes distance of midsagittal hard palate tracing
 
 
HARDPALATE_HMSP_MIDSAGITTALTRACE_MATLABPROCDISTANCE
 
 
 
 
HBC2
 
 
hyoid
 
 
distance
 
 
Distance between C2 body and hyoidale
 
 
HYOIDBONE_HBC2_C2BASE_HYOIDALE_DISTANCE
 
 
 
 
HBC3
 
 
hyoid
 
 
distance
 
 
Distance between C3 body and hyoidale
 
 
HYOIDBONE_HBC3_C3BASE_HYOIDALE_DISTANCE
 
 
 
 
HBC4
 
 
hyoid
 
 
distance
 
 
Distance between C4 body and hyoidale
 
 
HYOIDBONE_HBC4_C4BASE_HYOIDALE_DISTANCE
 
 
 
 
HBNP
 
 
hyoid
 
 
distance
 
 
Distance between posterior nasal spine and hyoidale
 
 
HYOIDBONE_HBNP_NSP_HYOIDALE_DISTANCE
 
 
 
 
HBPG
 
 
hyoid
 
 
distance
 
 
Distance between pogonion (chin) and hyoidale
 
 
HYOIDBONE_HBPG_POGONION_HYOIDALE_DISTANCE
 
 
 
 
L2CT
 
 
larynx
 
 
distance
 
 
Height of larynx (using corniculate tubercle) relative to C2
 
 
LARYNX_L2CT_C2BASE_CORNICULATETUBERCLE_DISTANCE
 
 
 
 
L2EA
 
 
larynx
 
 
distance
 
 
Height of larynx (using apex of epiglottis) relative to C2
 
 
LARYNX_L2EA_C2BASE_EPIGLOTTISAPEX_DISTANCE
 
 
 
 
L2EP
 
 
larynx
 
 
distance
 
 
Height of larynx (using petiole of epiglottis) relative to C2
 
 
LARYNX_L2EP_C2BASE_EPIGLOTTISPETIOLE_DISTANCE
 
 
 
 
L3CT
 
 
larynx
 
 
distance
 
 
Height of larynx (using corniculate tubercle) relative to C3
 
 
LARYNX_L3CT_C3BASE_CORNICULATETUBERCLE_DISTANCE
 
 
 
 
L3EA
 
 
larynx
 
 
distance
 
 
Height of larynx (using apex of epiglottis) relative to C3
 
 
LARYNX_L3EA_C3BASE_EPIGLOTTISAPEX_DISTANCE
 
 
 
 
L3EP
 
 
larynx
 
 
distance
 
 
Height of larynx (using petiole of epiglottis) relative to C3
 
 
LARYNX_L3EP_C3BASE_EPIGLOTTISPETIOLE_DISTANCE
 
 
 
 
L4CT
 
 
larynx
 
 
distance
 
 
Height of larynx (using corniculate tubercle) relative to C4
 
 
LARYNX_L4CT_C4BASE_CORNICULATETUBERCLE_DISTANCE
 
 
 
 
L4EA
 
 
larynx
 
 
distance
 
 
Height of larynx (using apex of epiglottis) relative to C4
 
 
LARYNX_L4EA_C4BASE_EPIGLOTTISAPEX_DISTANCE
 
 
 
 
L4EP
 
 
larynx
 
 
distance
 
 
Height of larynx (using petiole of epiglottis) relative to C4
 
 
LARYNX_L4EP_C4BASE_EPIGLOTTISPETIOLE_DISTANCE
 
 
 
 
L5CT
 
 
larynx
 
 
distance
 
 
Height of larynx (using corniculate tubercle) relative to C5
 
 
LARYNX_L5CT_C5BASE_CORNICULATETUBERCLE_DISTANCE
 
 
 
 
L5EA
 
 
larynx
 
 
distance
 
 
Height of larynx (using apex of epiglottis) relative to C5
 
 
LARYNX_L5EA_C5BASE_EPIGLOTTISAPEX_DISTANCE
 
 
 
 
L5EP
 
 
larynx
 
 
distance
 
 
Height of larynx (using petiole of epiglottis) relative to C5
 
 
LARYNX_L5EP_C5BASE_EPIGLOTTISPETIOLE_DISTANCE
 
 
 
 
LACT
 
 
larynx
 
 
distance
 
 
Height of larynx (using corniculate tubercle) relative to C1
 
 
LARYNX_LACT_ATLAS_CORNICULATETUBERCLE_DISTANCE
 
 
 
 
LAEA
 
 
larynx
 
 
distance
 
 
Height of larynx (using apex of epiglottis) relative to C1
 
 
LARYNX_LAEA_ATLAS_EPIGLOTTISAPEX_DISTANCE
 
 
 
 
LAEP
 
 
larynx
 
 
distance
 
 
Height of larynx (using petiole of epiglottis) relative to C1
 
 
LARYNX_LAEP_ATLAS_EPIGLOTTISPETIOLE_DISTANCE
 
 
 
 
LEPL
 
 
larynx
 
 
distance
 
 
Height of epiglottis from petiole to apex
 
 
LARYNX_LEPL_EPIGLOTTISAPEX_EPIGLOTTISPETIOLE_DISTANCE
 
 
 
 
LHCT
 
 
larynx
 
 
distance
 
 
Height of larynx (using corniculate tubercle) relative to hyoidale
 
 
LARYNX_LHCT_HYOIDALE_CORNICULATETUBERCLE_DISTANCE
 
 
 
 
LHEA
 
 
larynx
 
 
distance
 
 
Height of larynx (using apex of epiglottis) relative to hyoidale
 
 
LARYNX_LHEA_HYOIDALE_EPIGLOTTISAPEX_DISTANCE
 
 
 
 
LHEP
 
 
larynx
 
 
distance
 
 
Height of larynx (using petiole of epiglottis) relative to hyoidale
 
 
LARYNX_LHEP_HYOIDALE_EPIGLOTTISPETIOLE_DISTANCE
 
 
 
 
LNCT
 
 
larynx
 
 
distance
 
 
Height of larynx (using corniculate tubercle) relative to posterior
nasal spine
 
 
LARYNX_LNCT_NSP_CORNICULATETUBERCLE_DISTANCE
 
 
 
 
LNEA
 
 
larynx
 
 
distance
 
 
Height of larynx (using apex of epiglottis) relative to posterior nasal
spine
 
 
LARYNX_LNEA_NSP_EPIGLOTTISAPEX_DISTANCE
 
 
 
 
LNEP
 
 
larynx
 
 
distance
 
 
Height of larynx (using petiole of epiglottis) relative to posterior
nasal spine
 
 
LARYNX_LNEP_NSP_EPIGLOTTISPETIOLE_DISTANCE
 
 
 
 
MCGM
 
 
mandible
 
 
angle
 
 
Angle of the mandible using the menton and the mean locations of the
condyles and angles (gonion)
 
 
MANDIBLE_MCGM_GONIONMEANCONYLIONMEAN_GONIONMEANMENTON_ANGLE
 
 
 
 
MCGP
 
 
mandible
 
 
angle
 
 
Angle of the mandible using the pogonion and the mean locations of the
condyles and angles (gonion)
 
 
MANDIBLE_MCGP_GONIONMEANCONYLIONMEAN_GONIONMEANPOGONION_A
 
 
 
 
MCGD
 
 
mandible
 
 
distance
 
 
Height of ramus of mandible using mean location of condyles and angles
(gonion)
 
 
MANDIBLE_MCGD_CONDYLIONMEAN_GONIONMEAN_DISTANCE
 
 
 
 
MCGL
 
 
mandible
 
 
distance
 
 
Height of left ramus of mandible
 
 
MANDIBLE_MCGL_CONDYLIONLEFT_GONIONLEFT_DISTANCE
 
 
 
 
MCGR
 
 
mandible
 
 
distance
 
 
Height of right ramus of mandible
 
 
MANDIBLE_MCGR_CONDYLIONRIGHT_GONIONRIGHT_DISTANCE
 
 
 
 
MCMD
 
 
mandible
 
 
distance
 
 
Length of body of mandible using menton and mean location of condyles
 
 
MANDIBLE_MCMD_CONDYLIONMEAN_MENTON_DISTANCE
 
 
 
 
MCPD
 
 
mandible
 
 
distance
 
 
Length of body of mandible using pogonion and mean location of condyles
 
 
MANDIBLE_MCPD_CONDYLIONMEAN_POGONION_DISTANCE
 
 
 
 
MGMD
 
 
mandible
 
 
distance
 
 
Length of body of mandible using menton and mean location of angles
(gonion)
 
 
MANDIBLE_MGMD_GONIONMEAN_MENTON_DISTANCE
 
 
 
 
MGPD
 
 
mandible
 
 
distance
 
 
Length of body of mandible using pogonion and mean location of angles
(gonion)
 
 
MANDIBLE_MGPD_GONIONMEAN_POGONION_DISTANCE
 
 
 
 
MICD
 
 
mandible
 
 
distance
 
 
Width of mandible between the condyles
 
 
MANDIBLE_MICD_INTERCONDYLION_DISTANCE
 
 
 
 
MIGD
 
 
mandible
 
 
distance
 
 
Width of mandible between the angles (gonion)
 
 
MANDIBLE_MIGD_INTERGONIAL_DISTANCE
 
 
 
 
MMPG
 
 
mandible
 
 
distance
 
 
Height of chin
 
 
MANDIBLE_MMPG_MENTON_POGONION_DISTANCE
 
 
 
 
MPGL
 
 
mandible
 
 
distance
 
 
Length of left side of body of mandible
 
 
MANDIBLE_MPGL_POGONION_GONIONLEFT_DISTANCE
 
 
 
 
MPGR
 
 
mandible
 
 
distance
 
 
Length of right side of body of mandible
 
 
MANDIBLE_MPGR_POGONION_GONIONRIGHT_DISTANCE
 
 
 
 
OCHL
 
 
oral
 
 
ratio
 
 
Ratio between intercanine height of hard palate and intercanine distance
to the anterior-most point of the hard palate (i.e., a hard palate
height:length ratio)
 
 
ORALCAVITY_OCHL_INTERCANHEIGHT_INTERCANHPADIST_HLRATIO
 
 
 
 
OCHW
 
 
oral
 
 
ratio
 
 
Ratio between intercanine height of hard palate and intercanine width
(i.e., a hard palate height:width ratio)
 
 
ORALCAVITY_OCHW_INTERCANHEIGHT_INTERCANDIST_HWRATIO
 
 
 
 
OCLW
 
 
oral
 
 
ratio
 
 
Ratio between intercanine distance to the anterior-most point of the
hard palate and intercanine width (i.e., a hard palate length:width
ratio)
 
 
ORALCAVITY_OCLW_INTERCANDIST_INTERCANHPADIST_WLRATIO
 
 
 
 
OCMR
 
 
oral
 
 
ratio
 
 
Ratio of intercanine width to intermolar width
 
 
ORALCAVITY_OCMR_INTERCANDIST_INTERM2DIST_RATIO
 
 
 
 
OCPR
 
 
oral
 
 
ratio
 
 
Ratio of intercanine width to interpremolar width
 
 
ORALCAVITY_OCPR_INTERCANDIST_INTERPM2DIST_RATIO
 
 
 
 
OMHL
 
 
oral
 
 
ratio
 
 
Ratio between intermolar height of hard palate and intermolar distance
to the anterior-most point of the hard palate (i.e., a hard palate
height:length ratio)
 
 
ORALCAVITY_OMHL_INTERM2HEIGHT_INTERM2HPADIST_HLRATIO
 
 
 
 
OMHW
 
 
oral
 
 
ratio
 
 
Ratio between intermolar height of hard palate and intermolar width
(i.e., a hard palate height:width ratio)
 
 
ORALCAVITY_OMHW_INTERM2HEIGHT_INTERM2DIST_HWRATIO
 
 
 
 
OMLW
 
 
oral
 
 
ratio
 
 
Ratio between intermolar distance to the anterior-most point of the hard
palate and intermolar width of hard palate (i.e., a hard palate
length:width ratio)
 
 
ORALCAVITY_OMLW_INTERM2DIST_INTERM2HPADIST_WLRATIO
 
 
 
 
OPHL
 
 
oral
 
 
ratio
 
 
Ratio between interpremolar height of hard palate and interpremolar
distance to the anterior-most point of the hard palate (i.e., a hard
palate height:length ratio)
 
 
ORALCAVITY_OPHL_INTERPM2HEIGHT_INTERPM2HPADIST_HLRATIO
 
 
 
 
OPHW
 
 
oral
 
 
ratio
 
 
Ratio between interpremolar height of hard palate and interpremolar
width (i.e., a hard palate height:width ratio)
 
 
ORALCAVITY_OPHW_INTERPM2HEIGHT_INTERPM2DIST_HWRATIO
 
 
 
 
OPLW
 
 
oral
 
 
ratio
 
 
Ratio between interpremolar distance to the anterior-most point of the
hard palate and interpremolar width (i.e., a hard palate length:width
ratio)
 
 
ORALCAVITY_OPLW_INTERPM2DIST_INTERPM2HPADIST_WLRATIO
 
 
 
 
OPMR
 
 
oral
 
 
ratio
 
 
Ratio of interpremolar width to intermolar width
 
 
ORALCAVITY_OPMR_INTERPM2DIST_INTERM2DIST_RATIO
 
 
 
 
PPW2
 
 
pharynx
 
 
curvature
 
 
Curvature of pharynx wall down to projection of C2
 
 
PHARYNX_PPW2_PHARYNXWALL_TOC2PROJECTION_MEANCURVATURE
 
 
 
 
PPW3
 
 
pharynx
 
 
curvature
 
 
Curvature of pharynx wall down to projection of C3
 
 
PHARYNX_PPW3_PHARYNXWALL_TOC3PROJECTION_MEANCURVATURE
 
 
 
 
PPW4
 
 
pharynx
 
 
curvature
 
 
Curvature of pharynx wall down to projection of C4
 
 
PHARYNX_PPW4_PHARYNXWALL_TOC4PROJECTION_MEANCURVATURE
 
 
 
 
PPW5
 
 
pharynx
 
 
curvature
 
 
Curvature of pharynx wall down to projection of C5
 
 
PHARYNX_PPW5_PHARYNXWALL_TOC5PROJECTION_MEANCURVATURE
 
 
 
 
PPW6
 
 
pharynx
 
 
curvature
 
 
Curvature of pharynx wall down to projection of C6
 
 
PHARYNX_PPW6_PHARYNXWALL_TOC6PROJECTION_MEANCURVATURE
 
 
 
 
PPW7
 
 
pharynx
 
 
curvature
 
 
Curvature of pharynx wall down to projection of C7
 
 
PHARYNX_PPW7_PHARYNXWALL_TOC7PROJECTION_MEANCURVATURE
 
 
 
 
PPWC
 
 
pharynx
 
 
curvature
 
 
Curvature of pharynx wall complete trace
 
 
PHARYNX_PPWC_PHARYNXWALL_COMPLETETRACE_MEANCURVATURE
 
 
 
 
PNNP
 
 
pharynx
 
 
distance
 
 
Length (anteroposterior distance) of nasopharynx
 
 
PHARYNX_PNNP_NSP_NASOPHARYNX_DISTANCE
 
 
 
 
PWTP
 
 
pharynx
 
 
Procrustes dist.
 
 
Procrustes distance of pharyngeal wall tracing
 
 
PHARYNX_PWTP_MIDSAGITTALTRACE_MATLABPROCDISTANCE
 
 
 
 
AANP
 
 
skull
 
 
angle
 
 
Angle between the line from nasion to the anterior nasal spine and the
line from nasion to prosthion
 
 
SKULLANGLE_AANP_NASIONNSA_NASIONPROSTHION_ANGLE
 
 
 
 
AASN
 
 
skull
 
 
angle
 
 
Angle between the line from nasion to the anterior nasal spine and the
line from nasion to sella
 
 
SKULLANGLE_AASN_NASIONNSA_NASIONSELLA_ANGLE
 
 
 
 
ABSN
 
 
skull
 
 
angle
 
 
Angle between the line from sella to basion and the line from sella to
nasion
 
 
SKULLANGLE_ABSN_SELLABASION_SELLANASION_ANGLE
 
 
 
 
ACSN
 
 
skull
 
 
angle
 
 
Angle between the line from sella to the mean location of the condyles
and the line from sella to the nasion
 
 
SKULLANGLE_ACSN_SELLACONYLIONMEAN_SELLANASION_ANGLE
 
 
 
 
ALNS
 
 
skull
 
 
angle
 
 
Angle between line from nasion to sella and the nasal cavity floor
 
 
SKULLANGLE_ALNS_NASIONSELLALINE_NSANSPLINE_ANGLE
 
 
 
 
ANNF
 
 
skull
 
 
angle
 
 
Angle between the floor of the nasal cavity and the Frankfort horizontal
plane
 
 
SKULLANGLE_ANNF_NSANSP_SELLAFRANFORTPLANEPERP_ANGLE
 
 
 
 
ANSF
 
 
skull
 
 
angle
 
 
Angle between the line from nasion to sella and the Frankfort horizontal
plane
 
 
SKULLANGLE_ANSF_SELLANASION_SELLAFRANFORTPLANEPERP_ANGLE
 
 
 
 
APNP
 
 
skull
 
 
angle
 
 
Angle between the line from nasion to pogonion and the line from nasion
to prosthion
 
 
SKULLANGLE_APNP_NASIONPOGONION_NASIONPROSTHION_ANGLE
 
 
 
 
APNS
 
 
skull
 
 
angle
 
 
Angle between the line from nasion to pogonion and the line from nasion
to sella
 
 
SKULLANGLE_APNS_NASIONPOGONION_NASIONSELLA_ANGLE
 
 
 
 
APSN
 
 
skull
 
 
angle
 
 
Angle between the line from sella to the posterior nasal spine and the
line from sella to nasion
 
 
SKULLANGLE_APSN_SELLANSP_SELLANASION_ANGLE
 
 
 
 
ASCG
 
 
skull
 
 
angle
 
 
Angle between the line from the mean gonion location to the mean condyle
location and the line from the mean gonion location to menton
 
 
SKULLANGLE_ASCG_GONIONMEANCONDYLIONMEAN_GONIONMEANMENTON_A
 
 
 
 
ASCG*
 
 
skull
 
 
angle
 
 
Angle between the line from the mean condyle location to sella and the
line from the mean condyle location to the mean gonion location
 
 
SKULLANGLE_ASCG_CONDMEANSELLA_CONDMEANGONIONMEAN_ANGLE
 
 
 
 
ASNP
 
 
skull
 
 
angle
 
 
Angle between the line from nasion to sella and the line from nasion to
prosthion
 
 
SKULLANGLE_ASNP_NASIONSELLA_NASIONPROSTHION_ANGLE
 
 
 
 
SBAN
 
 
skull
 
 
distance
 
 
Length of anterior basicranium (using nasion)
 
 
SKULL_SBAN_BASION_NASION_DISTANCE
 
 
 
 
SBAP
 
 
skull
 
 
distance
 
 
Length of facial skeleton (using prosthion)
 
 
SKULL_SBAP_BASION_PROSTHION_DISTANCE
 
 
 
 
SBAS
 
 
skull
 
 
distance
 
 
Length of middle basicranium (using sella)
 
 
SKULL_SBAS_BASION_SELLA_DISTANCE
 
 
 
 
SBNA
 
 
skull
 
 
distance
 
 
Length of facial skeleton (using anterior nasal spine)
 
 
SKULL_SBNA_BASION_NSA_DISTANCE
 
 
 
 
SBNP
 
 
skull
 
 
distance
 
 
Distance between basion and posterior nasal spine (using posterior nasal
spine)
 
 
SKULL_SBNP_BASION_NSP_DISTANCE
 
 
 
 
SHBN
 
 
skull
 
 
distance
 
 
Length of head
 
 
SKULL_SHBN_HEADBACK_NASION_DISTANCE
 
 
 
 
SHSW
 
 
skull
 
 
distance
 
 
Width of head
 
 
SKULL_SHSW_HEADSIDELEFT_HEADSIDERIGHT_DISTANCE
 
 
 
 
SIOD
 
 
skull
 
 
distance
 
 
Distance between the eyes (infraorbital distance)
 
 
SKULL_SIOD_INTERORBITALE_DISTANCE
 
 
 
 
SNAM
 
 
skull
 
 
distance
 
 
Height of facial skeleton (using menton)
 
 
SKULL_SNAM_NASION_MENTON_DISTANCE
 
 
 
 
SNAP
 
 
skull
 
 
distance
 
 
Height of facial skeleton (using pogonion)
 
 
SKULL_SNAP_NASION_POGONION_DISTANCE
 
 
 
 
SNNA
 
 
skull
 
 
distance
 
 
Height of facial skeleton (using anterior nasal spine)
 
 
SKULL_SNNA_NASION_NSA_DISTANCE
 
 
 
 
SNNP
 
 
skull
 
 
distance
 
 
Length of anterior basicranium (using nasion and posterior nasal spine)
 
 
SKULL_SNNP_NASION_NSP_DISTANCE
 
 
 
 
SNOL
 
 
skull
 
 
distance
 
 
Distance from left infraorbital pit to bridge of nose
 
 
SKULL_SNOL_NASION_ORBITALELEFT_DISTANCE
 
 
 
 
SNOR
 
 
skull
 
 
distance
 
 
Distance from right infraorbital pit to bridge of nose
 
 
SKULL_SNOR_NASION_ORBITALERIGHT_DISTANCE
 
 
 
 
SSEG
 
 
skull
 
 
distance
 
 
Height of basicranium relative to mean condyle location
 
 
SKULL_SSEG_SELLA_CONDYLIONMEAN_DISTANCE
 
 
 
 
SSEN
 
 
skull
 
 
distance
 
 
Length of anterior basicranium (between sella and nasion)
 
 
SKULL_SSEN_SELLA_NASION_DISTANCE
 
 
 
 
SSEN*
 
 
skull
 
 
distance
 
 
Height of posterior nasal cavity
 
 
SKULL_SSEN_SELLA_NSP_DISTANCE
 
 
 
 
SPUN
 
 
soft palate
 
 
distance
 
 
Length of soft palate from posterior nasal spine to uvula tip
 
 
SOFTPALATE_SPUN_UVULA_NSP_DISTANCE
 
 
 
 
 
 Distributions and summaries 
 
 Tables 
 
 By domain 
  Counts:  
 
 Table continues below 
 
 
 
 
 
 
 
 
 
 
 
 cervical 
 dentition 
 general 
 hard palate 
 hyoid 
 larynx 
 mandible 
 
 
 
 
 7 
 11 
 6 
 33 
 5 
 22 
 14 
 
 
 
 
 
 
 
 
 
 
 
 
 oral 
 pharynx 
 skull 
 soft palate 
 
 
 
 
 12 
 9 
 30 
 1 
 
 
 
  Percents (%):  
 
 Table continues below 
 
 
 
 
 
 
 
 
 
 
 
 cervical 
 dentition 
 general 
 hard palate 
 hyoid 
 larynx 
 mandible 
 
 
 
 
 4.667 
 7.333 
 4 
 22 
 3.333 
 14.67 
 9.333 
 
 
 
 
 
 
 
 
 
 
 
 
 oral 
 pharynx 
 skull 
 soft palate 
 
 
 
 
 8 
 6 
 20 
 0.6667 
 
 
 
 
 
 By type 
  Counts:  
 
 
 
 
 
 
 
 
 
 
 angle 
 curvature 
 distance 
 Procrustes dist. 
 ratio 
 
 
 
 
 23 
 13 
 94 
 8 
 12 
 
 
 
  Percents (%):  
 
 
 
 
 
 
 
 
 
 
 angle 
 curvature 
 distance 
 Procrustes dist. 
 ratio 
 
 
 
 
 15.33 
 8.667 
 62.67 
 5.333 
 8 
 
 
 
 
 
 
 Histograms (& QQ-plots) 
 
 Each measure separately 
 
 
 
  Figure 2.    Histograms of the measures split
by  sex  (red=females, blue=males) but not by  rater .
Please note that the  x  and  y  scales are independent
between plots. 
 
 
 
 
 
  Figure 3.    QQ-plots of the measures split
by  sex  (red=females, blue=males) but not by  rater .
Please note that the  x  and  y  scales are independent
between plots. 
 
 
 
 
 By domain 
 
 
 
  Figure 4.    Histograms of the measures split
by  sex  (red=females, blue=males) and  domain . Please
note that the  x  and  y  scales are independent between
plots. 
 
 
 
 
 By type 
 
 
 
  Figure 5.    Histograms of the measures split
by  sex  (red=females, blue=males) and  type . Please note
that the  x  and  y  scales are independent between
plots. 
 
 
 
 
 
 Summaries 
 
 Across all participants 
 
 
  Table 2.    Summary statistics for all
measures across all participants. Please note that, for a normal
distribution, the expected skewness is 0.0 and kurtosis is 3.0. The
 p -values are those of the Shapiro-Wilk normality test. 
 
 
 
 
 
 
min
 
 
max
 
 
mean
 
 
median
 
 
sd
 
 
iqr
 
 
kurtosis
 
 
skewness
 
 
shapiro.p
 
 
 
 
 
 
CS2A
 
 
28.18
 
 
50.80
 
 
37.60
 
 
37.26
 
 
3.58
 
 
4.76
 
 
3.43
 
 
0.50
 
 
6.38e-10
 
 
 
 
CS2H
 
 
22.20
 
 
45.29
 
 
32.29
 
 
32.06
 
 
3.61
 
 
4.56
 
 
3.40
 
 
0.35
 
 
1.01e-06
 
 
 
 
CS3H
 
 
11.18
 
 
25.20
 
 
18.15
 
 
18.06
 
 
2.26
 
 
2.96
 
 
3.05
 
 
0.26
 
 
2.94e-05
 
 
 
 
CS4H
 
 
10.05
 
 
24.10
 
 
17.04
 
 
17.01
 
 
2.19
 
 
2.80
 
 
3.27
 
 
0.24
 
 
1.15e-05
 
 
 
 
CS5H
 
 
10.25
 
 
20.40
 
 
15.65
 
 
15.65
 
 
1.70
 
 
2.37
 
 
2.90
 
 
-0.01
 
 
0.391
 
 
 
 
CS6H
 
 
9.90
 
 
19.73
 
 
14.93
 
 
14.90
 
 
1.52
 
 
1.99
 
 
2.93
 
 
0.07
 
 
0.341
 
 
 
 
CS7H
 
 
14.15
 
 
16.54
 
 
15.34
 
 
15.33
 
 
0.38
 
 
0.50
 
 
3.14
 
 
0.03
 
 
0.352
 
 
 
 
DAII
 
 
89.37
 
 
137.94
 
 
112.51
 
 
112.47
 
 
8.09
 
 
10.72
 
 
2.97
 
 
0.14
 
 
0.0995
 
 
 
 
DAIM
 
 
68.98
 
 
116.02
 
 
93.35
 
 
93.27
 
 
7.81
 
 
10.70
 
 
2.91
 
 
-0.08
 
 
0.174
 
 
 
 
DAIP
 
 
59.53
 
 
109.05
 
 
86.84
 
 
86.92
 
 
8.01
 
 
10.92
 
 
2.97
 
 
-0.16
 
 
0.0254
 
 
 
 
DAIS
 
 
79.42
 
 
131.88
 
 
103.48
 
 
103.38
 
 
8.14
 
 
10.04
 
 
3.45
 
 
0.20
 
 
0.000116
 
 
 
 
DMAC
 
 
-0.06
 
 
0.00
 
 
-0.03
 
 
-0.03
 
 
0.01
 
 
0.01
 
 
3.63
 
 
0.06
 
 
0.000257
 
 
 
 
DICD
 
 
16.00
 
 
28.65
 
 
22.45
 
 
22.24
 
 
2.09
 
 
2.91
 
 
3.40
 
 
0.05
 
 
5.1e-07
 
 
 
 
DIIL
 
 
8.31
 
 
32.77
 
 
20.62
 
 
20.64
 
 
3.52
 
 
4.35
 
 
3.63
 
 
0.03
 
 
1.03e-05
 
 
 
 
DIMD
 
 
26.69
 
 
46.61
 
 
36.62
 
 
36.61
 
 
3.45
 
 
4.88
 
 
2.92
 
 
-0.02
 
 
0.0112
 
 
 
 
DIPD
 
 
20.85
 
 
40.71
 
 
30.42
 
 
30.29
 
 
3.13
 
 
4.11
 
 
3.15
 
 
-0.12
 
 
0.00148
 
 
 
 
DSIL
 
 
9.49
 
 
30.38
 
 
19.64
 
 
19.70
 
 
3.59
 
 
5.11
 
 
2.62
 
 
-0.03
 
 
0.0326
 
 
 
 
DDAP
 
 
-0.03
 
 
0.06
 
 
0.01
 
 
0.01
 
 
0.01
 
 
0.01
 
 
5.49
 
 
0.89
 
 
2.27e-23
 
 
 
 
SVTh
 
 
62.06
 
 
99.59
 
 
79.54
 
 
79.29
 
 
5.92
 
 
7.95
 
 
3.03
 
 
0.16
 
 
0.124
 
 
 
 
SVTh*
 
 
67.84
 
 
97.61
 
 
81.68
 
 
81.50
 
 
5.24
 
 
6.73
 
 
2.94
 
 
0.21
 
 
0.000456
 
 
 
 
SVTv
 
 
55.58
 
 
109.07
 
 
81.35
 
 
81.12
 
 
8.51
 
 
11.51
 
 
3.08
 
 
0.14
 
 
0.159
 
 
 
 
SVTv*
 
 
54.27
 
 
107.98
 
 
80.27
 
 
80.28
 
 
8.83
 
 
12.71
 
 
2.74
 
 
0.13
 
 
0.0115
 
 
 
 
GAPD
 
 
0.00
 
 
0.04
 
 
0.01
 
 
0.01
 
 
0.01
 
 
0.01
 
 
5.22
 
 
1.46
 
 
1.93e-30
 
 
 
 
GRPD
 
 
0.00
 
 
0.02
 
 
0.01
 
 
0.01
 
 
0.00
 
 
0.00
 
 
7.48
 
 
1.75
 
 
1.01e-31
 
 
 
 
HACP
 
 
-1.60
 
 
65.56
 
 
32.51
 
 
33.05
 
 
10.53
 
 
12.82
 
 
3.26
 
 
-0.31
 
 
1.87e-06
 
 
 
 
HARC
 
 
1.86
 
 
77.36
 
 
38.26
 
 
39.49
 
 
13.85
 
 
19.00
 
 
2.72
 
 
-0.22
 
 
1.31e-05
 
 
 
 
HARF
 
 
-13.57
 
 
92.17
 
 
21.75
 
 
18.34
 
 
16.06
 
 
19.28
 
 
4.65
 
 
1.22
 
 
5.24e-26
 
 
 
 
HDMP
 
 
-90.36
 
 
132.83
 
 
13.98
 
 
6.93
 
 
23.27
 
 
13.94
 
 
9.05
 
 
1.80
 
 
8.27e-38
 
 
 
 
HCCC
 
 
-0.18
 
 
0.08
 
 
-0.06
 
 
-0.06
 
 
0.04
 
 
0.05
 
 
3.59
 
 
0.09
 
 
0.000471
 
 
 
 
HM2C
 
 
-0.07
 
 
0.01
 
 
-0.04
 
 
-0.04
 
 
0.01
 
 
0.02
 
 
3.03
 
 
0.16
 
 
0.0539
 
 
 
 
HMAC
 
 
-0.09
 
 
0.14
 
 
0.05
 
 
0.05
 
 
0.04
 
 
0.04
 
 
4.10
 
 
-0.84
 
 
3.13e-18
 
 
 
 
HMDC
 
 
-0.09
 
 
0.01
 
 
-0.03
 
 
-0.03
 
 
0.01
 
 
0.01
 
 
3.99
 
 
0.20
 
 
2.45e-07
 
 
 
 
HP2C
 
 
-0.10
 
 
0.02
 
 
-0.04
 
 
-0.04
 
 
0.02
 
 
0.03
 
 
2.83
 
 
0.04
 
 
0.717
 
 
 
 
HACL
 
 
1.87
 
 
15.73
 
 
8.10
 
 
8.10
 
 
2.75
 
 
4.39
 
 
2.24
 
 
0.12
 
 
3.92e-11
 
 
 
 
HAML
 
 
23.29
 
 
46.64
 
 
35.92
 
 
36.03
 
 
3.87
 
 
5.47
 
 
2.68
 
 
-0.10
 
 
0.0263
 
 
 
 
HAPL
 
 
9.14
 
 
29.03
 
 
19.00
 
 
19.02
 
 
3.41
 
 
5.05
 
 
2.54
 
 
-0.09
 
 
0.000231
 
 
 
 
HBPL
 
 
77.37
 
 
112.01
 
 
93.15
 
 
92.85
 
 
5.67
 
 
7.93
 
 
2.91
 
 
0.29
 
 
2.54e-05
 
 
 
 
HCTH
 
 
-0.80
 
 
12.80
 
 
5.68
 
 
5.55
 
 
2.32
 
 
2.99
 
 
2.92
 
 
0.26
 
 
7.18e-05
 
 
 
 
HHPL
 
 
32.26
 
 
62.02
 
 
45.85
 
 
45.64
 
 
4.95
 
 
7.08
 
 
2.70
 
 
0.18
 
 
0.000245
 
 
 
 
HICH
 
 
-3.14
 
 
17.60
 
 
5.64
 
 
5.38
 
 
3.37
 
 
4.46
 
 
2.97
 
 
0.43
 
 
2.48e-09
 
 
 
 
HICL
 
 
7.43
 
 
23.01
 
 
15.35
 
 
15.31
 
 
2.49
 
 
3.33
 
 
3.00
 
 
0.15
 
 
0.0225
 
 
 
 
HICL*
 
 
5.46
 
 
17.70
 
 
11.60
 
 
11.46
 
 
1.86
 
 
2.38
 
 
3.12
 
 
0.17
 
 
0.00824
 
 
 
 
HIMH
 
 
2.97
 
 
19.98
 
 
11.18
 
 
11.08
 
 
2.62
 
 
3.24
 
 
3.32
 
 
0.26
 
 
0.000101
 
 
 
 
HIML
 
 
31.23
 
 
51.01
 
 
41.58
 
 
41.72
 
 
3.25
 
 
4.24
 
 
3.05
 
 
-0.11
 
 
0.0757
 
 
 
 
HIML*
 
 
29.65
 
 
47.41
 
 
38.48
 
 
38.54
 
 
2.87
 
 
3.66
 
 
3.12
 
 
-0.01
 
 
0.402
 
 
 
 
HIPH
 
 
4.01
 
 
22.71
 
 
12.83
 
 
12.83
 
 
2.89
 
 
3.69
 
 
2.99
 
 
0.11
 
 
0.215
 
 
 
 
HIPL
 
 
16.01
 
 
33.86
 
 
25.13
 
 
25.21
 
 
2.73
 
 
3.41
 
 
3.16
 
 
-0.13
 
 
0.109
 
 
 
 
HIPL*
 
 
13.77
 
 
29.84
 
 
21.97
 
 
21.94
 
 
2.49
 
 
3.20
 
 
3.23
 
 
-0.09
 
 
0.107
 
 
 
 
HMTC
 
 
-0.06
 
 
0.02
 
 
-0.01
 
 
-0.01
 
 
0.01
 
 
0.01
 
 
6.04
 
 
-0.83
 
 
8.73e-20
 
 
 
 
HMTH
 
 
2.82
 
 
21.40
 
 
12.20
 
 
12.09
 
 
2.74
 
 
3.51
 
 
3.35
 
 
0.20
 
 
0.000745
 
 
 
 
HNAP
 
 
4.47
 
 
24.43
 
 
12.95
 
 
13.04
 
 
3.25
 
 
4.26
 
 
2.86
 
 
0.16
 
 
0.0058
 
 
 
 
HNSL
 
 
43.58
 
 
71.60
 
 
57.47
 
 
57.23
 
 
4.69
 
 
6.25
 
 
2.99
 
 
0.16
 
 
0.0278
 
 
 
 
HPTH
 
 
4.06
 
 
18.98
 
 
11.61
 
 
11.47
 
 
2.55
 
 
3.29
 
 
3.07
 
 
0.14
 
 
0.00639
 
 
 
 
HCCP
 
 
-0.01
 
 
0.07
 
 
0.02
 
 
0.02
 
 
0.01
 
 
0.02
 
 
3.45
 
 
0.85
 
 
1.28e-20
 
 
 
 
HCMP
 
 
-0.02
 
 
0.06
 
 
0.01
 
 
0.01
 
 
0.01
 
 
0.01
 
 
6.76
 
 
1.59
 
 
6.04e-31
 
 
 
 
HCPP
 
 
-0.04
 
 
0.07
 
 
0.01
 
 
0.01
 
 
0.02
 
 
0.02
 
 
5.13
 
 
0.22
 
 
7.81e-22
 
 
 
 
HMSP
 
 
-0.01
 
 
0.04
 
 
0.01
 
 
0.01
 
 
0.01
 
 
0.01
 
 
6.79
 
 
1.33
 
 
3.41e-27
 
 
 
 
HBC2
 
 
20.84
 
 
54.21
 
 
36.70
 
 
35.60
 
 
5.85
 
 
8.24
 
 
2.82
 
 
0.55
 
 
1.69e-14
 
 
 
 
HBC3
 
 
19.89
 
 
47.09
 
 
32.09
 
 
31.69
 
 
4.51
 
 
5.77
 
 
3.26
 
 
0.42
 
 
7.93e-09
 
 
 
 
HBC4
 
 
20.62
 
 
55.51
 
 
36.03
 
 
35.75
 
 
5.57
 
 
7.44
 
 
2.97
 
 
0.27
 
 
0.000178
 
 
 
 
HBNP
 
 
43.75
 
 
88.32
 
 
62.88
 
 
61.76
 
 
7.96
 
 
11.29
 
 
2.67
 
 
0.44
 
 
4.21e-12
 
 
 
 
HBPG
 
 
31.95
 
 
66.74
 
 
49.50
 
 
49.66
 
 
5.75
 
 
7.81
 
 
3.03
 
 
-0.15
 
 
0.0382
 
 
 
 
L2CT
 
 
7.38
 
 
61.62
 
 
34.18
 
 
34.32
 
 
8.84
 
 
11.23
 
 
3.10
 
 
-0.02
 
 
0.195
 
 
 
 
L2EA
 
 
1.71
 
 
35.11
 
 
16.07
 
 
14.90
 
 
5.59
 
 
7.84
 
 
3.14
 
 
0.77
 
 
5.78e-20
 
 
 
 
L2EP
 
 
21.63
 
 
74.15
 
 
47.03
 
 
46.50
 
 
8.40
 
 
10.68
 
 
3.26
 
 
0.29
 
 
2.84e-05
 
 
 
 
L3CT
 
 
-5.95
 
 
41.05
 
 
17.15
 
 
16.67
 
 
7.54
 
 
10.01
 
 
3.15
 
 
0.17
 
 
0.00612
 
 
 
 
L3EA
 
 
7.68
 
 
32.66
 
 
17.09
 
 
16.58
 
 
4.68
 
 
6.35
 
 
3.05
 
 
0.57
 
 
1.48e-13
 
 
 
 
L3EP
 
 
15.68
 
 
59.84
 
 
34.98
 
 
34.50
 
 
6.78
 
 
8.57
 
 
3.42
 
 
0.39
 
 
1.73e-07
 
 
 
 
L4CT
 
 
-4.42
 
 
20.20
 
 
8.63
 
 
8.54
 
 
3.57
 
 
4.65
 
 
3.24
 
 
0.21
 
 
0.000185
 
 
 
 
L4EA
 
 
9.85
 
 
49.11
 
 
29.27
 
 
29.30
 
 
6.95
 
 
9.51
 
 
2.69
 
 
0.11
 
 
0.0101
 
 
 
 
L4EP
 
 
12.81
 
 
46.66
 
 
29.79
 
 
29.67
 
 
5.01
 
 
6.29
 
 
3.37
 
 
0.16
 
 
0.00496
 
 
 
 
L5CT
 
 
0.02
 
 
35.37
 
 
16.71
 
 
16.36
 
 
5.90
 
 
8.19
 
 
2.87
 
 
0.29
 
 
1.92e-05
 
 
 
 
L5EA
 
 
20.86
 
 
63.70
 
 
42.13
 
 
42.19
 
 
7.30
 
 
10.09
 
 
2.67
 
 
-0.04
 
 
0.103
 
 
 
 
L5EP
 
 
16.79
 
 
50.19
 
 
32.78
 
 
32.59
 
 
5.00
 
 
6.60
 
 
3.17
 
 
0.16
 
 
0.055
 
 
 
 
LACT
 
 
37.63
 
 
96.05
 
 
66.01
 
 
65.98
 
 
9.41
 
 
12.35
 
 
2.89
 
 
0.01
 
 
0.353
 
 
 
 
LAEA
 
 
17.49
 
 
67.01
 
 
42.55
 
 
41.67
 
 
8.38
 
 
11.04
 
 
2.97
 
 
0.34
 
 
2.97e-08
 
 
 
 
LAEP
 
 
48.38
 
 
105.40
 
 
75.03
 
 
74.78
 
 
8.99
 
 
11.77
 
 
3.13
 
 
0.21
 
 
0.00855
 
 
 
 
LEPL
 
 
9.70
 
 
57.44
 
 
33.83
 
 
33.89
 
 
6.90
 
 
8.64
 
 
3.31
 
 
-0.06
 
 
0.0492
 
 
 
 
LHCT
 
 
15.86
 
 
45.96
 
 
30.43
 
 
30.22
 
 
4.87
 
 
6.38
 
 
2.96
 
 
0.21
 
 
0.00265
 
 
 
 
LHEA
 
 
5.48
 
 
37.50
 
 
21.98
 
 
21.87
 
 
5.00
 
 
6.34
 
 
3.35
 
 
0.22
 
 
0.000139
 
 
 
 
LHEP
 
 
3.16
 
 
37.01
 
 
21.41
 
 
21.32
 
 
4.96
 
 
6.35
 
 
3.17
 
 
0.01
 
 
0.468
 
 
 
 
LNCT
 
 
54.27
 
 
107.98
 
 
80.27
 
 
80.28
 
 
8.83
 
 
12.71
 
 
2.74
 
 
0.13
 
 
0.0115
 
 
 
 
LNEA
 
 
33.97
 
 
76.03
 
 
54.56
 
 
54.05
 
 
6.86
 
 
9.12
 
 
2.86
 
 
0.17
 
 
0.00243
 
 
 
 
LNEP
 
 
55.58
 
 
109.07
 
 
81.35
 
 
81.12
 
 
8.51
 
 
11.51
 
 
3.08
 
 
0.14
 
 
0.159
 
 
 
 
MCGM
 
 
95.77
 
 
134.21
 
 
114.86
 
 
114.85
 
 
6.14
 
 
8.23
 
 
2.90
 
 
0.02
 
 
0.745
 
 
 
 
MCGP
 
 
90.10
 
 
127.82
 
 
108.42
 
 
108.50
 
 
6.00
 
 
7.91
 
 
2.90
 
 
0.07
 
 
0.522
 
 
 
 
MCGD
 
 
34.33
 
 
64.34
 
 
49.09
 
 
48.77
 
 
4.97
 
 
6.75
 
 
2.85
 
 
0.27
 
 
5.7e-05
 
 
 
 
MCGL
 
 
35.24
 
 
66.87
 
 
50.58
 
 
50.16
 
 
5.48
 
 
7.71
 
 
2.77
 
 
0.25
 
 
4.67e-05
 
 
 
 
MCGR
 
 
36.63
 
 
67.21
 
 
51.31
 
 
51.13
 
 
5.04
 
 
6.78
 
 
2.87
 
 
0.20
 
 
0.00192
 
 
 
 
MCMD
 
 
82.40
 
 
122.09
 
 
102.02
 
 
101.59
 
 
6.66
 
 
8.86
 
 
2.91
 
 
0.16
 
 
0.0113
 
 
 
 
MCPD
 
 
83.81
 
 
124.11
 
 
101.36
 
 
100.91
 
 
6.56
 
 
8.82
 
 
2.94
 
 
0.22
 
 
0.000551
 
 
 
 
MGMD
 
 
55.32
 
 
88.68
 
 
71.52
 
 
71.34
 
 
5.44
 
 
6.97
 
 
3.13
 
 
0.12
 
 
0.118
 
 
 
 
MGPD
 
 
57.92
 
 
91.59
 
 
74.87
 
 
74.72
 
 
5.65
 
 
7.33
 
 
3.04
 
 
0.16
 
 
0.0165
 
 
 
 
MICD
 
 
95.53
 
 
139.23
 
 
115.55
 
 
115.61
 
 
7.66
 
 
10.79
 
 
2.76
 
 
0.06
 
 
0.0373
 
 
 
 
MIGD
 
 
70.09
 
 
108.07
 
 
89.26
 
 
89.33
 
 
6.89
 
 
9.10
 
 
2.84
 
 
-0.09
 
 
0.00288
 
 
 
 
MMPG
 
 
4.12
 
 
15.23
 
 
9.22
 
 
9.17
 
 
1.86
 
 
2.57
 
 
3.00
 
 
0.22
 
 
0.000983
 
 
 
 
MPGL
 
 
70.35
 
 
106.77
 
 
87.66
 
 
87.39
 
 
5.74
 
 
7.53
 
 
3.09
 
 
0.19
 
 
0.007
 
 
 
 
MPGR
 
 
70.50
 
 
105.76
 
 
86.87
 
 
86.65
 
 
5.83
 
 
7.75
 
 
3.07
 
 
0.23
 
 
0.00189
 
 
 
 
OCHL
 
 
-0.47
 
 
2.29
 
 
0.74
 
 
0.73
 
 
0.43
 
 
0.59
 
 
3.00
 
 
0.21
 
 
0.00359
 
 
 
 
OCHW
 
 
-0.17
 
 
0.79
 
 
0.25
 
 
0.23
 
 
0.16
 
 
0.21
 
 
2.99
 
 
0.47
 
 
1.13e-10
 
 
 
 
OCLW
 
 
0.70
 
 
7.79
 
 
3.15
 
 
2.84
 
 
1.25
 
 
1.78
 
 
3.37
 
 
0.81
 
 
1.06e-20
 
 
 
 
OCMR
 
 
0.44
 
 
0.77
 
 
0.61
 
 
0.61
 
 
0.05
 
 
0.07
 
 
3.21
 
 
0.09
 
 
0.0287
 
 
 
 
OCPR
 
 
0.55
 
 
0.93
 
 
0.74
 
 
0.74
 
 
0.06
 
 
0.08
 
 
3.20
 
 
0.23
 
 
9.18e-05
 
 
 
 
OMHL
 
 
0.07
 
 
0.60
 
 
0.31
 
 
0.31
 
 
0.08
 
 
0.10
 
 
3.38
 
 
0.45
 
 
1.25e-08
 
 
 
 
OMHW
 
 
0.07
 
 
0.55
 
 
0.30
 
 
0.30
 
 
0.07
 
 
0.09
 
 
3.44
 
 
0.34
 
 
2.28e-07
 
 
 
 
OMLW
 
 
0.62
 
 
1.52
 
 
1.03
 
 
1.02
 
 
0.15
 
 
0.21
 
 
2.85
 
 
0.25
 
 
0.000222
 
 
 
 
OPHL
 
 
0.11
 
 
1.34
 
 
0.69
 
 
0.68
 
 
0.19
 
 
0.24
 
 
3.30
 
 
0.25
 
 
0.000677
 
 
 
 
OPHW
 
 
0.09
 
 
0.79
 
 
0.42
 
 
0.42
 
 
0.11
 
 
0.14
 
 
3.08
 
 
0.27
 
 
0.000101
 
 
 
 
OPLW
 
 
0.79
 
 
2.74
 
 
1.64
 
 
1.62
 
 
0.33
 
 
0.47
 
 
2.96
 
 
0.41
 
 
8.02e-09
 
 
 
 
OPMR
 
 
0.62
 
 
1.05
 
 
0.83
 
 
0.83
 
 
0.07
 
 
0.09
 
 
2.97
 
 
0.04
 
 
0.726
 
 
 
 
PPW2
 
 
-0.03
 
 
0.04
 
 
0.00
 
 
0.00
 
 
0.01
 
 
0.00
 
 
6.35
 
 
0.25
 
 
4.14e-31
 
 
 
 
PPW3
 
 
-0.02
 
 
0.02
 
 
0.00
 
 
0.00
 
 
0.01
 
 
0.00
 
 
5.38
 
 
0.53
 
 
1.75e-25
 
 
 
 
PPW4
 
 
-0.01
 
 
0.02
 
 
0.00
 
 
0.00
 
 
0.00
 
 
0.00
 
 
5.23
 
 
0.38
 
 
2.03e-22
 
 
 
 
PPW5
 
 
-0.01
 
 
0.01
 
 
0.00
 
 
0.00
 
 
0.00
 
 
0.00
 
 
4.34
 
 
0.30
 
 
3.23e-12
 
 
 
 
PPW6
 
 
0.00
 
 
0.01
 
 
0.00
 
 
0.00
 
 
0.00
 
 
0.00
 
 
3.91
 
 
0.46
 
 
1.04e-10
 
 
 
 
PPW7
 
 
0.00
 
 
0.00
 
 
0.00
 
 
0.00
 
 
0.00
 
 
0.00
 
 
2.62
 
 
-0.07
 
 
0.0102
 
 
 
 
PPWC
 
 
-0.01
 
 
0.02
 
 
0.00
 
 
0.00
 
 
0.00
 
 
0.00
 
 
6.41
 
 
0.61
 
 
1.27e-29
 
 
 
 
PNNP
 
 
11.22
 
 
36.93
 
 
24.60
 
 
24.62
 
 
4.05
 
 
5.62
 
 
3.03
 
 
0.02
 
 
0.191
 
 
 
 
PWTP
 
 
0.00
 
 
0.00
 
 
0.00
 
 
0.00
 
 
0.00
 
 
0.00
 
 
3.31
 
 
0.29
 
 
1.59e-06
 
 
 
 
AANP
 
 
-1.15
 
 
11.57
 
 
3.91
 
 
3.68
 
 
2.13
 
 
2.96
 
 
3.00
 
 
0.53
 
 
9.83e-13
 
 
 
 
AASN
 
 
71.23
 
 
101.59
 
 
86.80
 
 
86.77
 
 
4.75
 
 
6.42
 
 
3.07
 
 
0.13
 
 
0.218
 
 
 
 
ABSN
 
 
111.02
 
 
148.71
 
 
128.20
 
 
127.95
 
 
5.80
 
 
7.91
 
 
2.94
 
 
0.14
 
 
0.0863
 
 
 
 
ACSN
 
 
91.73
 
 
139.57
 
 
114.48
 
 
114.47
 
 
7.29
 
 
9.88
 
 
3.14
 
 
0.12
 
 
0.0533
 
 
 
 
ALNS
 
 
-0.86
 
 
19.42
 
 
7.12
 
 
6.95
 
 
3.52
 
 
5.12
 
 
2.73
 
 
0.33
 
 
1.13e-08
 
 
 
 
ANNF
 
 
78.68
 
 
104.16
 
 
91.88
 
 
91.65
 
 
4.17
 
 
5.65
 
 
2.92
 
 
0.10
 
 
0.24
 
 
 
 
ANSF
 
 
81.23
 
 
108.37
 
 
94.85
 
 
94.87
 
 
4.57
 
 
6.16
 
 
2.93
 
 
-0.09
 
 
0.345
 
 
 
 
APNP
 
 
-2.60
 
 
12.02
 
 
4.60
 
 
4.36
 
 
2.40
 
 
3.39
 
 
2.71
 
 
0.40
 
 
9.93e-11
 
 
 
 
APNS
 
 
65.09
 
 
93.30
 
 
79.29
 
 
79.34
 
 
4.43
 
 
6.17
 
 
2.91
 
 
0.03
 
 
0.389
 
 
 
 
APSN
 
 
61.19
 
 
94.18
 
 
77.09
 
 
77.30
 
 
5.23
 
 
7.04
 
 
2.84
 
 
-0.16
 
 
0.00963
 
 
 
 
ASCG
 
 
95.77
 
 
134.21
 
 
114.86
 
 
114.85
 
 
6.14
 
 
8.23
 
 
2.90
 
 
0.02
 
 
0.745
 
 
 
 
ASCG*
 
 
136.84
 
 
179.51
 
 
162.53
 
 
162.89
 
 
7.43
 
 
10.55
 
 
2.65
 
 
-0.22
 
 
2e-05
 
 
 
 
ASNP
 
 
69.15
 
 
96.20
 
 
83.43
 
 
83.33
 
 
4.22
 
 
5.56
 
 
2.91
 
 
0.15
 
 
0.0495
 
 
 
 
SBAN
 
 
85.97
 
 
119.55
 
 
101.74
 
 
101.42
 
 
5.44
 
 
7.51
 
 
2.98
 
 
0.26
 
 
8.34e-05
 
 
 
 
SBAP
 
 
77.37
 
 
112.01
 
 
93.15
 
 
92.85
 
 
5.67
 
 
7.93
 
 
2.91
 
 
0.29
 
 
2.54e-05
 
 
 
 
SBAS
 
 
30.03
 
 
56.62
 
 
43.14
 
 
43.00
 
 
4.34
 
 
5.38
 
 
3.26
 
 
0.25
 
 
2.58e-05
 
 
 
 
SBNA
 
 
78.46
 
 
113.15
 
 
94.64
 
 
94.15
 
 
5.96
 
 
8.16
 
 
2.88
 
 
0.29
 
 
1.53e-05
 
 
 
 
SBNP
 
 
25.34
 
 
49.59
 
 
37.56
 
 
37.34
 
 
4.07
 
 
5.73
 
 
2.70
 
 
0.23
 
 
2.32e-05
 
 
 
 
SHBN
 
 
165.00
 
 
211.51
 
 
186.92
 
 
186.60
 
 
7.93
 
 
10.87
 
 
3.06
 
 
0.24
 
 
0.00109
 
 
 
 
SHSW
 
 
108.82
 
 
158.40
 
 
131.49
 
 
131.28
 
 
8.58
 
 
11.33
 
 
2.82
 
 
0.12
 
 
0.0476
 
 
 
 
SIOD
 
 
59.03
 
 
93.32
 
 
74.88
 
 
74.34
 
 
5.68
 
 
7.39
 
 
2.92
 
 
0.24
 
 
0.000165
 
 
 
 
SNAM
 
 
92.66
 
 
140.13
 
 
114.35
 
 
114.02
 
 
8.31
 
 
11.65
 
 
2.81
 
 
0.14
 
 
0.0362
 
 
 
 
SNAP
 
 
85.52
 
 
132.05
 
 
107.49
 
 
107.15
 
 
8.11
 
 
10.92
 
 
2.86
 
 
0.19
 
 
0.00582
 
 
 
 
SNNA
 
 
37.25
 
 
62.43
 
 
49.33
 
 
49.24
 
 
4.03
 
 
5.44
 
 
2.97
 
 
0.21
 
 
0.00543
 
 
 
 
SNNP
 
 
59.21
 
 
89.25
 
 
73.38
 
 
73.22
 
 
4.77
 
 
6.07
 
 
3.27
 
 
0.17
 
 
0.0015
 
 
 
 
SNOL
 
 
42.20
 
 
61.47
 
 
51.79
 
 
51.72
 
 
3.22
 
 
4.24
 
 
2.91
 
 
0.11
 
 
0.11
 
 
 
 
SNOR
 
 
41.11
 
 
60.71
 
 
51.51
 
 
51.52
 
 
3.20
 
 
4.28
 
 
3.00
 
 
-0.01
 
 
0.522
 
 
 
 
SSEG
 
 
15.25
 
 
41.30
 
 
26.77
 
 
26.56
 
 
3.63
 
 
4.63
 
 
3.59
 
 
0.34
 
 
1.86e-06
 
 
 
 
SSEN
 
 
57.71
 
 
85.91
 
 
69.56
 
 
69.07
 
 
3.99
 
 
5.14
 
 
3.41
 
 
0.45
 
 
1.99e-08
 
 
 
 
SSEN*
 
 
31.24
 
 
55.77
 
 
43.78
 
 
43.67
 
 
3.88
 
 
4.93
 
 
3.01
 
 
0.17
 
 
0.0139
 
 
 
 
SPUN
 
 
11.70
 
 
39.41
 
 
26.36
 
 
26.40
 
 
4.42
 
 
6.04
 
 
2.92
 
 
0.01
 
 
0.903
 
 
 
 
 
 
 Males only 
 
 
  Table 3.    Summary statistics for all
measures across males only. Please note that, for a normal distribution,
the expected skewness is 0.0 and kurtosis is 3.0. The  p -values
are those of the Shapiro-Wilk normality test. 
 
 
 
 
 
 
min
 
 
max
 
 
mean
 
 
median
 
 
sd
 
 
iqr
 
 
kurtosis
 
 
skewness
 
 
shapiro.p
 
 
 
 
 
 
CS2A
 
 
30.48
 
 
50.80
 
 
39.65
 
 
39.41
 
 
3.43
 
 
4.43
 
 
3.54
 
 
0.45
 
 
0.000398
 
 
 
 
CS2H
 
 
24.04
 
 
45.22
 
 
33.86
 
 
33.77
 
 
3.62
 
 
4.81
 
 
3.17
 
 
0.35
 
 
0.0066
 
 
 
 
CS3H
 
 
11.18
 
 
25.20
 
 
19.50
 
 
19.61
 
 
2.20
 
 
2.90
 
 
3.43
 
 
-0.25
 
 
0.0404
 
 
 
 
CS4H
 
 
11.36
 
 
24.10
 
 
18.27
 
 
18.29
 
 
2.26
 
 
2.73
 
 
3.10
 
 
-0.14
 
 
0.195
 
 
 
 
CS5H
 
 
10.25
 
 
20.40
 
 
15.94
 
 
16.11
 
 
1.74
 
 
2.20
 
 
3.23
 
 
-0.19
 
 
0.0377
 
 
 
 
CS6H
 
 
11.47
 
 
19.73
 
 
15.50
 
 
15.51
 
 
1.49
 
 
1.90
 
 
2.84
 
 
-0.08
 
 
0.483
 
 
 
 
CS7H
 
 
14.15
 
 
16.54
 
 
15.46
 
 
15.47
 
 
0.38
 
 
0.49
 
 
3.25
 
 
-0.14
 
 
0.409
 
 
 
 
DAII
 
 
89.37
 
 
137.94
 
 
112.00
 
 
111.92
 
 
8.09
 
 
10.66
 
 
3.05
 
 
0.14
 
 
0.743
 
 
 
 
DAIM
 
 
68.98
 
 
111.30
 
 
93.50
 
 
93.73
 
 
7.67
 
 
10.79
 
 
2.79
 
 
-0.22
 
 
0.0177
 
 
 
 
DAIP
 
 
59.53
 
 
104.16
 
 
87.09
 
 
87.47
 
 
7.86
 
 
11.06
 
 
2.98
 
 
-0.33
 
 
0.00266
 
 
 
 
DAIS
 
 
82.05
 
 
129.74
 
 
103.91
 
 
103.69
 
 
8.05
 
 
10.70
 
 
3.16
 
 
0.18
 
 
0.418
 
 
 
 
DMAC
 
 
-0.05
 
 
0.00
 
 
-0.03
 
 
-0.03
 
 
0.01
 
 
0.01
 
 
3.38
 
 
0.21
 
 
0.145
 
 
 
 
DICD
 
 
16.00
 
 
28.44
 
 
23.00
 
 
23.09
 
 
2.20
 
 
2.62
 
 
3.52
 
 
-0.25
 
 
0.000516
 
 
 
 
DIIL
 
 
8.31
 
 
31.16
 
 
21.05
 
 
21.05
 
 
3.71
 
 
4.24
 
 
3.43
 
 
-0.15
 
 
0.0462
 
 
 
 
DIMD
 
 
27.02
 
 
46.28
 
 
37.58
 
 
37.82
 
 
3.43
 
 
4.88
 
 
2.96
 
 
-0.13
 
 
0.155
 
 
 
 
DIPD
 
 
21.02
 
 
40.71
 
 
31.38
 
 
31.34
 
 
3.27
 
 
4.14
 
 
3.50
 
 
-0.29
 
 
0.00155
 
 
 
 
DSIL
 
 
9.49
 
 
30.38
 
 
20.46
 
 
20.64
 
 
3.80
 
 
5.57
 
 
2.57
 
 
-0.12
 
 
0.115
 
 
 
 
DDAP
 
 
-0.02
 
 
0.05
 
 
0.01
 
 
0.01
 
 
0.01
 
 
0.01
 
 
4.81
 
 
0.96
 
 
1.2e-13
 
 
 
 
SVTh
 
 
62.06
 
 
99.59
 
 
82.23
 
 
83.00
 
 
6.18
 
 
8.28
 
 
3.05
 
 
-0.28
 
 
0.0437
 
 
 
 
SVTh*
 
 
68.01
 
 
97.61
 
 
84.44
 
 
84.42
 
 
5.44
 
 
6.82
 
 
2.91
 
 
-0.28
 
 
0.0107
 
 
 
 
SVTv
 
 
55.58
 
 
109.07
 
 
86.49
 
 
87.19
 
 
8.57
 
 
9.77
 
 
3.75
 
 
-0.43
 
 
9.61e-05
 
 
 
 
SVTv*
 
 
54.27
 
 
107.98
 
 
86.33
 
 
87.53
 
 
8.53
 
 
9.44
 
 
4.07
 
 
-0.70
 
 
2.8e-08
 
 
 
 
GAPD
 
 
0.00
 
 
0.04
 
 
0.01
 
 
0.01
 
 
0.01
 
 
0.01
 
 
5.72
 
 
1.48
 
 
1.3e-17
 
 
 
 
GRPD
 
 
0.00
 
 
0.02
 
 
0.01
 
 
0.01
 
 
0.00
 
 
0.00
 
 
6.44
 
 
1.63
 
 
2.56e-19
 
 
 
 
HACP
 
 
0.80
 
 
65.56
 
 
34.23
 
 
35.06
 
 
10.20
 
 
12.46
 
 
3.27
 
 
-0.42
 
 
0.000402
 
 
 
 
HARC
 
 
2.38
 
 
77.36
 
 
35.21
 
 
35.55
 
 
13.50
 
 
18.86
 
 
2.79
 
 
0.01
 
 
0.241
 
 
 
 
HARF
 
 
-13.57
 
 
91.86
 
 
19.80
 
 
17.42
 
 
14.89
 
 
16.37
 
 
5.55
 
 
1.37
 
 
3.48e-16
 
 
 
 
HDMP
 
 
-80.93
 
 
115.68
 
 
15.14
 
 
8.23
 
 
21.91
 
 
16.06
 
 
7.65
 
 
1.55
 
 
4.91e-22
 
 
 
 
HCCC
 
 
-0.18
 
 
0.07
 
 
-0.06
 
 
-0.06
 
 
0.04
 
 
0.05
 
 
3.89
 
 
0.11
 
 
0.00513
 
 
 
 
HM2C
 
 
-0.07
 
 
0.00
 
 
-0.03
 
 
-0.03
 
 
0.01
 
 
0.02
 
 
3.34
 
 
0.18
 
 
0.0614
 
 
 
 
HMAC
 
 
-0.08
 
 
0.13
 
 
0.05
 
 
0.05
 
 
0.03
 
 
0.04
 
 
4.56
 
 
-0.86
 
 
1.56e-10
 
 
 
 
HMDC
 
 
-0.07
 
 
0.01
 
 
-0.03
 
 
-0.03
 
 
0.01
 
 
0.01
 
 
3.52
 
 
-0.08
 
 
0.264
 
 
 
 
HP2C
 
 
-0.09
 
 
0.01
 
 
-0.04
 
 
-0.04
 
 
0.02
 
 
0.03
 
 
2.79
 
 
-0.07
 
 
0.636
 
 
 
 
HACL
 
 
1.87
 
 
15.73
 
 
8.38
 
 
8.41
 
 
2.97
 
 
4.78
 
 
2.22
 
 
0.12
 
 
3.15e-05
 
 
 
 
HAML
 
 
27.02
 
 
46.64
 
 
36.70
 
 
36.97
 
 
3.81
 
 
5.57
 
 
2.49
 
 
-0.16
 
 
0.0166
 
 
 
 
HAPL
 
 
10.59
 
 
27.90
 
 
19.51
 
 
19.67
 
 
3.50
 
 
5.07
 
 
2.51
 
 
-0.14
 
 
0.0101
 
 
 
 
HBPL
 
 
80.23
 
 
112.01
 
 
96.61
 
 
96.90
 
 
5.70
 
 
7.86
 
 
2.85
 
 
-0.21
 
 
0.227
 
 
 
 
HCTH
 
 
-0.25
 
 
10.84
 
 
5.12
 
 
5.04
 
 
2.17
 
 
2.89
 
 
2.74
 
 
0.25
 
 
0.0206
 
 
 
 
HHPL
 
 
33.39
 
 
62.02
 
 
46.87
 
 
46.82
 
 
5.27
 
 
7.89
 
 
2.48
 
 
0.02
 
 
0.0373
 
 
 
 
HICH
 
 
-2.81
 
 
17.60
 
 
5.25
 
 
5.00
 
 
3.44
 
 
4.72
 
 
3.29
 
 
0.57
 
 
3.63e-06
 
 
 
 
HICL
 
 
7.91
 
 
22.73
 
 
15.87
 
 
15.85
 
 
2.56
 
 
3.54
 
 
2.76
 
 
-0.06
 
 
0.734
 
 
 
 
HICL*
 
 
6.02
 
 
17.70
 
 
12.11
 
 
12.08
 
 
1.94
 
 
2.78
 
 
3.06
 
 
0.14
 
 
0.038
 
 
 
 
HIMH
 
 
4.44
 
 
19.98
 
 
12.01
 
 
11.94
 
 
2.55
 
 
3.39
 
 
3.09
 
 
0.18
 
 
0.431
 
 
 
 
HIML
 
 
33.55
 
 
51.01
 
 
42.80
 
 
42.74
 
 
2.87
 
 
3.83
 
 
3.13
 
 
-0.03
 
 
0.409
 
 
 
 
HIML*
 
 
31.52
 
 
47.41
 
 
39.47
 
 
39.41
 
 
2.57
 
 
3.32
 
 
3.26
 
 
0.08
 
 
0.358
 
 
 
 
HIPH
 
 
4.01
 
 
22.71
 
 
13.22
 
 
13.37
 
 
3.05
 
 
4.25
 
 
2.87
 
 
-0.01
 
 
0.91
 
 
 
 
HIPL
 
 
16.01
 
 
33.86
 
 
25.97
 
 
26.02
 
 
2.66
 
 
3.60
 
 
3.19
 
 
-0.12
 
 
0.851
 
 
 
 
HIPL*
 
 
14.98
 
 
29.84
 
 
22.74
 
 
22.82
 
 
2.40
 
 
2.91
 
 
3.42
 
 
-0.07
 
 
0.262
 
 
 
 
HMTC
 
 
-0.06
 
 
0.02
 
 
-0.01
 
 
-0.01
 
 
0.01
 
 
0.01
 
 
5.67
 
 
-0.60
 
 
2.99e-08
 
 
 
 
HMTH
 
 
4.47
 
 
21.18
 
 
12.93
 
 
12.69
 
 
2.71
 
 
3.59
 
 
2.99
 
 
0.13
 
 
0.542
 
 
 
 
HNAP
 
 
5.10
 
 
24.43
 
 
13.47
 
 
13.44
 
 
3.38
 
 
4.51
 
 
2.90
 
 
0.19
 
 
0.115
 
 
 
 
HNSL
 
 
46.18
 
 
71.60
 
 
60.00
 
 
60.20
 
 
4.79
 
 
6.49
 
 
2.92
 
 
-0.23
 
 
0.0837
 
 
 
 
HPTH
 
 
4.06
 
 
18.98
 
 
11.51
 
 
11.42
 
 
2.55
 
 
3.30
 
 
3.22
 
 
0.14
 
 
0.127
 
 
 
 
HCCP
 
 
-0.01
 
 
0.07
 
 
0.02
 
 
0.02
 
 
0.01
 
 
0.02
 
 
3.59
 
 
0.83
 
 
1.05e-10
 
 
 
 
HCMP
 
 
-0.02
 
 
0.06
 
 
0.01
 
 
0.01
 
 
0.01
 
 
0.01
 
 
6.22
 
 
1.48
 
 
3.94e-18
 
 
 
 
HCPP
 
 
-0.04
 
 
0.07
 
 
0.01
 
 
0.01
 
 
0.02
 
 
0.02
 
 
4.75
 
 
0.22
 
 
8.69e-10
 
 
 
 
HMSP
 
 
-0.01
 
 
0.04
 
 
0.01
 
 
0.01
 
 
0.01
 
 
0.01
 
 
6.61
 
 
1.31
 
 
3.74e-16
 
 
 
 
HBC2
 
 
20.84
 
 
54.21
 
 
41.24
 
 
41.39
 
 
5.68
 
 
7.79
 
 
2.92
 
 
-0.21
 
 
0.14
 
 
 
 
HBC3
 
 
22.83
 
 
47.09
 
 
34.83
 
 
34.58
 
 
4.63
 
 
6.73
 
 
2.62
 
 
0.14
 
 
0.0534
 
 
 
 
HBC4
 
 
22.56
 
 
55.51
 
 
37.43
 
 
37.05
 
 
5.71
 
 
8.17
 
 
2.78
 
 
0.22
 
 
0.0879
 
 
 
 
HBNP
 
 
43.75
 
 
88.32
 
 
69.35
 
 
69.82
 
 
7.46
 
 
10.24
 
 
2.91
 
 
-0.30
 
 
0.0217
 
 
 
 
HBPG
 
 
31.95
 
 
66.74
 
 
51.45
 
 
51.65
 
 
5.87
 
 
7.49
 
 
3.41
 
 
-0.39
 
 
0.00308
 
 
 
 
L2CT
 
 
7.38
 
 
61.62
 
 
38.33
 
 
38.34
 
 
9.15
 
 
11.51
 
 
3.73
 
 
-0.56
 
 
5.43e-06
 
 
 
 
L2EA
 
 
1.71
 
 
35.11
 
 
17.84
 
 
17.53
 
 
5.90
 
 
8.21
 
 
2.74
 
 
0.36
 
 
0.000146
 
 
 
 
L2EP
 
 
25.31
 
 
74.15
 
 
50.10
 
 
50.21
 
 
9.22
 
 
12.62
 
 
2.91
 
 
0.07
 
 
0.388
 
 
 
 
L3CT
 
 
-5.30
 
 
41.05
 
 
19.84
 
 
20.10
 
 
8.10
 
 
10.33
 
 
3.12
 
 
-0.18
 
 
0.243
 
 
 
 
L3EA
 
 
8.12
 
 
32.66
 
 
17.68
 
 
17.28
 
 
4.71
 
 
6.50
 
 
2.80
 
 
0.43
 
 
4.09e-05
 
 
 
 
L3EP
 
 
17.38
 
 
57.18
 
 
36.69
 
 
36.29
 
 
7.77
 
 
10.47
 
 
2.73
 
 
0.21
 
 
0.045
 
 
 
 
L4CT
 
 
-4.42
 
 
20.15
 
 
8.39
 
 
8.31
 
 
3.93
 
 
5.38
 
 
2.98
 
 
0.12
 
 
0.469
 
 
 
 
L4EA
 
 
12.25
 
 
49.11
 
 
30.03
 
 
30.10
 
 
7.45
 
 
11.19
 
 
2.42
 
 
-0.02
 
 
0.0207
 
 
 
 
L4EP
 
 
15.27
 
 
46.66
 
 
30.41
 
 
30.28
 
 
5.83
 
 
8.42
 
 
2.69
 
 
0.15
 
 
0.196
 
 
 
 
L5CT
 
 
0.02
 
 
32.52
 
 
14.49
 
 
14.15
 
 
5.54
 
 
7.30
 
 
3.05
 
 
0.37
 
 
0.0038
 
 
 
 
L5EA
 
 
22.58
 
 
62.37
 
 
42.26
 
 
42.31
 
 
7.60
 
 
11.26
 
 
2.50
 
 
-0.09
 
 
0.0359
 
 
 
 
L5EP
 
 
16.79
 
 
50.19
 
 
33.31
 
 
33.50
 
 
5.28
 
 
6.74
 
 
3.21
 
 
0.03
 
 
0.796
 
 
 
 
LACT
 
 
39.26
 
 
96.05
 
 
71.72
 
 
72.19
 
 
9.21
 
 
11.84
 
 
3.80
 
 
-0.64
 
 
2e-07
 
 
 
 
LAEA
 
 
17.49
 
 
67.01
 
 
45.93
 
 
45.72
 
 
8.88
 
 
11.76
 
 
2.96
 
 
-0.02
 
 
0.0437
 
 
 
 
LAEP
 
 
51.26
 
 
105.40
 
 
79.83
 
 
79.68
 
 
9.19
 
 
11.73
 
 
3.21
 
 
-0.15
 
 
0.149
 
 
 
 
LEPL
 
 
13.42
 
 
57.44
 
 
35.27
 
 
35.84
 
 
7.76
 
 
9.74
 
 
2.89
 
 
-0.14
 
 
0.374
 
 
 
 
LHCT
 
 
19.30
 
 
45.96
 
 
32.86
 
 
32.77
 
 
4.81
 
 
6.26
 
 
2.98
 
 
-0.16
 
 
0.161
 
 
 
 
LHEA
 
 
9.63
 
 
37.50
 
 
24.82
 
 
24.70
 
 
5.13
 
 
7.05
 
 
2.96
 
 
-0.04
 
 
0.448
 
 
 
 
LHEP
 
 
4.58
 
 
37.01
 
 
21.89
 
 
21.85
 
 
5.07
 
 
6.58
 
 
3.16
 
 
-0.01
 
 
0.924
 
 
 
 
LNCT
 
 
54.27
 
 
107.98
 
 
86.33
 
 
87.53
 
 
8.53
 
 
9.44
 
 
4.07
 
 
-0.70
 
 
2.8e-08
 
 
 
 
LNEA
 
 
33.97
 
 
76.03
 
 
57.85
 
 
58.04
 
 
7.18
 
 
9.72
 
 
3.34
 
 
-0.36
 
 
0.00496
 
 
 
 
LNEP
 
 
55.58
 
 
109.07
 
 
86.49
 
 
87.19
 
 
8.57
 
 
9.77
 
 
3.75
 
 
-0.43
 
 
9.61e-05
 
 
 
 
MCGM
 
 
95.77
 
 
134.21
 
 
114.43
 
 
114.63
 
 
6.57
 
 
9.39
 
 
2.56
 
 
-0.04
 
 
0.133
 
 
 
 
MCGP
 
 
92.34
 
 
127.82
 
 
108.01
 
 
108.15
 
 
6.47
 
 
9.30
 
 
2.56
 
 
0.02
 
 
0.233
 
 
 
 
MCGD
 
 
39.17
 
 
64.34
 
 
52.75
 
 
52.37
 
 
4.51
 
 
6.55
 
 
2.63
 
 
0.09
 
 
0.148
 
 
 
 
MCGL
 
 
41.16
 
 
66.87
 
 
54.20
 
 
54.02
 
 
5.10
 
 
7.45
 
 
2.47
 
 
0.08
 
 
0.0629
 
 
 
 
MCGR
 
 
40.21
 
 
67.21
 
 
54.73
 
 
54.86
 
 
4.74
 
 
6.69
 
 
2.88
 
 
-0.04
 
 
0.27
 
 
 
 
MCMD
 
 
91.80
 
 
122.09
 
 
107.37
 
 
107.75
 
 
5.61
 
 
7.96
 
 
2.76
 
 
-0.03
 
 
0.382
 
 
 
 
MCPD
 
 
90.19
 
 
124.11
 
 
106.68
 
 
106.83
 
 
5.61
 
 
7.90
 
 
2.78
 
 
0.03
 
 
0.631
 
 
 
 
MGMD
 
 
55.32
 
 
88.68
 
 
74.70
 
 
74.53
 
 
5.13
 
 
6.75
 
 
3.07
 
 
0.03
 
 
0.628
 
 
 
 
MGPD
 
 
65.00
 
 
91.59
 
 
78.30
 
 
78.20
 
 
5.40
 
 
7.39
 
 
2.60
 
 
0.10
 
 
0.0981
 
 
 
 
MICD
 
 
101.05
 
 
139.23
 
 
119.86
 
 
120.12
 
 
7.25
 
 
9.96
 
 
2.66
 
 
-0.14
 
 
0.0307
 
 
 
 
MIGD
 
 
74.35
 
 
108.07
 
 
93.64
 
 
94.28
 
 
6.83
 
 
8.01
 
 
2.91
 
 
-0.59
 
 
1.85e-08
 
 
 
 
MMPG
 
 
4.93
 
 
15.17
 
 
9.58
 
 
9.49
 
 
1.87
 
 
2.57
 
 
3.03
 
 
0.22
 
 
0.0704
 
 
 
 
MPGL
 
 
70.35
 
 
106.77
 
 
91.68
 
 
91.54
 
 
5.38
 
 
7.15
 
 
3.30
 
 
-0.08
 
 
0.486
 
 
 
 
MPGR
 
 
70.50
 
 
105.76
 
 
91.03
 
 
90.82
 
 
5.43
 
 
7.71
 
 
3.19
 
 
-0.01
 
 
0.296
 
 
 
 
OCHL
 
 
-0.47
 
 
2.10
 
 
0.66
 
 
0.65
 
 
0.42
 
 
0.57
 
 
2.99
 
 
0.18
 
 
0.167
 
 
 
 
OCHW
 
 
-0.17
 
 
0.79
 
 
0.23
 
 
0.21
 
 
0.16
 
 
0.21
 
 
3.11
 
 
0.55
 
 
4.34e-06
 
 
 
 
OCLW
 
 
0.78
 
 
7.72
 
 
3.17
 
 
2.83
 
 
1.34
 
 
1.91
 
 
3.45
 
 
0.88
 
 
1.52e-12
 
 
 
 
OCMR
 
 
0.44
 
 
0.77
 
 
0.61
 
 
0.61
 
 
0.05
 
 
0.07
 
 
3.32
 
 
0.04
 
 
0.377
 
 
 
 
OCPR
 
 
0.55
 
 
0.92
 
 
0.73
 
 
0.73
 
 
0.06
 
 
0.08
 
 
3.29
 
 
0.27
 
 
0.0107
 
 
 
 
OMHL
 
 
0.12
 
 
0.58
 
 
0.33
 
 
0.32
 
 
0.08
 
 
0.10
 
 
3.25
 
 
0.32
 
 
0.0122
 
 
 
 
OMHW
 
 
0.11
 
 
0.55
 
 
0.32
 
 
0.32
 
 
0.07
 
 
0.09
 
 
3.23
 
 
0.32
 
 
0.0057
 
 
 
 
OMLW
 
 
0.62
 
 
1.52
 
 
1.04
 
 
1.03
 
 
0.15
 
 
0.21
 
 
2.93
 
 
0.27
 
 
0.0238
 
 
 
 
OPHL
 
 
0.11
 
 
1.27
 
 
0.69
 
 
0.69
 
 
0.19
 
 
0.27
 
 
2.92
 
 
0.03
 
 
0.952
 
 
 
 
OPHW
 
 
0.09
 
 
0.79
 
 
0.43
 
 
0.42
 
 
0.12
 
 
0.16
 
 
2.89
 
 
0.19
 
 
0.311
 
 
 
 
OPLW
 
 
0.79
 
 
2.72
 
 
1.65
 
 
1.61
 
 
0.34
 
 
0.49
 
 
2.82
 
 
0.37
 
 
0.00108
 
 
 
 
OPMR
 
 
0.65
 
 
1.02
 
 
0.84
 
 
0.83
 
 
0.07
 
 
0.09
 
 
2.83
 
 
0.00
 
 
0.621
 
 
 
 
PPW2
 
 
-0.03
 
 
0.04
 
 
0.00
 
 
0.00
 
 
0.01
 
 
0.00
 
 
6.84
 
 
-0.16
 
 
1.2e-18
 
 
 
 
PPW3
 
 
-0.02
 
 
0.02
 
 
0.00
 
 
0.00
 
 
0.01
 
 
0.00
 
 
4.79
 
 
0.46
 
 
1.89e-12
 
 
 
 
PPW4
 
 
-0.01
 
 
0.02
 
 
0.00
 
 
0.00
 
 
0.00
 
 
0.00
 
 
5.17
 
 
0.63
 
 
7.53e-12
 
 
 
 
PPW5
 
 
-0.01
 
 
0.01
 
 
0.00
 
 
0.00
 
 
0.00
 
 
0.00
 
 
3.66
 
 
0.36
 
 
0.000495
 
 
 
 
PPW6
 
 
0.00
 
 
0.01
 
 
0.00
 
 
0.00
 
 
0.00
 
 
0.00
 
 
4.27
 
 
0.46
 
 
3.12e-06
 
 
 
 
PPW7
 
 
0.00
 
 
0.00
 
 
0.00
 
 
0.00
 
 
0.00
 
 
0.00
 
 
2.71
 
 
0.11
 
 
0.326
 
 
 
 
PPWC
 
 
-0.01
 
 
0.02
 
 
0.00
 
 
0.00
 
 
0.00
 
 
0.00
 
 
5.74
 
 
0.68
 
 
3.33e-16
 
 
 
 
PNNP
 
 
12.73
 
 
36.93
 
 
25.08
 
 
25.07
 
 
4.42
 
 
6.30
 
 
2.76
 
 
-0.09
 
 
0.222
 
 
 
 
PWTP
 
 
0.00
 
 
0.00
 
 
0.00
 
 
0.00
 
 
0.00
 
 
0.00
 
 
2.95
 
 
-0.10
 
 
0.843
 
 
 
 
AANP
 
 
-0.69
 
 
11.57
 
 
4.17
 
 
3.96
 
 
2.33
 
 
3.51
 
 
2.70
 
 
0.45
 
 
5.06e-06
 
 
 
 
AASN
 
 
71.23
 
 
101.59
 
 
87.30
 
 
87.41
 
 
4.85
 
 
6.44
 
 
3.24
 
 
0.00
 
 
0.629
 
 
 
 
ABSN
 
 
111.06
 
 
144.54
 
 
127.53
 
 
127.19
 
 
5.67
 
 
7.91
 
 
2.93
 
 
0.22
 
 
0.226
 
 
 
 
ACSN
 
 
92.72
 
 
136.56
 
 
114.37
 
 
114.28
 
 
7.41
 
 
9.69
 
 
3.03
 
 
0.13
 
 
0.335
 
 
 
 
ALNS
 
 
0.10
 
 
19.42
 
 
6.72
 
 
6.43
 
 
3.52
 
 
5.07
 
 
2.92
 
 
0.44
 
 
1.31e-05
 
 
 
 
ANNF
 
 
78.68
 
 
103.96
 
 
92.48
 
 
92.32
 
 
4.59
 
 
6.02
 
 
2.88
 
 
-0.03
 
 
0.722
 
 
 
 
ANSF
 
 
81.23
 
 
108.37
 
 
93.69
 
 
93.96
 
 
4.56
 
 
6.15
 
 
3.08
 
 
0.00
 
 
0.614
 
 
 
 
APNP
 
 
-1.06
 
 
11.33
 
 
4.38
 
 
3.99
 
 
2.47
 
 
3.42
 
 
2.65
 
 
0.52
 
 
8.47e-08
 
 
 
 
APNS
 
 
65.09
 
 
93.30
 
 
80.03
 
 
80.29
 
 
4.43
 
 
6.05
 
 
3.13
 
 
-0.13
 
 
0.699
 
 
 
 
APSN
 
 
61.19
 
 
89.25
 
 
77.46
 
 
77.78
 
 
5.18
 
 
7.32
 
 
2.77
 
 
-0.28
 
 
0.0147
 
 
 
 
ASCG
 
 
95.77
 
 
134.21
 
 
114.43
 
 
114.63
 
 
6.57
 
 
9.39
 
 
2.56
 
 
-0.04
 
 
0.133
 
 
 
 
ASCG*
 
 
136.84
 
 
179.28
 
 
162.31
 
 
162.61
 
 
7.28
 
 
10.14
 
 
2.88
 
 
-0.20
 
 
0.214
 
 
 
 
ASNP
 
 
69.15
 
 
94.92
 
 
83.61
 
 
83.73
 
 
4.18
 
 
5.45
 
 
3.07
 
 
-0.04
 
 
0.843
 
 
 
 
SBAN
 
 
93.83
 
 
119.55
 
 
106.02
 
 
106.14
 
 
4.74
 
 
6.52
 
 
2.88
 
 
0.04
 
 
0.598
 
 
 
 
SBAP
 
 
80.23
 
 
112.01
 
 
96.61
 
 
96.90
 
 
5.70
 
 
7.86
 
 
2.85
 
 
-0.21
 
 
0.227
 
 
 
 
SBAS
 
 
33.15
 
 
56.62
 
 
45.63
 
 
45.67
 
 
4.38
 
 
5.95
 
 
2.89
 
 
0.00
 
 
0.453
 
 
 
 
SBNA
 
 
79.41
 
 
113.15
 
 
98.61
 
 
99.09
 
 
5.98
 
 
7.76
 
 
3.13
 
 
-0.36
 
 
0.00509
 
 
 
 
SBNP
 
 
25.34
 
 
49.59
 
 
39.09
 
 
39.23
 
 
4.10
 
 
6.14
 
 
2.56
 
 
0.03
 
 
0.0363
 
 
 
 
SHBN
 
 
175.37
 
 
211.51
 
 
192.43
 
 
192.18
 
 
6.89
 
 
9.46
 
 
2.94
 
 
0.30
 
 
0.0114
 
 
 
 
SHSW
 
 
115.00
 
 
158.40
 
 
137.06
 
 
137.14
 
 
7.99
 
 
11.23
 
 
2.63
 
 
-0.04
 
 
0.377
 
 
 
 
SIOD
 
 
61.07
 
 
93.32
 
 
77.27
 
 
76.98
 
 
5.65
 
 
7.94
 
 
2.76
 
 
0.16
 
 
0.0733
 
 
 
 
SNAM
 
 
98.78
 
 
140.13
 
 
119.89
 
 
119.79
 
 
7.48
 
 
9.94
 
 
2.83
 
 
-0.02
 
 
0.697
 
 
 
 
SNAP
 
 
93.13
 
 
132.05
 
 
112.86
 
 
112.37
 
 
7.49
 
 
9.91
 
 
2.75
 
 
0.03
 
 
0.267
 
 
 
 
SNNA
 
 
40.51
 
 
62.43
 
 
51.77
 
 
51.73
 
 
3.86
 
 
5.09
 
 
3.09
 
 
-0.03
 
 
0.66
 
 
 
 
SNNP
 
 
62.39
 
 
89.07
 
 
76.78
 
 
76.85
 
 
4.22
 
 
5.84
 
 
3.25
 
 
0.01
 
 
0.405
 
 
 
 
SNOL
 
 
45.67
 
 
61.47
 
 
53.56
 
 
53.46
 
 
2.95
 
 
3.76
 
 
2.92
 
 
0.06
 
 
0.283
 
 
 
 
SNOR
 
 
44.88
 
 
60.71
 
 
53.34
 
 
53.34
 
 
2.87
 
 
3.74
 
 
2.92
 
 
-0.04
 
 
0.798
 
 
 
 
SSEG
 
 
19.33
 
 
41.30
 
 
28.46
 
 
28.41
 
 
3.60
 
 
4.57
 
 
3.25
 
 
0.37
 
 
0.00289
 
 
 
 
SSEN
 
 
61.55
 
 
85.91
 
 
72.20
 
 
72.01
 
 
3.93
 
 
4.89
 
 
3.27
 
 
0.15
 
 
0.2
 
 
 
 
SSEN*
 
 
35.51
 
 
55.77
 
 
46.48
 
 
46.32
 
 
3.54
 
 
4.59
 
 
3.14
 
 
-0.15
 
 
0.091
 
 
 
 
SPUN
 
 
15.00
 
 
39.41
 
 
27.05
 
 
27.04
 
 
4.60
 
 
6.41
 
 
2.80
 
 
0.12
 
 
0.484
 
 
 
 
 
 
 Females only 
 
 
  Table 4.    Summary statistics for all
measures across females only. Please note that, for a normal
distribution, the expected skewness is 0.0 and kurtosis is 3.0. The
 p -values are those of the Shapiro-Wilk normality test. 
 
 
 
 
 
 
min
 
 
max
 
 
mean
 
 
median
 
 
sd
 
 
iqr
 
 
kurtosis
 
 
skewness
 
 
shapiro.p
 
 
 
 
 
 
CS2A
 
 
28.18
 
 
50.31
 
 
36.51
 
 
36.36
 
 
3.15
 
 
4.30
 
 
3.58
 
 
0.52
 
 
2.07e-07
 
 
 
 
CS2H
 
 
22.20
 
 
45.29
 
 
31.45
 
 
31.27
 
 
3.31
 
 
4.30
 
 
3.48
 
 
0.27
 
 
0.00145
 
 
 
 
CS3H
 
 
11.58
 
 
25.08
 
 
17.41
 
 
17.26
 
 
1.94
 
 
2.42
 
 
3.79
 
 
0.38
 
 
5.01e-07
 
 
 
 
CS4H
 
 
10.05
 
 
23.62
 
 
16.37
 
 
16.25
 
 
1.84
 
 
2.13
 
 
3.79
 
 
0.10
 
 
3.6e-05
 
 
 
 
CS5H
 
 
10.67
 
 
20.22
 
 
15.49
 
 
15.42
 
 
1.66
 
 
2.30
 
 
2.78
 
 
0.07
 
 
0.124
 
 
 
 
CS6H
 
 
9.90
 
 
19.34
 
 
14.62
 
 
14.60
 
 
1.44
 
 
1.88
 
 
3.13
 
 
0.12
 
 
0.314
 
 
 
 
CS7H
 
 
14.18
 
 
16.49
 
 
15.27
 
 
15.26
 
 
0.36
 
 
0.47
 
 
3.21
 
 
0.05
 
 
0.593
 
 
 
 
DAII
 
 
89.92
 
 
136.86
 
 
112.79
 
 
112.60
 
 
8.07
 
 
10.74
 
 
2.93
 
 
0.14
 
 
0.221
 
 
 
 
DAIM
 
 
69.49
 
 
116.02
 
 
93.27
 
 
92.94
 
 
7.88
 
 
10.55
 
 
2.98
 
 
0.00
 
 
0.713
 
 
 
 
DAIP
 
 
60.64
 
 
109.05
 
 
86.70
 
 
86.58
 
 
8.09
 
 
10.70
 
 
2.98
 
 
-0.08
 
 
0.599
 
 
 
 
DAIS
 
 
79.42
 
 
131.88
 
 
103.24
 
 
103.15
 
 
8.19
 
 
9.74
 
 
3.60
 
 
0.22
 
 
9.12e-05
 
 
 
 
DMAC
 
 
-0.06
 
 
0.00
 
 
-0.03
 
 
-0.03
 
 
0.01
 
 
0.01
 
 
3.71
 
 
-0.01
 
 
0.00134
 
 
 
 
DICD
 
 
16.12
 
 
28.65
 
 
22.15
 
 
22.05
 
 
1.96
 
 
2.13
 
 
3.55
 
 
0.15
 
 
5.58e-06
 
 
 
 
DIIL
 
 
9.17
 
 
32.77
 
 
20.39
 
 
20.39
 
 
3.39
 
 
4.14
 
 
3.83
 
 
0.11
 
 
3.01e-05
 
 
 
 
DIMD
 
 
26.69
 
 
46.61
 
 
36.10
 
 
36.07
 
 
3.35
 
 
4.05
 
 
2.98
 
 
0.01
 
 
0.0469
 
 
 
 
DIPD
 
 
20.85
 
 
37.11
 
 
29.90
 
 
30.03
 
 
2.92
 
 
3.98
 
 
2.96
 
 
-0.16
 
 
0.00514
 
 
 
 
DSIL
 
 
9.92
 
 
28.53
 
 
19.19
 
 
19.34
 
 
3.39
 
 
4.94
 
 
2.58
 
 
-0.08
 
 
0.0258
 
 
 
 
DDAP
 
 
-0.03
 
 
0.06
 
 
0.01
 
 
0.01
 
 
0.01
 
 
0.01
 
 
5.95
 
 
0.84
 
 
1.03e-18
 
 
 
 
SVTh
 
 
62.94
 
 
98.41
 
 
78.09
 
 
78.06
 
 
5.24
 
 
6.87
 
 
3.53
 
 
0.23
 
 
0.00439
 
 
 
 
SVTh*
 
 
67.84
 
 
96.60
 
 
80.20
 
 
80.07
 
 
4.48
 
 
5.54
 
 
3.51
 
 
0.23
 
 
0.00112
 
 
 
 
SVTv
 
 
57.44
 
 
105.29
 
 
78.56
 
 
78.64
 
 
7.05
 
 
9.13
 
 
3.35
 
 
0.09
 
 
0.296
 
 
 
 
SVTv*
 
 
57.74
 
 
102.04
 
 
76.96
 
 
76.77
 
 
7.06
 
 
9.76
 
 
3.11
 
 
0.19
 
 
0.064
 
 
 
 
GAPD
 
 
0.00
 
 
0.04
 
 
0.01
 
 
0.01
 
 
0.01
 
 
0.01
 
 
5.15
 
 
1.50
 
 
1.92e-26
 
 
 
 
GRPD
 
 
0.00
 
 
0.02
 
 
0.01
 
 
0.00
 
 
0.00
 
 
0.00
 
 
7.47
 
 
1.68
 
 
9.39e-25
 
 
 
 
HACP
 
 
-1.60
 
 
63.77
 
 
31.56
 
 
31.84
 
 
10.60
 
 
12.56
 
 
3.31
 
 
-0.25
 
 
0.000325
 
 
 
 
HARC
 
 
1.86
 
 
74.68
 
 
39.92
 
 
41.32
 
 
13.77
 
 
18.63
 
 
2.85
 
 
-0.37
 
 
2.36e-06
 
 
 
 
HARF
 
 
-12.50
 
 
92.17
 
 
22.80
 
 
19.10
 
 
16.57
 
 
20.56
 
 
4.28
 
 
1.13
 
 
3.31e-20
 
 
 
 
HDMP
 
 
-90.36
 
 
132.83
 
 
13.34
 
 
6.32
 
 
23.97
 
 
12.74
 
 
9.60
 
 
1.91
 
 
1.62e-32
 
 
 
 
HCCC
 
 
-0.18
 
 
0.08
 
 
-0.07
 
 
-0.07
 
 
0.04
 
 
0.05
 
 
3.44
 
 
0.09
 
 
0.0458
 
 
 
 
HM2C
 
 
-0.07
 
 
0.01
 
 
-0.04
 
 
-0.04
 
 
0.01
 
 
0.02
 
 
2.88
 
 
0.17
 
 
0.156
 
 
 
 
HMAC
 
 
-0.09
 
 
0.14
 
 
0.04
 
 
0.05
 
 
0.04
 
 
0.05
 
 
3.86
 
 
-0.82
 
 
7.08e-14
 
 
 
 
HMDC
 
 
-0.09
 
 
0.01
 
 
-0.03
 
 
-0.03
 
 
0.01
 
 
0.01
 
 
4.25
 
 
0.35
 
 
5.55e-08
 
 
 
 
HP2C
 
 
-0.10
 
 
0.02
 
 
-0.04
 
 
-0.04
 
 
0.02
 
 
0.03
 
 
2.87
 
 
0.10
 
 
0.45
 
 
 
 
HACL
 
 
1.87
 
 
15.70
 
 
7.95
 
 
7.98
 
 
2.62
 
 
4.26
 
 
2.14
 
 
0.07
 
 
5.37e-09
 
 
 
 
HAML
 
 
23.29
 
 
46.24
 
 
35.49
 
 
35.44
 
 
3.84
 
 
5.19
 
 
2.80
 
 
-0.07
 
 
0.584
 
 
 
 
HAPL
 
 
9.14
 
 
29.03
 
 
18.73
 
 
18.69
 
 
3.33
 
 
4.91
 
 
2.55
 
 
-0.08
 
 
0.00372
 
 
 
 
HBPL
 
 
77.37
 
 
110.83
 
 
91.31
 
 
91.12
 
 
4.72
 
 
6.00
 
 
3.53
 
 
0.30
 
 
0.000868
 
 
 
 
HCTH
 
 
-0.80
 
 
12.80
 
 
5.99
 
 
5.80
 
 
2.34
 
 
3.08
 
 
2.97
 
 
0.23
 
 
0.00145
 
 
 
 
HHPL
 
 
32.26
 
 
61.55
 
 
45.30
 
 
45.06
 
 
4.69
 
 
6.25
 
 
2.86
 
 
0.21
 
 
0.0149
 
 
 
 
HICH
 
 
-3.14
 
 
16.67
 
 
5.86
 
 
5.56
 
 
3.31
 
 
4.44
 
 
2.83
 
 
0.36
 
 
7.98e-06
 
 
 
 
HICL
 
 
7.43
 
 
23.01
 
 
15.06
 
 
15.01
 
 
2.41
 
 
3.22
 
 
3.30
 
 
0.25
 
 
0.00214
 
 
 
 
HICL*
 
 
5.46
 
 
17.30
 
 
11.32
 
 
11.28
 
 
1.75
 
 
2.44
 
 
3.05
 
 
0.10
 
 
0.553
 
 
 
 
HIMH
 
 
2.97
 
 
19.92
 
 
10.72
 
 
10.62
 
 
2.54
 
 
3.03
 
 
3.61
 
 
0.33
 
 
2.88e-05
 
 
 
 
HIML
 
 
31.23
 
 
50.86
 
 
40.92
 
 
41.05
 
 
3.25
 
 
4.17
 
 
3.03
 
 
-0.04
 
 
0.232
 
 
 
 
HIML*
 
 
29.65
 
 
46.73
 
 
37.94
 
 
37.95
 
 
2.88
 
 
3.67
 
 
3.09
 
 
0.05
 
 
0.544
 
 
 
 
HIPH
 
 
4.35
 
 
21.07
 
 
12.62
 
 
12.63
 
 
2.77
 
 
3.42
 
 
3.09
 
 
0.14
 
 
0.0594
 
 
 
 
HIPL
 
 
16.63
 
 
32.28
 
 
24.67
 
 
24.77
 
 
2.66
 
 
3.36
 
 
3.16
 
 
-0.16
 
 
0.0271
 
 
 
 
HIPL*
 
 
13.77
 
 
29.00
 
 
21.55
 
 
21.53
 
 
2.44
 
 
3.07
 
 
3.15
 
 
-0.10
 
 
0.258
 
 
 
 
HMTC
 
 
-0.06
 
 
0.02
 
 
-0.01
 
 
-0.01
 
 
0.01
 
 
0.01
 
 
6.12
 
 
-0.93
 
 
4.78e-17
 
 
 
 
HMTH
 
 
2.82
 
 
21.40
 
 
11.80
 
 
11.79
 
 
2.67
 
 
3.28
 
 
3.67
 
 
0.24
 
 
0.000461
 
 
 
 
HNAP
 
 
4.47
 
 
21.95
 
 
12.67
 
 
12.74
 
 
3.15
 
 
4.11
 
 
2.74
 
 
0.09
 
 
0.0595
 
 
 
 
HNSL
 
 
43.58
 
 
70.48
 
 
56.12
 
 
56.09
 
 
4.03
 
 
4.98
 
 
3.36
 
 
0.08
 
 
0.0787
 
 
 
 
HPTH
 
 
4.21
 
 
18.80
 
 
11.67
 
 
11.50
 
 
2.54
 
 
3.29
 
 
2.98
 
 
0.13
 
 
0.0508
 
 
 
 
HCCP
 
 
-0.01
 
 
0.07
 
 
0.02
 
 
0.02
 
 
0.01
 
 
0.02
 
 
3.37
 
 
0.85
 
 
8.57e-17
 
 
 
 
HCMP
 
 
-0.02
 
 
0.06
 
 
0.01
 
 
0.01
 
 
0.01
 
 
0.01
 
 
7.03
 
 
1.64
 
 
9.79e-26
 
 
 
 
HCPP
 
 
-0.04
 
 
0.07
 
 
0.01
 
 
0.01
 
 
0.02
 
 
0.01
 
 
5.35
 
 
0.23
 
 
4.36e-19
 
 
 
 
HMSP
 
 
-0.01
 
 
0.04
 
 
0.01
 
 
0.01
 
 
0.01
 
 
0.01
 
 
6.62
 
 
1.29
 
 
1.42e-21
 
 
 
 
HBC2
 
 
23.75
 
 
53.24
 
 
34.24
 
 
33.85
 
 
4.25
 
 
5.34
 
 
3.75
 
 
0.64
 
 
7.4e-10
 
 
 
 
HBC3
 
 
19.89
 
 
46.22
 
 
30.61
 
 
30.53
 
 
3.67
 
 
4.90
 
 
3.60
 
 
0.16
 
 
0.00694
 
 
 
 
HBC4
 
 
20.62
 
 
51.83
 
 
35.27
 
 
34.99
 
 
5.34
 
 
7.18
 
 
3.06
 
 
0.26
 
 
0.0076
 
 
 
 
HBNP
 
 
44.43
 
 
80.06
 
 
59.38
 
 
59.08
 
 
5.72
 
 
7.43
 
 
3.08
 
 
0.36
 
 
0.000172
 
 
 
 
HBPG
 
 
32.98
 
 
63.92
 
 
48.44
 
 
48.43
 
 
5.39
 
 
7.44
 
 
2.97
 
 
-0.14
 
 
0.066
 
 
 
 
L2CT
 
 
10.00
 
 
60.05
 
 
31.92
 
 
32.30
 
 
7.78
 
 
9.68
 
 
3.38
 
 
0.06
 
 
0.0225
 
 
 
 
L2EA
 
 
5.83
 
 
33.73
 
 
15.12
 
 
13.93
 
 
5.17
 
 
6.34
 
 
3.75
 
 
1.03
 
 
1.36e-19
 
 
 
 
L2EP
 
 
21.63
 
 
71.35
 
 
45.35
 
 
45.34
 
 
7.40
 
 
9.34
 
 
3.40
 
 
0.19
 
 
0.0296
 
 
 
 
L3CT
 
 
-5.95
 
 
40.31
 
 
15.68
 
 
15.39
 
 
6.78
 
 
8.50
 
 
3.39
 
 
0.22
 
 
0.00863
 
 
 
 
L3EA
 
 
7.68
 
 
32.57
 
 
16.78
 
 
16.31
 
 
4.64
 
 
5.86
 
 
3.25
 
 
0.66
 
 
8.49e-12
 
 
 
 
L3EP
 
 
15.68
 
 
59.84
 
 
34.05
 
 
33.87
 
 
5.97
 
 
7.19
 
 
3.82
 
 
0.30
 
 
9.53e-05
 
 
 
 
L4CT
 
 
-3.76
 
 
20.20
 
 
8.76
 
 
8.60
 
 
3.36
 
 
4.36
 
 
3.33
 
 
0.32
 
 
5.52e-05
 
 
 
 
L4EA
 
 
9.85
 
 
48.77
 
 
28.87
 
 
28.71
 
 
6.63
 
 
8.75
 
 
2.89
 
 
0.16
 
 
0.052
 
 
 
 
L4EP
 
 
12.81
 
 
45.96
 
 
29.45
 
 
29.42
 
 
4.47
 
 
5.55
 
 
3.77
 
 
0.02
 
 
0.00134
 
 
 
 
L5CT
 
 
3.61
 
 
35.37
 
 
17.93
 
 
17.49
 
 
5.74
 
 
7.37
 
 
2.83
 
 
0.29
 
 
0.000525
 
 
 
 
L5EA
 
 
20.86
 
 
63.70
 
 
42.06
 
 
42.12
 
 
7.14
 
 
9.47
 
 
2.77
 
 
-0.01
 
 
0.734
 
 
 
 
L5EP
 
 
17.22
 
 
48.81
 
 
32.48
 
 
32.08
 
 
4.82
 
 
6.23
 
 
3.14
 
 
0.22
 
 
0.0338
 
 
 
 
LACT
 
 
37.63
 
 
91.10
 
 
62.88
 
 
63.02
 
 
7.94
 
 
10.65
 
 
3.33
 
 
0.04
 
 
0.124
 
 
 
 
LAEA
 
 
18.81
 
 
65.55
 
 
40.72
 
 
39.92
 
 
7.49
 
 
9.56
 
 
3.15
 
 
0.42
 
 
7.5e-07
 
 
 
 
LAEP
 
 
48.38
 
 
103.47
 
 
72.43
 
 
72.23
 
 
7.73
 
 
9.98
 
 
3.37
 
 
0.15
 
 
0.0993
 
 
 
 
LEPL
 
 
9.70
 
 
53.91
 
 
33.05
 
 
33.26
 
 
6.25
 
 
7.98
 
 
3.52
 
 
-0.20
 
 
0.00275
 
 
 
 
LHCT
 
 
15.86
 
 
43.98
 
 
29.10
 
 
29.01
 
 
4.37
 
 
5.11
 
 
3.46
 
 
0.31
 
 
0.000188
 
 
 
 
LHEA
 
 
5.48
 
 
37.13
 
 
20.45
 
 
20.62
 
 
4.19
 
 
5.31
 
 
3.69
 
 
-0.06
 
 
0.0038
 
 
 
 
LHEP
 
 
3.16
 
 
35.14
 
 
21.15
 
 
21.12
 
 
4.89
 
 
6.13
 
 
3.18
 
 
0.01
 
 
0.311
 
 
 
 
LNCT
 
 
57.74
 
 
102.04
 
 
76.96
 
 
76.77
 
 
7.06
 
 
9.76
 
 
3.11
 
 
0.19
 
 
0.064
 
 
 
 
LNEA
 
 
34.61
 
 
72.09
 
 
52.81
 
 
52.35
 
 
5.99
 
 
7.86
 
 
2.95
 
 
0.27
 
 
0.00131
 
 
 
 
LNEP
 
 
57.44
 
 
105.29
 
 
78.56
 
 
78.64
 
 
7.05
 
 
9.13
 
 
3.35
 
 
0.09
 
 
0.296
 
 
 
 
MCGM
 
 
97.00
 
 
132.18
 
 
115.09
 
 
115.01
 
 
5.89
 
 
7.70
 
 
3.10
 
 
0.10
 
 
0.169
 
 
 
 
MCGP
 
 
90.10
 
 
126.61
 
 
108.63
 
 
108.63
 
 
5.72
 
 
7.31
 
 
3.10
 
 
0.14
 
 
0.23
 
 
 
 
MCGD
 
 
34.33
 
 
57.45
 
 
47.10
 
 
46.89
 
 
3.97
 
 
5.46
 
 
2.64
 
 
0.02
 
 
0.0142
 
 
 
 
MCGL
 
 
35.24
 
 
61.56
 
 
48.61
 
 
48.35
 
 
4.61
 
 
6.13
 
 
2.77
 
 
0.12
 
 
0.088
 
 
 
 
MCGR
 
 
36.63
 
 
62.94
 
 
49.45
 
 
49.50
 
 
4.15
 
 
5.65
 
 
2.65
 
 
-0.04
 
 
0.116
 
 
 
 
MCMD
 
 
82.40
 
 
118.86
 
 
99.17
 
 
99.38
 
 
5.28
 
 
6.88
 
 
3.24
 
 
-0.08
 
 
0.274
 
 
 
 
MCPD
 
 
83.81
 
 
115.85
 
 
98.52
 
 
98.49
 
 
5.11
 
 
6.60
 
 
3.11
 
 
-0.07
 
 
0.466
 
 
 
 
MGMD
 
 
55.36
 
 
87.30
 
 
69.80
 
 
69.94
 
 
4.79
 
 
6.14
 
 
3.20
 
 
-0.03
 
 
0.301
 
 
 
 
MGPD
 
 
57.92
 
 
88.19
 
 
73.03
 
 
73.20
 
 
4.87
 
 
6.46
 
 
3.03
 
 
-0.09
 
 
0.596
 
 
 
 
MICD
 
 
95.53
 
 
134.02
 
 
113.21
 
 
113.32
 
 
6.82
 
 
8.85
 
 
2.87
 
 
-0.02
 
 
0.0439
 
 
 
 
MIGD
 
 
70.09
 
 
103.12
 
 
86.87
 
 
87.29
 
 
5.63
 
 
7.02
 
 
3.17
 
 
-0.45
 
 
3.75e-08
 
 
 
 
MMPG
 
 
4.12
 
 
15.23
 
 
9.02
 
 
9.00
 
 
1.82
 
 
2.44
 
 
2.97
 
 
0.21
 
 
0.00737
 
 
 
 
MPGL
 
 
71.96
 
 
98.90
 
 
85.48
 
 
85.70
 
 
4.66
 
 
6.29
 
 
3.03
 
 
-0.08
 
 
0.166
 
 
 
 
MPGR
 
 
70.61
 
 
101.05
 
 
84.64
 
 
84.84
 
 
4.70
 
 
6.53
 
 
2.92
 
 
-0.06
 
 
0.606
 
 
 
 
OCHL
 
 
-0.46
 
 
2.29
 
 
0.78
 
 
0.76
 
 
0.43
 
 
0.58
 
 
2.98
 
 
0.22
 
 
0.0356
 
 
 
 
OCHW
 
 
-0.11
 
 
0.76
 
 
0.27
 
 
0.24
 
 
0.15
 
 
0.20
 
 
2.96
 
 
0.45
 
 
1.1e-07
 
 
 
 
OCLW
 
 
0.70
 
 
7.79
 
 
3.15
 
 
2.85
 
 
1.20
 
 
1.76
 
 
3.23
 
 
0.75
 
 
1.58e-15
 
 
 
 
OCMR
 
 
0.44
 
 
0.77
 
 
0.61
 
 
0.61
 
 
0.05
 
 
0.07
 
 
3.14
 
 
0.12
 
 
0.164
 
 
 
 
OCPR
 
 
0.56
 
 
0.93
 
 
0.74
 
 
0.74
 
 
0.06
 
 
0.08
 
 
3.15
 
 
0.22
 
 
0.00653
 
 
 
 
OMHL
 
 
0.07
 
 
0.60
 
 
0.31
 
 
0.30
 
 
0.08
 
 
0.11
 
 
3.57
 
 
0.54
 
 
4.2e-08
 
 
 
 
OMHW
 
 
0.07
 
 
0.54
 
 
0.30
 
 
0.29
 
 
0.07
 
 
0.09
 
 
3.61
 
 
0.37
 
 
2.32e-06
 
 
 
 
OMLW
 
 
0.65
 
 
1.50
 
 
1.03
 
 
1.02
 
 
0.15
 
 
0.20
 
 
2.79
 
 
0.24
 
 
0.00722
 
 
 
 
OPHL
 
 
0.14
 
 
1.34
 
 
0.69
 
 
0.68
 
 
0.18
 
 
0.23
 
 
3.56
 
 
0.40
 
 
1.61e-05
 
 
 
 
OPHW
 
 
0.11
 
 
0.76
 
 
0.42
 
 
0.42
 
 
0.11
 
 
0.14
 
 
3.18
 
 
0.32
 
 
0.000148
 
 
 
 
OPLW
 
 
0.88
 
 
2.74
 
 
1.64
 
 
1.62
 
 
0.32
 
 
0.46
 
 
3.04
 
 
0.43
 
 
6.76e-07
 
 
 
 
OPMR
 
 
0.62
 
 
1.05
 
 
0.83
 
 
0.83
 
 
0.06
 
 
0.09
 
 
3.05
 
 
0.05
 
 
0.922
 
 
 
 
PPW2
 
 
-0.03
 
 
0.04
 
 
0.00
 
 
0.00
 
 
0.01
 
 
0.00
 
 
6.10
 
 
0.44
 
 
8.74e-26
 
 
 
 
PPW3
 
 
-0.02
 
 
0.02
 
 
0.00
 
 
0.00
 
 
0.01
 
 
0.00
 
 
5.57
 
 
0.58
 
 
5.19e-22
 
 
 
 
PPW4
 
 
-0.01
 
 
0.01
 
 
0.00
 
 
0.00
 
 
0.00
 
 
0.00
 
 
5.23
 
 
0.33
 
 
9.04e-19
 
 
 
 
PPW5
 
 
-0.01
 
 
0.01
 
 
0.00
 
 
0.00
 
 
0.00
 
 
0.00
 
 
4.42
 
 
0.26
 
 
2.32e-10
 
 
 
 
PPW6
 
 
0.00
 
 
0.01
 
 
0.00
 
 
0.00
 
 
0.00
 
 
0.00
 
 
3.73
 
 
0.47
 
 
1.28e-07
 
 
 
 
PPW7
 
 
0.00
 
 
0.00
 
 
0.00
 
 
0.00
 
 
0.00
 
 
0.00
 
 
2.49
 
 
-0.02
 
 
0.00467
 
 
 
 
PPWC
 
 
-0.01
 
 
0.02
 
 
0.00
 
 
0.00
 
 
0.00
 
 
0.00
 
 
6.72
 
 
0.60
 
 
3e-25
 
 
 
 
PNNP
 
 
11.22
 
 
36.92
 
 
24.34
 
 
24.23
 
 
3.81
 
 
5.34
 
 
3.20
 
 
0.03
 
 
0.368
 
 
 
 
PWTP
 
 
0.00
 
 
0.00
 
 
0.00
 
 
0.00
 
 
0.00
 
 
0.00
 
 
4.15
 
 
0.52
 
 
3.01e-10
 
 
 
 
AANP
 
 
-1.15
 
 
11.32
 
 
3.76
 
 
3.59
 
 
2.00
 
 
2.76
 
 
3.10
 
 
0.53
 
 
3.37e-09
 
 
 
 
AASN
 
 
72.83
 
 
101.46
 
 
86.53
 
 
86.48
 
 
4.68
 
 
6.23
 
 
3.01
 
 
0.19
 
 
0.159
 
 
 
 
ABSN
 
 
111.02
 
 
148.71
 
 
128.57
 
 
128.28
 
 
5.84
 
 
7.82
 
 
2.97
 
 
0.10
 
 
0.647
 
 
 
 
ACSN
 
 
91.73
 
 
139.57
 
 
114.54
 
 
114.68
 
 
7.23
 
 
9.93
 
 
3.20
 
 
0.11
 
 
0.0604
 
 
 
 
ALNS
 
 
-0.86
 
 
18.32
 
 
7.33
 
 
7.14
 
 
3.51
 
 
4.94
 
 
2.66
 
 
0.27
 
 
3.63e-05
 
 
 
 
ANNF
 
 
80.30
 
 
104.16
 
 
91.56
 
 
91.38
 
 
3.89
 
 
5.38
 
 
2.84
 
 
0.12
 
 
0.261
 
 
 
 
ANSF
 
 
81.81
 
 
107.86
 
 
95.48
 
 
95.44
 
 
4.46
 
 
6.20
 
 
2.91
 
 
-0.13
 
 
0.384
 
 
 
 
APNP
 
 
-2.60
 
 
12.02
 
 
4.72
 
 
4.59
 
 
2.36
 
 
3.35
 
 
2.78
 
 
0.34
 
 
1.52e-06
 
 
 
 
APNS
 
 
65.37
 
 
90.98
 
 
78.89
 
 
78.75
 
 
4.38
 
 
6.08
 
 
2.86
 
 
0.12
 
 
0.082
 
 
 
 
APSN
 
 
61.40
 
 
94.18
 
 
76.89
 
 
77.06
 
 
5.25
 
 
6.87
 
 
2.90
 
 
-0.09
 
 
0.348
 
 
 
 
ASCG
 
 
97.00
 
 
132.18
 
 
115.09
 
 
115.01
 
 
5.89
 
 
7.70
 
 
3.10
 
 
0.10
 
 
0.169
 
 
 
 
ASCG*
 
 
138.25
 
 
179.51
 
 
162.64
 
 
163.02
 
 
7.52
 
 
10.56
 
 
2.54
 
 
-0.23
 
 
3.87e-05
 
 
 
 
ASNP
 
 
71.89
 
 
96.20
 
 
83.33
 
 
83.10
 
 
4.24
 
 
5.59
 
 
2.85
 
 
0.25
 
 
0.00367
 
 
 
 
SBAN
 
 
85.97
 
 
113.47
 
 
99.44
 
 
99.25
 
 
4.29
 
 
5.36
 
 
3.28
 
 
0.03
 
 
0.241
 
 
 
 
SBAP
 
 
77.37
 
 
110.83
 
 
91.31
 
 
91.12
 
 
4.72
 
 
6.00
 
 
3.53
 
 
0.30
 
 
0.000868
 
 
 
 
SBAS
 
 
30.03
 
 
56.00
 
 
41.79
 
 
41.87
 
 
3.67
 
 
4.51
 
 
3.66
 
 
0.05
 
 
0.00179
 
 
 
 
SBNA
 
 
78.46
 
 
111.26
 
 
92.50
 
 
92.27
 
 
4.72
 
 
6.23
 
 
3.45
 
 
0.24
 
 
0.0111
 
 
 
 
SBNP
 
 
25.65
 
 
49.08
 
 
36.73
 
 
36.46
 
 
3.80
 
 
5.11
 
 
2.90
 
 
0.29
 
 
0.00104
 
 
 
 
SHBN
 
 
165.00
 
 
207.70
 
 
183.99
 
 
184.03
 
 
6.82
 
 
9.23
 
 
3.27
 
 
0.19
 
 
0.00781
 
 
 
 
SHSW
 
 
108.82
 
 
150.12
 
 
128.49
 
 
128.66
 
 
7.29
 
 
10.28
 
 
2.74
 
 
-0.08
 
 
0.217
 
 
 
 
SIOD
 
 
59.03
 
 
91.58
 
 
73.57
 
 
73.17
 
 
5.26
 
 
7.03
 
 
2.98
 
 
0.21
 
 
0.0249
 
 
 
 
SNAM
 
 
92.66
 
 
133.29
 
 
111.38
 
 
111.16
 
 
7.13
 
 
9.81
 
 
2.84
 
 
0.05
 
 
0.394
 
 
 
 
SNAP
 
 
85.52
 
 
125.26
 
 
104.60
 
 
104.42
 
 
6.86
 
 
9.14
 
 
2.79
 
 
0.03
 
 
0.687
 
 
 
 
SNNA
 
 
37.25
 
 
60.62
 
 
48.03
 
 
47.94
 
 
3.47
 
 
4.94
 
 
3.04
 
 
0.13
 
 
0.404
 
 
 
 
SNNP
 
 
59.21
 
 
89.25
 
 
71.54
 
 
71.71
 
 
3.98
 
 
4.79
 
 
4.00
 
 
0.05
 
 
2.76e-05
 
 
 
 
SNOL
 
 
42.20
 
 
60.24
 
 
50.83
 
 
50.84
 
 
2.95
 
 
3.79
 
 
3.02
 
 
0.11
 
 
0.289
 
 
 
 
SNOR
 
 
41.11
 
 
59.30
 
 
50.53
 
 
50.58
 
 
2.92
 
 
3.85
 
 
3.17
 
 
-0.05
 
 
0.481
 
 
 
 
SSEG
 
 
15.25
 
 
40.05
 
 
25.86
 
 
25.78
 
 
3.30
 
 
3.96
 
 
3.79
 
 
0.22
 
 
0.000881
 
 
 
 
SSEN
 
 
57.71
 
 
80.91
 
 
68.13
 
 
68.05
 
 
3.21
 
 
3.82
 
 
3.74
 
 
0.29
 
 
0.000255
 
 
 
 
SSEN*
 
 
31.24
 
 
54.20
 
 
42.32
 
 
42.43
 
 
3.21
 
 
4.10
 
 
3.49
 
 
0.06
 
 
0.101
 
 
 
 
SPUN
 
 
11.70
 
 
38.20
 
 
25.99
 
 
26.21
 
 
4.28
 
 
5.70
 
 
2.88
 
 
-0.10
 
 
0.545
 
 
 
 
 
 
 
 
 Inter-correlations and similarities between measures 
 
 
  Table 5.    Correlations between measures
(showing those with Pearson’s  r  >= 0.90). 
 
 
 
 
measure1
 
 
measure2
 
 
Pearson’s  r 
 
 
 p 
 
 
Are they identical?
 
 
 
 
 
 
SVTv
 
 
LNEP
 
 
1.000
 
 
0
 
 
Yes
 
 
 
 
MCGM
 
 
ASCG
 
 
1.000
 
 
0
 
 
Yes
 
 
 
 
HBPL
 
 
SBAP
 
 
1.000
 
 
0
 
 
Yes
 
 
 
 
SVTv*
 
 
LNCT
 
 
1.000
 
 
0
 
 
Yes
 
 
 
 
HICH
 
 
OCHW
 
 
0.984
 
 
0
 
 
No
 
 
 
 
DAIM
 
 
DAIP
 
 
0.981
 
 
0
 
 
No
 
 
 
 
SNAM
 
 
SNAP
 
 
0.967
 
 
0
 
 
No
 
 
 
 
MCGM
 
 
MCGP
 
 
0.960
 
 
0
 
 
No
 
 
 
 
MCGP
 
 
ASCG
 
 
0.960
 
 
0
 
 
No
 
 
 
 
MCMD
 
 
MCPD
 
 
0.959
 
 
0
 
 
No
 
 
 
 
L2CT
 
 
L3CT
 
 
0.957
 
 
0
 
 
No
 
 
 
 
MGMD
 
 
MGPD
 
 
0.946
 
 
0
 
 
No
 
 
 
 
L2EP
 
 
L3EP
 
 
0.946
 
 
0
 
 
No
 
 
 
 
MGPD
 
 
MPGR
 
 
0.935
 
 
0
 
 
No
 
 
 
 
L4EA
 
 
L5EA
 
 
0.933
 
 
0
 
 
No
 
 
 
 
MCGD
 
 
MCGL
 
 
0.925
 
 
0
 
 
No
 
 
 
 
MGPD
 
 
MPGL
 
 
0.924
 
 
0
 
 
No
 
 
 
 
L2CT
 
 
LACT
 
 
0.921
 
 
0
 
 
No
 
 
 
 
SVTh*
 
 
HBPL
 
 
0.920
 
 
0
 
 
No
 
 
 
 
SVTh*
 
 
SBAP
 
 
0.920
 
 
0
 
 
No
 
 
 
 
HIMH
 
 
OMHW
 
 
0.916
 
 
0
 
 
No
 
 
 
 
L2EP
 
 
LAEP
 
 
0.912
 
 
0
 
 
No
 
 
 
 
MCGD
 
 
MCGR
 
 
0.912
 
 
0
 
 
No
 
 
 
 
HAML
 
 
HAPL
 
 
0.904
 
 
0
 
 
No
 
 
 
 
 
 Measures pefectly correlated 
 Here we manually check the  meaning  of those measures that
have a correlation of 1.0 and are numerically identical. 
 
 
  Table 6.    Comparing LNEP and SVTv: they are
indeed the same thing, we’ll keep SVTv. 
 
 
 
 
measure
 
 
full name
 
 
description
 
 
 
 
 
 
SVTv
 
 
GENERAL_SVTV_NSP_EPIGLOTTISPETIOLE_DISTANCE
 
 
Length of the vertical supralaryngeal vocal tract (using the petiole of
the epiglottis and and the posterior nasal spine)
 
 
 
 
LNEP
 
 
LARYNX_LNEP_NSP_EPIGLOTTISPETIOLE_DISTANCE
 
 
Height of larynx (using petiole of epiglottis) relative to posterior
nasal spine
 
 
 
 
 
 
  Table 7.    Comparing ASCG and MCGM: they are
indeed the same thing, we’ll keep MCGM. 
 
 
 
 
measure
 
 
full name
 
 
description
 
 
 
 
 
 
MCGM
 
 
MANDIBLE_MCGM_GONIONMEANCONYLIONMEAN_GONIONMEANMENTON_ANGLE
 
 
Angle of the mandible using the menton and the mean locations of the
condyles and angles (gonion)
 
 
 
 
ASCG
 
 
SKULLANGLE_ASCG_GONIONMEANCONDYLIONMEAN_GONIONMEANMENTON_A
 
 
Angle between the line from the mean gonion location to the mean condyle
location and the line from the mean gonion location to menton
 
 
 
 
 
 
  Table 8.    Comparing HBPL and SBAP: they are
indeed the same thing, we’ll keep HBPL. 
 
 
 
 
measure
 
 
full name
 
 
description
 
 
 
 
 
 
HBPL
 
 
HARDPALATE_HBPL_BASION_PROSTHION_DISTANCE
 
 
Length (anteroposterior distance) of hard palate (lower face)
 
 
 
 
SBAP
 
 
SKULL_SBAP_BASION_PROSTHION_DISTANCE
 
 
Length of facial skeleton (using prosthion)
 
 
 
 
 
 
  Table 9.   _Comparing SVTv* and LNCT: they are
indeed the same thing, we’ll keep SVTv*._
 
 
 
 
measure
 
 
full name
 
 
description
 
 
 
 
 
 
SVTv*
 
 
GENERAL_SVTV_NSP_CORNICULATETUBERCLE_DISTANCE
 
 
Length of the vertical supralaryngeal vocal tract (using the corniculate
tubercle and the posterior nasal spine)
 
 
 
 
LNCT
 
 
LARYNX_LNCT_NSP_CORNICULATETUBERCLE_DISTANCE
 
 
Height of larynx (using corniculate tubercle) relative to posterior
nasal spine
 
 
 
 
 Removing these four redundant measures (LNEP, ASCG, SBAP, LNCT)
reduces the number of measures to 146; however, this procedure leaves
untouched the several very highly correlated measures, whose
correlations seem to be substantively motivated but not  a
priori  trivial. 
 
 Distributions and summaries of the remaning measures 
 
 By domain 
  Counts:  
 
 Table continues below 
 
 
 
 
 
 
 
 
 
 
 
 cervical 
 dentition 
 general 
 hard palate 
 hyoid 
 larynx 
 mandible 
 
 
 
 
 7 
 11 
 6 
 33 
 5 
 20 
 14 
 
 
 
 
 
 
 
 
 
 
 
 
 oral 
 pharynx 
 skull 
 soft palate 
 
 
 
 
 12 
 9 
 28 
 1 
 
 
 
  Percents (%):  
 
 Table continues below 
 
 
 
 
 
 
 
 
 
 
 
 cervical 
 dentition 
 general 
 hard palate 
 hyoid 
 larynx 
 mandible 
 
 
 
 
 4.795 
 7.534 
 4.11 
 22.6 
 3.425 
 13.7 
 9.589 
 
 
 
 
 
 
 
 
 
 
 
 
 oral 
 pharynx 
 skull 
 soft palate 
 
 
 
 
 8.219 
 6.164 
 19.18 
 0.6849 
 
 
 
 
 
 By type 
  Counts:  
 
 
 
 
 
 
 
 
 
 
 angle 
 curvature 
 distance 
 Procrustes dist. 
 ratio 
 
 
 
 
 22 
 13 
 91 
 8 
 12 
 
 
 
  Percents (%):  
 
 
 
 
 
 
 
 
 
 
 angle 
 curvature 
 distance 
 Procrustes dist. 
 ratio 
 
 
 
 
 15.07 
 8.904 
 62.33 
 5.479 
 8.219 
 
 
 
 
 
 
 
 
 Correlations between the twins 
 Here we look, for each measure, at the relationship between the
values of the members of the same twin pair. 
 
 All twins together 
 
 
 
  Figure 6.    Scatterplot of the values for
the two twins in the same pair for each measure separately, for each
rater (color) and together (black line). All twins (MZ and DZ) are
considered. Please note that the  x  and  y  scales are
independent between plots. 
 
 
 
 
 MZ twins only 
 
 
 
  Figure 7.    Scatterplot of the values for
the two twins in the same pair for each measure separately, for each
rater (color) and together (black line). Only MZ twins are considered.
Please note that the  x  and  y  scales are independent
between plots. 
 
 
 
 
 DZ twins only 
 
 
 
  Figure 8.    Scatterplot of the values for
the two twins in the same pair for each measure separately, for each
rater (color) and together (black line). Only DZ twins are considered.
Please note that the  x  and  y  scales are independent
between plots. 
 
 
 
 
 
 Inter-raters agreement 
 Given that all our data was landmarked in parallel by two raters, we
can compute the inter-rater agreement for all our variables. We used
both  Krippendorff’s α  and the intraclass correlations
coefficient ( ICC ); for both, values closer to 1.0 indicate high
agreement, 0.0 means randomness, and negative values indicate systematic
disagreement. Please note that the GSEM model used to estimate
heritability (see Section  The SEM
genetic model ) also allows us to estimate a measure of inter-rater
agreement defined as (1 - {standardized rater error variance}) =
( A  + ( C  or  D ) +  E )/{the observed
phenotypic variance,  var (phenotype)} – we will denote this as
 agr (GSEM). 
 
 Bland–Altman plots 
 For a given measure, the Bland–Altman plot represents, for each
measured participant, the relationship between the mean of the two
raters (on the x-axis) and their difference (on the y axis). The plot
also shows the mean difference (or the fixed bias between the two
raters) and the ± 1.96 SD lines (allowing the identification of outlier
participants in terms of the difference between the two raters). If the
range between these two lines, i.e., between (mean - 1.96 SD) and (mean
+ 1.96 SD) are not substantively important, the two raters can be
considered as indistinguishable. 
 
 
   Figure 1.    The
Bland–Altman plots for each measure. The x-axis is the mean of the two
raters, while the y-axis is the difference between the two raters (here,
rater 1 - rater 2). The blue dotted line in the middle is the mean
difference, while the two red dotted lines above and below it are the
mean difference ± 1.96 SD. Please see text on how to interpret such
plots.  
 
 Of the 146 measures, 71 have a significantly positive fixed bias
(i.e., the 95% CI of the fixed bias is above 0, meaning that rater 1
systematically produced higher values than rater 2), 75 have a
significantly negative fixed bias, and the renaming 0 have a
non-significant fixed bias. However, even the statistically significant
fixed biases are small when compared with the range of the measures. 
 Inspecting the plots shows that for most measures there seems to be
no problematic relationship between mean and difference, for very few
measure there are potentially problematic patterns: 
 
 GRPD: the difference seems to be larger at larger means 
 PPW2, PPW3, PPW4, PPWC: perfect relationships between mean and
difference 
 
 Taken together, these suggest that the two raters are indeed very
much in agreement for the large majority of the measures. 
 
 
 By measure 
  ICC(C,1):  
 
 
 
 
 
 
 
 
 
 
 
 Min. 
 1st Qu. 
 Median 
 Mean 
 3rd Qu. 
 Max. 
 
 
 
 
 0.06739 
 0.4367 
 0.6285 
 0.6003 
 0.7832 
 0.9826 
 
 
 
  SD = 0.2261217  IQR = 0.3465256   
  Krippendorff’s α:  
 
 
 
 
 
 
 
 
 
 
 
 
 Min. 
 1st Qu. 
 Median 
 Mean 
 3rd Qu. 
 Max. 
 NA’s 
 
 
 
 
 -6.165 
 0.4567 
 0.673 
 0.5687 
 0.8704 
 0.9999 
 5 
 
 
 
  SD = 0.6521419  IQR = 0.4137198   
  agr(GSEM):  
 
 
 
 
 
 
 
 
 
 
 
 Min. 
 1st Qu. 
 Median 
 Mean 
 3rd Qu. 
 Max. 
 
 
 
 
 0.08687 
 0.413 
 0.5973 
 0.5676 
 0.7483 
 0.9686 
 
 
 
  SD = 0.2179913  IQR = 0.3352556   
 
 
 
  Figure 10.    Histograms of Krippendorff’s α
(including and excluding the negative values, left and middle plots
respectively), of ICC(C,1) in the middle, and of  agr (GSEM) on
the right, as well as the scatterplots of these measures (excluding the
1 extreme negative outlier for Krippendorff’s α). Correlations:
Krippendorff’s α x ICC(C,1): Pearson’s  r  = 0.34,  p  =
4.87e-05, Spearman’s  ρ  = 0.32,  p  = 0.000149;
Krippendorff’s α x  agr (GSEM): Pearson’s  r  = 0.34,
 p  = 3.57e-05, Spearman’s  ρ  = 0.31,  p  =
0.000246; ICC(C,1) x  agr (GSEM): Pearson’s  r  = 0.99,
 p  = 4.13e-112, Spearman’s  ρ  = 0.99,  p  =
0. 
 
 
 
 
 
  Figure 11.    Inter-rater reliability
estimates ordered by decreasing ICC(C,1) values (blue triangle), also
showing the Krippendorff’s α (red circle) and  agr (GSEM) as
black squares. The plot is split in two for better fit
horizontally. 
 
 
 
 
  Table 10.    The measures ordered by
decreasing ICC(C,1),  agr (GSEM), and Krippendorff’s α. 
 
 
 
 
measure
 
 
ICC(C,1)
 
 
 agr (GSEM)
 
 
Krippendorff’s α
 
 
 
 
 
 
MICD
 
 
0.98
 
 
0.97
 
 
0.98
 
 
 
 
SHBN
 
 
0.96
 
 
0.94
 
 
0.96
 
 
 
 
MIGD
 
 
0.95
 
 
0.89
 
 
0.94
 
 
 
 
SHSW
 
 
0.94
 
 
0.88
 
 
0.46
 
 
 
 
SVTh*
 
 
0.93
 
 
0.91
 
 
0.93
 
 
 
 
MCPD
 
 
0.92
 
 
0.87
 
 
0.89
 
 
 
 
MCMD
 
 
0.92
 
 
0.87
 
 
0.91
 
 
 
 
HBPL
 
 
0.91
 
 
0.88
 
 
0.90
 
 
 
 
SSEN
 
 
0.90
 
 
0.88
 
 
0.87
 
 
 
 
SNAM
 
 
0.90
 
 
0.86
 
 
0.87
 
 
 
 
SBNA
 
 
0.90
 
 
0.87
 
 
0.89
 
 
 
 
SVTh
 
 
0.90
 
 
0.88
 
 
0.56
 
 
 
 
SSEN*
 
 
0.90
 
 
0.86
 
 
0.89
 
 
 
 
SSEG
 
 
0.90
 
 
0.88
 
 
0.90
 
 
 
 
SBAN
 
 
0.89
 
 
0.85
 
 
0.88
 
 
 
 
MCGM
 
 
0.89
 
 
0.88
 
 
0.88
 
 
 
 
SNAP
 
 
0.88
 
 
0.83
 
 
0.87
 
 
 
 
ASCG*
 
 
0.87
 
 
0.88
 
 
0.87
 
 
 
 
ACSN
 
 
0.87
 
 
0.86
 
 
0.84
 
 
 
 
MCGP
 
 
0.87
 
 
0.86
 
 
0.86
 
 
 
 
MPGL
 
 
0.84
 
 
0.78
 
 
0.81
 
 
 
 
MPGR
 
 
0.84
 
 
0.78
 
 
0.81
 
 
 
 
HBNP
 
 
0.84
 
 
0.72
 
 
0.83
 
 
 
 
MGPD
 
 
0.83
 
 
0.80
 
 
0.80
 
 
 
 
APNS
 
 
0.83
 
 
0.82
 
 
0.67
 
 
 
 
SNNP
 
 
0.82
 
 
0.76
 
 
0.77
 
 
 
 
ABSN
 
 
0.82
 
 
0.82
 
 
0.76
 
 
 
 
LACT
 
 
0.81
 
 
0.74
 
 
0.74
 
 
 
 
HBPG
 
 
0.81
 
 
0.79
 
 
0.81
 
 
 
 
SVTv*
 
 
0.80
 
 
0.69
 
 
0.78
 
 
 
 
SBNP
 
 
0.80
 
 
0.79
 
 
0.80
 
 
 
 
MGMD
 
 
0.80
 
 
0.76
 
 
0.79
 
 
 
 
GAPD
 
 
0.80
 
 
0.77
 
 
0.81
 
 
 
 
DIPD
 
 
0.80
 
 
0.78
 
 
0.80
 
 
 
 
HBC4
 
 
0.79
 
 
0.79
 
 
0.78
 
 
 
 
SBAS
 
 
0.79
 
 
0.76
 
 
0.74
 
 
 
 
ANSF
 
 
0.79
 
 
0.77
 
 
0.78
 
 
 
 
HBC2
 
 
0.78
 
 
0.65
 
 
0.76
 
 
 
 
HNSL
 
 
0.77
 
 
0.73
 
 
0.76
 
 
 
 
DIMD
 
 
0.77
 
 
0.73
 
 
0.76
 
 
 
 
LAEA
 
 
0.77
 
 
0.71
 
 
0.76
 
 
 
 
APNP
 
 
0.76
 
 
0.76
 
 
1.00
 
 
 
 
L4EA
 
 
0.76
 
 
0.75
 
 
0.74
 
 
 
 
L3EA
 
 
0.76
 
 
0.75
 
 
0.71
 
 
 
 
LNEA
 
 
0.75
 
 
0.69
 
 
0.74
 
 
 
 
ANNF
 
 
0.74
 
 
0.74
 
 
0.67
 
 
 
 
PNNP
 
 
0.74
 
 
0.74
 
 
0.65
 
 
 
 
LAEP
 
 
0.74
 
 
0.66
 
 
0.71
 
 
 
 
L5EA
 
 
0.74
 
 
0.72
 
 
0.72
 
 
 
 
SVTv
 
 
0.73
 
 
0.61
 
 
0.72
 
 
 
 
L2CT
 
 
0.73
 
 
0.68
 
 
0.67
 
 
 
 
APSN
 
 
0.73
 
 
0.72
 
 
0.70
 
 
 
 
ASNP
 
 
0.72
 
 
0.71
 
 
0.59
 
 
 
 
L5CT
 
 
0.71
 
 
0.69
 
 
0.63
 
 
 
 
MCGD
 
 
0.71
 
 
0.60
 
 
0.70
 
 
 
 
DICD
 
 
0.71
 
 
0.68
 
 
0.70
 
 
 
 
HBC3
 
 
0.71
 
 
0.63
 
 
0.70
 
 
 
 
SIOD
 
 
0.71
 
 
0.61
 
 
0.24
 
 
 
 
PPW7
 
 
0.71
 
 
0.64
 
 
-0.05
 
 
 
 
OMLW
 
 
0.70
 
 
0.63
 
 
0.21
 
 
 
 
L3CT
 
 
0.69
 
 
0.66
 
 
0.95
 
 
 
 
CS7H
 
 
0.69
 
 
0.66
 
 
0.62
 
 
 
 
MCGR
 
 
0.68
 
 
0.58
 
 
0.67
 
 
 
 
MCGL
 
 
0.68
 
 
0.58
 
 
0.67
 
 
 
 
L2EP
 
 
0.67
 
 
0.61
 
 
0.51
 
 
 
 
LHCT
 
 
0.66
 
 
0.58
 
 
0.63
 
 
 
 
L5EP
 
 
0.66
 
 
0.65
 
 
0.51
 
 
 
 
DAII
 
 
0.66
 
 
0.66
 
 
0.58
 
 
 
 
SNNA
 
 
0.66
 
 
0.57
 
 
0.55
 
 
 
 
L2EA
 
 
0.65
 
 
0.61
 
 
0.63
 
 
 
 
HIML*
 
 
0.65
 
 
0.61
 
 
0.64
 
 
 
 
DAIS
 
 
0.65
 
 
0.65
 
 
0.55
 
 
 
 
HIML
 
 
0.63
 
 
0.57
 
 
0.57
 
 
 
 
SPUN
 
 
0.63
 
 
0.61
 
 
0.54
 
 
 
 
AASN
 
 
0.63
 
 
0.61
 
 
0.43
 
 
 
 
DAIM
 
 
0.63
 
 
0.61
 
 
0.53
 
 
 
 
HIPL*
 
 
0.63
 
 
0.60
 
 
0.60
 
 
 
 
DAIP
 
 
0.62
 
 
0.61
 
 
0.49
 
 
 
 
LHEA
 
 
0.61
 
 
0.51
 
 
0.60
 
 
 
 
ALNS
 
 
0.60
 
 
0.60
 
 
0.55
 
 
 
 
CS2A
 
 
0.60
 
 
0.52
 
 
0.53
 
 
 
 
HIPL
 
 
0.60
 
 
0.58
 
 
0.47
 
 
 
 
CS3H
 
 
0.60
 
 
0.48
 
 
0.57
 
 
 
 
CS2H
 
 
0.59
 
 
0.54
 
 
0.34
 
 
 
 
CS4H
 
 
0.59
 
 
0.48
 
 
0.54
 
 
 
 
L3EP
 
 
0.57
 
 
0.54
 
 
0.37
 
 
 
 
OPMR
 
 
0.57
 
 
0.56
 
 
0.57
 
 
 
 
HAML
 
 
0.56
 
 
0.51
 
 
-0.07
 
 
 
 
CS6H
 
 
0.56
 
 
0.52
 
 
0.32
 
 
 
 
HDMP
 
 
0.56
 
 
0.51
 
 
1.00
 
 
 
 
SNOL
 
 
0.54
 
 
0.45
 
 
0.50
 
 
 
 
SNOR
 
 
0.53
 
 
0.44
 
 
0.46
 
 
 
 
OCPR
 
 
0.52
 
 
0.52
 
 
0.51
 
 
 
 
HAPL
 
 
0.52
 
 
0.50
 
 
-0.17
 
 
 
 
HPTH
 
 
0.52
 
 
0.50
 
 
0.46
 
 
 
 
HHPL
 
 
0.50
 
 
0.48
 
 
0.01
 
 
 
 
OPLW
 
 
0.50
 
 
0.49
 
 
-0.19
 
 
 
 
OPHW
 
 
0.50
 
 
0.48
 
 
0.45
 
 
 
 
OMHW
 
 
0.48
 
 
0.48
 
 
0.40
 
 
 
 
PWTP
 
 
0.48
 
 
0.42
 
 
1.00
 
 
 
 
HACP
 
 
0.47
 
 
0.45
 
 
0.47
 
 
 
 
LHEP
 
 
0.47
 
 
0.42
 
 
0.39
 
 
 
 
HIMH
 
 
0.47
 
 
0.43
 
 
0.41
 
 
 
 
OCMR
 
 
0.47
 
 
0.46
 
 
0.47
 
 
 
 
HMTH
 
 
0.46
 
 
0.44
 
 
0.41
 
 
 
 
L4CT
 
 
0.46
 
 
0.46
 
 
0.89
 
 
 
 
DSIL
 
 
0.45
 
 
0.42
 
 
-0.06
 
 
 
 
L4EP
 
 
0.44
 
 
0.43
 
 
0.22
 
 
 
 
OMHL
 
 
0.44
 
 
0.42
 
 
0.39
 
 
 
 
HCTH
 
 
0.44
 
 
0.38
 
 
0.22
 
 
 
 
CS5H
 
 
0.43
 
 
0.40
 
 
0.41
 
 
 
 
PPW6
 
 
0.43
 
 
0.36
 
 
1.00
 
 
 
 
HIPH
 
 
0.43
 
 
0.41
 
 
0.39
 
 
 
 
PPW5
 
 
0.41
 
 
0.34
 
 
1.00
 
 
 
 
OCHW
 
 
0.41
 
 
0.40
 
 
1.00
 
 
 
 
LEPL
 
 
0.40
 
 
0.36
 
 
0.30
 
 
 
 
AANP
 
 
0.40
 
 
0.38
 
 
1.00
 
 
 
 
HICL
 
 
0.40
 
 
0.38
 
 
0.08
 
 
 
 
HICH
 
 
0.39
 
 
0.39
 
 
1.00
 
 
 
 
HICL*
 
 
0.39
 
 
0.34
 
 
0.08
 
 
 
 
OPHL
 
 
0.38
 
 
0.38
 
 
0.19
 
 
 
 
HNAP
 
 
0.37
 
 
0.36
 
 
0.02
 
 
 
 
HCPP
 
 
0.37
 
 
0.34
 
 
1.00
 
 
 
 
HARC
 
 
0.36
 
 
0.34
 
 
0.22
 
 
 
 
OCHL
 
 
0.35
 
 
0.33
 
 
1.00
 
 
 
 
MMPG
 
 
0.32
 
 
0.29
 
 
0.28
 
 
 
 
PPW4
 
 
0.31
 
 
0.30
 
 
1.00
 
 
 
 
HCCC
 
 
0.31
 
 
0.27
 
 
1.00
 
 
 
 
GRPD
 
 
0.30
 
 
0.25
 
 
0.32
 
 
 
 
PPWC
 
 
0.27
 
 
0.25
 
 
NaN
 
 
 
 
HP2C
 
 
0.26
 
 
0.26
 
 
0.93
 
 
 
 
HACL
 
 
0.26
 
 
0.24
 
 
-0.52
 
 
 
 
OCLW
 
 
0.25
 
 
0.29
 
 
-0.46
 
 
 
 
DDAP
 
 
0.24
 
 
0.23
 
 
1.00
 
 
 
 
HCMP
 
 
0.23
 
 
0.25
 
 
0.98
 
 
 
 
DIIL
 
 
0.23
 
 
0.22
 
 
0.18
 
 
 
 
HM2C
 
 
0.23
 
 
0.21
 
 
0.93
 
 
 
 
DMAC
 
 
0.21
 
 
0.20
 
 
0.13
 
 
 
 
HMDC
 
 
0.21
 
 
0.22
 
 
1.00
 
 
 
 
PPW3
 
 
0.19
 
 
0.19
 
 
NaN
 
 
 
 
HARF
 
 
0.16
 
 
0.19
 
 
1.00
 
 
 
 
HCCP
 
 
0.11
 
 
0.10
 
 
-6.16
 
 
 
 
HMSP
 
 
0.11
 
 
0.10
 
 
1.00
 
 
 
 
PPW2
 
 
0.09
 
 
0.11
 
 
NaN
 
 
 
 
HMAC
 
 
0.08
 
 
0.10
 
 
NaN
 
 
 
 
HMTC
 
 
0.07
 
 
0.09
 
 
NaN
 
 
 
 
 It can be seen that while the three measures of agreement tend to
roughly agree,  Krippendorff’s α  has some undesirable properties
on our data in the sense that: 
 
 there is one extreme negative outlier (HCCP with  α  =
-6.16), 
 there are 5 measures where the estimate is missing
( NaN ), and 
 there are several measures where  α  is at (or very close to)
the ceiling of 1.0, while  ICC ( C ,1) and
 agr (GSEM) have much less extreme values (see the scatter plots
above). 
 
 Moreover,  ICC ( C ,1) and  agr (GSEM) are
basically perfectly correlated, but while  ICC ( C ,1) is
a much more general measure of inter-rater agreement,  agr (GSEM)
is specific to our model (which means that it might better account for
its particularities). 
 Focusing on those measure that have different estimates for
 ICC ( C ,1) and  agr (GSEM), we first computed the
“raw” difference Δ agreement = (ICC(C,1) -  agr (GSEM)) and
estimated which of these differences are significant (by comparing the
95% CIs of  ICC ( C ,1) and  agr (GSEM)), as shown
in the table below: 
 
 
  Table 11.    The measures ordered by Δ
agreement = (ICC(C,1) -  agr (GSEM)) and its significance (judged
by comparing the 95%CIs of ICC(C,1) and  agr (GSEM)). 
 
 
 
 
measure
 
 
ICC(C,1)
 
 
95% CI
 
 
 agr (GSEM)
 
 
95% CI
 
 
Δ agreement
 
 
Significant?
 
 
 
 
 
 
SVTv
 
 
0.73
 
 
(0.70, 0.77)
 
 
0.61
 
 
(0.55, 0.66)
 
 
0.13
 
 
TRUE
 
 
 
 
HBC2
 
 
0.78
 
 
(0.74, 0.81)
 
 
0.65
 
 
(0.60, 0.70)
 
 
0.12
 
 
TRUE
 
 
 
 
CS3H
 
 
0.60
 
 
(0.54, 0.64)
 
 
0.48
 
 
(0.41, 0.54)
 
 
0.12
 
 
TRUE
 
 
 
 
MCGD
 
 
0.71
 
 
(0.67, 0.75)
 
 
0.60
 
 
(0.54, 0.65)
 
 
0.11
 
 
TRUE
 
 
 
 
HBNP
 
 
0.84
 
 
(0.81, 0.86)
 
 
0.72
 
 
(0.68, 0.76)
 
 
0.11
 
 
TRUE
 
 
 
 
SVTv*
 
 
0.80
 
 
(0.77, 0.83)
 
 
0.69
 
 
(0.65, 0.73)
 
 
0.11
 
 
TRUE
 
 
 
 
MCGR
 
 
0.68
 
 
(0.64, 0.72)
 
 
0.58
 
 
(0.52, 0.63)
 
 
0.10
 
 
TRUE
 
 
 
 
MCGL
 
 
0.68
 
 
(0.64, 0.72)
 
 
0.58
 
 
(0.52, 0.63)
 
 
0.10
 
 
TRUE
 
 
 
 
SIOD
 
 
0.71
 
 
(0.67, 0.74)
 
 
0.61
 
 
(0.56, 0.66)
 
 
0.10
 
 
TRUE
 
 
 
 
LACT
 
 
0.81
 
 
(0.78, 0.83)
 
 
0.74
 
 
(0.70, 0.77)
 
 
0.07
 
 
TRUE
 
 
 
 
MPGL
 
 
0.84
 
 
(0.82, 0.86)
 
 
0.78
 
 
(0.74, 0.81)
 
 
0.07
 
 
TRUE
 
 
 
 
SNNP
 
 
0.82
 
 
(0.80, 0.85)
 
 
0.76
 
 
(0.72, 0.80)
 
 
0.06
 
 
TRUE
 
 
 
 
SHSW
 
 
0.94
 
 
(0.93, 0.95)
 
 
0.88
 
 
(0.86, 0.90)
 
 
0.06
 
 
TRUE
 
 
 
 
MPGR
 
 
0.84
 
 
(0.81, 0.86)
 
 
0.78
 
 
(0.75, 0.81)
 
 
0.06
 
 
TRUE
 
 
 
 
MIGD
 
 
0.95
 
 
(0.94, 0.95)
 
 
0.89
 
 
(0.87, 0.91)
 
 
0.05
 
 
TRUE
 
 
 
 
SNAP
 
 
0.88
 
 
(0.86, 0.90)
 
 
0.83
 
 
(0.80, 0.86)
 
 
0.05
 
 
TRUE
 
 
 
 
MCMD
 
 
0.92
 
 
(0.90, 0.93)
 
 
0.87
 
 
(0.84, 0.89)
 
 
0.05
 
 
TRUE
 
 
 
 
MCPD
 
 
0.92
 
 
(0.90, 0.93)
 
 
0.87
 
 
(0.85, 0.89)
 
 
0.05
 
 
TRUE
 
 
 
 
SBAN
 
 
0.89
 
 
(0.88, 0.91)
 
 
0.85
 
 
(0.82, 0.87)
 
 
0.05
 
 
TRUE
 
 
 
 
SNAM
 
 
0.90
 
 
(0.89, 0.92)
 
 
0.86
 
 
(0.84, 0.88)
 
 
0.04
 
 
TRUE
 
 
 
 
SHBN
 
 
0.96
 
 
(0.95, 0.96)
 
 
0.94
 
 
(0.93, 0.95)
 
 
0.02
 
 
TRUE
 
 
 
 
MICD
 
 
0.98
 
 
(0.98, 0.99)
 
 
0.97
 
 
(0.96, 0.97)
 
 
0.01
 
 
TRUE
 
 
 
 
CS4H
 
 
0.59
 
 
(0.53, 0.64)
 
 
0.48
 
 
(0.42, 0.54)
 
 
0.11
 
 
FALSE
 
 
 
 
LHEA
 
 
0.61
 
 
(0.56, 0.66)
 
 
0.51
 
 
(0.45, 0.57)
 
 
0.10
 
 
FALSE
 
 
 
 
SNOR
 
 
0.53
 
 
(0.47, 0.58)
 
 
0.44
 
 
(0.38, 0.50)
 
 
0.09
 
 
FALSE
 
 
 
 
SNNA
 
 
0.66
 
 
(0.61, 0.70)
 
 
0.57
 
 
(0.51, 0.62)
 
 
0.09
 
 
FALSE
 
 
 
 
SNOL
 
 
0.54
 
 
(0.48, 0.59)
 
 
0.45
 
 
(0.39, 0.51)
 
 
0.09
 
 
FALSE
 
 
 
 
CS2A
 
 
0.60
 
 
(0.55, 0.65)
 
 
0.52
 
 
(0.46, 0.57)
 
 
0.09
 
 
FALSE
 
 
 
 
LAEP
 
 
0.74
 
 
(0.70, 0.77)
 
 
0.66
 
 
(0.61, 0.70)
 
 
0.08
 
 
FALSE
 
 
 
 
LHCT
 
 
0.66
 
 
(0.62, 0.70)
 
 
0.58
 
 
(0.53, 0.64)
 
 
0.08
 
 
FALSE
 
 
 
 
HBC3
 
 
0.71
 
 
(0.67, 0.74)
 
 
0.63
 
 
(0.58, 0.68)
 
 
0.07
 
 
FALSE
 
 
 
 
PPW5
 
 
0.41
 
 
(0.35, 0.48)
 
 
0.34
 
 
(0.27, 0.41)
 
 
0.07
 
 
FALSE
 
 
 
 
PPW6
 
 
0.43
 
 
(0.37, 0.50)
 
 
0.36
 
 
(0.29, 0.43)
 
 
0.07
 
 
FALSE
 
 
 
 
PWTP
 
 
0.48
 
 
(0.42, 0.54)
 
 
0.42
 
 
(0.35, 0.48)
 
 
0.07
 
 
FALSE
 
 
 
 
PPW7
 
 
0.71
 
 
(0.67, 0.74)
 
 
0.64
 
 
(0.59, 0.69)
 
 
0.06
 
 
FALSE
 
 
 
 
OMLW
 
 
0.70
 
 
(0.65, 0.73)
 
 
0.63
 
 
(0.58, 0.68)
 
 
0.06
 
 
FALSE
 
 
 
 
LNEA
 
 
0.75
 
 
(0.71, 0.78)
 
 
0.69
 
 
(0.64, 0.73)
 
 
0.06
 
 
FALSE
 
 
 
 
HIML
 
 
0.63
 
 
(0.58, 0.67)
 
 
0.57
 
 
(0.51, 0.63)
 
 
0.06
 
 
FALSE
 
 
 
 
HCTH
 
 
0.44
 
 
(0.37, 0.50)
 
 
0.38
 
 
(0.31, 0.45)
 
 
0.06
 
 
FALSE
 
 
 
 
L2EP
 
 
0.67
 
 
(0.62, 0.71)
 
 
0.61
 
 
(0.56, 0.66)
 
 
0.05
 
 
FALSE
 
 
 
 
HAML
 
 
0.56
 
 
(0.50, 0.61)
 
 
0.51
 
 
(0.44, 0.56)
 
 
0.05
 
 
FALSE
 
 
 
 
L2CT
 
 
0.73
 
 
(0.69, 0.76)
 
 
0.68
 
 
(0.63, 0.72)
 
 
0.05
 
 
FALSE
 
 
 
 
LAEA
 
 
0.77
 
 
(0.73, 0.80)
 
 
0.71
 
 
(0.67, 0.75)
 
 
0.05
 
 
FALSE
 
 
 
 
HICL*
 
 
0.39
 
 
(0.32, 0.45)
 
 
0.34
 
 
(0.26, 0.41)
 
 
0.05
 
 
FALSE
 
 
 
 
CS2H
 
 
0.59
 
 
(0.53, 0.64)
 
 
0.54
 
 
(0.48, 0.60)
 
 
0.05
 
 
FALSE
 
 
 
 
LEPL
 
 
0.40
 
 
(0.34, 0.47)
 
 
0.36
 
 
(0.29, 0.43)
 
 
0.05
 
 
FALSE
 
 
 
 
GRPD
 
 
0.30
 
 
(0.22, 0.36)
 
 
0.25
 
 
(0.17, 0.32)
 
 
0.05
 
 
FALSE
 
 
 
 
LHEP
 
 
0.47
 
 
(0.41, 0.53)
 
 
0.42
 
 
(0.36, 0.49)
 
 
0.04
 
 
FALSE
 
 
 
 
L2EA
 
 
0.65
 
 
(0.61, 0.70)
 
 
0.61
 
 
(0.56, 0.66)
 
 
0.04
 
 
FALSE
 
 
 
 
CS6H
 
 
0.56
 
 
(0.50, 0.61)
 
 
0.52
 
 
(0.45, 0.57)
 
 
0.04
 
 
FALSE
 
 
 
 
HDMP
 
 
0.56
 
 
(0.50, 0.61)
 
 
0.51
 
 
(0.45, 0.57)
 
 
0.04
 
 
FALSE
 
 
 
 
MGMD
 
 
0.80
 
 
(0.77, 0.83)
 
 
0.76
 
 
(0.72, 0.79)
 
 
0.04
 
 
FALSE
 
 
 
 
HIML*
 
 
0.65
 
 
(0.60, 0.69)
 
 
0.61
 
 
(0.55, 0.66)
 
 
0.04
 
 
FALSE
 
 
 
 
HNSL
 
 
0.77
 
 
(0.74, 0.80)
 
 
0.73
 
 
(0.69, 0.77)
 
 
0.04
 
 
FALSE
 
 
 
 
HCCC
 
 
0.31
 
 
(0.24, 0.38)
 
 
0.27
 
 
(0.20, 0.35)
 
 
0.04
 
 
FALSE
 
 
 
 
CS5H
 
 
0.43
 
 
(0.37, 0.50)
 
 
0.40
 
 
(0.33, 0.46)
 
 
0.04
 
 
FALSE
 
 
 
 
DSIL
 
 
0.45
 
 
(0.39, 0.51)
 
 
0.42
 
 
(0.35, 0.48)
 
 
0.04
 
 
FALSE
 
 
 
 
DIMD
 
 
0.77
 
 
(0.73, 0.80)
 
 
0.73
 
 
(0.69, 0.77)
 
 
0.04
 
 
FALSE
 
 
 
 
SBAS
 
 
0.79
 
 
(0.76, 0.82)
 
 
0.76
 
 
(0.72, 0.79)
 
 
0.04
 
 
FALSE
 
 
 
 
SSEN*
 
 
0.90
 
 
(0.88, 0.91)
 
 
0.86
 
 
(0.84, 0.88)
 
 
0.04
 
 
FALSE
 
 
 
 
HIMH
 
 
0.47
 
 
(0.41, 0.53)
 
 
0.43
 
 
(0.37, 0.50)
 
 
0.03
 
 
FALSE
 
 
 
 
MGPD
 
 
0.83
 
 
(0.80, 0.85)
 
 
0.80
 
 
(0.76, 0.83)
 
 
0.03
 
 
FALSE
 
 
 
 
L3EP
 
 
0.57
 
 
(0.52, 0.62)
 
 
0.54
 
 
(0.48, 0.60)
 
 
0.03
 
 
FALSE
 
 
 
 
SBNA
 
 
0.90
 
 
(0.88, 0.91)
 
 
0.87
 
 
(0.84, 0.89)
 
 
0.03
 
 
FALSE
 
 
 
 
L3CT
 
 
0.69
 
 
(0.64, 0.73)
 
 
0.66
 
 
(0.61, 0.70)
 
 
0.03
 
 
FALSE
 
 
 
 
SSEN
 
 
0.90
 
 
(0.89, 0.92)
 
 
0.88
 
 
(0.85, 0.90)
 
 
0.03
 
 
FALSE
 
 
 
 
CS7H
 
 
0.69
 
 
(0.64, 0.72)
 
 
0.66
 
 
(0.61, 0.70)
 
 
0.03
 
 
FALSE
 
 
 
 
DICD
 
 
0.71
 
 
(0.67, 0.75)
 
 
0.68
 
 
(0.64, 0.73)
 
 
0.03
 
 
FALSE
 
 
 
 
OCHL
 
 
0.35
 
 
(0.28, 0.42)
 
 
0.33
 
 
(0.25, 0.40)
 
 
0.03
 
 
FALSE
 
 
 
 
GAPD
 
 
0.80
 
 
(0.77, 0.82)
 
 
0.77
 
 
(0.74, 0.80)
 
 
0.03
 
 
FALSE
 
 
 
 
HIPL
 
 
0.60
 
 
(0.55, 0.65)
 
 
0.58
 
 
(0.52, 0.63)
 
 
0.03
 
 
FALSE
 
 
 
 
MMPG
 
 
0.32
 
 
(0.24, 0.39)
 
 
0.29
 
 
(0.22, 0.37)
 
 
0.02
 
 
FALSE
 
 
 
 
HCPP
 
 
0.37
 
 
(0.30, 0.43)
 
 
0.34
 
 
(0.27, 0.41)
 
 
0.02
 
 
FALSE
 
 
 
 
L5CT
 
 
0.71
 
 
(0.67, 0.75)
 
 
0.69
 
 
(0.64, 0.73)
 
 
0.02
 
 
FALSE
 
 
 
 
HBPL
 
 
0.91
 
 
(0.89, 0.92)
 
 
0.88
 
 
(0.86, 0.90)
 
 
0.02
 
 
FALSE
 
 
 
 
HIPL*
 
 
0.63
 
 
(0.58, 0.67)
 
 
0.60
 
 
(0.55, 0.65)
 
 
0.02
 
 
FALSE
 
 
 
 
SPUN
 
 
0.63
 
 
(0.58, 0.67)
 
 
0.61
 
 
(0.55, 0.66)
 
 
0.02
 
 
FALSE
 
 
 
 
HACP
 
 
0.47
 
 
(0.41, 0.53)
 
 
0.45
 
 
(0.38, 0.51)
 
 
0.02
 
 
FALSE
 
 
 
 
AANP
 
 
0.40
 
 
(0.33, 0.47)
 
 
0.38
 
 
(0.31, 0.45)
 
 
0.02
 
 
FALSE
 
 
 
 
HACL
 
 
0.26
 
 
(0.19, 0.33)
 
 
0.24
 
 
(0.17, 0.32)
 
 
0.02
 
 
FALSE
 
 
 
 
PPWC
 
 
0.27
 
 
(0.19, 0.34)
 
 
0.25
 
 
(0.17, 0.32)
 
 
0.02
 
 
FALSE
 
 
 
 
HNAP
 
 
0.37
 
 
(0.30, 0.44)
 
 
0.36
 
 
(0.28, 0.42)
 
 
0.02
 
 
FALSE
 
 
 
 
L4EP
 
 
0.44
 
 
(0.38, 0.50)
 
 
0.43
 
 
(0.36, 0.49)
 
 
0.02
 
 
FALSE
 
 
 
 
HHPL
 
 
0.50
 
 
(0.44, 0.56)
 
 
0.48
 
 
(0.42, 0.54)
 
 
0.02
 
 
FALSE
 
 
 
 
DIPD
 
 
0.80
 
 
(0.77, 0.82)
 
 
0.78
 
 
(0.75, 0.81)
 
 
0.02
 
 
FALSE
 
 
 
 
HBPG
 
 
0.81
 
 
(0.78, 0.83)
 
 
0.79
 
 
(0.76, 0.82)
 
 
0.02
 
 
FALSE
 
 
 
 
OMHL
 
 
0.44
 
 
(0.37, 0.50)
 
 
0.42
 
 
(0.35, 0.49)
 
 
0.02
 
 
FALSE
 
 
 
 
HMTH
 
 
0.46
 
 
(0.40, 0.52)
 
 
0.44
 
 
(0.38, 0.51)
 
 
0.02
 
 
FALSE
 
 
 
 
HAPL
 
 
0.52
 
 
(0.46, 0.57)
 
 
0.50
 
 
(0.44, 0.56)
 
 
0.02
 
 
FALSE
 
 
 
 
SVTh
 
 
0.90
 
 
(0.88, 0.91)
 
 
0.88
 
 
(0.86, 0.90)
 
 
0.02
 
 
FALSE
 
 
 
 
HICL
 
 
0.40
 
 
(0.33, 0.46)
 
 
0.38
 
 
(0.31, 0.45)
 
 
0.02
 
 
FALSE
 
 
 
 
HPTH
 
 
0.52
 
 
(0.46, 0.57)
 
 
0.50
 
 
(0.44, 0.56)
 
 
0.02
 
 
FALSE
 
 
 
 
HIPH
 
 
0.43
 
 
(0.36, 0.49)
 
 
0.41
 
 
(0.34, 0.48)
 
 
0.02
 
 
FALSE
 
 
 
 
SBNP
 
 
0.80
 
 
(0.77, 0.83)
 
 
0.79
 
 
(0.75, 0.82)
 
 
0.01
 
 
FALSE
 
 
 
 
ANSF
 
 
0.79
 
 
(0.75, 0.81)
 
 
0.77
 
 
(0.73, 0.80)
 
 
0.01
 
 
FALSE
 
 
 
 
HM2C
 
 
0.23
 
 
(0.15, 0.30)
 
 
0.21
 
 
(0.14, 0.29)
 
 
0.01
 
 
FALSE
 
 
 
 
HCCP
 
 
0.11
 
 
(0.03, 0.19)
 
 
0.10
 
 
(0.04, 0.18)
 
 
0.01
 
 
FALSE
 
 
 
 
OPHW
 
 
0.50
 
 
(0.43, 0.55)
 
 
0.48
 
 
(0.42, 0.54)
 
 
0.01
 
 
FALSE
 
 
 
 
AASN
 
 
0.63
 
 
(0.58, 0.67)
 
 
0.61
 
 
(0.56, 0.66)
 
 
0.01
 
 
FALSE
 
 
 
 
HARC
 
 
0.36
 
 
(0.29, 0.42)
 
 
0.34
 
 
(0.27, 0.41)
 
 
0.01
 
 
FALSE
 
 
 
 
DAIM
 
 
0.63
 
 
(0.58, 0.67)
 
 
0.61
 
 
(0.56, 0.66)
 
 
0.01
 
 
FALSE
 
 
 
 
OPLW
 
 
0.50
 
 
(0.44, 0.56)
 
 
0.49
 
 
(0.42, 0.55)
 
 
0.01
 
 
FALSE
 
 
 
 
SSEG
 
 
0.90
 
 
(0.88, 0.91)
 
 
0.88
 
 
(0.86, 0.90)
 
 
0.01
 
 
FALSE
 
 
 
 
SVTh*
 
 
0.93
 
 
(0.91, 0.94)
 
 
0.91
 
 
(0.90, 0.93)
 
 
0.01
 
 
FALSE
 
 
 
 
DAIP
 
 
0.62
 
 
(0.57, 0.67)
 
 
0.61
 
 
(0.56, 0.66)
 
 
0.01
 
 
FALSE
 
 
 
 
OPMR
 
 
0.57
 
 
(0.52, 0.62)
 
 
0.56
 
 
(0.50, 0.61)
 
 
0.01
 
 
FALSE
 
 
 
 
L5EA
 
 
0.74
 
 
(0.70, 0.77)
 
 
0.72
 
 
(0.68, 0.76)
 
 
0.01
 
 
FALSE
 
 
 
 
PPW4
 
 
0.31
 
 
(0.24, 0.38)
 
 
0.30
 
 
(0.23, 0.37)
 
 
0.01
 
 
FALSE
 
 
 
 
APNS
 
 
0.83
 
 
(0.80, 0.85)
 
 
0.82
 
 
(0.79, 0.84)
 
 
0.01
 
 
FALSE
 
 
 
 
DDAP
 
 
0.24
 
 
(0.17, 0.32)
 
 
0.23
 
 
(0.16, 0.31)
 
 
0.01
 
 
FALSE
 
 
 
 
ASNP
 
 
0.72
 
 
(0.68, 0.76)
 
 
0.71
 
 
(0.67, 0.75)
 
 
0.01
 
 
FALSE
 
 
 
 
L5EP
 
 
0.66
 
 
(0.61, 0.70)
 
 
0.65
 
 
(0.60, 0.70)
 
 
0.01
 
 
FALSE
 
 
 
 
DMAC
 
 
0.21
 
 
(0.13, 0.28)
 
 
0.20
 
 
(0.12, 0.27)
 
 
0.01
 
 
FALSE
 
 
 
 
OCHW
 
 
0.41
 
 
(0.34, 0.47)
 
 
0.40
 
 
(0.33, 0.46)
 
 
0.01
 
 
FALSE
 
 
 
 
HP2C
 
 
0.26
 
 
(0.19, 0.34)
 
 
0.26
 
 
(0.18, 0.33)
 
 
0.01
 
 
FALSE
 
 
 
 
L4EA
 
 
0.76
 
 
(0.72, 0.79)
 
 
0.75
 
 
(0.71, 0.78)
 
 
0.01
 
 
FALSE
 
 
 
 
HMSP
 
 
0.11
 
 
(0.03, 0.19)
 
 
0.10
 
 
(0.04, 0.18)
 
 
0.01
 
 
FALSE
 
 
 
 
OCMR
 
 
0.47
 
 
(0.40, 0.53)
 
 
0.46
 
 
(0.40, 0.52)
 
 
0.01
 
 
FALSE
 
 
 
 
ALNS
 
 
0.60
 
 
(0.55, 0.65)
 
 
0.60
 
 
(0.54, 0.65)
 
 
0.01
 
 
FALSE
 
 
 
 
MCGP
 
 
0.87
 
 
(0.84, 0.88)
 
 
0.86
 
 
(0.83, 0.88)
 
 
0.01
 
 
FALSE
 
 
 
 
HICH
 
 
0.39
 
 
(0.32, 0.46)
 
 
0.39
 
 
(0.32, 0.45)
 
 
0.01
 
 
FALSE
 
 
 
 
ACSN
 
 
0.87
 
 
(0.85, 0.89)
 
 
0.86
 
 
(0.84, 0.88)
 
 
0.00
 
 
FALSE
 
 
 
 
ANNF
 
 
0.74
 
 
(0.71, 0.78)
 
 
0.74
 
 
(0.70, 0.78)
 
 
0.00
 
 
FALSE
 
 
 
 
MCGM
 
 
0.89
 
 
(0.87, 0.90)
 
 
0.88
 
 
(0.86, 0.90)
 
 
0.00
 
 
FALSE
 
 
 
 
DIIL
 
 
0.23
 
 
(0.15, 0.30)
 
 
0.22
 
 
(0.15, 0.30)
 
 
0.00
 
 
FALSE
 
 
 
 
APSN
 
 
0.73
 
 
(0.69, 0.76)
 
 
0.72
 
 
(0.68, 0.76)
 
 
0.00
 
 
FALSE
 
 
 
 
L3EA
 
 
0.76
 
 
(0.72, 0.79)
 
 
0.75
 
 
(0.72, 0.79)
 
 
0.00
 
 
FALSE
 
 
 
 
HBC4
 
 
0.79
 
 
(0.76, 0.82)
 
 
0.79
 
 
(0.76, 0.82)
 
 
0.00
 
 
FALSE
 
 
 
 
OCPR
 
 
0.52
 
 
(0.46, 0.57)
 
 
0.52
 
 
(0.46, 0.57)
 
 
0.00
 
 
FALSE
 
 
 
 
OMHW
 
 
0.48
 
 
(0.42, 0.54)
 
 
0.48
 
 
(0.42, 0.54)
 
 
0.00
 
 
FALSE
 
 
 
 
DAIS
 
 
0.65
 
 
(0.60, 0.69)
 
 
0.65
 
 
(0.60, 0.69)
 
 
0.00
 
 
FALSE
 
 
 
 
ABSN
 
 
0.82
 
 
(0.79, 0.84)
 
 
0.82
 
 
(0.79, 0.85)
 
 
0.00
 
 
FALSE
 
 
 
 
APNP
 
 
0.76
 
 
(0.73, 0.79)
 
 
0.76
 
 
(0.73, 0.80)
 
 
0.00
 
 
FALSE
 
 
 
 
PPW3
 
 
0.19
 
 
(0.12, 0.27)
 
 
0.19
 
 
(0.12, 0.27)
 
 
0.00
 
 
FALSE
 
 
 
 
OPHL
 
 
0.38
 
 
(0.31, 0.44)
 
 
0.38
 
 
(0.31, 0.45)
 
 
0.00
 
 
FALSE
 
 
 
 
PNNP
 
 
0.74
 
 
(0.70, 0.77)
 
 
0.74
 
 
(0.70, 0.78)
 
 
0.00
 
 
FALSE
 
 
 
 
L4CT
 
 
0.46
 
 
(0.39, 0.52)
 
 
0.46
 
 
(0.40, 0.52)
 
 
-0.01
 
 
FALSE
 
 
 
 
DAII
 
 
0.66
 
 
(0.61, 0.70)
 
 
0.66
 
 
(0.62, 0.71)
 
 
-0.01
 
 
FALSE
 
 
 
 
ASCG*
 
 
0.87
 
 
(0.85, 0.89)
 
 
0.88
 
 
(0.86, 0.90)
 
 
-0.01
 
 
FALSE
 
 
 
 
HMDC
 
 
0.21
 
 
(0.13, 0.28)
 
 
0.22
 
 
(0.14, 0.29)
 
 
-0.01
 
 
FALSE
 
 
 
 
HMAC
 
 
0.08
 
 
(0.01, 0.16)
 
 
0.10
 
 
(0.02, 0.18)
 
 
-0.01
 
 
FALSE
 
 
 
 
PPW2
 
 
0.09
 
 
(0.01, 0.17)
 
 
0.11
 
 
(0.03, 0.18)
 
 
-0.01
 
 
FALSE
 
 
 
 
HMTC
 
 
0.07
 
 
(-0.01, 0.15)
 
 
0.09
 
 
(0.03, 0.17)
 
 
-0.02
 
 
FALSE
 
 
 
 
HCMP
 
 
0.23
 
 
(0.15, 0.30)
 
 
0.25
 
 
(0.17, 0.33)
 
 
-0.02
 
 
FALSE
 
 
 
 
HARF
 
 
0.16
 
 
(0.08, 0.23)
 
 
0.19
 
 
(0.11, 0.26)
 
 
-0.03
 
 
FALSE
 
 
 
 
OCLW
 
 
0.25
 
 
(0.17, 0.32)
 
 
0.29
 
 
(0.21, 0.36)
 
 
-0.04
 
 
FALSE
 
 
 
 
 However, this tends to overestimate the number of disagreements if
the two estimates of inter-rater agreement are highly correlated but
systematically different (i.e., if there is a linear relationship
between them with a non-zero intercept). Thus, we regressed linearly the
one on the other (i.e., we performed two linear regressions, once
 ICC ( C ,1) on  agr (GSEM), and separately
 agr (GSEM) on  ICC ( C ,1)) and we obtained that
both are highly significant, with a very high adjusted R 2  =
97.9%, intercepts  α =0.02 ( p =0.0209) and -0.00
( p =0.509), and slopes 1.03 ( p =3.24e-122) and 0.95
( p =3.24e-122), respectively, strongly suggesting an almost
perfect identity relationship between them. 
   ICC ( C ,1) on
 agr (GSEM):  
 
 
 
 
 
 
 
 
 
 
   
 Estimate 
 Std. Error 
 t value 
 Pr(>|t|) 
 
 
 
 
  (Intercept)  
 0.01792 
 0.007672 
 2.335 
 0.02091 
 
 
  rreliability  
 1.026 
 0.01262 
 81.29 
 3.241e-122 
 
 
 
 
 Fitting linear model: ICCc1 ~ rreliability 
 
 
 
 
 
 
 
 
 Observations 
 Residual Std. Error 
  \(R^2\)  
 Adjusted  \(R^2\)  
 
 
 
 
 146 
 0.03314 
 0.9787 
 0.9785 
 
 
 
 
 
 
  Figure 12.    Outliers and influential
observations for the linear regression of ICC(C,1) on
 agr (GSEM). Showing the top 10. 
 
 
   agr (GSEM) on
 ICC ( C ,1):  
 
 
 
 
 
 
 
 
 
 
   
 Estimate 
 Std. Error 
 t value 
 Pr(>|t|) 
 
 
 
 
  (Intercept)  
 -0.004982 
 0.007523 
 -0.6622 
 0.5089 
 
 
  ICCc1  
 0.9537 
 0.01173 
 81.29 
 3.241e-122 
 
 
 
 
 Fitting linear model: rreliability ~ ICCc1 
 
 
 
 
 
 
 
 
 Observations 
 Residual Std. Error 
  \(R^2\)  
 Adjusted  \(R^2\)  
 
 
 
 
 146 
 0.03195 
 0.9787 
 0.9785 
 
 
 
 
 
 
  Figure 13.    Outliers and influential
observations for the linear regression of  agr (GSEM) on
ICC(C,1). Showing the top 10. 
 
 
 The only possible outlier (as identified by
 outlierTest() ) is SVTv. 
 Given all these, it seems  ICC(C,1)  and  agr (GSEM)
are the best measures of agreement for us, and are virtually
identical. 
 
 
 By domain 
 Including ANOVA and the significant pair-wise differences after
Tukey’s HSD: 
 
 
 
  Figure 14.    Inter-rater agreements by
domain. 
 
 
 
 Krippendorff’s α 
 
 Analysis of Variance Model 
 
 
 
 
 
 
 
 
 
 
   
 Df 
 Sum Sq 
 Mean Sq 
 F value 
 Pr(>F) 
 
 
 
 
  domain  
 10 
 4.585 
 0.4585 
 1.085 
 0.3787 
 
 
  Residuals  
 130 
 54.96 
 0.4227 
 NA 
 NA 
 
 
 
 
 
 ICC(C,1) 
 
 
 
 
 
 
 
 
 
 
 
   
 Df 
 Sum Sq 
 Mean Sq 
 F value 
 Pr(>F) 
 
 
 
 
  domain  
 10 
 3.644 
 0.3644 
 13.05 
 9.343e-16 
 
 
  Residuals  
 135 
 3.77 
 0.02793 
 NA 
 NA 
 
 
 
Table: Analysis of Variance Model
 
 
  Table 12.    Pairwise significant comparisons
(Tukey’s HSD) 
 
 
 
 
 
 
diff
 
 
lwr
 
 
upr
 
 
p.adj
 
 
 
 
 
 
pharynx-mandible
 
 
-0.40
 
 
-0.63
 
 
-0.16
 
 
0.00001
 
 
 
 
hard palate-mandible
 
 
-0.39
 
 
-0.57
 
 
-0.22
 
 
0.00000
 
 
 
 
pharynx-hyoid
 
 
-0.38
 
 
-0.68
 
 
-0.07
 
 
0.00370
 
 
 
 
hard palate-hyoid
 
 
-0.38
 
 
-0.64
 
 
-0.12
 
 
0.00031
 
 
 
 
pharynx-skull
 
 
-0.37
 
 
-0.58
 
 
-0.16
 
 
0.00000
 
 
 
 
hard palate-skull
 
 
-0.37
 
 
-0.51
 
 
-0.23
 
 
0.00000
 
 
 
 
pharynx-general
 
 
-0.34
 
 
-0.63
 
 
-0.05
 
 
0.00849
 
 
 
 
oral-mandible
 
 
-0.34
 
 
-0.55
 
 
-0.12
 
 
0.00005
 
 
 
 
hard palate-general
 
 
-0.34
 
 
-0.58
 
 
-0.09
 
 
0.00064
 
 
 
 
oral-hyoid
 
 
-0.32
 
 
-0.61
 
 
-0.03
 
 
0.01814
 
 
 
 
oral-skull
 
 
-0.31
 
 
-0.50
 
 
-0.13
 
 
0.00001
 
 
 
 
oral-general
 
 
-0.28
 
 
-0.55
 
 
-0.01
 
 
0.04098
 
 
 
 
dentition-mandible
 
 
-0.26
 
 
-0.48
 
 
-0.04
 
 
0.00822
 
 
 
 
pharynx-larynx
 
 
-0.25
 
 
-0.47
 
 
-0.03
 
 
0.01405
 
 
 
 
hard palate-larynx
 
 
-0.25
 
 
-0.40
 
 
-0.09
 
 
0.00004
 
 
 
 
dentition-skull
 
 
-0.24
 
 
-0.43
 
 
-0.04
 
 
0.00542
 
 
 
 
 
 
 agr(GSEM) 
 
 
 
 
 
 
 
 
 
 
 
   
 Df 
 Sum Sq 
 Mean Sq 
 F value 
 Pr(>F) 
 
 
 
 
  domain  
 10 
 3.207 
 0.3207 
 11.75 
 2.19e-14 
 
 
  Residuals  
 135 
 3.683 
 0.02728 
 NA 
 NA 
 
 
 
Table: Analysis of Variance Model
 
 
  Table 13.    Pairwise significant comparisons
(Tukey’s HSD) 
 
 
 
 
 
 
diff
 
 
lwr
 
 
upr
 
 
p.adj
 
 
 
 
 
 
pharynx-mandible
 
 
-0.38
 
 
-0.61
 
 
-0.15
 
 
0.00002
 
 
 
 
pharynx-skull
 
 
-0.37
 
 
-0.58
 
 
-0.17
 
 
0.00000
 
 
 
 
hard palate-mandible
 
 
-0.36
 
 
-0.54
 
 
-0.19
 
 
0.00000
 
 
 
 
hard palate-skull
 
 
-0.36
 
 
-0.50
 
 
-0.22
 
 
0.00000
 
 
 
 
pharynx-hyoid
 
 
-0.35
 
 
-0.65
 
 
-0.04
 
 
0.01150
 
 
 
 
hard palate-hyoid
 
 
-0.33
 
 
-0.59
 
 
-0.07
 
 
0.00254
 
 
 
 
pharynx-general
 
 
-0.31
 
 
-0.60
 
 
-0.03
 
 
0.01868
 
 
 
 
hard palate-general
 
 
-0.30
 
 
-0.54
 
 
-0.06
 
 
0.00362
 
 
 
 
oral-mandible
 
 
-0.30
 
 
-0.51
 
 
-0.08
 
 
0.00055
 
 
 
 
oral-skull
 
 
-0.29
 
 
-0.48
 
 
-0.11
 
 
0.00005
 
 
 
 
pharynx-larynx
 
 
-0.24
 
 
-0.46
 
 
-0.02
 
 
0.01868
 
 
 
 
cervical-skull
 
 
-0.23
 
 
-0.46
 
 
-0.01
 
 
0.03956
 
 
 
 
hard palate-larynx
 
 
-0.22
 
 
-0.38
 
 
-0.07
 
 
0.00022
 
 
 
 
dentition-mandible
 
 
-0.22
 
 
-0.44
 
 
0.00
 
 
0.04074
 
 
 
 
dentition-skull
 
 
-0.22
 
 
-0.41
 
 
-0.03
 
 
0.01260
 
 
 
 
 
 
 
 By type 
 Including ANOVA and the significant pair-wise differences after
Tukey’s HSD: 
 
 
 
  Figure 15.    Inter-rater agreement by
type. 
 
 
 
 Krippendorff’s α 
 
 Analysis of Variance Model 
 
 
 
 
 
 
 
 
 
 
   
 Df 
 Sum Sq 
 Mean Sq 
 F value 
 Pr(>F) 
 
 
 
 
  type  
 4 
 3.906 
 0.9764 
 2.387 
 0.05414 
 
 
  Residuals  
 136 
 55.63 
 0.4091 
 NA 
 NA 
 
 
 
 
 
 ICC(C,1) 
 
 
 
 
 
 
 
 
 
 
 
   
 Df 
 Sum Sq 
 Mean Sq 
 F value 
 Pr(>F) 
 
 
 
 
  type  
 4 
 2.648 
 0.662 
 19.59 
 7.744e-13 
 
 
  Residuals  
 141 
 4.766 
 0.0338 
 NA 
 NA 
 
 
 
Table: Analysis of Variance Model
 
 
  Table 14.    Pairwise significant comparisons
(Tukey’s HSD) 
 
 
 
 
 
 
diff
 
 
lwr
 
 
upr
 
 
p.adj
 
 
 
 
 
 
curvature-distance
 
 
-0.39
 
 
-0.54
 
 
-0.24
 
 
0.00000
 
 
 
 
curvature-angle
 
 
-0.38
 
 
-0.56
 
 
-0.20
 
 
0.00000
 
 
 
 
Procrustes dist.-distance
 
 
-0.34
 
 
-0.53
 
 
-0.15
 
 
0.00001
 
 
 
 
Procrustes dist.-angle
 
 
-0.33
 
 
-0.54
 
 
-0.12
 
 
0.00020
 
 
 
 
ratio-distance
 
 
-0.21
 
 
-0.36
 
 
-0.05
 
 
0.00284
 
 
 
 
ratio-angle
 
 
-0.20
 
 
-0.38
 
 
-0.02
 
 
0.02309
 
 
 
 
 
 
 agr(GSEM) 
 
 
 
 
 
 
 
 
 
 
 
   
 Df 
 Sum Sq 
 Mean Sq 
 F value 
 Pr(>F) 
 
 
 
 
  type  
 4 
 2.378 
 0.5946 
 18.58 
 2.761e-12 
 
 
  Residuals  
 141 
 4.512 
 0.032 
 NA 
 NA 
 
 
 
Table: Analysis of Variance Model
 
 
  Table 15.    Pairwise significant comparisons
(Tukey’s HSD) 
 
 
 
 
 
 
diff
 
 
lwr
 
 
upr
 
 
p.adj
 
 
 
 
 
 
curvature-angle
 
 
-0.39
 
 
-0.56
 
 
-0.22
 
 
0.00000
 
 
 
 
curvature-distance
 
 
-0.36
 
 
-0.51
 
 
-0.22
 
 
0.00000
 
 
 
 
Procrustes dist.-angle
 
 
-0.35
 
 
-0.55
 
 
-0.14
 
 
0.00006
 
 
 
 
Procrustes dist.-distance
 
 
-0.32
 
 
-0.50
 
 
-0.14
 
 
0.00003
 
 
 
 
ratio-angle
 
 
-0.20
 
 
-0.38
 
 
-0.03
 
 
0.01583
 
 
 
 
ratio-distance
 
 
-0.17
 
 
-0.33
 
 
-0.02
 
 
0.01590
 
 
 
 
 
 
 
 Versus twin correlations 
 
 “Raw” twin correlations 
 
 
 
  Figure 16.    Inter-rater agreement vs raw
twin correlations. 
 
 
 
 
 Corrected twin correlations 
 
 
 
  Figure 17.    Inter-rater agreement vs
corrected twin correlations. 
 
 
 
 
 
 Versus best model selected 
 Including ANOVA and the significant pair-wise differences after
Tukey’s HSD: 
 
 
 
  Figure 18.    Inter-rater agreement by the
best genetic model selected. 
 
 
 
 Krippendorff’s α 
 
 Analysis of Variance Model 
 
 
 
 
 
 
 
 
 
 
   
 Df 
 Sum Sq 
 Mean Sq 
 F value 
 Pr(>F) 
 
 
 
 
  genetic_model  
 1 
 0.4445 
 0.4445 
 1.045 
 0.3083 
 
 
  Residuals  
 139 
 59.1 
 0.4252 
 NA 
 NA 
 
 
 
 
 
 ICC(C,1) 
 
 Analysis of Variance Model 
 
 
 
 
 
 
 
 
 
 
   
 Df 
 Sum Sq 
 Mean Sq 
 F value 
 Pr(>F) 
 
 
 
 
  genetic_model  
 1 
 0.05053 
 0.05053 
 0.9881 
 0.3219 
 
 
  Residuals  
 144 
 7.363 
 0.05114 
 NA 
 NA 
 
 
 
 
 
 agr(GSEM) 
 
 Analysis of Variance Model 
 
 
 
 
 
 
 
 
 
 
   
 Df 
 Sum Sq 
 Mean Sq 
 F value 
 Pr(>F) 
 
 
 
 
  genetic_model  
 1 
 0.01947 
 0.01947 
 0.408 
 0.524 
 
 
  Residuals  
 144 
 6.871 
 0.04772 
 NA 
 NA 
 
 
 
 
 
 
 Versus  A ,  C  or  D , and  E 
estimates 
 
 
 
  Figure 19.    Inter-rater agreement vs
 A ,  C  or  D , and  E  estimates. Showing
the Pearson’s correlations and linear regression lines (solid if the
corresponding correlation is significant, dotted otherwise). 
 
 
 
 
 Versus narrow-sense heritability ( h  2 ),
broad-sense heritability ( H  2 ) or familiality
( F  2 ), and environmental variance
( e  2 ) 
 
 
 
  Figure 20.    Inter-rater agreement vs
 h  2 ,  H  2  or
 F  2 , and  e  2  estimates. Showing
the Pearson’s correlations and linear regression lines (solid if the
corresponding correlation is significant, dotted otherwise). 
 
 
 
 
 Summary for inter-rater agreement 
  First , which measure of inter-rater agreement should
we use?  Krippendorff’s α  seems to have estimation issues for
some measures ( NaN  for 5 and 1 extremely negative value of
-6.16), and seems to be at ceiling for most (at or very close to 1.0),
while  ICC ( C ,1) and  agr (GSEM) are always
estimated (quite robustly) and show much more inter-measure variation.
Moreover,  ICC ( C ,1) and  agr (GSEM) correlate
very well (Pearson’s  r =0.99,  p =3.2e-122), but much
less so with  Krippendorff’s α  ( ICC ( C ,1):
Pearson’s  r =0.34,  p =4.8e-05;  agr (GSEM):
Pearson’s  r =0.34,  p =4.6e-05), which is in good part
due to those measures at ceiling for  α  but not for the other
two. Therefore, we will use   ICC ( C ,1) 
and   agr (GSEM)  as measures of inter-rather
agreement. 
  Second , a higher  ICC ( C ,1): 
 
  is reached for the  mandible , “ general ”,
 hyoid  and  skull  (while the oral, hard palate, and
pharynx have the lowest agreement);  
  is reached for  distances  and  angles  (while the
lowest is for Procrustes distances and curvatures);  
  is  positively and significantly correlated  with higher
MZ (very strongly) and DZ (moderately) twin correlations;  
  does not differ between the ACE and ADE models;  
  is  positively and significantly correlated  with all
unstandardized genetic components, particularly so with the additive and
dominant genetic variances A and D,  
  is  correlated (positively)  only with the narrow-sense
heritability,  h  2 .  
 
 A higher  agr (GSEM) has pretty much the same properties as
 ICC ( C ,1): 
 
  is reached for the  mandible , “ general ”,
 hyoid  and  skull  (while the oral, hard palate, and
pharynx have the lowest agreement);  
  is reached for  distances  and  angles  (while the
lowest is for Procrustes distances and curvatures);  
  is  positively and significantly correlated  with higher
MZ (very strongly) but not at all with DZ twin correlations;  
  does not differ between the ACE and ADE models;  
  is  positively and significantly correlated  with all
unstandardized genetic components, particularly so with the additive and
dominant genetic variances A and D,  
  is  correlated (positively)  only with the narrow-sense
heritability,  h  2 .  
 
 Given the general nature of  ICC ( C ,1), we will use
it as our measure of inter-rater agreement, keeping in mind that
 agr (GSEM) is virtually identical and may better account for the
particularities of our design, but might be more sensitive to the
unbalanced number of MZ and DZ twins in our data as well. 
  Third , following guidelines for the interpretation
of  ICC ( C ,1) and using 0.75 as a cut-off point, there
are 44 measures with  ICC ( C ,1) >= 0.75, and 37 with
the lower bound of the 95% CI of the  ICC ( C ,1) >=
0.75: 
 
 
  Table 16.    Measures with good intra-rates
agreement. 95% CI is the 95% confidence interval of
 ICC ( C ,1), and 95% CI >= 0.75 means that this 95% CI
is located above the 0.75 threshold. We also show for these the
corresponding  agr (GSEM). 
 
 
 
 
measure
 
 
domain
 
 
type
 
 
ICC(C,1)
 
 
95% CI
 
 
ICC(C,1) >= 0.75
 
 
95% CI >= 0.75
 
 
agr(GSEM)
 
 
95% CI
 
 
agr(GSEM) >= 0.75
 
 
95% CI >= 0.75
 
 
 
 
 
 
MICD
 
 
mandible
 
 
distance
 
 
0.983
 
 
[0.980, 0.985]
 
 
Yes
 
 
Yes
 
 
0.969
 
 
[0.963, 0.974]
 
 
Yes
 
 
Yes
 
 
 
 
SHBN
 
 
skull
 
 
distance
 
 
0.959
 
 
[0.952, 0.965]
 
 
Yes
 
 
Yes
 
 
0.938
 
 
[0.926, 0.948]
 
 
Yes
 
 
Yes
 
 
 
 
MIGD
 
 
mandible
 
 
distance
 
 
0.946
 
 
[0.937, 0.954]
 
 
Yes
 
 
Yes
 
 
0.894
 
 
[0.875, 0.910]
 
 
Yes
 
 
Yes
 
 
 
 
SHSW
 
 
skull
 
 
distance
 
 
0.941
 
 
[0.931, 0.949]
 
 
Yes
 
 
Yes
 
 
0.879
 
 
[0.858, 0.898]
 
 
Yes
 
 
Yes
 
 
 
 
SVTh*
 
 
general
 
 
distance
 
 
0.926
 
 
[0.914, 0.936]
 
 
Yes
 
 
Yes
 
 
0.913
 
 
[0.897, 0.926]
 
 
Yes
 
 
Yes
 
 
 
 
MCPD
 
 
mandible
 
 
distance
 
 
0.918
 
 
[0.905, 0.930]
 
 
Yes
 
 
Yes
 
 
0.868
 
 
[0.846, 0.888]
 
 
Yes
 
 
Yes
 
 
 
 
MCMD
 
 
mandible
 
 
distance
 
 
0.916
 
 
[0.902, 0.927]
 
 
Yes
 
 
Yes
 
 
0.866
 
 
[0.843, 0.886]
 
 
Yes
 
 
Yes
 
 
 
 
HBPL
 
 
hard palate
 
 
distance
 
 
0.907
 
 
[0.892, 0.920]
 
 
Yes
 
 
Yes
 
 
0.885
 
 
[0.864, 0.903]
 
 
Yes
 
 
Yes
 
 
 
 
SSEN
 
 
skull
 
 
distance
 
 
0.905
 
 
[0.889, 0.918]
 
 
Yes
 
 
Yes
 
 
0.877
 
 
[0.855, 0.896]
 
 
Yes
 
 
Yes
 
 
 
 
SNAM
 
 
skull
 
 
distance
 
 
0.902
 
 
[0.886, 0.915]
 
 
Yes
 
 
Yes
 
 
0.859
 
 
[0.835, 0.880]
 
 
Yes
 
 
Yes
 
 
 
 
SBNA
 
 
skull
 
 
distance
 
 
0.898
 
 
[0.881, 0.912]
 
 
Yes
 
 
Yes
 
 
0.867
 
 
[0.844, 0.888]
 
 
Yes
 
 
Yes
 
 
 
 
SVTh
 
 
general
 
 
distance
 
 
0.897
 
 
[0.881, 0.912]
 
 
Yes
 
 
Yes
 
 
0.881
 
 
[0.860, 0.899]
 
 
Yes
 
 
Yes
 
 
 
 
SSEN*
 
 
skull
 
 
distance
 
 
0.896
 
 
[0.880, 0.911]
 
 
Yes
 
 
Yes
 
 
0.860
 
 
[0.836, 0.881]
 
 
Yes
 
 
Yes
 
 
 
 
SSEG
 
 
skull
 
 
distance
 
 
0.895
 
 
[0.879, 0.910]
 
 
Yes
 
 
Yes
 
 
0.882
 
 
[0.862, 0.900]
 
 
Yes
 
 
Yes
 
 
 
 
SBAN
 
 
skull
 
 
distance
 
 
0.894
 
 
[0.877, 0.909]
 
 
Yes
 
 
Yes
 
 
0.848
 
 
[0.821, 0.871]
 
 
Yes
 
 
Yes
 
 
 
 
MCGM
 
 
mandible
 
 
angle
 
 
0.885
 
 
[0.867, 0.901]
 
 
Yes
 
 
Yes
 
 
0.881
 
 
[0.860, 0.899]
 
 
Yes
 
 
Yes
 
 
 
 
SNAP
 
 
skull
 
 
distance
 
 
0.880
 
 
[0.861, 0.897]
 
 
Yes
 
 
Yes
 
 
0.830
 
 
[0.801, 0.855]
 
 
Yes
 
 
Yes
 
 
 
 
ASCG*
 
 
skull
 
 
angle
 
 
0.873
 
 
[0.852, 0.890]
 
 
Yes
 
 
Yes
 
 
0.881
 
 
[0.860, 0.899]
 
 
Yes
 
 
Yes
 
 
 
 
ACSN
 
 
skull
 
 
angle
 
 
0.867
 
 
[0.847, 0.886]
 
 
Yes
 
 
Yes
 
 
0.863
 
 
[0.839, 0.883]
 
 
Yes
 
 
Yes
 
 
 
 
MCGP
 
 
mandible
 
 
angle
 
 
0.865
 
 
[0.844, 0.884]
 
 
Yes
 
 
Yes
 
 
0.859
 
 
[0.835, 0.880]
 
 
Yes
 
 
Yes
 
 
 
 
MPGL
 
 
mandible
 
 
distance
 
 
0.843
 
 
[0.819, 0.864]
 
 
Yes
 
 
Yes
 
 
0.778
 
 
[0.742, 0.809]
 
 
Yes
 
 
No
 
 
 
 
MPGR
 
 
mandible
 
 
distance
 
 
0.840
 
 
[0.815, 0.861]
 
 
Yes
 
 
Yes
 
 
0.782
 
 
[0.746, 0.813]
 
 
Yes
 
 
No
 
 
 
 
HBNP
 
 
hyoid
 
 
distance
 
 
0.835
 
 
[0.810, 0.857]
 
 
Yes
 
 
Yes
 
 
0.724
 
 
[0.681, 0.761]
 
 
No
 
 
No
 
 
 
 
MGPD
 
 
mandible
 
 
distance
 
 
0.831
 
 
[0.805, 0.853]
 
 
Yes
 
 
Yes
 
 
0.797
 
 
[0.764, 0.827]
 
 
Yes
 
 
Yes
 
 
 
 
APNS
 
 
skull
 
 
angle
 
 
0.826
 
 
[0.800, 0.850]
 
 
Yes
 
 
Yes
 
 
0.815
 
 
[0.785, 0.842]
 
 
Yes
 
 
Yes
 
 
 
 
SNNP
 
 
skull
 
 
distance
 
 
0.824
 
 
[0.797, 0.848]
 
 
Yes
 
 
Yes
 
 
0.760
 
 
[0.721, 0.795]
 
 
Yes
 
 
No
 
 
 
 
ABSN
 
 
skull
 
 
angle
 
 
0.819
 
 
[0.792, 0.843]
 
 
Yes
 
 
Yes
 
 
0.819
 
 
[0.789, 0.845]
 
 
Yes
 
 
Yes
 
 
 
 
LACT
 
 
larynx
 
 
distance
 
 
0.808
 
 
[0.779, 0.833]
 
 
Yes
 
 
Yes
 
 
0.737
 
 
[0.696, 0.773]
 
 
No
 
 
No
 
 
 
 
HBPG
 
 
hyoid
 
 
distance
 
 
0.807
 
 
[0.778, 0.833]
 
 
Yes
 
 
Yes
 
 
0.790
 
 
[0.757, 0.819]
 
 
Yes
 
 
Yes
 
 
 
 
SVTv*
 
 
general
 
 
distance
 
 
0.804
 
 
[0.774, 0.830]
 
 
Yes
 
 
Yes
 
 
0.692
 
 
[0.647, 0.733]
 
 
No
 
 
No
 
 
 
 
SBNP
 
 
skull
 
 
distance
 
 
0.801
 
 
[0.771, 0.827]
 
 
Yes
 
 
Yes
 
 
0.786
 
 
[0.752, 0.817]
 
 
Yes
 
 
Yes
 
 
 
 
MGMD
 
 
mandible
 
 
distance
 
 
0.800
 
 
[0.769, 0.826]
 
 
Yes
 
 
Yes
 
 
0.759
 
 
[0.720, 0.793]
 
 
Yes
 
 
No
 
 
 
 
GAPD
 
 
general
 
 
Procrustes dist.
 
 
0.798
 
 
[0.767, 0.825]
 
 
Yes
 
 
Yes
 
 
0.772
 
 
[0.736, 0.805]
 
 
Yes
 
 
No
 
 
 
 
DIPD
 
 
dentition
 
 
distance
 
 
0.798
 
 
[0.767, 0.824]
 
 
Yes
 
 
Yes
 
 
0.780
 
 
[0.745, 0.810]
 
 
Yes
 
 
No
 
 
 
 
HBC4
 
 
hyoid
 
 
distance
 
 
0.794
 
 
[0.763, 0.821]
 
 
Yes
 
 
Yes
 
 
0.791
 
 
[0.757, 0.820]
 
 
Yes
 
 
Yes
 
 
 
 
SBAS
 
 
skull
 
 
distance
 
 
0.793
 
 
[0.762, 0.820]
 
 
Yes
 
 
Yes
 
 
0.756
 
 
[0.717, 0.791]
 
 
Yes
 
 
No
 
 
 
 
ANSF
 
 
skull
 
 
angle
 
 
0.785
 
 
[0.754, 0.814]
 
 
Yes
 
 
Yes
 
 
0.771
 
 
[0.734, 0.803]
 
 
Yes
 
 
No
 
 
 
 
HBC2
 
 
hyoid
 
 
distance
 
 
0.776
 
 
[0.743, 0.806]
 
 
Yes
 
 
No
 
 
0.652
 
 
[0.602, 0.697]
 
 
No
 
 
No
 
 
 
 
HNSL
 
 
hard palate
 
 
distance
 
 
0.770
 
 
[0.735, 0.800]
 
 
Yes
 
 
No
 
 
0.731
 
 
[0.688, 0.769]
 
 
No
 
 
No
 
 
 
 
DIMD
 
 
dentition
 
 
distance
 
 
0.767
 
 
[0.733, 0.797]
 
 
Yes
 
 
No
 
 
0.730
 
 
[0.689, 0.767]
 
 
No
 
 
No
 
 
 
 
LAEA
 
 
larynx
 
 
distance
 
 
0.766
 
 
[0.731, 0.796]
 
 
Yes
 
 
No
 
 
0.715
 
 
[0.673, 0.753]
 
 
No
 
 
No
 
 
 
 
APNP
 
 
skull
 
 
angle
 
 
0.764
 
 
[0.729, 0.794]
 
 
Yes
 
 
No
 
 
0.764
 
 
[0.727, 0.797]
 
 
Yes
 
 
No
 
 
 
 
L4EA
 
 
larynx
 
 
distance
 
 
0.758
 
 
[0.723, 0.789]
 
 
Yes
 
 
No
 
 
0.750
 
 
[0.712, 0.784]
 
 
No
 
 
No
 
 
 
 
L3EA
 
 
larynx
 
 
distance
 
 
0.757
 
 
[0.722, 0.789]
 
 
Yes
 
 
No
 
 
0.754
 
 
[0.717, 0.787]
 
 
Yes
 
 
No
 
 
 
 
 In decreasing order of  ICC ( C ,1): MICD (0.98 [0.980,
0.985]), SHBN (0.96 [0.952, 0.965]), MIGD (0.95 [0.937, 0.954]), SHSW
(0.94 [0.931, 0.949]), SVTh* (0.93 [0.914, 0.936]), MCPD (0.92 [0.905,
0.930]), MCMD (0.92 [0.902, 0.927]), HBPL (0.91 [0.892, 0.920]), SSEN
(0.9 [0.889, 0.918]), SNAM (0.9 [0.886, 0.915]), SBNA (0.9 [0.881,
0.912]), SVTh (0.9 [0.881, 0.912]), SSEN* (0.9 [0.880, 0.911]), SSEG
(0.9 [0.879, 0.910]), SBAN (0.89 [0.877, 0.909]), MCGM (0.89 [0.867,
0.901]), SNAP (0.88 [0.861, 0.897]), ASCG* (0.87 [0.852, 0.890]), ACSN
(0.87 [0.847, 0.886]), MCGP (0.87 [0.844, 0.884]), MPGL (0.84 [0.819,
0.864]), MPGR (0.84 [0.815, 0.861]), HBNP (0.84 [0.810, 0.857]), MGPD
(0.83 [0.805, 0.853]), APNS (0.83 [0.800, 0.850]), SNNP (0.82 [0.797,
0.848]), ABSN (0.82 [0.792, 0.843]), LACT (0.81 [0.779, 0.833]), HBPG
(0.81 [0.778, 0.833]), SVTv* (0.8 [0.774, 0.830]), SBNP (0.8 [0.771,
0.827]), MGMD (0.8 [0.769, 0.826]), GAPD (0.8 [0.767, 0.825]), DIPD (0.8
[0.767, 0.824]), HBC4 (0.79 [0.763, 0.821]), SBAS (0.79 [0.762, 0.820]),
ANSF (0.79 [0.754, 0.814]), HBC2 (0.78 [0.743, 0.806]), HNSL (0.77
[0.735, 0.800]), DIMD (0.77 [0.733, 0.797]), LAEA (0.77 [0.731, 0.796]),
APNP (0.76 [0.729, 0.794]), L4EA (0.76 [0.723, 0.789]), L3EA (0.76
[0.722, 0.789]). 
 
 
 
 The twin correlations 
 
 “Raw” (i.e., uncorrected) correlations 
 
 By measure 
 
 rMZ 
 
 
 
  Figure 21.    Histogram of MZ
correlations. 
 
 
 
 
 
 
 
 
 
 
 
 
 
 Min. 
 1st Qu. 
 Median 
 Mean 
 3rd Qu. 
 Max. 
 
 
 
 
 0.0171 
 0.2793 
 0.4654 
 0.4636 
 0.6422 
 0.9225 
 
 
 
  SD  = 0.22,  IQR  = 0.36. 
 
 
 rDZ 
 
 
 
  Figure 22.    Histogram of DZ
correlations. 
 
 
 
 
 
 
 
 
 
 
 
 
 
 Min. 
 1st Qu. 
 Median 
 Mean 
 3rd Qu. 
 Max. 
 
 
 
 
 -0.1935 
 0.1051 
 0.2226 
 0.215 
 0.3114 
 0.5535 
 
 
 
  SD  = 0.14,  IQR  = 0.21. 
 
 
 rMZ vs rDZ 
 
 
 
  Figure 23.    Plots of MZ and DZ correlations
for each phenotype by measure (sorted in decreasing order of MZ
correlation). For each phenotype we show the MZ correlation (yellow
circle) and the DZ correlation (red arrow). The colored squared close to
the bottom of the plots show the type of the measures. 
 
 
 
 
  Table 17.    Measures with
 r MZ   > 0.90 ordered decreasingly by
 r MZ  . 
 
 
 
 
measure
 
 
domain
 
 
type
 
 
r MZ 
 
 
r DZ 
 
 
Krippendorff α
 
 
ICC(C,1)
 
 
95% CI
 
 
agr(GSEM)
 
 
95% CI
 
 
 
 
 
 
MICD
 
 
mandible
 
 
distance
 
 
0.9225
 
 
0.1909
 
 
0.98
 
 
0.98
 
 
[0.980, 0.985]
 
 
0.97
 
 
[0.963, 0.974]
 
 
 
 
MIGD
 
 
mandible
 
 
distance
 
 
0.9056
 
 
0.4124
 
 
0.94
 
 
0.95
 
 
[0.937, 0.954]
 
 
0.89
 
 
[0.875, 0.910]
 
 
 
 
 
 
 
 Comparing  r MZ   and  r DZ   
 Out of a total of 146 measures,  r MZ   >
 r DZ   for 138 measures (94.5%) and, of those,
 r MZ   > 2 r DZ   for 80 measures
(58%). 
 
 
 
 Corrected correlations 
 These are derived from the SEM phentypic model (see below for
details). 
 
 By measure 
 
 rMZ 
 
 
 
  Figure 24.    Histogram of MZ
correlations. 
 
 
 
 
 
 
 
 
 
 
 
 
 
 Min. 
 1st Qu. 
 Median 
 Mean 
 3rd Qu. 
 Max. 
 
 
 
 
 0.04099 
 0.5339 
 0.6522 
 0.6477 
 0.7983 
 1.025 
 
 
 
  SD  = 0.18,  IQR  = 0.26. There are 3 (2.1%) measures
with rMZ > 1.0: CS2A (1.025), SNOL (1.022), SNOR (1.011), but it
seems that for all them this could be due to numeric errors (as a
reminder, the latent twin covariance matrices are estimated freely,
without any constraints). 
 
 
 rDZ 
 
 
 
  Figure 25.    Histogram of DZ
correlations. 
 
 
 
 
 
 
 
 
 
 
 
 
 
 Min. 
 1st Qu. 
 Median 
 Mean 
 3rd Qu. 
 Max. 
 
 
 
 
 -0.7238 
 0.2462 
 0.3936 
 0.3762 
 0.5234 
 1.532 
 
 
 
  SD  = 0.28,  IQR  = 0.28. There are 3 (2.1%) measures
with rDZ > 1.0: HCCP (1.532), HMDC (1.158), HMSP (1.04). The first
two (HCCP and HMDC) are clearly too large to be just rounding error and
suggest that they violate various assumptions of the model and should be
flaged as problematic(see next section). 
 
 
 rMZ vs rDZ 
 
 
 
  Figure 26.    Plots of MZ and DZ correlations
for each phenotype by measure (sorted in decreasing order of MZ
correlation). For each phenotype we show the MZ correlation (yellow
circle) and the DZ correlation (red arrow). The colored squared close to
the bottom of the plots show the type of the measures. 
 
 
 
 
  Table 18.    Measures with
 r MZ   > 0.90 ordered decreasingly by
 r MZ  . 
 
 
 
 
measure
 
 
domain
 
 
type
 
 
r MZ 
 
 
r DZ 
 
 
Krippendorff α
 
 
ICC(C,1)
 
 
95% CI
 
 
agr(GSEM)
 
 
95% CI
 
 
 
 
 
 
CS2A
 
 
cervical
 
 
distance
 
 
1.0255
 
 
0.5612
 
 
0.53
 
 
0.60
 
 
[0.551, 0.651]
 
 
0.52
 
 
[0.461, 0.573]
 
 
 
 
SNOL
 
 
skull
 
 
distance
 
 
1.0218
 
 
0.6586
 
 
0.50
 
 
0.54
 
 
[0.480, 0.592]
 
 
0.45
 
 
[0.388, 0.508]
 
 
 
 
SNOR
 
 
skull
 
 
distance
 
 
1.0111
 
 
0.4789
 
 
0.46
 
 
0.53
 
 
[0.472, 0.585]
 
 
0.44
 
 
[0.378, 0.498]
 
 
 
 
SIOD
 
 
skull
 
 
distance
 
 
0.9636
 
 
0.3870
 
 
0.24
 
 
0.71
 
 
[0.666, 0.744]
 
 
0.61
 
 
[0.556, 0.663]
 
 
 
 
SNNP
 
 
skull
 
 
distance
 
 
0.9191
 
 
0.6066
 
 
0.77
 
 
0.82
 
 
[0.797, 0.848]
 
 
0.76
 
 
[0.721, 0.795]
 
 
 
 
 
 
 
 Comparing  r MZ   and  r DZ   
 Out of a total of 146 measures,  r MZ   >
 r DZ   for 130 measures (89%) and, of those,
 r MZ   > 2 r DZ   for 53 measures
(40.8%). 
 
 
 
 
 Measurements with warning flags 
 While we present below the estimates for all valid measurements
(except for the exact duplicates) in all participants with usable data,
there are several phenotypic measurements that should be treated with
care given that they violate in various ways and to various degrees the
assumptions of the model. More precisely, we computed a subjective
“warning score” based on: 
 
 the visual inspection of the histograms and QQ-plots, particularly
focusing on high skewness, kurtosis and signs of bi-modality, 
 weird latent twin correlations from the phenotypic model (see
previous section), 
 the comparison of the estimated skewness against -1 and 1 (high
skewness) and (-1,-0.5) and (0.5,1) (moderate skewness), 
 the comparison of kurtosis to (2,5), and 
 the formal Shapiro-Wilk normality test (please note that this is
highly sensitive, especially for large samples). 
 
 This composite subjective “warning score” should be used to weight
the interpretation of the results for each individual measure, with
values ≤ 3 probably posing no problems, and only scores ≥ 5 raising
potentially serious issues. 
 
 
  Table 19.    Measures with warnings that
shuld be interpretated with care. The  score  goes from 1 (very
weak reasons to worry) to 10 (very strong reasons to worry); probably
only scores > 3 might pose problems, and only ≥ 5 probably pose
serious problems… 
 
 
 
 
measure
 
 
warning score
 
 
reasons
 
 
 
 
 
 
 GAPD 
 
 
8
 
 
Seems skewed, highly skewed, leptokurtic, Shapiro-Wilk significant
 
 
 
 
 GRPD 
 
 
8
 
 
Seems skewed, highly skewed, leptokurtic, Shapiro-Wilk significant
 
 
 
 
 HARF 
 
 
8
 
 
Seems skewed, highly skewed, Shapiro-Wilk significant
 
 
 
 
 HCCP 
 
 
8
 
 
Latent rDZ correlation much larger than 1.0, moderately skewed,
Shapiro-Wilk significant
 
 
 
 
 HCMP 
 
 
8
 
 
Seems skewed, highly skewed, leptokurtic, Shapiro-Wilk significant
 
 
 
 
 HDMP 
 
 
8
 
 
Sugests high kurtosis, highly skewed, leptokurtic, Shapiro-Wilk
significant
 
 
 
 
 HMDC 
 
 
8
 
 
Latent rDZ correlation much larger than 1.0, Shapiro-Wilk significant
 
 
 
 
 HMSP 
 
 
8
 
 
Seems skewed, highly skewed, leptokurtic, Shapiro-Wilk significant
 
 
 
 
 PPW2 
 
 
8
 
 
Sugests high kurtosis, leptokurtic, Shapiro-Wilk significant
 
 
 
 
 PPW3 
 
 
8
 
 
Sugests high kurtosis, moderately skewed, leptokurtic, Shapiro-Wilk
significant
 
 
 
 
 PPWC 
 
 
8
 
 
Sugests high kurtosis, moderately skewed, leptokurtic, Shapiro-Wilk
significant
 
 
 
 
 PPW4 
 
 
6
 
 
Sugests high kurtosis, leptokurtic, Shapiro-Wilk significant
 
 
 
 
 DDAP 
 
 
5
 
 
Seems skewed, moderately skewed, leptokurtic, Shapiro-Wilk significant
 
 
 
 
 HCPP 
 
 
5
 
 
Seems skewed, leptokurtic, Shapiro-Wilk significant
 
 
 
 
 HMTC 
 
 
5
 
 
Seems bimodal, moderately skewed, leptokurtic, Shapiro-Wilk significant
 
 
 
 
 AANP 
 
 
3
 
 
Seems skewed, moderately skewed, Shapiro-Wilk significant
 
 
 
 
 HBC2 
 
 
3
 
 
moderately skewed, Shapiro-Wilk significant
 
 
 
 
 HMAC 
 
 
3
 
 
Seems skewed, moderately skewed, Shapiro-Wilk significant
 
 
 
 
 L2EA 
 
 
3
 
 
Seems skewed, moderately skewed, Shapiro-Wilk significant
 
 
 
 
 L3EA 
 
 
3
 
 
moderately skewed, Shapiro-Wilk significant
 
 
 
 
 OCLW 
 
 
3
 
 
Seems skewed, moderately skewed, Shapiro-Wilk significant
 
 
 
 
 PPW5 
 
 
3
 
 
Sugests high kurtosis, Shapiro-Wilk significant
 
 
 
 
 HACL 
 
 
2
 
 
Seems bimodal, Shapiro-Wilk significant
 
 
 
 
 PPW6 
 
 
2
 
 
Sugests high kurtosis, Shapiro-Wilk significant
 
 
 
 
 PWTP 
 
 
2
 
 
Seems skewed, Shapiro-Wilk significant
 
 
 
 
 ALNS 
 
 
1
 
 
Seems skewed, Shapiro-Wilk significant
 
 
 
 
 APNP 
 
 
1
 
 
Shapiro-Wilk significant
 
 
 
 
 APSN 
 
 
1
 
 
Shapiro-Wilk significant
 
 
 
 
 ASCG* 
 
 
1
 
 
Shapiro-Wilk significant
 
 
 
 
 CS2A 
 
 
1
 
 
Shapiro-Wilk significant
 
 
 
 
 CS2H 
 
 
1
 
 
Shapiro-Wilk significant
 
 
 
 
 CS3H 
 
 
1
 
 
Shapiro-Wilk significant
 
 
 
 
 CS4H 
 
 
1
 
 
Shapiro-Wilk significant
 
 
 
 
 DAIS 
 
 
1
 
 
Shapiro-Wilk significant
 
 
 
 
 DICD 
 
 
1
 
 
Shapiro-Wilk significant
 
 
 
 
 DIIL 
 
 
1
 
 
Shapiro-Wilk significant
 
 
 
 
 DIPD 
 
 
1
 
 
Shapiro-Wilk significant
 
 
 
 
 DMAC 
 
 
1
 
 
Shapiro-Wilk significant
 
 
 
 
 HACP 
 
 
1
 
 
Shapiro-Wilk significant
 
 
 
 
 HAPL 
 
 
1
 
 
Shapiro-Wilk significant
 
 
 
 
 HARC 
 
 
1
 
 
Shapiro-Wilk significant
 
 
 
 
 HBC3 
 
 
1
 
 
Shapiro-Wilk significant
 
 
 
 
 HBC4 
 
 
1
 
 
Shapiro-Wilk significant
 
 
 
 
 HBNP 
 
 
1
 
 
Shapiro-Wilk significant
 
 
 
 
 HBPL 
 
 
1
 
 
Shapiro-Wilk significant
 
 
 
 
 HCCC 
 
 
1
 
 
Shapiro-Wilk significant
 
 
 
 
 HCTH 
 
 
1
 
 
Shapiro-Wilk significant
 
 
 
 
 HHPL 
 
 
1
 
 
Shapiro-Wilk significant
 
 
 
 
 HICH 
 
 
1
 
 
Shapiro-Wilk significant
 
 
 
 
 HICL* 
 
 
1
 
 
Shapiro-Wilk significant
 
 
 
 
 HIMH 
 
 
1
 
 
Shapiro-Wilk significant
 
 
 
 
 HMTH 
 
 
1
 
 
Shapiro-Wilk significant
 
 
 
 
 HNAP 
 
 
1
 
 
Shapiro-Wilk significant
 
 
 
 
 HPTH 
 
 
1
 
 
Shapiro-Wilk significant
 
 
 
 
 L2EP 
 
 
1
 
 
Shapiro-Wilk significant
 
 
 
 
 L3CT 
 
 
1
 
 
Shapiro-Wilk significant
 
 
 
 
 L3EP 
 
 
1
 
 
Shapiro-Wilk significant
 
 
 
 
 L4CT 
 
 
1
 
 
Shapiro-Wilk significant
 
 
 
 
 L4EP 
 
 
1
 
 
Shapiro-Wilk significant
 
 
 
 
 L5CT 
 
 
1
 
 
Shapiro-Wilk significant
 
 
 
 
 LACT 
 
 
1
 
 
Seems skewed
 
 
 
 
 LAEA 
 
 
1
 
 
Shapiro-Wilk significant
 
 
 
 
 LAEP 
 
 
1
 
 
Shapiro-Wilk significant
 
 
 
 
 LHCT 
 
 
1
 
 
Shapiro-Wilk significant
 
 
 
 
 LHEA 
 
 
1
 
 
Shapiro-Wilk significant
 
 
 
 
 LNEA 
 
 
1
 
 
Shapiro-Wilk significant
 
 
 
 
 MCGD 
 
 
1
 
 
Shapiro-Wilk significant
 
 
 
 
 MCGL 
 
 
1
 
 
Shapiro-Wilk significant
 
 
 
 
 MCGR 
 
 
1
 
 
Shapiro-Wilk significant
 
 
 
 
 MCPD 
 
 
1
 
 
Shapiro-Wilk significant
 
 
 
 
 MIGD 
 
 
1
 
 
Shapiro-Wilk significant
 
 
 
 
 MMPG 
 
 
1
 
 
Shapiro-Wilk significant
 
 
 
 
 MPGL 
 
 
1
 
 
Shapiro-Wilk significant
 
 
 
 
 MPGR 
 
 
1
 
 
Shapiro-Wilk significant
 
 
 
 
 OCHL 
 
 
1
 
 
Shapiro-Wilk significant
 
 
 
 
 OCHW 
 
 
1
 
 
Shapiro-Wilk significant
 
 
 
 
 OCPR 
 
 
1
 
 
Shapiro-Wilk significant
 
 
 
 
 OMHL 
 
 
1
 
 
Shapiro-Wilk significant
 
 
 
 
 OMHW 
 
 
1
 
 
Shapiro-Wilk significant
 
 
 
 
 OMLW 
 
 
1
 
 
Shapiro-Wilk significant
 
 
 
 
 OPHL 
 
 
1
 
 
Shapiro-Wilk significant
 
 
 
 
 OPHW 
 
 
1
 
 
Shapiro-Wilk significant
 
 
 
 
 OPLW 
 
 
1
 
 
Shapiro-Wilk significant
 
 
 
 
 SBAN 
 
 
1
 
 
Shapiro-Wilk significant
 
 
 
 
 SBAS 
 
 
1
 
 
Shapiro-Wilk significant
 
 
 
 
 SBNA 
 
 
1
 
 
Shapiro-Wilk significant
 
 
 
 
 SBNP 
 
 
1
 
 
Shapiro-Wilk significant
 
 
 
 
 SHBN 
 
 
1
 
 
Shapiro-Wilk significant
 
 
 
 
 SIOD 
 
 
1
 
 
Shapiro-Wilk significant
 
 
 
 
 SNAP 
 
 
1
 
 
Shapiro-Wilk significant
 
 
 
 
 SNNA 
 
 
1
 
 
Shapiro-Wilk significant
 
 
 
 
 SNNP 
 
 
1
 
 
Shapiro-Wilk significant
 
 
 
 
 SSEG 
 
 
1
 
 
Shapiro-Wilk significant
 
 
 
 
 SSEN 
 
 
1
 
 
Shapiro-Wilk significant
 
 
 
 
 SVTh* 
 
 
1
 
 
Shapiro-Wilk significant
 
 
 
 
 
 
 
 The SEM genetic model 
 
 Description of the SEM model 
 
 Introduction and notations 
 The SEM model is fitted using   OpenMX   (called from
 R  using the  OpenMx  library). 
 As a reminder, for each individual measure,  m , we have the
values observed for the two twins of each twin pair (denoted as  twin
1  and  twin 2 , respectively), by each of the two raters
(denoted as  rater 1  and  rater 2 , respectively), such
that we have, in fact, four types of values: those measured by rater 1
for twin 1 denoted as  m  11 , those measured by rater 1
for twin 2 denoted as  m  12 , those measured by rater 2
for twin 1 denoted as  m  21 , and those measured by
rater 2 for twin 2 denoted as  m  22  (thus, the general
convention is  m   rt  , where  r  is the
rater and  t  the twin). We  normalize  (i.e.,
 z -score) the measure  m  separately by rater, but for a
given rater, across both twins in all twin pairs and across MZ and DZ
twins; that is, we compute  m ’  r.   =
( m   r.   -
 mean ( m   r.  )) /
 sd ( m   r.  ), where  r  ∈ {1,2}
is the rater, and the notation   r.   means the values
for both twins. 
 
 
 Modelling the two raters 
  A priori  we can image at least four ways to model our two
raters,  rater 1  and  rater 2 , depending on how we
consider their (latent) means and error variances: 
 
  IMIV : the two raters have identical means and error
variances: this model assumes that the two raters are essentially
identical (i.e., they make essentially the same judgments barring some
random noise), and has the smallest number of free parameters; 
  DMIV : the two raters have different means but
identical error variances: this model assumes that the raters have
different latent “true scores”, but identical error rates; 
  IMDV : the two raters have identical means but
different error variances: this model assumes that the raters have the
same “true scores”, but different error rates; 
  DMDV : the two raters have different means and error
variances: this model assumes that the raters may be different in both
respects, and has the largest number of free parameters. 
 
 Before seeing the data, in an ideal world we might want to fit the
least constrained model  DMDV  (i.e., metaphotically, let
the data decide), but this comes with too many free parameters for our
sample. At the other extreme, while the two raters were trained in the
same way and did discuss edge cases between them, making sure their
criteria stay in sync, they were emphatically not clones of each others
and debriefing during and after the landmarking revealed subtle
differences and perceptual biases, potentially suggesting that the
 IMIV  might be too simplistic. 
 However, it is difficult to decide between these four possibilities
based only on  a priori  considerations, so we first examined the
correlations (Pearson’s and Spearman) and the differences between the
values obtained by the first and second rater for the same individual
and measure. As can be seen in the figure below, the two raters tend to
agree rather well on the  mean  of the “raw” measurements (3rd
histogram, narrowly centered on 0.0, and 2nd scatterplot), while their
correlations are medium to high. This suggests that the two models that
assume identical means might be retained, but it is still hard to decide
if the rater error variances should be considered equal or
different. 
 
 
   Figure 2.    Deciding
between alernative ways to model the two raters. From top-left to
bottom-right: first row: histograms of the correlations between the two
raters (Pearson’s and Spearman’s), the histogram of the differences in
mean estimates between the raters, scatterplot between the mean
differences and inter-rater Pearson’s correlations; second row:
scatterplots between mean rater differences. Please note that these were
computed on the “raw” (i.e., not  z -scored) measurements across
twin pairs, zygosity types and measures.  
 
 To help settle this, for each measure  m , we fitted two SEM
models (for details, please see next section), one with one rater error
variance (denoted as  M   m ,1 r  ) and
the other with two rater error variances (denoted
 M   m ,2 r  ). Please note that we are
not interested here in deciding for each individual measure  m 
what rater model fits best, but rather in obtaining the overall
distribution of the the best fitting model across all the measures, and
to combine this with the  a priori  reasoning and the exploratory
plots discussed above to decide on a single rater model to apply to all
measures. Please note that while having a different rater model for each
measure might seem the best approach, this inflates the number of tests
performed, requiring a stricter multiple testing correction which, on
our database, would drastically reduce our power to detect significant
effects. Moreover, arguably, given that the measures are derived, using
more or less complex procedures, from the actual landmarks and
semilandmarks that were placed by each rater in “one go”, it seems
reasonable to use the same rater model across measures. In particular,
we used both  Akaike’s
Information Criterion  or  AIC  (in fact, the AIC is corrected
using the “Parameters Penalty” as implemented by  OpenMx ,
which uses the number of free parameters in the model rather than the
degrees of freedom; please see the help pages for  summary 
and  mxCompare  for details) and the likelihood ratio test
(again, please see  mxCompare  in  OpenMx ), and
the summaries over all measures are as follows (please note that the
comparison is performed between the 2-raters and 1-rater model, which
means that the difference in AIC scores, ΔAIC, is negative if an only if
the 2-rater model has a lower AIC score than the 1-rater model – i.e.,
it is the better model – and it is positive otherwise): 
 
 
 
  Figure 28.    Histogram of the ΔAIC
differences in AIC scores between the 2-raters and 1-rate models (i.e.,
negative ΔAIC means that the 2-rater model fits the data better) across
all measures. Please note that the dashed vertical lines marking the 2
(red) and 10 (blue) AIC points thresholds are barely visible. 
 
 
 
 
 
 
 
 
 
 
 
 
 
 Min. 
 1st Qu. 
 Median 
 Mean 
 3rd Qu. 
 Max. 
 
 
 
 
 -1.999 
 -1.895 
 -1.583 
 -0.6897 
 -0.6001 
 28.06 
 
 
 
 
  using a cutoff of 2 AIC points for “different enough”, we
have: 
 
 0 (0%) measures supporting the 2-raters model (i.e., with ΔAIC <
-2), 
 136 (93.2%) measures for which the two models are equivalent (i.e.,
-2 <= ΔAIC <= 2), and 
 10 (6.8%) measures supporting the 1-rater model (i.e., with ΔAIC
> 2). 
  
  adding a more stringent cutoff of 10 AIC points for
“overwhelmingly different”, we have: 
 
 0 (0%) measures overwhelmingly supporting the 2-raters model (i.e.,
with ΔAIC < -), 
 144 (98.6%) measures for which the two models are equivalent (i.e.,
-10 <= ΔAIC <= 10), and 
 2 (1.4%) measures overwhelmingly supporting the 1-rater model (i.e.,
with ΔAIC > 10). 
  
  finally, using the likelihood test and an  α -level of
0.05 for judging significance: 
 
 10 (6.8%) measures show a significant difference between the two
models (i.e.,  p  < 0.05), of which:
 
 0 (0%) favour the 2-raters model (i.e., ΔAIC < 0) and 
 10 (100%) favour the 1-rater model (i.e., ΔAIC > 0). 
  
  
 
 Taking everything together, it is clear that  modelling the two
raters as being essentially identical  (i.e., with the same latent
mean and error variances),  IMIV , is the best choice we
can make. 
 
 
 The phenotypic model 
 The first SEM model that we define is at the  phenotypic 
level, where we model the two raters and the effects of the various
covariates on the measurements. 
 For a given measure  m  and twin pair (MZ or DZ) with twins 1
and 2, for a particular member of this pair,  i , we model these
covariates as: 
 
  m   i   ~  μ  +
 β   s   sex   i   +
 β   a   age   i   +
 β   a 2  age   i   2 
+  β   b   ICV   i   +
 β   as   age   i   sex   i  
+
 β   bs   ICV   i   sex   i   
 where: 
 
 the symbol ~ stands for linear regression, 
  μ  is the intercept, modeled as the same for twin type and
twin pair member, 
 the covariates for the twin member considered  i  are
 sex   i  ,  age   i   and
 age   i   2  at scan (the squared age
models non-linear developmental and aging effects), and intracranial
brain volume  ICV   i  , each with its own
regression slope  β   s  ,
 β   a  ,  β   a2   and
 β   b  , 
 in principle, we can also model some potentially relevant
interactions such between age and sex, and ICV and sex, but given our
small sample size we decided to not do that here (i.e., we forced their
slopes,  β   as   and
 β   bs  , to be 0.0). 
 
 Please note that: 
 
 there is a single intercept in the model, reflecting the fact that
we have no  a priori  reasons to believe that there should be
differences in intercept between twin pair members and zygosity types
(i.e., between MZ and DZ twins), 
 likewise, we have no reason to assume that the slopes differ between
twin types and twin pair members as well, 
 we do not model here the interactions between covariates do to the
size of our sample. 
 
 With these, the SEM model is: 
 
 
   Figure 3.    The
phenotypic SEM model. Given a measure  m  (not explicitely shwon
here),  f1  and  f2  are the latent values of this measure
for the two members of a twin pair,  1  and  2 , as
measured each by the two raters. This produces 4 observed values, 2 per
twin pair member, denoted as  Ph11  for the first rater’s value
for the first twin and as  Ph21  for the second rater’s value for
the first twin, and as  Ph12  for the first rater’s value for the
second twin and as  Ph22  for the second rater’s value for the
second twin, respectively.  s f  2   is the
rater’s unique error variance,  s e  2   is the
mesurement error, and  s 12mz   and
 s 12dz   represent the correlations between the two
twins wen they are MZ and DZ twins, respectively.   
 
 We derive from this model the latent phenotype correlations for MZ
and DZ twins respectively,  r   MZ   and
 r   DZ  , which we use to decide if we should
further fit an ADE model (if and only if
 r   MZ   > 2 r   DZ  )
or and ACE model (otherwise). 
 ( N.B.  Please note that for 0 phenotypes there were some
issues obtaining a estimate of these latent correlations, and we had to
“fall back” on using the “raw” MZ and DZ correlations when making this
heuristic decision; nevertheless, as our main interest resides with the
additive component  A , this should not affect our results too
much, a point supported by checks using different phenotypic
models.) 
 
 
 Fitting the ADE or ACE models 
 With these, we further fitted the suggested model (ACE or ADE): 
 
 
   Figure 4.    The genetic
SEM model. Most conventions are as in the phenotypic SEM model above,
except that now the latent measurements  f1  and  f2  is
each decomposed into the effects of the additive genetic variance
 A , of the non-shared environment  E , and of the
dominance genetic variance  D  or the shared environment
 C , as appropriate. The correlation between the additive
components  A  of the two twins differs between MZ (1.0) and DZ
(0.5) twins, as does the correlations between dominance effects
 D  (1.0 for MZ and 0.25 for DZ), but that for the shared
environment is the same (1.0).   
 
 Besides fitting this model, we also fit models where we fix each of
the components  A ,  E  and  C  or  D  to 0,
in order to statistically test their contributions. 
 
 
 
 Results for the SEM models 
 With these, we focus now on the results of fitting these models for
each measure independently. We report both the  unstandardized 
components  A ,  C  or  D , and  E , as well
as the  standardized  measures of interest
 A  std  (or narrow-sense heritability,
 h  2 ),  C  std  (and associated
familiality,  F  2 ) or  D  std  (and
associated broad-sense heritability,  H  2 ), and
 A  std  (or non-shared environmental variance,
 e  2 ). The results of main interest are the
latter. 
 Please note that we show here all the measures, irrespective of their
warning flag values, but that we will remove those with warnings ≥ 5
later on… 
 
 By measure 
 There are 93 measures where  ACE  seems the appropriate model
(of which 29 have  ICC(C,1)  >= 0.75 and 24 have 95% CI
 ICC(C,1)  low >= 0.75), and 53 where  ADE  seems the
appropriate model (of which 15 have  ICC(C,1)  >= 0.75 and 13
have 95% CI  ICC(C,1)  low >= 0.75): 
 
 
  Table 20.    The genetic models for all
measures, showing first those with  ACE , ordered by
agreement. 
 
 
 
 
measure
 
 
domain
 
 
type
 
 
genetic model
 
 
ICC(C,1)
 
 
ICC(C,1) >= 0.75
 
 
95% ICC(C,1) low >= 0.75
 
 
 
 
 
 
MICD
 
 
mandible
 
 
distance
 
 
ACE
 
 
0.98
 
 
Yes
 
 
Yes
 
 
 
 
SHBN
 
 
skull
 
 
distance
 
 
ACE
 
 
0.96
 
 
Yes
 
 
Yes
 
 
 
 
MIGD
 
 
mandible
 
 
distance
 
 
ACE
 
 
0.95
 
 
Yes
 
 
Yes
 
 
 
 
SHSW
 
 
skull
 
 
distance
 
 
ACE
 
 
0.94
 
 
Yes
 
 
Yes
 
 
 
 
SVTh*
 
 
general
 
 
distance
 
 
ACE
 
 
0.93
 
 
Yes
 
 
Yes
 
 
 
 
HBPL
 
 
hard palate
 
 
distance
 
 
ACE
 
 
0.91
 
 
Yes
 
 
Yes
 
 
 
 
SSEN
 
 
skull
 
 
distance
 
 
ACE
 
 
0.90
 
 
Yes
 
 
Yes
 
 
 
 
SNAM
 
 
skull
 
 
distance
 
 
ACE
 
 
0.90
 
 
Yes
 
 
Yes
 
 
 
 
SBNA
 
 
skull
 
 
distance
 
 
ACE
 
 
0.90
 
 
Yes
 
 
Yes
 
 
 
 
SVTh
 
 
general
 
 
distance
 
 
ACE
 
 
0.90
 
 
Yes
 
 
Yes
 
 
 
 
SSEG
 
 
skull
 
 
distance
 
 
ACE
 
 
0.90
 
 
Yes
 
 
Yes
 
 
 
 
SBAN
 
 
skull
 
 
distance
 
 
ACE
 
 
0.89
 
 
Yes
 
 
Yes
 
 
 
 
MCGM
 
 
mandible
 
 
angle
 
 
ACE
 
 
0.89
 
 
Yes
 
 
Yes
 
 
 
 
SNAP
 
 
skull
 
 
distance
 
 
ACE
 
 
0.88
 
 
Yes
 
 
Yes
 
 
 
 
ASCG*
 
 
skull
 
 
angle
 
 
ACE
 
 
0.87
 
 
Yes
 
 
Yes
 
 
 
 
MCGP
 
 
mandible
 
 
angle
 
 
ACE
 
 
0.87
 
 
Yes
 
 
Yes
 
 
 
 
HBNP
 
 
hyoid
 
 
distance
 
 
ACE
 
 
0.84
 
 
Yes
 
 
Yes
 
 
 
 
APNS
 
 
skull
 
 
angle
 
 
ACE
 
 
0.83
 
 
Yes
 
 
Yes
 
 
 
 
SNNP
 
 
skull
 
 
distance
 
 
ACE
 
 
0.82
 
 
Yes
 
 
Yes
 
 
 
 
LACT
 
 
larynx
 
 
distance
 
 
ACE
 
 
0.81
 
 
Yes
 
 
Yes
 
 
 
 
HBPG
 
 
hyoid
 
 
distance
 
 
ACE
 
 
0.81
 
 
Yes
 
 
Yes
 
 
 
 
SVTv*
 
 
general
 
 
distance
 
 
ACE
 
 
0.80
 
 
Yes
 
 
Yes
 
 
 
 
GAPD
 
 
general
 
 
Procrustes dist.
 
 
ACE
 
 
0.80
 
 
Yes
 
 
Yes
 
 
 
 
HBC4
 
 
hyoid
 
 
distance
 
 
ACE
 
 
0.79
 
 
Yes
 
 
Yes
 
 
 
 
MCPD
 
 
mandible
 
 
distance
 
 
ADE
 
 
0.92
 
 
Yes
 
 
Yes
 
 
 
 
MCMD
 
 
mandible
 
 
distance
 
 
ADE
 
 
0.92
 
 
Yes
 
 
Yes
 
 
 
 
SSEN*
 
 
skull
 
 
distance
 
 
ADE
 
 
0.90
 
 
Yes
 
 
Yes
 
 
 
 
ACSN
 
 
skull
 
 
angle
 
 
ADE
 
 
0.87
 
 
Yes
 
 
Yes
 
 
 
 
MPGL
 
 
mandible
 
 
distance
 
 
ADE
 
 
0.84
 
 
Yes
 
 
Yes
 
 
 
 
MPGR
 
 
mandible
 
 
distance
 
 
ADE
 
 
0.84
 
 
Yes
 
 
Yes
 
 
 
 
MGPD
 
 
mandible
 
 
distance
 
 
ADE
 
 
0.83
 
 
Yes
 
 
Yes
 
 
 
 
ABSN
 
 
skull
 
 
angle
 
 
ADE
 
 
0.82
 
 
Yes
 
 
Yes
 
 
 
 
SBNP
 
 
skull
 
 
distance
 
 
ADE
 
 
0.80
 
 
Yes
 
 
Yes
 
 
 
 
MGMD
 
 
mandible
 
 
distance
 
 
ADE
 
 
0.80
 
 
Yes
 
 
Yes
 
 
 
 
DIPD
 
 
dentition
 
 
distance
 
 
ADE
 
 
0.80
 
 
Yes
 
 
Yes
 
 
 
 
SBAS
 
 
skull
 
 
distance
 
 
ADE
 
 
0.79
 
 
Yes
 
 
Yes
 
 
 
 
ANSF
 
 
skull
 
 
angle
 
 
ADE
 
 
0.79
 
 
Yes
 
 
Yes
 
 
 
 
HBC2
 
 
hyoid
 
 
distance
 
 
ACE
 
 
0.78
 
 
Yes
 
 
No
 
 
 
 
HNSL
 
 
hard palate
 
 
distance
 
 
ACE
 
 
0.77
 
 
Yes
 
 
No
 
 
 
 
DIMD
 
 
dentition
 
 
distance
 
 
ACE
 
 
0.77
 
 
Yes
 
 
No
 
 
 
 
LAEA
 
 
larynx
 
 
distance
 
 
ACE
 
 
0.77
 
 
Yes
 
 
No
 
 
 
 
L4EA
 
 
larynx
 
 
distance
 
 
ACE
 
 
0.76
 
 
Yes
 
 
No
 
 
 
 
APNP
 
 
skull
 
 
angle
 
 
ADE
 
 
0.76
 
 
Yes
 
 
No
 
 
 
 
L3EA
 
 
larynx
 
 
distance
 
 
ADE
 
 
0.76
 
 
Yes
 
 
No
 
 
 
 
LAEP
 
 
larynx
 
 
distance
 
 
ACE
 
 
0.74
 
 
No
 
 
No
 
 
 
 
L5EA
 
 
larynx
 
 
distance
 
 
ACE
 
 
0.74
 
 
No
 
 
No
 
 
 
 
SVTv
 
 
general
 
 
distance
 
 
ACE
 
 
0.73
 
 
No
 
 
No
 
 
 
 
L2CT
 
 
larynx
 
 
distance
 
 
ACE
 
 
0.73
 
 
No
 
 
No
 
 
 
 
ASNP
 
 
skull
 
 
angle
 
 
ACE
 
 
0.72
 
 
No
 
 
No
 
 
 
 
L5CT
 
 
larynx
 
 
distance
 
 
ACE
 
 
0.71
 
 
No
 
 
No
 
 
 
 
MCGD
 
 
mandible
 
 
distance
 
 
ACE
 
 
0.71
 
 
No
 
 
No
 
 
 
 
HBC3
 
 
hyoid
 
 
distance
 
 
ACE
 
 
0.71
 
 
No
 
 
No
 
 
 
 
PPW7
 
 
pharynx
 
 
curvature
 
 
ACE
 
 
0.71
 
 
No
 
 
No
 
 
 
 
OMLW
 
 
oral
 
 
ratio
 
 
ACE
 
 
0.70
 
 
No
 
 
No
 
 
 
 
L3CT
 
 
larynx
 
 
distance
 
 
ACE
 
 
0.69
 
 
No
 
 
No
 
 
 
 
CS7H
 
 
cervical
 
 
distance
 
 
ACE
 
 
0.69
 
 
No
 
 
No
 
 
 
 
MCGR
 
 
mandible
 
 
distance
 
 
ACE
 
 
0.68
 
 
No
 
 
No
 
 
 
 
MCGL
 
 
mandible
 
 
distance
 
 
ACE
 
 
0.68
 
 
No
 
 
No
 
 
 
 
L2EP
 
 
larynx
 
 
distance
 
 
ACE
 
 
0.67
 
 
No
 
 
No
 
 
 
 
LHCT
 
 
larynx
 
 
distance
 
 
ACE
 
 
0.66
 
 
No
 
 
No
 
 
 
 
SNNA
 
 
skull
 
 
distance
 
 
ACE
 
 
0.66
 
 
No
 
 
No
 
 
 
 
HIML*
 
 
hard palate
 
 
distance
 
 
ACE
 
 
0.65
 
 
No
 
 
No
 
 
 
 
DAIS
 
 
dentition
 
 
angle
 
 
ACE
 
 
0.65
 
 
No
 
 
No
 
 
 
 
HIML
 
 
hard palate
 
 
distance
 
 
ACE
 
 
0.63
 
 
No
 
 
No
 
 
 
 
SPUN
 
 
soft palate
 
 
distance
 
 
ACE
 
 
0.63
 
 
No
 
 
No
 
 
 
 
AASN
 
 
skull
 
 
angle
 
 
ACE
 
 
0.63
 
 
No
 
 
No
 
 
 
 
HIPL*
 
 
hard palate
 
 
distance
 
 
ACE
 
 
0.63
 
 
No
 
 
No
 
 
 
 
LHEA
 
 
larynx
 
 
distance
 
 
ACE
 
 
0.61
 
 
No
 
 
No
 
 
 
 
ALNS
 
 
skull
 
 
angle
 
 
ACE
 
 
0.60
 
 
No
 
 
No
 
 
 
 
CS2A
 
 
cervical
 
 
distance
 
 
ACE
 
 
0.60
 
 
No
 
 
No
 
 
 
 
CS3H
 
 
cervical
 
 
distance
 
 
ACE
 
 
0.60
 
 
No
 
 
No
 
 
 
 
CS4H
 
 
cervical
 
 
distance
 
 
ACE
 
 
0.59
 
 
No
 
 
No
 
 
 
 
L3EP
 
 
larynx
 
 
distance
 
 
ACE
 
 
0.57
 
 
No
 
 
No
 
 
 
 
HAML
 
 
hard palate
 
 
distance
 
 
ACE
 
 
0.56
 
 
No
 
 
No
 
 
 
 
SNOL
 
 
skull
 
 
distance
 
 
ACE
 
 
0.54
 
 
No
 
 
No
 
 
 
 
HAPL
 
 
hard palate
 
 
distance
 
 
ACE
 
 
0.52
 
 
No
 
 
No
 
 
 
 
HHPL
 
 
hard palate
 
 
distance
 
 
ACE
 
 
0.50
 
 
No
 
 
No
 
 
 
 
OPLW
 
 
oral
 
 
ratio
 
 
ACE
 
 
0.50
 
 
No
 
 
No
 
 
 
 
OPHW
 
 
oral
 
 
ratio
 
 
ACE
 
 
0.50
 
 
No
 
 
No
 
 
 
 
OMHW
 
 
oral
 
 
ratio
 
 
ACE
 
 
0.48
 
 
No
 
 
No
 
 
 
 
PWTP
 
 
pharynx
 
 
Procrustes dist.
 
 
ACE
 
 
0.48
 
 
No
 
 
No
 
 
 
 
HACP
 
 
hard palate
 
 
angle
 
 
ACE
 
 
0.47
 
 
No
 
 
No
 
 
 
 
OCMR
 
 
oral
 
 
ratio
 
 
ACE
 
 
0.47
 
 
No
 
 
No
 
 
 
 
DSIL
 
 
dentition
 
 
distance
 
 
ACE
 
 
0.45
 
 
No
 
 
No
 
 
 
 
OMHL
 
 
oral
 
 
ratio
 
 
ACE
 
 
0.44
 
 
No
 
 
No
 
 
 
 
HCTH
 
 
hard palate
 
 
distance
 
 
ACE
 
 
0.44
 
 
No
 
 
No
 
 
 
 
CS5H
 
 
cervical
 
 
distance
 
 
ACE
 
 
0.43
 
 
No
 
 
No
 
 
 
 
PPW6
 
 
pharynx
 
 
curvature
 
 
ACE
 
 
0.43
 
 
No
 
 
No
 
 
 
 
HIPH
 
 
hard palate
 
 
distance
 
 
ACE
 
 
0.43
 
 
No
 
 
No
 
 
 
 
PPW5
 
 
pharynx
 
 
curvature
 
 
ACE
 
 
0.41
 
 
No
 
 
No
 
 
 
 
OCHW
 
 
oral
 
 
ratio
 
 
ACE
 
 
0.41
 
 
No
 
 
No
 
 
 
 
HICH
 
 
hard palate
 
 
distance
 
 
ACE
 
 
0.39
 
 
No
 
 
No
 
 
 
 
HICL*
 
 
hard palate
 
 
distance
 
 
ACE
 
 
0.39
 
 
No
 
 
No
 
 
 
 
OPHL
 
 
oral
 
 
ratio
 
 
ACE
 
 
0.38
 
 
No
 
 
No
 
 
 
 
HNAP
 
 
hard palate
 
 
distance
 
 
ACE
 
 
0.37
 
 
No
 
 
No
 
 
 
 
HARC
 
 
hard palate
 
 
angle
 
 
ACE
 
 
0.36
 
 
No
 
 
No
 
 
 
 
OCHL
 
 
oral
 
 
ratio
 
 
ACE
 
 
0.35
 
 
No
 
 
No
 
 
 
 
PPW4
 
 
pharynx
 
 
curvature
 
 
ACE
 
 
0.31
 
 
No
 
 
No
 
 
 
 
HCCC
 
 
hard palate
 
 
curvature
 
 
ACE
 
 
0.31
 
 
No
 
 
No
 
 
 
 
PPWC
 
 
pharynx
 
 
curvature
 
 
ACE
 
 
0.27
 
 
No
 
 
No
 
 
 
 
HACL
 
 
hard palate
 
 
distance
 
 
ACE
 
 
0.26
 
 
No
 
 
No
 
 
 
 
OCLW
 
 
oral
 
 
ratio
 
 
ACE
 
 
0.25
 
 
No
 
 
No
 
 
 
 
DMAC
 
 
dentition
 
 
curvature
 
 
ACE
 
 
0.21
 
 
No
 
 
No
 
 
 
 
HMDC
 
 
hard palate
 
 
curvature
 
 
ACE
 
 
0.21
 
 
No
 
 
No
 
 
 
 
HARF
 
 
hard palate
 
 
angle
 
 
ACE
 
 
0.16
 
 
No
 
 
No
 
 
 
 
HCCP
 
 
hard palate
 
 
Procrustes dist.
 
 
ACE
 
 
0.11
 
 
No
 
 
No
 
 
 
 
HMSP
 
 
hard palate
 
 
Procrustes dist.
 
 
ACE
 
 
0.11
 
 
No
 
 
No
 
 
 
 
HMTC
 
 
hard palate
 
 
distance
 
 
ACE
 
 
0.07
 
 
No
 
 
No
 
 
 
 
LNEA
 
 
larynx
 
 
distance
 
 
ADE
 
 
0.75
 
 
No
 
 
No
 
 
 
 
ANNF
 
 
skull
 
 
angle
 
 
ADE
 
 
0.74
 
 
No
 
 
No
 
 
 
 
PNNP
 
 
pharynx
 
 
distance
 
 
ADE
 
 
0.74
 
 
No
 
 
No
 
 
 
 
APSN
 
 
skull
 
 
angle
 
 
ADE
 
 
0.73
 
 
No
 
 
No
 
 
 
 
DICD
 
 
dentition
 
 
distance
 
 
ADE
 
 
0.71
 
 
No
 
 
No
 
 
 
 
SIOD
 
 
skull
 
 
distance
 
 
ADE
 
 
0.71
 
 
No
 
 
No
 
 
 
 
L5EP
 
 
larynx
 
 
distance
 
 
ADE
 
 
0.66
 
 
No
 
 
No
 
 
 
 
DAII
 
 
dentition
 
 
angle
 
 
ADE
 
 
0.66
 
 
No
 
 
No
 
 
 
 
L2EA
 
 
larynx
 
 
distance
 
 
ADE
 
 
0.65
 
 
No
 
 
No
 
 
 
 
DAIM
 
 
dentition
 
 
angle
 
 
ADE
 
 
0.63
 
 
No
 
 
No
 
 
 
 
DAIP
 
 
dentition
 
 
angle
 
 
ADE
 
 
0.62
 
 
No
 
 
No
 
 
 
 
HIPL
 
 
hard palate
 
 
distance
 
 
ADE
 
 
0.60
 
 
No
 
 
No
 
 
 
 
CS2H
 
 
cervical
 
 
distance
 
 
ADE
 
 
0.59
 
 
No
 
 
No
 
 
 
 
OPMR
 
 
oral
 
 
ratio
 
 
ADE
 
 
0.57
 
 
No
 
 
No
 
 
 
 
CS6H
 
 
cervical
 
 
distance
 
 
ADE
 
 
0.56
 
 
No
 
 
No
 
 
 
 
HDMP
 
 
hard palate
 
 
angle
 
 
ADE
 
 
0.56
 
 
No
 
 
No
 
 
 
 
SNOR
 
 
skull
 
 
distance
 
 
ADE
 
 
0.53
 
 
No
 
 
No
 
 
 
 
OCPR
 
 
oral
 
 
ratio
 
 
ADE
 
 
0.52
 
 
No
 
 
No
 
 
 
 
HPTH
 
 
hard palate
 
 
distance
 
 
ADE
 
 
0.52
 
 
No
 
 
No
 
 
 
 
LHEP
 
 
larynx
 
 
distance
 
 
ADE
 
 
0.47
 
 
No
 
 
No
 
 
 
 
HIMH
 
 
hard palate
 
 
distance
 
 
ADE
 
 
0.47
 
 
No
 
 
No
 
 
 
 
HMTH
 
 
hard palate
 
 
distance
 
 
ADE
 
 
0.46
 
 
No
 
 
No
 
 
 
 
L4CT
 
 
larynx
 
 
distance
 
 
ADE
 
 
0.46
 
 
No
 
 
No
 
 
 
 
L4EP
 
 
larynx
 
 
distance
 
 
ADE
 
 
0.44
 
 
No
 
 
No
 
 
 
 
LEPL
 
 
larynx
 
 
distance
 
 
ADE
 
 
0.40
 
 
No
 
 
No
 
 
 
 
AANP
 
 
skull
 
 
angle
 
 
ADE
 
 
0.40
 
 
No
 
 
No
 
 
 
 
HICL
 
 
hard palate
 
 
distance
 
 
ADE
 
 
0.40
 
 
No
 
 
No
 
 
 
 
HCPP
 
 
hard palate
 
 
Procrustes dist.
 
 
ADE
 
 
0.37
 
 
No
 
 
No
 
 
 
 
MMPG
 
 
mandible
 
 
distance
 
 
ADE
 
 
0.32
 
 
No
 
 
No
 
 
 
 
GRPD
 
 
general
 
 
Procrustes dist.
 
 
ADE
 
 
0.30
 
 
No
 
 
No
 
 
 
 
HP2C
 
 
hard palate
 
 
curvature
 
 
ADE
 
 
0.26
 
 
No
 
 
No
 
 
 
 
DDAP
 
 
dentition
 
 
Procrustes dist.
 
 
ADE
 
 
0.24
 
 
No
 
 
No
 
 
 
 
HCMP
 
 
hard palate
 
 
Procrustes dist.
 
 
ADE
 
 
0.23
 
 
No
 
 
No
 
 
 
 
DIIL
 
 
dentition
 
 
distance
 
 
ADE
 
 
0.23
 
 
No
 
 
No
 
 
 
 
HM2C
 
 
hard palate
 
 
curvature
 
 
ADE
 
 
0.23
 
 
No
 
 
No
 
 
 
 
PPW3
 
 
pharynx
 
 
curvature
 
 
ADE
 
 
0.19
 
 
No
 
 
No
 
 
 
 
PPW2
 
 
pharynx
 
 
curvature
 
 
ADE
 
 
0.09
 
 
No
 
 
No
 
 
 
 
HMAC
 
 
hard palate
 
 
curvature
 
 
ADE
 
 
0.08
 
 
No
 
 
No
 
 
 
 
 
 
 The standardized and unstandardied estimates 
 
  ACE  
 
 All phenotypes 
 
 Unstandardized components 
 
 
 
  Figure 31.     A ,  C  and
 E  components (with 95% CI) for all measures for which
 ACE  is the best-fitting model ordered by decreasing  A 
estimate. 
 
 
 
 
 Standardized components 
 
 
 
  Figure 32.     h  2  (aka
 a  2 ),  c  2  and
 e  2  components (with 95% CI) for all measures for
which  ACE  is the best-fitting model ordered by decreasing
 h  2  estimate. 
 
 
 
 
 
 Inter-rater reliable phenotypes 
 
 
 
  Figure 33.     h  2  (aka
 a  2 ),  c  2 ,  F  2 
and  e  2  components (with 95% CI) for all measures for
which  ACE  is the best-fitting model ordered by decreasing
 h  2  estimate, only for the measures with good
inter-rater agreement (i.e.,  ICC(C,1)  >= 0.75). 
 
 
 
 
 
  ADE  
 
 All phenotypes 
 
 Unstandardized components 
 
 
 
  Figure 34.     A ,  D  and
 E  components (with 95% CI) for all measures for which
 ACE  is the best-fitting model ordered by decreasing  A 
estimate. 
 
 
 
 
 Standardized components 
 
 
 
  Figure 35.     h  2  (aka
 a  2 ),  d  2 ,  H  2 ,
and  e  2  components (with 95% CI) for all measures for
which  ACE  is the best-fitting model ordered by decreasing
 h  2  estimate. 
 
 
 
 
 
 Inter-rater reliable phenotypes 
 
 
 
  Figure 36.     h  2  (aka
 a  2 ),  d  2 ,  H  2 
and  e  2  components (with 95% CI) for all measures for
which  ACE  is the best-fitting model ordered by decreasing
 h  2  estimate, only for the measures with good
inter-rater agreement (i.e.,  ICC(C,1)  >= 0.75). 
 
 
 
 
 
 Both  ACE  and  ADE  together 
 
 All measures 
 
 Unstandardized components 
 
 
 
  Figure 37.     A ,
 C / D  and  E  components (with 95% CI) for all
measures ordered by decreasing  A  estimate. 
 
 
 
 
 Standardized components 
 
 
 
  Figure 38.     h  2  (aka
 a  2 ),  d  2 ,  H  2 ,
and  e  2  components (with 95% CI) for all measures for
which  ACE  is the best-fitting model ordered by decreasing
 h  2  estimate. 
 
 
 
 
 
 Inter-rater reliable measures 
 
 
 
  Figure 39.     h  2  (aka
 a  2 ),  d  2 ,  H  2 ,
and  e  2  components (with 95% CI) for all measures for
which  ACE  is the best-fitting model ordered by decreasing
 h  2  estimate. 
 
 
 
 
 
 Measures by components 
 The significant contribution of a component is judged from the AIC
and likelihood ratio test (LRT) against the model with that component
fixed to 0.0 (i.e., for the ΔAIC this must be > 2, and for the test,
 p  < 0.05 – we did not apply any multiple testing correction
here). 
 
  A  and  h  2  
 Relationship between the significance of  A  judged by AIC and
LRT is perfect: 
 
 
 
 
 
 
 
 
   
 FALSE 
 TRUE 
 
 
 
 
  FALSE  
 121 
 0 
 
 
  TRUE  
 0 
 25 
 
 
 
 but that between AIC (or, equivalently, LRT) and the inclusion of 0
within the 95%CI of  h  2  is not, with the latter being
more liberal (i.e., finding more measures with a significant
 h  2 ): 
 
 
 
 
 
 
 
 
   
 FALSE 
 TRUE 
 
 
 
 
  FALSE  
 60 
 61 
 
 
  TRUE  
 0 
 25 
 
 
 
 
 
Measures with a significant  A  component, ordered by the
decreasing strength of evidence for  A , inter-rater agreement
and estimate of  h  2 . In  dark blue  measures
with a significant  A  component after Holm’s correction (14),
while in  blue  those only nominally significant (11); in
 bold  measures with  ICC(C,1)  > 0.75 (44), in
 bold italic  mesures with the lower 95%CI of  ICC(C,1) 
> 0.75 (37); in gray those with  h  2  < 0.20
(61). Column  h  2  > 0 is TRUE if and only if 0 is
not included in the 95% CI of  h  2  (this is a much
less strict test than ΔAIC and LTR).
 
 
 
 
measure
 
 
domain
 
 
type
 
 
 ICC ( C ,1)
 
 
genetic model
 
 
 A 
 
 
ΔAIC
 
 
 p 
 
 
 p (Holm)
 
 
 h  2 
 
 
 h  2 >0
 
 
 
 
 
 
 MICD 
 
 
mandible
 
 
distance
 
 
0.98 (0.98, 0.99)
 
 
ACE
 
 
0.46 (0.27, 0.55)
 
 
51.51
 
 
0.0000
 
 
0.0000
 
 
0.87 (0.51, 0.90)
 
 
TRUE
 
 
 
 
 SHBN 
 
 
skull
 
 
distance
 
 
0.96 (0.95, 0.96)
 
 
ACE
 
 
0.34 (0.19, 0.57)
 
 
33.99
 
 
0.0000
 
 
0.0000
 
 
0.56 (0.31, 0.91)
 
 
TRUE
 
 
 
 
 MIGD 
 
 
mandible
 
 
distance
 
 
0.95 (0.94, 0.95)
 
 
ACE
 
 
0.31 (0.17, 0.45)
 
 
31.18
 
 
0.0000
 
 
0.0000
 
 
0.69 (0.39, 0.91)
 
 
TRUE
 
 
 
 
 SBAN 
 
 
skull
 
 
distance
 
 
0.89 (0.88, 0.91)
 
 
ACE
 
 
0.38 (0.34, 0.58)
 
 
25.36
 
 
0.0000
 
 
0.0000
 
 
0.66 (0.36, 0.92)
 
 
TRUE
 
 
 
 
 SNAM 
 
 
skull
 
 
distance
 
 
0.90 (0.89, 0.92)
 
 
ACE
 
 
0.46 (0.24, 0.59)
 
 
22.38
 
 
0.0000
 
 
0.0001
 
 
0.78 (0.40, 0.88)
 
 
TRUE
 
 
 
 
 SNAP 
 
 
skull
 
 
distance
 
 
0.88 (0.86, 0.90)
 
 
ACE
 
 
0.46 (0.23, 0.58)
 
 
22.03
 
 
0.0000
 
 
0.0001
 
 
0.79 (0.40, 0.88)
 
 
TRUE
 
 
 
 
 MCGP 
 
 
mandible
 
 
angle
 
 
0.87 (0.84, 0.88)
 
 
ACE
 
 
0.61 (0.30, 0.80)
 
 
19.77
 
 
0.0000
 
 
0.0004
 
 
0.74 (0.38, 0.86)
 
 
TRUE
 
 
 
 
 SSEG 
 
 
skull
 
 
distance
 
 
0.90 (0.88, 0.91)
 
 
ACE
 
 
0.59 (0.29, 0.72)
 
 
18.76
 
 
0.0000
 
 
0.0007
 
 
0.77 (0.38, 0.83)
 
 
TRUE
 
 
 
 
 SBNA 
 
 
skull
 
 
distance
 
 
0.90 (0.88, 0.91)
 
 
ACE
 
 
0.44 (0.21, 0.64)
 
 
18.57
 
 
0.0000
 
 
0.0008
 
 
0.67 (0.32, 0.87)
 
 
TRUE
 
 
 
 
 MCGM 
 
 
mandible
 
 
angle
 
 
0.89 (0.87, 0.90)
 
 
ACE
 
 
0.56 (0.27, 0.80)
 
 
16.46
 
 
0.0000
 
 
0.0024
 
 
0.67 (0.43, 0.85)
 
 
TRUE
 
 
 
 
 ASCG* 
 
 
skull
 
 
angle
 
 
0.87 (0.85, 0.89)
 
 
ACE
 
 
0.56 (0.26, 0.80)
 
 
16.46
 
 
0.0000
 
 
0.0024
 
 
0.67 (0.41, 0.85)
 
 
TRUE
 
 
 
 
 SNNP 
 
 
skull
 
 
distance
 
 
0.82 (0.80, 0.85)
 
 
ACE
 
 
0.33 (0.15, 0.55)
 
 
16.01
 
 
0.0000
 
 
0.0030
 
 
0.62 (0.28, 0.95)
 
 
TRUE
 
 
 
 
 SHSW 
 
 
skull
 
 
distance
 
 
0.94 (0.93, 0.95)
 
 
ACE
 
 
0.26 (0.12, 0.40)
 
 
15.32
 
 
0.0000
 
 
0.0042
 
 
0.63 (0.28, 0.86)
 
 
TRUE
 
 
 
 
 SSEN 
 
 
skull
 
 
distance
 
 
0.90 (0.89, 0.92)
 
 
ACE
 
 
0.31 (0.13, 0.57)
 
 
13.24
 
 
0.0001
 
 
0.0126
 
 
0.47 (0.19, 0.87)
 
 
TRUE
 
 
 
 
 ASNP 
 
 
skull
 
 
angle
 
 
0.72 (0.68, 0.76)
 
 
ACE
 
 
0.50 (0.18, 0.69)
 
 
9.58
 
 
0.0007
 
 
0.0881
 
 
0.73 (0.26, 0.91)
 
 
TRUE
 
 
 
 
 APNS 
 
 
skull
 
 
angle
 
 
0.83 (0.80, 0.85)
 
 
ACE
 
 
0.56 (0.22, 0.69)
 
 
9.45
 
 
0.0007
 
 
0.0934
 
 
0.73 (0.25, 0.81)
 
 
TRUE
 
 
 
 
 CS2A 
 
 
cervical
 
 
distance
 
 
0.60 (0.55, 0.65)
 
 
ACE
 
 
0.35 (0.12, 0.48)
 
 
9.45
 
 
0.0007
 
 
0.0934
 
 
0.88 (0.36, 1.00)
 
 
TRUE
 
 
 
 
 MCGD 
 
 
mandible
 
 
distance
 
 
0.71 (0.67, 0.75)
 
 
ACE
 
 
0.34 (0.14, 0.43)
 
 
9.39
 
 
0.0007
 
 
0.0951
 
 
0.80 (0.31, 0.91)
 
 
TRUE
 
 
 
 
 HBPL 
 
 
hard palate
 
 
distance
 
 
0.91 (0.89, 0.92)
 
 
ACE
 
 
0.27 (0.00, 0.55)
 
 
7.47
 
 
0.0021
 
 
0.2667
 
 
0.38 (0.12, 0.78)
 
 
TRUE
 
 
 
 
 AASN 
 
 
skull
 
 
angle
 
 
0.63 (0.58, 0.67)
 
 
ACE
 
 
0.38 (0.13, 0.59)
 
 
5.26
 
 
0.0071
 
 
0.8956
 
 
0.66 (0.17, 0.92)
 
 
TRUE
 
 
 
 
 HBNP 
 
 
hyoid
 
 
distance
 
 
0.84 (0.81, 0.86)
 
 
ACE
 
 
0.24 (0.05, 0.37)
 
 
4.20
 
 
0.0128
 
 
1.0000
 
 
0.57 (0.11, 0.78)
 
 
TRUE
 
 
 
 
 HBC4 
 
 
hyoid
 
 
distance
 
 
0.79 (0.76, 0.82)
 
 
ACE
 
 
0.39 (0.03, 0.61)
 
 
2.60
 
 
0.0319
 
 
1.0000
 
 
0.52 (0.04, 0.72)
 
 
TRUE
 
 
 
 
 SVTh* 
 
 
general
 
 
distance
 
 
0.93 (0.91, 0.94)
 
 
ACE
 
 
0.21 (0.00, 0.48)
 
 
2.84
 
 
0.0278
 
 
1.0000
 
 
0.26 (0.03, 0.62)
 
 
TRUE
 
 
 
 
 SNOL 
 
 
skull
 
 
distance
 
 
0.54 (0.48, 0.59)
 
 
ACE
 
 
0.25 (0.03, 0.44)
 
 
3.20
 
 
0.0226
 
 
1.0000
 
 
0.69 (0.09, 1.00)
 
 
TRUE
 
 
 
 
 MCGR 
 
 
mandible
 
 
distance
 
 
0.68 (0.64, 0.72)
 
 
ACE
 
 
0.25 (0.02, 0.39)
 
 
2.58
 
 
0.0323
 
 
1.0000
 
 
0.59 (0.05, 0.82)
 
 
TRUE
 
 
 
 
 SBAS 
 
 
skull
 
 
distance
 
 
0.79 (0.76, 0.82)
 
 
ADE
 
 
0.49 (0.00, 0.61)
 
 
0.53
 
 
0.1115
 
 
1.0000
 
 
0.78 (0.00, 0.86)
 
 
TRUE
 
 
 
 
 ANSF 
 
 
skull
 
 
angle
 
 
0.79 (0.75, 0.81)
 
 
ADE
 
 
0.52 (0.00, 0.68)
 
 
-0.49
 
 
0.2187
 
 
1.0000
 
 
0.73 (0.00, 0.84)
 
 
FALSE
 
 
 
 
 SBNP 
 
 
skull
 
 
distance
 
 
0.80 (0.77, 0.83)
 
 
ADE
 
 
0.46 (0.00, 0.69)
 
 
-0.72
 
 
0.2571
 
 
1.0000
 
 
0.65 (0.00, 0.86)
 
 
TRUE
 
 
 
 
 SSEN* 
 
 
skull
 
 
distance
 
 
0.90 (0.88, 0.91)
 
 
ADE
 
 
0.29 (0.00, 0.58)
 
 
-1.27
 
 
0.3933
 
 
1.0000
 
 
0.46 (0.00, 0.83)
 
 
TRUE
 
 
 
 
 HBPG 
 
 
hyoid
 
 
distance
 
 
0.81 (0.78, 0.83)
 
 
ACE
 
 
0.27 (0.00, 0.53)
 
 
0.11
 
 
0.1468
 
 
1.0000
 
 
0.37 (0.00, 0.68)
 
 
TRUE
 
 
 
 
 ABSN 
 
 
skull
 
 
angle
 
 
0.82 (0.79, 0.84)
 
 
ADE
 
 
0.29 (0.00, 0.76)
 
 
-1.54
 
 
0.4954
 
 
1.0000
 
 
0.36 (0.00, 0.84)
 
 
FALSE
 
 
 
 
 DIPD 
 
 
dentition
 
 
distance
 
 
0.80 (0.77, 0.82)
 
 
ADE
 
 
0.23 (0.00, 0.57)
 
 
-1.67
 
 
0.5682
 
 
1.0000
 
 
0.33 (0.00, 0.72)
 
 
TRUE
 
 
 
 
 ACSN 
 
 
skull
 
 
angle
 
 
0.87 (0.85, 0.89)
 
 
ADE
 
 
0.25 (0.00, 0.77)
 
 
-1.68
 
 
0.5739
 
 
1.0000
 
 
0.30 (0.00, 0.84)
 
 
FALSE
 
 
 
 
 MCPD 
 
 
mandible
 
 
distance
 
 
0.92 (0.90, 0.93)
 
 
ADE
 
 
0.14 (0.00, 0.51)
 
 
-1.75
 
 
0.6150
 
 
1.0000
 
 
0.26 (0.00, 0.86)
 
 
TRUE
 
 
 
 
 MPGR 
 
 
mandible
 
 
distance
 
 
0.84 (0.81, 0.86)
 
 
ADE
 
 
0.14 (0.00, 0.54)
 
 
-1.82
 
 
0.6679
 
 
1.0000
 
 
0.24 (0.00, 0.88)
 
 
TRUE
 
 
 
 
 SVTv* 
 
 
general
 
 
distance
 
 
0.80 (0.77, 0.83)
 
 
ACE
 
 
0.11 (0.00, 0.34)
 
 
-0.96
 
 
0.3074
 
 
1.0000
 
 
0.24 (0.00, 0.74)
 
 
TRUE
 
 
 
 
 SVTh 
 
 
general
 
 
distance
 
 
0.90 (0.88, 0.91)
 
 
ACE
 
 
0.13 (0.00, 0.42)
 
 
-0.54
 
 
0.2270
 
 
1.0000
 
 
0.17 (0.00, 0.53)
 
 
TRUE
 
 
 
 
 LACT 
 
 
larynx
 
 
distance
 
 
0.81 (0.78, 0.83)
 
 
ACE
 
 
0.09 (0.00, 0.38)
 
 
-1.46
 
 
0.4633
 
 
1.0000
 
 
0.17 (0.00, 0.69)
 
 
TRUE
 
 
 
 
 MPGL 
 
 
mandible
 
 
distance
 
 
0.84 (0.82, 0.86)
 
 
ADE
 
 
0.08 (0.00, 0.48)
 
 
-1.93
 
 
0.7983
 
 
1.0000
 
 
0.15 (0.00, 0.83)
 
 
TRUE
 
 
 
 
 MCMD 
 
 
mandible
 
 
distance
 
 
0.92 (0.90, 0.93)
 
 
ADE
 
 
0.05 (0.00, 0.51)
 
 
-1.97
 
 
0.8518
 
 
1.0000
 
 
0.09 (0.00, 0.86)
 
 
TRUE
 
 
 
 
 MGPD 
 
 
mandible
 
 
distance
 
 
0.83 (0.80, 0.85)
 
 
ADE
 
 
0.03 (0.00, 0.61)
 
 
-1.99
 
 
0.9329
 
 
1.0000
 
 
0.05 (0.00, 0.87)
 
 
FALSE
 
 
 
 
 GAPD 
 
 
general
 
 
Procrustes dist.
 
 
0.80 (0.77, 0.82)
 
 
ACE
 
 
0.00 (0.00, 0.23)
 
 
-2.00
 
 
1.0000
 
 
1.0000
 
 
0.00 (0.00, 0.34)
 
 
FALSE
 
 
 
 
 MGMD 
 
 
mandible
 
 
distance
 
 
0.80 (0.77, 0.83)
 
 
ADE
 
 
0.00 (0.00, 0.41)
 
 
-2.00
 
 
1.0000
 
 
1.0000
 
 
0.00 (0.00, 0.65)
 
 
FALSE
 
 
 
 
 L4EA 
 
 
larynx
 
 
distance
 
 
0.76 (0.72, 0.79)
 
 
ACE
 
 
0.31 (0.00, 0.55)
 
 
0.72
 
 
0.0994
 
 
1.0000
 
 
0.43 (0.00, 0.69)
 
 
TRUE
 
 
 
 
 HBC2 
 
 
hyoid
 
 
distance
 
 
0.78 (0.74, 0.81)
 
 
ACE
 
 
0.14 (0.00, 0.33)
 
 
-0.33
 
 
0.1964
 
 
1.0000
 
 
0.35 (0.00, 0.73)
 
 
TRUE
 
 
 
 
 HNSL 
 
 
hard palate
 
 
distance
 
 
0.77 (0.74, 0.80)
 
 
ACE
 
 
0.19 (0.00, 0.49)
 
 
0.97
 
 
0.0848
 
 
1.0000
 
 
0.31 (0.00, 0.78)
 
 
TRUE
 
 
 
 
 DIMD 
 
 
dentition
 
 
distance
 
 
0.77 (0.73, 0.80)
 
 
ACE
 
 
0.18 (0.00, 0.50)
 
 
-0.33
 
 
0.1967
 
 
1.0000
 
 
0.29 (0.00, 0.75)
 
 
TRUE
 
 
 
 
 LAEA 
 
 
larynx
 
 
distance
 
 
0.77 (0.73, 0.80)
 
 
ACE
 
 
0.16 (0.00, 0.38)
 
 
-1.27
 
 
0.3944
 
 
1.0000
 
 
0.27 (0.00, 0.61)
 
 
TRUE
 
 
 
 
 L3EA 
 
 
larynx
 
 
distance
 
 
0.76 (0.72, 0.79)
 
 
ADE
 
 
0.02 (0.00, 0.50)
 
 
-2.00
 
 
0.9736
 
 
1.0000
 
 
0.02 (0.00, 0.63)
 
 
FALSE
 
 
 
 
 APNP 
 
 
skull
 
 
angle
 
 
0.76 (0.73, 0.79)
 
 
ADE
 
 
0.00 (0.00, 0.61)
 
 
-2.00
 
 
1.0000
 
 
1.0000
 
 
0.00 (0.00, 0.75)
 
 
FALSE
 
 
 
 
 SNOR 
 
 
skull
 
 
distance
 
 
0.53 (0.47, 0.58)
 
 
ADE
 
 
0.32 (0.00, 0.43)
 
 
-0.88
 
 
0.2903
 
 
1.0000
 
 
0.91 (0.00, 1.00)
 
 
TRUE
 
 
 
 
 ANNF 
 
 
skull
 
 
angle
 
 
0.74 (0.71, 0.78)
 
 
ADE
 
 
0.48 (0.00, 0.63)
 
 
-0.94
 
 
0.3032
 
 
1.0000
 
 
0.67 (0.00, 0.80)
 
 
TRUE
 
 
 
 
 CS6H 
 
 
cervical
 
 
distance
 
 
0.56 (0.50, 0.61)
 
 
ADE
 
 
0.30 (0.00, 0.40)
 
 
-1.42
 
 
0.4454
 
 
1.0000
 
 
0.65 (0.00, 0.78)
 
 
TRUE
 
 
 
 
 HICL 
 
 
hard palate
 
 
distance
 
 
0.40 (0.33, 0.46)
 
 
ADE
 
 
0.22 (0.00, 0.34)
 
 
-1.69
 
 
0.5781
 
 
1.0000
 
 
0.60 (0.00, 0.87)
 
 
TRUE
 
 
 
 
 SIOD 
 
 
skull
 
 
distance
 
 
0.71 (0.67, 0.74)
 
 
ADE
 
 
0.26 (0.00, 0.51)
 
 
-1.10
 
 
0.3438
 
 
1.0000
 
 
0.58 (0.00, 1.00)
 
 
FALSE
 
 
 
 
 DIIL 
 
 
dentition
 
 
distance
 
 
0.23 (0.15, 0.30)
 
 
ADE
 
 
0.13 (0.00, 0.23)
 
 
-1.84
 
 
0.6878
 
 
1.0000
 
 
0.58 (0.00, 1.00)
 
 
TRUE
 
 
 
 
 CS5H 
 
 
cervical
 
 
distance
 
 
0.43 (0.37, 0.50)
 
 
ACE
 
 
0.20 (0.00, 0.31)
 
 
-0.68
 
 
0.2510
 
 
1.0000
 
 
0.55 (0.00, 0.79)
 
 
TRUE
 
 
 
 
 PPW6 
 
 
pharynx
 
 
curvature
 
 
0.43 (0.37, 0.50)
 
 
ACE
 
 
0.16 (0.00, 0.29)
 
 
-0.70
 
 
0.2546
 
 
1.0000
 
 
0.54 (0.00, 0.92)
 
 
TRUE
 
 
 
 
 APSN 
 
 
skull
 
 
angle
 
 
0.73 (0.69, 0.76)
 
 
ADE
 
 
0.38 (0.00, 0.72)
 
 
-1.22
 
 
0.3764
 
 
1.0000
 
 
0.54 (0.00, 0.91)
 
 
TRUE
 
 
 
 
 LHCT 
 
 
larynx
 
 
distance
 
 
0.66 (0.62, 0.70)
 
 
ACE
 
 
0.25 (0.00, 0.37)
 
 
0.26
 
 
0.1326
 
 
1.0000
 
 
0.54 (0.00, 0.74)
 
 
TRUE
 
 
 
 
 HBC3 
 
 
hyoid
 
 
distance
 
 
0.71 (0.67, 0.74)
 
 
ACE
 
 
0.27 (0.00, 0.42)
 
 
1.42
 
 
0.0643
 
 
1.0000
 
 
0.54 (0.00, 0.76)
 
 
TRUE
 
 
 
 
 L4EP 
 
 
larynx
 
 
distance
 
 
0.44 (0.38, 0.50)
 
 
ADE
 
 
0.20 (0.00, 0.31)
 
 
-1.74
 
 
0.6070
 
 
1.0000
 
 
0.49 (0.00, 0.72)
 
 
TRUE
 
 
 
 
 MCGL 
 
 
mandible
 
 
distance
 
 
0.68 (0.64, 0.72)
 
 
ACE
 
 
0.21 (0.00, 0.37)
 
 
0.92
 
 
0.0875
 
 
1.0000
 
 
0.48 (0.00, 0.78)
 
 
TRUE
 
 
 
 
 HIMH 
 
 
hard palate
 
 
distance
 
 
0.47 (0.41, 0.53)
 
 
ADE
 
 
0.19 (0.00, 0.29)
 
 
-1.70
 
 
0.5836
 
 
1.0000
 
 
0.48 (0.00, 0.67)
 
 
TRUE
 
 
 
 
 L5EP 
 
 
larynx
 
 
distance
 
 
0.66 (0.61, 0.70)
 
 
ADE
 
 
0.30 (0.00, 0.46)
 
 
-1.51
 
 
0.4847
 
 
1.0000
 
 
0.47 (0.00, 0.67)
 
 
FALSE
 
 
 
 
 OPMR 
 
 
oral
 
 
ratio
 
 
0.57 (0.52, 0.62)
 
 
ADE
 
 
0.25 (0.00, 0.41)
 
 
-1.68
 
 
0.5714
 
 
1.0000
 
 
0.46 (0.00, 0.69)
 
 
FALSE
 
 
 
 
 SPUN 
 
 
soft palate
 
 
distance
 
 
0.63 (0.58, 0.67)
 
 
ACE
 
 
0.25 (0.00, 0.47)
 
 
0.23
 
 
0.1351
 
 
1.0000
 
 
0.45 (0.00, 0.77)
 
 
TRUE
 
 
 
 
 HIML 
 
 
hard palate
 
 
distance
 
 
0.63 (0.58, 0.67)
 
 
ACE
 
 
0.21 (0.00, 0.43)
 
 
0.21
 
 
0.1369
 
 
1.0000
 
 
0.44 (0.00, 0.81)
 
 
TRUE
 
 
 
 
 L5EA 
 
 
larynx
 
 
distance
 
 
0.74 (0.70, 0.77)
 
 
ACE
 
 
0.29 (0.00, 0.52)
 
 
0.49
 
 
0.1144
 
 
1.0000
 
 
0.43 (0.00, 0.70)
 
 
TRUE
 
 
 
 
 OPHW 
 
 
oral
 
 
ratio
 
 
0.50 (0.43, 0.55)
 
 
ACE
 
 
0.19 (0.00, 0.33)
 
 
-0.97
 
 
0.3098
 
 
1.0000
 
 
0.42 (0.00, 0.66)
 
 
TRUE
 
 
 
 
 L2EA 
 
 
larynx
 
 
distance
 
 
0.65 (0.61, 0.70)
 
 
ADE
 
 
0.21 (0.00, 0.35)
 
 
-1.77
 
 
0.6311
 
 
1.0000
 
 
0.40 (0.00, 0.63)
 
 
TRUE
 
 
 
 
 SNNA 
 
 
skull
 
 
distance
 
 
0.66 (0.61, 0.70)
 
 
ACE
 
 
0.13 (0.00, 0.41)
 
 
-0.44
 
 
0.2117
 
 
1.0000
 
 
0.31 (0.00, 0.92)
 
 
TRUE
 
 
 
 
 DICD 
 
 
dentition
 
 
distance
 
 
0.71 (0.67, 0.75)
 
 
ADE
 
 
0.19 (0.00, 0.51)
 
 
-1.81
 
 
0.6626
 
 
1.0000
 
 
0.31 (0.00, 0.74)
 
 
TRUE
 
 
 
 
 DSIL 
 
 
dentition
 
 
distance
 
 
0.45 (0.39, 0.51)
 
 
ACE
 
 
0.12 (0.00, 0.34)
 
 
-1.48
 
 
0.4696
 
 
1.0000
 
 
0.30 (0.00, 0.82)
 
 
FALSE
 
 
 
 
 OMHW 
 
 
oral
 
 
ratio
 
 
0.48 (0.42, 0.54)
 
 
ACE
 
 
0.14 (0.00, 0.32)
 
 
-1.55
 
 
0.5010
 
 
1.0000
 
 
0.30 (0.00, 0.63)
 
 
TRUE
 
 
 
 
 OMHL 
 
 
oral
 
 
ratio
 
 
0.44 (0.37, 0.50)
 
 
ACE
 
 
0.12 (0.00, 0.25)
 
 
-1.61
 
 
0.5309
 
 
1.0000
 
 
0.30 (0.00, 0.60)
 
 
FALSE
 
 
 
 
 PPW7 
 
 
pharynx
 
 
curvature
 
 
0.71 (0.67, 0.74)
 
 
ACE
 
 
0.15 (0.00, 0.44)
 
 
-0.67
 
 
0.2487
 
 
1.0000
 
 
0.30 (0.00, 0.80)
 
 
TRUE
 
 
 
 
 OCMR 
 
 
oral
 
 
ratio
 
 
0.47 (0.40, 0.53)
 
 
ACE
 
 
0.13 (0.00, 0.34)
 
 
-1.53
 
 
0.4918
 
 
1.0000
 
 
0.29 (0.00, 0.70)
 
 
TRUE
 
 
 
 
 L3EP 
 
 
larynx
 
 
distance
 
 
0.57 (0.52, 0.62)
 
 
ACE
 
 
0.14 (0.00, 0.38)
 
 
-1.30
 
 
0.4015
 
 
1.0000
 
 
0.29 (0.00, 0.72)
 
 
TRUE
 
 
 
 
 L2EP 
 
 
larynx
 
 
distance
 
 
0.67 (0.62, 0.71)
 
 
ACE
 
 
0.15 (0.00, 0.40)
 
 
-1.16
 
 
0.3589
 
 
1.0000
 
 
0.28 (0.00, 0.71)
 
 
TRUE
 
 
 
 
 OCPR 
 
 
oral
 
 
ratio
 
 
0.52 (0.46, 0.57)
 
 
ADE
 
 
0.14 (0.00, 0.31)
 
 
-1.91
 
 
0.7656
 
 
1.0000
 
 
0.27 (0.00, 0.56)
 
 
TRUE
 
 
 
 
 HIPH 
 
 
hard palate
 
 
distance
 
 
0.43 (0.36, 0.49)
 
 
ACE
 
 
0.11 (0.00, 0.30)
 
 
-1.69
 
 
0.5761
 
 
1.0000
 
 
0.26 (0.00, 0.72)
 
 
FALSE
 
 
 
 
 HARC 
 
 
hard palate
 
 
angle
 
 
0.36 (0.29, 0.42)
 
 
ACE
 
 
0.09 (0.00, 0.20)
 
 
-1.74
 
 
0.6133
 
 
1.0000
 
 
0.26 (0.00, 0.59)
 
 
TRUE
 
 
 
 
 PPWC 
 
 
pharynx
 
 
curvature
 
 
0.27 (0.19, 0.34)
 
 
ACE
 
 
0.06 (0.00, 0.19)
 
 
-1.85
 
 
0.7021
 
 
1.0000
 
 
0.26 (0.00, 0.88)
 
 
FALSE
 
 
 
 
 ALNS 
 
 
skull
 
 
angle
 
 
0.60 (0.55, 0.65)
 
 
ACE
 
 
0.15 (0.00, 0.50)
 
 
-1.29
 
 
0.3995
 
 
1.0000
 
 
0.26 (0.00, 0.80)
 
 
TRUE
 
 
 
 
 CS7H 
 
 
cervical
 
 
distance
 
 
0.69 (0.64, 0.72)
 
 
ACE
 
 
0.15 (0.00, 0.48)
 
 
-1.20
 
 
0.3724
 
 
1.0000
 
 
0.25 (0.00, 0.74)
 
 
TRUE
 
 
 
 
 LNEA 
 
 
larynx
 
 
distance
 
 
0.75 (0.71, 0.78)
 
 
ADE
 
 
0.14 (0.00, 0.38)
 
 
-1.86
 
 
0.7051
 
 
1.0000
 
 
0.25 (0.00, 0.63)
 
 
TRUE
 
 
 
 
 DAIS 
 
 
dentition
 
 
angle
 
 
0.65 (0.60, 0.69)
 
 
ACE
 
 
0.15 (0.00, 0.36)
 
 
-1.65
 
 
0.5555
 
 
1.0000
 
 
0.24 (0.00, 0.54)
 
 
TRUE
 
 
 
 
 HIML* 
 
 
hard palate
 
 
distance
 
 
0.65 (0.60, 0.69)
 
 
ACE
 
 
0.13 (0.00, 0.44)
 
 
-1.21
 
 
0.3745
 
 
1.0000
 
 
0.24 (0.00, 0.78)
 
 
TRUE
 
 
 
 
 LHEA 
 
 
larynx
 
 
distance
 
 
0.61 (0.56, 0.66)
 
 
ACE
 
 
0.09 (0.00, 0.22)
 
 
-1.77
 
 
0.6304
 
 
1.0000
 
 
0.23 (0.00, 0.52)
 
 
TRUE
 
 
 
 
 HM2C 
 
 
hard palate
 
 
curvature
 
 
0.23 (0.15, 0.30)
 
 
ADE
 
 
0.05 (0.00, 0.18)
 
 
-1.98
 
 
0.8799
 
 
1.0000
 
 
0.22 (0.00, 0.91)
 
 
FALSE
 
 
 
 
 OPHL 
 
 
oral
 
 
ratio
 
 
0.38 (0.31, 0.44)
 
 
ACE
 
 
0.08 (0.00, 0.26)
 
 
-1.85
 
 
0.6986
 
 
1.0000
 
 
0.22 (0.00, 0.67)
 
 
TRUE
 
 
 
 
 OCLW 
 
 
oral
 
 
ratio
 
 
0.25 (0.17, 0.32)
 
 
ACE
 
 
0.06 (0.00, 0.14)
 
 
-1.90
 
 
0.7532
 
 
1.0000
 
 
0.21 (0.00, 0.50)
 
 
FALSE
 
 
 
 
 CS3H 
 
 
cervical
 
 
distance
 
 
0.60 (0.54, 0.64)
 
 
ACE
 
 
0.07 (0.00, 0.34)
 
 
-1.62
 
 
0.5358
 
 
1.0000
 
 
0.20 (0.00, 0.89)
 
 
TRUE
 
 
 
 
 OCHW 
 
 
oral
 
 
ratio
 
 
0.41 (0.34, 0.47)
 
 
ACE
 
 
0.07 (0.00, 0.29)
 
 
-1.79
 
 
0.6499
 
 
1.0000
 
 
0.20 (0.00, 0.72)
 
 
TRUE
 
 
 
 
 HCTH 
 
 
hard palate
 
 
distance
 
 
0.44 (0.37, 0.50)
 
 
ACE
 
 
0.06 (0.00, 0.26)
 
 
-1.86
 
 
0.7132
 
 
1.0000
 
 
0.19 (0.00, 0.73)
 
 
TRUE
 
 
 
 
 HICH 
 
 
hard palate
 
 
distance
 
 
0.39 (0.32, 0.46)
 
 
ACE
 
 
0.06 (0.00, 0.27)
 
 
-1.86
 
 
0.7118
 
 
1.0000
 
 
0.17 (0.00, 0.68)
 
 
TRUE
 
 
 
 
 HNAP 
 
 
hard palate
 
 
distance
 
 
0.37 (0.30, 0.44)
 
 
ACE
 
 
0.06 (0.00, 0.30)
 
 
-1.90
 
 
0.7467
 
 
1.0000
 
 
0.17 (0.00, 0.39)
 
 
TRUE
 
 
 
 
 HIPL* 
 
 
hard palate
 
 
distance
 
 
0.63 (0.58, 0.67)
 
 
ACE
 
 
0.09 (0.00, 0.40)
 
 
-1.77
 
 
0.6348
 
 
1.0000
 
 
0.15 (0.00, 0.68)
 
 
FALSE
 
 
 
 
 OPLW 
 
 
oral
 
 
ratio
 
 
0.50 (0.44, 0.56)
 
 
ACE
 
 
0.07 (0.00, 0.29)
 
 
-1.86
 
 
0.7104
 
 
1.0000
 
 
0.15 (0.00, 0.61)
 
 
TRUE
 
 
 
 
 HICL* 
 
 
hard palate
 
 
distance
 
 
0.39 (0.32, 0.45)
 
 
ACE
 
 
0.04 (0.00, 0.26)
 
 
-1.94
 
 
0.8037
 
 
1.0000
 
 
0.14 (0.00, 0.80)
 
 
TRUE
 
 
 
 
 HMTH 
 
 
hard palate
 
 
distance
 
 
0.46 (0.40, 0.52)
 
 
ADE
 
 
0.06 (0.00, 0.35)
 
 
-1.97
 
 
0.8641
 
 
1.0000
 
 
0.14 (0.00, 0.77)
 
 
TRUE
 
 
 
 
 L5CT 
 
 
larynx
 
 
distance
 
 
0.71 (0.67, 0.75)
 
 
ACE
 
 
0.08 (0.00, 0.42)
 
 
-1.73
 
 
0.6066
 
 
1.0000
 
 
0.13 (0.00, 0.65)
 
 
FALSE
 
 
 
 
 HAPL 
 
 
hard palate
 
 
distance
 
 
0.52 (0.46, 0.57)
 
 
ACE
 
 
0.06 (0.00, 0.31)
 
 
-1.92
 
 
0.7836
 
 
1.0000
 
 
0.12 (0.00, 0.61)
 
 
FALSE
 
 
 
 
 PPW4 
 
 
pharynx
 
 
curvature
 
 
0.31 (0.24, 0.38)
 
 
ACE
 
 
0.03 (0.00, 0.20)
 
 
-1.97
 
 
0.8625
 
 
1.0000
 
 
0.10 (0.00, 0.71)
 
 
FALSE
 
 
 
 
 OMLW 
 
 
oral
 
 
ratio
 
 
0.70 (0.65, 0.73)
 
 
ACE
 
 
0.05 (0.00, 0.36)
 
 
-1.88
 
 
0.7280
 
 
1.0000
 
 
0.09 (0.00, 0.68)
 
 
TRUE
 
 
 
 
 LAEP 
 
 
larynx
 
 
distance
 
 
0.74 (0.70, 0.77)
 
 
ACE
 
 
0.03 (0.00, 0.34)
 
 
-1.94
 
 
0.8069
 
 
1.0000
 
 
0.07 (0.00, 0.65)
 
 
TRUE
 
 
 
 
 HAML 
 
 
hard palate
 
 
distance
 
 
0.56 (0.50, 0.61)
 
 
ACE
 
 
0.02 (0.00, 0.34)
 
 
-1.99
 
 
0.9059
 
 
1.0000
 
 
0.04 (0.00, 0.75)
 
 
FALSE
 
 
 
 
 HIPL 
 
 
hard palate
 
 
distance
 
 
0.60 (0.55, 0.65)
 
 
ADE
 
 
0.02 (0.00, 0.39)
 
 
-2.00
 
 
0.9641
 
 
1.0000
 
 
0.03 (0.00, 0.68)
 
 
FALSE
 
 
 
 
 L2CT 
 
 
larynx
 
 
distance
 
 
0.73 (0.69, 0.76)
 
 
ACE
 
 
0.02 (0.00, 0.35)
 
 
-1.99
 
 
0.9076
 
 
1.0000
 
 
0.03 (0.00, 0.61)
 
 
FALSE
 
 
 
 
 PPW5 
 
 
pharynx
 
 
curvature
 
 
0.41 (0.35, 0.48)
 
 
ACE
 
 
0.01 (0.00, 0.22)
 
 
-2.00
 
 
0.9666
 
 
1.0000
 
 
0.02 (0.00, 0.74)
 
 
FALSE
 
 
 
 
 PNNP 
 
 
pharynx
 
 
distance
 
 
0.74 (0.70, 0.77)
 
 
ADE
 
 
0.01 (0.00, 0.67)
 
 
-2.00
 
 
0.9749
 
 
1.0000
 
 
0.02 (0.00, 0.85)
 
 
FALSE
 
 
 
 
 DMAC 
 
 
dentition
 
 
curvature
 
 
0.21 (0.13, 0.28)
 
 
ACE
 
 
0.00 (0.00, 0.16)
 
 
-2.00
 
 
1.0000
 
 
1.0000
 
 
0.00 (0.00, 0.85)
 
 
FALSE
 
 
 
 
 PWTP 
 
 
pharynx
 
 
Procrustes dist.
 
 
0.48 (0.42, 0.54)
 
 
ACE
 
 
0.00 (0.00, 0.27)
 
 
-2.00
 
 
1.0000
 
 
1.0000
 
 
0.00 (0.00, 0.70)
 
 
FALSE
 
 
 
 
 HARF 
 
 
hard palate
 
 
angle
 
 
0.16 (0.08, 0.23)
 
 
ACE
 
 
0.00 (0.00, 0.09)
 
 
-2.00
 
 
1.0000
 
 
1.0000
 
 
0.00 (0.00, 0.48)
 
 
FALSE
 
 
 
 
 HMTC 
 
 
hard palate
 
 
distance
 
 
0.07 (-0.01, 0.15)
 
 
ACE
 
 
0.00 (0.00, 0.14)
 
 
-2.00
 
 
1.0000
 
 
1.0000
 
 
0.00 (0.00, 1.00)
 
 
FALSE
 
 
 
 
 PPW2 
 
 
pharynx
 
 
curvature
 
 
0.09 (0.01, 0.17)
 
 
ADE
 
 
0.00 (0.00, 0.14)
 
 
-2.00
 
 
1.0000
 
 
1.0000
 
 
0.00 (0.00, 1.00)
 
 
FALSE
 
 
 
 
 HACP 
 
 
hard palate
 
 
angle
 
 
0.47 (0.41, 0.53)
 
 
ACE
 
 
0.00 (0.00, 0.19)
 
 
-2.00
 
 
1.0000
 
 
1.0000
 
 
0.00 (0.00, 0.42)
 
 
FALSE
 
 
 
 
 AANP 
 
 
skull
 
 
angle
 
 
0.40 (0.33, 0.47)
 
 
ADE
 
 
0.00 (0.00, 0.24)
 
 
-2.00
 
 
1.0000
 
 
1.0000
 
 
0.00 (0.00, 0.63)
 
 
FALSE
 
 
 
 
 HCPP 
 
 
hard palate
 
 
Procrustes dist.
 
 
0.37 (0.30, 0.43)
 
 
ADE
 
 
0.00 (0.00, 0.25)
 
 
-2.00
 
 
1.0000
 
 
1.0000
 
 
0.00 (0.00, 0.74)
 
 
FALSE
 
 
 
 
 GRPD 
 
 
general
 
 
Procrustes dist.
 
 
0.30 (0.22, 0.36)
 
 
ADE
 
 
0.00 (0.00, 0.26)
 
 
-2.00
 
 
1.0000
 
 
1.0000
 
 
0.00 (0.00, 1.00)
 
 
FALSE
 
 
 
 
 PPW3 
 
 
pharynx
 
 
curvature
 
 
0.19 (0.12, 0.27)
 
 
ADE
 
 
0.00 (0.00, 0.13)
 
 
-2.00
 
 
1.0000
 
 
1.0000
 
 
0.00 (0.00, 0.75)
 
 
FALSE
 
 
 
 
 CS2H 
 
 
cervical
 
 
distance
 
 
0.59 (0.53, 0.64)
 
 
ADE
 
 
0.00 (0.00, 0.47)
 
 
-2.00
 
 
1.0000
 
 
1.0000
 
 
0.00 (0.00, 0.94)
 
 
FALSE
 
 
 
 
 HMAC 
 
 
hard palate
 
 
curvature
 
 
0.08 (0.01, 0.16)
 
 
ADE
 
 
0.00 (0.00, 0.10)
 
 
-2.00
 
 
1.0000
 
 
1.0000
 
 
0.00 (0.00, 1.00)
 
 
FALSE
 
 
 
 
 L4CT 
 
 
larynx
 
 
distance
 
 
0.46 (0.39, 0.52)
 
 
ADE
 
 
0.00 (0.00, 0.24)
 
 
-2.00
 
 
1.0000
 
 
1.0000
 
 
0.00 (0.00, 0.49)
 
 
FALSE
 
 
 
 
 OCHL 
 
 
oral
 
 
ratio
 
 
0.35 (0.28, 0.42)
 
 
ACE
 
 
0.00 (0.00, 0.25)
 
 
-2.00
 
 
1.0000
 
 
1.0000
 
 
0.00 (0.00, 0.76)
 
 
FALSE
 
 
 
 
 DAII 
 
 
dentition
 
 
angle
 
 
0.66 (0.61, 0.70)
 
 
ADE
 
 
0.00 (0.00, 0.27)
 
 
-2.00
 
 
1.0000
 
 
1.0000
 
 
0.00 (0.00, 0.37)
 
 
FALSE
 
 
 
 
 LEPL 
 
 
larynx
 
 
distance
 
 
0.40 (0.34, 0.47)
 
 
ADE
 
 
0.00 (0.00, 0.21)
 
 
-2.00
 
 
1.0000
 
 
1.0000
 
 
0.00 (0.00, 0.63)
 
 
FALSE
 
 
 
 
 MMPG 
 
 
mandible
 
 
distance
 
 
0.32 (0.24, 0.39)
 
 
ADE
 
 
0.00 (0.00, 0.30)
 
 
-2.00
 
 
1.0000
 
 
1.0000
 
 
0.00 (0.00, 1.00)
 
 
FALSE
 
 
 
 
 HP2C 
 
 
hard palate
 
 
curvature
 
 
0.26 (0.19, 0.34)
 
 
ADE
 
 
0.00 (0.00, 0.16)
 
 
-2.00
 
 
1.0000
 
 
1.0000
 
 
0.00 (0.00, 0.62)
 
 
FALSE
 
 
 
 
 HCCC 
 
 
hard palate
 
 
curvature
 
 
0.31 (0.24, 0.38)
 
 
ACE
 
 
0.00 (0.00, 0.20)
 
 
-2.00
 
 
1.0000
 
 
1.0000
 
 
0.00 (0.00, 0.79)
 
 
FALSE
 
 
 
 
 HDMP 
 
 
hard palate
 
 
angle
 
 
0.56 (0.50, 0.61)
 
 
ADE
 
 
0.00 (0.00, 0.20)
 
 
-2.00
 
 
1.0000
 
 
1.0000
 
 
0.00 (0.00, 0.43)
 
 
FALSE
 
 
 
 
 LHEP 
 
 
larynx
 
 
distance
 
 
0.47 (0.41, 0.53)
 
 
ADE
 
 
0.00 (0.00, 0.20)
 
 
-2.00
 
 
1.0000
 
 
1.0000
 
 
0.00 (0.00, 0.50)
 
 
FALSE
 
 
 
 
 L3CT 
 
 
larynx
 
 
distance
 
 
0.69 (0.64, 0.73)
 
 
ACE
 
 
0.00 (0.00, 0.32)
 
 
-2.00
 
 
1.0000
 
 
1.0000
 
 
0.00 (0.00, 0.52)
 
 
FALSE
 
 
 
 
 HHPL 
 
 
hard palate
 
 
distance
 
 
0.50 (0.44, 0.56)
 
 
ACE
 
 
0.00 (0.00, 0.27)
 
 
-2.00
 
 
1.0000
 
 
1.0000
 
 
0.00 (0.00, 0.58)
 
 
FALSE
 
 
 
 
 HMDC 
 
 
hard palate
 
 
curvature
 
 
0.21 (0.13, 0.28)
 
 
ACE
 
 
0.00 (0.00, 0.15)
 
 
-2.00
 
 
1.0000
 
 
1.0000
 
 
0.00 (0.00, 0.69)
 
 
FALSE
 
 
 
 
 DAIP 
 
 
dentition
 
 
angle
 
 
0.62 (0.57, 0.67)
 
 
ADE
 
 
0.00 (0.00, 0.43)
 
 
-2.00
 
 
1.0000
 
 
1.0000
 
 
0.00 (0.00, 0.73)
 
 
FALSE
 
 
 
 
 HCMP 
 
 
hard palate
 
 
Procrustes dist.
 
 
0.23 (0.15, 0.30)
 
 
ADE
 
 
0.00 (0.00, 0.19)
 
 
-2.00
 
 
1.0000
 
 
1.0000
 
 
0.00 (0.00, 0.76)
 
 
FALSE
 
 
 
 
 HPTH 
 
 
hard palate
 
 
distance
 
 
0.52 (0.46, 0.57)
 
 
ADE
 
 
0.00 (0.00, 0.44)
 
 
-2.00
 
 
1.0000
 
 
1.0000
 
 
0.00 (0.00, 0.85)
 
 
FALSE
 
 
 
 
 SVTv 
 
 
general
 
 
distance
 
 
0.73 (0.70, 0.77)
 
 
ACE
 
 
0.00 (0.00, 0.13)
 
 
-2.00
 
 
1.0000
 
 
1.0000
 
 
0.00 (0.00, 0.36)
 
 
FALSE
 
 
 
 
 HMSP 
 
 
hard palate
 
 
Procrustes dist.
 
 
0.11 (0.03, 0.19)
 
 
ACE
 
 
0.00 (0.00, 0.15)
 
 
-2.00
 
 
1.0000
 
 
1.0000
 
 
0.00 (0.00, 1.00)
 
 
FALSE
 
 
 
 
 DDAP 
 
 
dentition
 
 
Procrustes dist.
 
 
0.24 (0.17, 0.32)
 
 
ADE
 
 
0.00 (0.00, 0.23)
 
 
-2.00
 
 
1.0000
 
 
1.0000
 
 
0.00 (0.00, 1.00)
 
 
FALSE
 
 
 
 
 HACL 
 
 
hard palate
 
 
distance
 
 
0.26 (0.19, 0.33)
 
 
ACE
 
 
0.00 (0.00, 0.16)
 
 
-2.00
 
 
1.0000
 
 
1.0000
 
 
0.00 (0.00, 0.67)
 
 
FALSE
 
 
 
 
 HCCP 
 
 
hard palate
 
 
Procrustes dist.
 
 
0.11 (0.03, 0.19)
 
 
ACE
 
 
0.00 (0.00, 0.13)
 
 
-2.00
 
 
1.0000
 
 
1.0000
 
 
0.00 (0.00, 1.00)
 
 
FALSE
 
 
 
 
 DAIM 
 
 
dentition
 
 
angle
 
 
0.63 (0.58, 0.67)
 
 
ADE
 
 
0.00 (0.00, 0.42)
 
 
-2.00
 
 
1.0000
 
 
1.0000
 
 
0.00 (0.00, 0.72)
 
 
FALSE
 
 
 
 
 CS4H 
 
 
cervical
 
 
distance
 
 
0.59 (0.53, 0.64)
 
 
ACE
 
 
0.00 (0.00, 0.14)
 
 
-2.00
 
 
1.0000
 
 
1.0000
 
 
0.00 (0.00, 0.32)
 
 
FALSE
 
 
 
 
 
 
  C  and  c  2  
 Relationship between the significance of  C  judged by AIC and
LRT is almost perfect: 
 
 
 
 
 
 
 
 
   
 FALSE 
 TRUE 
 
 
 
 
  FALSE  
 83 
 1 
 
 
  TRUE  
 0 
 9 
 
 
 
 but that between AIC( (or, equivalently, LRT) and the inclusion of 0
within the 95%CI of  c  2  is not, with the latter being
more liberal (i.e., finding more measures with a significant
 c  2 ): 
 
 
 
 
 
 
 
 
   
 FALSE 
 TRUE 
 
 
 
 
  FALSE  
 16 
 68 
 
 
  TRUE  
 0 
 9 
 
 
 
 
 
Measures with a significant  C  component, ordered by the
decreasing strength of evidence for  C , inter-rater agreement
and estimate of  c  2 . In  dark blue  measures
with a significant  C  component after Holm’s correction (0),
while in  blue  those only nominally significant (10); in
 bold  measures with  ICC(C,1)  > 0.75 (29), in
 bold italic  mesures with the lower 95%CI of  ICC(C,1) 
> 0.75 (24); in gray those with  c  2  < 0.20
(33). Column  c  2  > 0 is TRUE if and only if 0 is
not included in the 95% CI of  c  2  (this is a much
less strict test than ΔAIC and LTR).
 
 
 
 
measure
 
 
domain
 
 
type
 
 
 ICC ( C ,1)
 
 
genetic model
 
 
 C 
 
 
ΔAIC
 
 
 p 
 
 
 p (Holm)
 
 
 c  2 
 
 
 c  2 >0
 
 
 
 
 
 
 GAPD 
 
 
general
 
 
Procrustes dist.
 
 
0.80 (0.77, 0.82)
 
 
ACE
 
 
0.47 (0.23, 0.58)
 
 
6.26
 
 
0.0041
 
 
0.3769
 
 
0.70 (0.35, 0.77)
 
 
TRUE
 
 
 
 
 SVTh 
 
 
general
 
 
distance
 
 
0.90 (0.88, 0.91)
 
 
ACE
 
 
0.45 (0.25, 0.66)
 
 
5.94
 
 
0.0048
 
 
0.4460
 
 
0.59 (0.22, 0.79)
 
 
TRUE
 
 
 
 
 SVTh* 
 
 
general
 
 
distance
 
 
0.93 (0.91, 0.94)
 
 
ACE
 
 
0.42 (0.15, 0.64)
 
 
5.75
 
 
0.0054
 
 
0.4893
 
 
0.55 (0.20, 0.77)
 
 
TRUE
 
 
 
 
 SVTv 
 
 
general
 
 
distance
 
 
0.73 (0.70, 0.77)
 
 
ACE
 
 
0.25 (0.19, 0.32)
 
 
5.38
 
 
0.0066
 
 
0.5941
 
 
0.62 (0.25, 0.72)
 
 
TRUE
 
 
 
 
 CS4H 
 
 
cervical
 
 
distance
 
 
0.59 (0.53, 0.64)
 
 
ACE
 
 
0.21 (0.15, 0.29)
 
 
5.03
 
 
0.0080
 
 
0.7128
 
 
0.56 (0.40, 0.70)
 
 
TRUE
 
 
 
 
 HBPL 
 
 
hard palate
 
 
distance
 
 
0.91 (0.89, 0.92)
 
 
ACE
 
 
0.32 (0.00, 0.53)
 
 
2.86
 
 
0.0274
 
 
1.0000
 
 
0.45 (0.08, 0.71)
 
 
TRUE
 
 
 
 
 HNSL 
 
 
hard palate
 
 
distance
 
 
0.77 (0.74, 0.80)
 
 
ACE
 
 
0.30 (0.00, 0.52)
 
 
2.09
 
 
0.0431
 
 
1.0000
 
 
0.49 (0.02, 0.80)
 
 
TRUE
 
 
 
 
 L2CT 
 
 
larynx
 
 
distance
 
 
0.73 (0.69, 0.76)
 
 
ACE
 
 
0.34 (0.07, 0.45)
 
 
2.32
 
 
0.0377
 
 
1.0000
 
 
0.60 (0.04, 0.72)
 
 
TRUE
 
 
 
 
 L3CT 
 
 
larynx
 
 
distance
 
 
0.69 (0.64, 0.73)
 
 
ACE
 
 
0.34 (0.04, 0.44)
 
 
2.64
 
 
0.0312
 
 
1.0000
 
 
0.58 (0.06, 0.68)
 
 
TRUE
 
 
 
 
 SSEN 
 
 
skull
 
 
distance
 
 
0.90 (0.89, 0.92)
 
 
ACE
 
 
0.27 (0.00, 0.46)
 
 
1.86
 
 
0.0494
 
 
1.0000
 
 
0.40 (0.00, 0.67)
 
 
TRUE
 
 
 
 
 LACT 
 
 
larynx
 
 
distance
 
 
0.81 (0.78, 0.83)
 
 
ACE
 
 
0.25 (0.00, 0.41)
 
 
1.11
 
 
0.0778
 
 
1.0000
 
 
0.47 (0.00, 0.71)
 
 
TRUE
 
 
 
 
 SVTv* 
 
 
general
 
 
distance
 
 
0.80 (0.77, 0.83)
 
 
ACE
 
 
0.19 (0.00, 0.35)
 
 
0.45
 
 
0.1179
 
 
1.0000
 
 
0.43 (0.00, 0.73)
 
 
TRUE
 
 
 
 
 SHBN 
 
 
skull
 
 
distance
 
 
0.96 (0.95, 0.96)
 
 
ACE
 
 
0.20 (0.00, 0.37)
 
 
1.32
 
 
0.0685
 
 
1.0000
 
 
0.34 (0.00, 0.58)
 
 
TRUE
 
 
 
 
 SNNP 
 
 
skull
 
 
distance
 
 
0.82 (0.80, 0.85)
 
 
ACE
 
 
0.16 (0.00, 0.35)
 
 
-0.49
 
 
0.2193
 
 
1.0000
 
 
0.29 (0.00, 0.63)
 
 
TRUE
 
 
 
 
 SBAN 
 
 
skull
 
 
distance
 
 
0.89 (0.88, 0.91)
 
 
ACE
 
 
0.13 (0.00, 0.31)
 
 
-0.71
 
 
0.2559
 
 
1.0000
 
 
0.22 (0.00, 0.52)
 
 
TRUE
 
 
 
 
 HBPG 
 
 
hyoid
 
 
distance
 
 
0.81 (0.78, 0.83)
 
 
ACE
 
 
0.16 (0.00, 0.48)
 
 
-1.43
 
 
0.4501
 
 
1.0000
 
 
0.22 (0.00, 0.62)
 
 
TRUE
 
 
 
 
 SHSW 
 
 
skull
 
 
distance
 
 
0.94 (0.93, 0.95)
 
 
ACE
 
 
0.08 (0.00, 0.23)
 
 
-1.28
 
 
0.3961
 
 
1.0000
 
 
0.19 (0.00, 0.53)
 
 
TRUE
 
 
 
 
 MIGD 
 
 
mandible
 
 
distance
 
 
0.95 (0.94, 0.95)
 
 
ACE
 
 
0.09 (0.00, 0.23)
 
 
-1.05
 
 
0.3302
 
 
1.0000
 
 
0.19 (0.00, 0.49)
 
 
TRUE
 
 
 
 
 SBNA 
 
 
skull
 
 
distance
 
 
0.90 (0.88, 0.91)
 
 
ACE
 
 
0.11 (0.00, 0.35)
 
 
-1.41
 
 
0.4429
 
 
1.0000
 
 
0.17 (0.00, 0.51)
 
 
TRUE
 
 
 
 
 ASCG* 
 
 
skull
 
 
angle
 
 
0.87 (0.85, 0.89)
 
 
ACE
 
 
0.12 (0.00, 0.43)
 
 
-1.62
 
 
0.5360
 
 
1.0000
 
 
0.14 (0.00, 0.48)
 
 
TRUE
 
 
 
 
 MCGM 
 
 
mandible
 
 
angle
 
 
0.89 (0.87, 0.90)
 
 
ACE
 
 
0.12 (0.00, 0.43)
 
 
-1.62
 
 
0.5360
 
 
1.0000
 
 
0.14 (0.00, 0.48)
 
 
TRUE
 
 
 
 
 HBNP 
 
 
hyoid
 
 
distance
 
 
0.84 (0.81, 0.86)
 
 
ACE
 
 
0.06 (0.00, 0.25)
 
 
-1.73
 
 
0.6006
 
 
1.0000
 
 
0.13 (0.00, 0.56)
 
 
TRUE
 
 
 
 
 HBC4 
 
 
hyoid
 
 
distance
 
 
0.79 (0.76, 0.82)
 
 
ACE
 
 
0.09 (0.00, 0.45)
 
 
-1.80
 
 
0.6586
 
 
1.0000
 
 
0.12 (0.00, 0.57)
 
 
FALSE
 
 
 
 
 MCGP 
 
 
mandible
 
 
angle
 
 
0.87 (0.84, 0.88)
 
 
ACE
 
 
0.06 (0.00, 0.37)
 
 
-1.90
 
 
0.7469
 
 
1.0000
 
 
0.07 (0.00, 0.44)
 
 
TRUE
 
 
 
 
 SNAM 
 
 
skull
 
 
distance
 
 
0.90 (0.89, 0.92)
 
 
ACE
 
 
0.03 (0.00, 0.27)
 
 
-1.94
 
 
0.8118
 
 
1.0000
 
 
0.06 (0.00, 0.43)
 
 
TRUE
 
 
 
 
 SNAP 
 
 
skull
 
 
distance
 
 
0.88 (0.86, 0.90)
 
 
ACE
 
 
0.03 (0.00, 0.26)
 
 
-1.95
 
 
0.8194
 
 
1.0000
 
 
0.05 (0.00, 0.43)
 
 
TRUE
 
 
 
 
 APNS 
 
 
skull
 
 
angle
 
 
0.83 (0.80, 0.85)
 
 
ACE
 
 
0.01 (0.00, 0.38)
 
 
-2.00
 
 
0.9520
 
 
1.0000
 
 
0.02 (0.00, 0.48)
 
 
FALSE
 
 
 
 
 SSEG 
 
 
skull
 
 
distance
 
 
0.90 (0.88, 0.91)
 
 
ACE
 
 
0.01 (0.00, 0.31)
 
 
-2.00
 
 
0.9694
 
 
1.0000
 
 
0.01 (0.00, 0.39)
 
 
FALSE
 
 
 
 
 MICD 
 
 
mandible
 
 
distance
 
 
0.98 (0.98, 0.99)
 
 
ACE
 
 
0.00 (0.00, 0.20)
 
 
-2.00
 
 
0.9775
 
 
1.0000
 
 
0.01 (0.00, 0.36)
 
 
FALSE
 
 
 
 
 DIMD 
 
 
dentition
 
 
distance
 
 
0.77 (0.73, 0.80)
 
 
ACE
 
 
0.25 (0.00, 0.49)
 
 
0.26
 
 
0.1330
 
 
1.0000
 
 
0.40 (0.00, 0.72)
 
 
TRUE
 
 
 
 
 HBC2 
 
 
hyoid
 
 
distance
 
 
0.78 (0.74, 0.81)
 
 
ACE
 
 
0.12 (0.00, 0.30)
 
 
-1.03
 
 
0.3247
 
 
1.0000
 
 
0.29 (0.00, 0.67)
 
 
TRUE
 
 
 
 
 LAEA 
 
 
larynx
 
 
distance
 
 
0.77 (0.73, 0.80)
 
 
ACE
 
 
0.13 (0.00, 0.37)
 
 
-1.56
 
 
0.5088
 
 
1.0000
 
 
0.23 (0.00, 0.58)
 
 
TRUE
 
 
 
 
 L4EA 
 
 
larynx
 
 
distance
 
 
0.76 (0.72, 0.79)
 
 
ACE
 
 
0.12 (0.00, 0.46)
 
 
-1.64
 
 
0.5502
 
 
1.0000
 
 
0.17 (0.00, 0.61)
 
 
FALSE
 
 
 
 
 HMTC 
 
 
hard palate
 
 
distance
 
 
0.07 (-0.01, 0.15)
 
 
ACE
 
 
0.08 (0.00, 0.14)
 
 
-1.57
 
 
0.5133
 
 
1.0000
 
 
0.89 (0.00, 1.00)
 
 
TRUE
 
 
 
 
 HCCP 
 
 
hard palate
 
 
Procrustes dist.
 
 
0.11 (0.03, 0.19)
 
 
ACE
 
 
0.09 (0.00, 0.15)
 
 
-0.33
 
 
0.1967
 
 
1.0000
 
 
0.88 (0.00, 1.00)
 
 
TRUE
 
 
 
 
 HMSP 
 
 
hard palate
 
 
Procrustes dist.
 
 
0.11 (0.03, 0.19)
 
 
ACE
 
 
0.08 (0.00, 0.15)
 
 
-0.92
 
 
0.2976
 
 
1.0000
 
 
0.83 (0.00, 1.00)
 
 
TRUE
 
 
 
 
 HAML 
 
 
hard palate
 
 
distance
 
 
0.56 (0.50, 0.61)
 
 
ACE
 
 
0.28 (0.00, 0.38)
 
 
0.96
 
 
0.0852
 
 
1.0000
 
 
0.62 (0.00, 0.78)
 
 
TRUE
 
 
 
 
 CS3H 
 
 
cervical
 
 
distance
 
 
0.60 (0.54, 0.64)
 
 
ACE
 
 
0.22 (0.00, 0.36)
 
 
0.46
 
 
0.1171
 
 
1.0000
 
 
0.60 (0.00, 0.90)
 
 
TRUE
 
 
 
 
 HCCC 
 
 
hard palate
 
 
curvature
 
 
0.31 (0.24, 0.38)
 
 
ACE
 
 
0.15 (0.00, 0.22)
 
 
-0.47
 
 
0.2162
 
 
1.0000
 
 
0.57 (0.00, 0.85)
 
 
TRUE
 
 
 
 
 SNNA 
 
 
skull
 
 
distance
 
 
0.66 (0.61, 0.70)
 
 
ACE
 
 
0.24 (0.00, 0.43)
 
 
0.87
 
 
0.0904
 
 
1.0000
 
 
0.56 (0.00, 0.91)
 
 
TRUE
 
 
 
 
 PWTP 
 
 
pharynx
 
 
Procrustes dist.
 
 
0.48 (0.42, 0.54)
 
 
ACE
 
 
0.20 (0.00, 0.28)
 
 
-0.61
 
 
0.2376
 
 
1.0000
 
 
0.54 (0.00, 0.71)
 
 
TRUE
 
 
 
 
 OCHL 
 
 
oral
 
 
ratio
 
 
0.35 (0.28, 0.42)
 
 
ACE
 
 
0.17 (0.00, 0.25)
 
 
-0.69
 
 
0.2517
 
 
1.0000
 
 
0.54 (0.00, 0.77)
 
 
TRUE
 
 
 
 
 OMLW 
 
 
oral
 
 
ratio
 
 
0.70 (0.65, 0.73)
 
 
ACE
 
 
0.27 (0.00, 0.39)
 
 
1.18
 
 
0.0744
 
 
1.0000
 
 
0.52 (0.00, 0.71)
 
 
TRUE
 
 
 
 
 HHPL 
 
 
hard palate
 
 
distance
 
 
0.50 (0.44, 0.56)
 
 
ACE
 
 
0.24 (0.00, 0.33)
 
 
1.29
 
 
0.0698
 
 
1.0000
 
 
0.51 (0.00, 0.66)
 
 
TRUE
 
 
 
 
 PPW5 
 
 
pharynx
 
 
curvature
 
 
0.41 (0.35, 0.48)
 
 
ACE
 
 
0.14 (0.00, 0.22)
 
 
-0.86
 
 
0.2856
 
 
1.0000
 
 
0.51 (0.00, 0.73)
 
 
TRUE
 
 
 
 
 LAEP 
 
 
larynx
 
 
distance
 
 
0.74 (0.70, 0.77)
 
 
ACE
 
 
0.25 (0.00, 0.36)
 
 
0.61
 
 
0.1063
 
 
1.0000
 
 
0.50 (0.00, 0.67)
 
 
TRUE
 
 
 
 
 HMDC 
 
 
hard palate
 
 
curvature
 
 
0.21 (0.13, 0.28)
 
 
ACE
 
 
0.11 (0.00, 0.18)
 
 
0.10
 
 
0.1472
 
 
1.0000
 
 
0.49 (0.00, 0.84)
 
 
TRUE
 
 
 
 
 HNAP 
 
 
hard palate
 
 
distance
 
 
0.37 (0.30, 0.44)
 
 
ACE
 
 
0.16 (0.00, 0.30)
 
 
-1.17
 
 
0.3635
 
 
1.0000
 
 
0.48 (0.00, 0.82)
 
 
TRUE
 
 
 
 
 HIML* 
 
 
hard palate
 
 
distance
 
 
0.65 (0.60, 0.69)
 
 
ACE
 
 
0.25 (0.00, 0.45)
 
 
0.36
 
 
0.1247
 
 
1.0000
 
 
0.47 (0.00, 0.77)
 
 
TRUE
 
 
 
 
 ALNS 
 
 
skull
 
 
angle
 
 
0.60 (0.55, 0.65)
 
 
ACE
 
 
0.27 (0.00, 0.50)
 
 
-0.50
 
 
0.2214
 
 
1.0000
 
 
0.46 (0.00, 0.78)
 
 
TRUE
 
 
 
 
 HICL* 
 
 
hard palate
 
 
distance
 
 
0.39 (0.32, 0.45)
 
 
ACE
 
 
0.14 (0.00, 0.25)
 
 
-1.41
 
 
0.4431
 
 
1.0000
 
 
0.44 (0.00, 0.78)
 
 
TRUE
 
 
 
 
 L5CT 
 
 
larynx
 
 
distance
 
 
0.71 (0.67, 0.75)
 
 
ACE
 
 
0.27 (0.00, 0.44)
 
 
0.58
 
 
0.1080
 
 
1.0000
 
 
0.44 (0.00, 0.65)
 
 
TRUE
 
 
 
 
 DMAC 
 
 
dentition
 
 
curvature
 
 
0.21 (0.13, 0.28)
 
 
ACE
 
 
0.08 (0.00, 0.15)
 
 
-1.71
 
 
0.5871
 
 
1.0000
 
 
0.43 (0.00, 0.82)
 
 
TRUE
 
 
 
 
 PPW7 
 
 
pharynx
 
 
curvature
 
 
0.71 (0.67, 0.74)
 
 
ACE
 
 
0.22 (0.00, 0.44)
 
 
-0.12
 
 
0.1702
 
 
1.0000
 
 
0.43 (0.00, 0.77)
 
 
TRUE
 
 
 
 
 HIPL* 
 
 
hard palate
 
 
distance
 
 
0.63 (0.58, 0.67)
 
 
ACE
 
 
0.23 (0.00, 0.41)
 
 
-0.63
 
 
0.2413
 
 
1.0000
 
 
0.41 (0.00, 0.67)
 
 
TRUE
 
 
 
 
 CS7H 
 
 
cervical
 
 
distance
 
 
0.69 (0.64, 0.72)
 
 
ACE
 
 
0.24 (0.00, 0.48)
 
 
-0.55
 
 
0.2291
 
 
1.0000
 
 
0.40 (0.00, 0.72)
 
 
TRUE
 
 
 
 
 PPW4 
 
 
pharynx
 
 
curvature
 
 
0.31 (0.24, 0.38)
 
 
ACE
 
 
0.09 (0.00, 0.19)
 
 
-1.58
 
 
0.5182
 
 
1.0000
 
 
0.36 (0.00, 0.69)
 
 
TRUE
 
 
 
 
 HACL 
 
 
hard palate
 
 
distance
 
 
0.26 (0.19, 0.33)
 
 
ACE
 
 
0.08 (0.00, 0.15)
 
 
-1.71
 
 
0.5929
 
 
1.0000
 
 
0.35 (0.00, 0.55)
 
 
TRUE
 
 
 
 
 DSIL 
 
 
dentition
 
 
distance
 
 
0.45 (0.39, 0.51)
 
 
ACE
 
 
0.13 (0.00, 0.32)
 
 
-1.35
 
 
0.4190
 
 
1.0000
 
 
0.35 (0.00, 0.78)
 
 
FALSE
 
 
 
 
 HCTH 
 
 
hard palate
 
 
distance
 
 
0.44 (0.37, 0.50)
 
 
ACE
 
 
0.12 (0.00, 0.25)
 
 
-1.56
 
 
0.5066
 
 
1.0000
 
 
0.34 (0.00, 0.70)
 
 
TRUE
 
 
 
 
 HAPL 
 
 
hard palate
 
 
distance
 
 
0.52 (0.46, 0.57)
 
 
ACE
 
 
0.16 (0.00, 0.31)
 
 
-1.41
 
 
0.4416
 
 
1.0000
 
 
0.34 (0.00, 0.60)
 
 
TRUE
 
 
 
 
 OCHW 
 
 
oral
 
 
ratio
 
 
0.41 (0.34, 0.47)
 
 
ACE
 
 
0.13 (0.00, 0.27)
 
 
-1.34
 
 
0.4172
 
 
1.0000
 
 
0.33 (0.00, 0.68)
 
 
TRUE
 
 
 
 
 L2EP 
 
 
larynx
 
 
distance
 
 
0.67 (0.62, 0.71)
 
 
ACE
 
 
0.17 (0.00, 0.39)
 
 
-1.04
 
 
0.3260
 
 
1.0000
 
 
0.33 (0.00, 0.68)
 
 
TRUE
 
 
 
 
 SNOL 
 
 
skull
 
 
distance
 
 
0.54 (0.48, 0.59)
 
 
ACE
 
 
0.11 (0.00, 0.34)
 
 
-1.38
 
 
0.4296
 
 
1.0000
 
 
0.31 (0.00, 0.90)
 
 
TRUE
 
 
 
 
 L3EP 
 
 
larynx
 
 
distance
 
 
0.57 (0.52, 0.62)
 
 
ACE
 
 
0.15 (0.00, 0.37)
 
 
-1.26
 
 
0.3905
 
 
1.0000
 
 
0.31 (0.00, 0.68)
 
 
TRUE
 
 
 
 
 HICH 
 
 
hard palate
 
 
distance
 
 
0.39 (0.32, 0.46)
 
 
ACE
 
 
0.11 (0.00, 0.25)
 
 
-1.50
 
 
0.4790
 
 
1.0000
 
 
0.30 (0.00, 0.63)
 
 
TRUE
 
 
 
 
 OPLW 
 
 
oral
 
 
ratio
 
 
0.50 (0.44, 0.56)
 
 
ACE
 
 
0.13 (0.00, 0.28)
 
 
-1.49
 
 
0.4765
 
 
1.0000
 
 
0.29 (0.00, 0.58)
 
 
TRUE
 
 
 
 
 PPWC 
 
 
pharynx
 
 
curvature
 
 
0.27 (0.19, 0.34)
 
 
ACE
 
 
0.06 (0.00, 0.18)
 
 
-1.82
 
 
0.6694
 
 
1.0000
 
 
0.28 (0.00, 0.82)
 
 
FALSE
 
 
 
 
 HIPH 
 
 
hard palate
 
 
distance
 
 
0.43 (0.36, 0.49)
 
 
ACE
 
 
0.11 (0.00, 0.29)
 
 
-1.67
 
 
0.5653
 
 
1.0000
 
 
0.27 (0.00, 0.68)
 
 
FALSE
 
 
 
 
 HIML 
 
 
hard palate
 
 
distance
 
 
0.63 (0.58, 0.67)
 
 
ACE
 
 
0.13 (0.00, 0.38)
 
 
-1.33
 
 
0.4128
 
 
1.0000
 
 
0.27 (0.00, 0.73)
 
 
TRUE
 
 
 
 
 HACP 
 
 
hard palate
 
 
angle
 
 
0.47 (0.41, 0.53)
 
 
ACE
 
 
0.11 (0.00, 0.19)
 
 
-1.50
 
 
0.4791
 
 
1.0000
 
 
0.25 (0.00, 0.43)
 
 
TRUE
 
 
 
 
 OCMR 
 
 
oral
 
 
ratio
 
 
0.47 (0.40, 0.53)
 
 
ACE
 
 
0.11 (0.00, 0.31)
 
 
-1.64
 
 
0.5504
 
 
1.0000
 
 
0.25 (0.00, 0.65)
 
 
TRUE
 
 
 
 
 OPHL 
 
 
oral
 
 
ratio
 
 
0.38 (0.31, 0.44)
 
 
ACE
 
 
0.09 (0.00, 0.25)
 
 
-1.81
 
 
0.6632
 
 
1.0000
 
 
0.25 (0.00, 0.64)
 
 
FALSE
 
 
 
 
 SPUN 
 
 
soft palate
 
 
distance
 
 
0.63 (0.58, 0.67)
 
 
ACE
 
 
0.12 (0.00, 0.42)
 
 
-1.59
 
 
0.5205
 
 
1.0000
 
 
0.21 (0.00, 0.69)
 
 
TRUE
 
 
 
 
 L5EA 
 
 
larynx
 
 
distance
 
 
0.74 (0.70, 0.77)
 
 
ACE
 
 
0.12 (0.00, 0.45)
 
 
-1.65
 
 
0.5514
 
 
1.0000
 
 
0.18 (0.00, 0.63)
 
 
FALSE
 
 
 
 
 MCGL 
 
 
mandible
 
 
distance
 
 
0.68 (0.64, 0.72)
 
 
ACE
 
 
0.08 (0.00, 0.30)
 
 
-1.60
 
 
0.5260
 
 
1.0000
 
 
0.18 (0.00, 0.65)
 
 
FALSE
 
 
 
 
 AASN 
 
 
skull
 
 
angle
 
 
0.63 (0.58, 0.67)
 
 
ACE
 
 
0.10 (0.00, 0.40)
 
 
-1.65
 
 
0.5527
 
 
1.0000
 
 
0.18 (0.00, 0.65)
 
 
TRUE
 
 
 
 
 PPW6 
 
 
pharynx
 
 
curvature
 
 
0.43 (0.37, 0.50)
 
 
ACE
 
 
0.05 (0.00, 0.26)
 
 
-1.86
 
 
0.7101
 
 
1.0000
 
 
0.18 (0.00, 0.82)
 
 
TRUE
 
 
 
 
 OMHW 
 
 
oral
 
 
ratio
 
 
0.48 (0.42, 0.54)
 
 
ACE
 
 
0.08 (0.00, 0.30)
 
 
-1.86
 
 
0.7044
 
 
1.0000
 
 
0.17 (0.00, 0.59)
 
 
TRUE
 
 
 
 
 DAIS 
 
 
dentition
 
 
angle
 
 
0.65 (0.60, 0.69)
 
 
ACE
 
 
0.11 (0.00, 0.35)
 
 
-1.85
 
 
0.6993
 
 
1.0000
 
 
0.17 (0.00, 0.52)
 
 
FALSE
 
 
 
 
 LHEA 
 
 
larynx
 
 
distance
 
 
0.61 (0.56, 0.66)
 
 
ACE
 
 
0.05 (0.00, 0.21)
 
 
-1.92
 
 
0.7747
 
 
1.0000
 
 
0.14 (0.00, 0.50)
 
 
TRUE
 
 
 
 
 HBC3 
 
 
hyoid
 
 
distance
 
 
0.71 (0.67, 0.74)
 
 
ACE
 
 
0.06 (0.00, 0.33)
 
 
-1.85
 
 
0.6958
 
 
1.0000
 
 
0.12 (0.00, 0.64)
 
 
TRUE
 
 
 
 
 MCGR 
 
 
mandible
 
 
distance
 
 
0.68 (0.64, 0.72)
 
 
ACE
 
 
0.05 (0.00, 0.27)
 
 
-1.81
 
 
0.6670
 
 
1.0000
 
 
0.12 (0.00, 0.61)
 
 
TRUE
 
 
 
 
 ASNP 
 
 
skull
 
 
angle
 
 
0.72 (0.68, 0.76)
 
 
ACE
 
 
0.08 (0.00, 0.41)
 
 
-1.85
 
 
0.6944
 
 
1.0000
 
 
0.12 (0.00, 0.58)
 
 
TRUE
 
 
 
 
 CS2A 
 
 
cervical
 
 
distance
 
 
0.60 (0.55, 0.65)
 
 
ACE
 
 
0.05 (0.00, 0.28)
 
 
-1.90
 
 
0.7503
 
 
1.0000
 
 
0.12 (0.00, 0.66)
 
 
TRUE
 
 
 
 
 OMHL 
 
 
oral
 
 
ratio
 
 
0.44 (0.37, 0.50)
 
 
ACE
 
 
0.04 (0.00, 0.23)
 
 
-1.94
 
 
0.8051
 
 
1.0000
 
 
0.11 (0.00, 0.55)
 
 
TRUE
 
 
 
 
 HARF 
 
 
hard palate
 
 
angle
 
 
0.16 (0.08, 0.23)
 
 
ACE
 
 
0.02 (0.00, 0.09)
 
 
-1.84
 
 
0.6936
 
 
1.0000
 
 
0.11 (0.00, 0.50)
 
 
FALSE
 
 
 
 
 OPHW 
 
 
oral
 
 
ratio
 
 
0.50 (0.43, 0.55)
 
 
ACE
 
 
0.04 (0.00, 0.29)
 
 
-1.96
 
 
0.8332
 
 
1.0000
 
 
0.09 (0.00, 0.60)
 
 
TRUE
 
 
 
 
 LHCT 
 
 
larynx
 
 
distance
 
 
0.66 (0.62, 0.70)
 
 
ACE
 
 
0.04 (0.00, 0.32)
 
 
-1.96
 
 
0.8337
 
 
1.0000
 
 
0.08 (0.00, 0.65)
 
 
FALSE
 
 
 
 
 HARC 
 
 
hard palate
 
 
angle
 
 
0.36 (0.29, 0.42)
 
 
ACE
 
 
0.03 (0.00, 0.18)
 
 
-1.97
 
 
0.8679
 
 
1.0000
 
 
0.08 (0.00, 0.52)
 
 
TRUE
 
 
 
 
 CS5H 
 
 
cervical
 
 
distance
 
 
0.43 (0.37, 0.50)
 
 
ACE
 
 
0.02 (0.00, 0.27)
 
 
-1.99
 
 
0.9073
 
 
1.0000
 
 
0.06 (0.00, 0.70)
 
 
TRUE
 
 
 
 
 MCGD 
 
 
mandible
 
 
distance
 
 
0.71 (0.67, 0.75)
 
 
ACE
 
 
0.01 (0.00, 0.20)
 
 
-1.99
 
 
0.9427
 
 
1.0000
 
 
0.02 (0.00, 0.47)
 
 
FALSE
 
 
 
 
 OCLW 
 
 
oral
 
 
ratio
 
 
0.25 (0.17, 0.32)
 
 
ACE
 
 
0.00 (0.00, 0.13)
 
 
-2.00
 
 
0.9865
 
 
1.0000
 
 
0.01 (0.00, 0.01)
 
 
FALSE
 
 
 
 
 
 
  D  and  d  2  
 Relationship between the significance of  D  judged by AIC and
LRT is perfect: 
 
 
 
 
 
 
 
 
   
 FALSE 
 TRUE 
 
 
 
 
  FALSE  
 51 
 0 
 
 
  TRUE  
 0 
 2 
 
 
 
 but that between AIC( (or, equivalently, LRT) and the inclusion of 0
within the 95%CI of  d  2  is not, with the latter being
more liberal (i.e., finding more measures with a significant
 d  2 ): 
 
 
 
 
 
 
 
 
   
 FALSE 
 TRUE 
 
 
 
 
  FALSE  
 12 
 39 
 
 
  TRUE  
 0 
 2 
 
 
 
 
 
Measures with a significant  D  component, ordered by the
decreasing strength of evidence for  D , inter-rater agreement
and estimate of  d  2 . In  dark blue  measures
with a significant  D  component after Holm’s correction (0),
while in  blue  those only nominally significant (2); in
 bold  measures with  ICC(C,1)  > 0.75 (15), in
 bold italic  mesures with the lower 95%CI of  ICC(C,1) 
> 0.75 (13); in gray those with  d  2  < 0.20
(14). Column  c  2  > 0 is TRUE if and only if 0 is
not included in the 95% CI of  d  2  (this is a much
less strict test than ΔAIC and LTR).
 
 
 
 
measure
 
 
domain
 
 
type
 
 
 ICC ( C ,1)
 
 
genetic model
 
 
 C 
 
 
ΔAIC
 
 
 p 
 
 
 p (Holm)
 
 
 c  2 
 
 
 c  2 >0
 
 
 
 
 
 
 DAII 
 
 
dentition
 
 
angle
 
 
0.66 (0.61, 0.70)
 
 
ADE
 
 
0.40 (0.13, 0.52)
 
 
4.01
 
 
0.0142
 
 
0.7524
 
 
0.63 (0.19, 0.74)
 
 
TRUE
 
 
 
 
 MGMD 
 
 
mandible
 
 
distance
 
 
0.80 (0.77, 0.83)
 
 
ADE
 
 
0.53 (0.13, 0.63)
 
 
3.60
 
 
0.0180
 
 
0.9362
 
 
0.85 (0.18, 0.90)
 
 
TRUE
 
 
 
 
 MGPD 
 
 
mandible
 
 
distance
 
 
0.83 (0.80, 0.85)
 
 
ADE
 
 
0.50 (0.00, 0.64)
 
 
0.16
 
 
0.1414
 
 
1.0000
 
 
0.78 (0.00, 0.88)
 
 
TRUE
 
 
 
 
 MCMD 
 
 
mandible
 
 
distance
 
 
0.92 (0.90, 0.93)
 
 
ADE
 
 
0.41 (0.00, 0.54)
 
 
0.62
 
 
0.1055
 
 
1.0000
 
 
0.75 (0.00, 0.88)
 
 
TRUE
 
 
 
 
 MPGL 
 
 
mandible
 
 
distance
 
 
0.84 (0.82, 0.86)
 
 
ADE
 
 
0.33 (0.00, 0.50)
 
 
-0.74
 
 
0.2609
 
 
1.0000
 
 
0.62 (0.00, 0.84)
 
 
TRUE
 
 
 
 
 MPGR 
 
 
mandible
 
 
distance
 
 
0.84 (0.81, 0.86)
 
 
ADE
 
 
0.33 (0.00, 0.55)
 
 
-0.68
 
 
0.2511
 
 
1.0000
 
 
0.60 (0.00, 0.89)
 
 
TRUE
 
 
 
 
 MCPD 
 
 
mandible
 
 
distance
 
 
0.92 (0.90, 0.93)
 
 
ADE
 
 
0.31 (0.00, 0.52)
 
 
-0.49
 
 
0.2185
 
 
1.0000
 
 
0.57 (0.00, 0.87)
 
 
TRUE
 
 
 
 
 ACSN 
 
 
skull
 
 
angle
 
 
0.87 (0.85, 0.89)
 
 
ADE
 
 
0.41 (0.00, 0.78)
 
 
-0.97
 
 
0.3101
 
 
1.0000
 
 
0.50 (0.00, 0.85)
 
 
TRUE
 
 
 
 
 ABSN 
 
 
skull
 
 
angle
 
 
0.82 (0.79, 0.84)
 
 
ADE
 
 
0.34 (0.00, 0.76)
 
 
-1.25
 
 
0.3859
 
 
1.0000
 
 
0.42 (0.00, 0.84)
 
 
TRUE
 
 
 
 
 SSEN* 
 
 
skull
 
 
distance
 
 
0.90 (0.88, 0.91)
 
 
ADE
 
 
0.20 (0.00, 0.58)
 
 
-1.58
 
 
0.5147
 
 
1.0000
 
 
0.32 (0.00, 0.83)
 
 
TRUE
 
 
 
 
 DIPD 
 
 
dentition
 
 
distance
 
 
0.80 (0.77, 0.82)
 
 
ADE
 
 
0.23 (0.00, 0.57)
 
 
-1.66
 
 
0.5597
 
 
1.0000
 
 
0.32 (0.00, 0.73)
 
 
TRUE
 
 
 
 
 SBNP 
 
 
skull
 
 
distance
 
 
0.80 (0.77, 0.83)
 
 
ADE
 
 
0.11 (0.00, 0.66)
 
 
-1.91
 
 
0.7614
 
 
1.0000
 
 
0.16 (0.00, 0.85)
 
 
TRUE
 
 
 
 
 ANSF 
 
 
skull
 
 
angle
 
 
0.79 (0.75, 0.81)
 
 
ADE
 
 
0.04 (0.00, 0.65)
 
 
-1.99
 
 
0.9278
 
 
1.0000
 
 
0.05 (0.00, 0.83)
 
 
FALSE
 
 
 
 
 SBAS 
 
 
skull
 
 
distance
 
 
0.79 (0.76, 0.82)
 
 
ADE
 
 
0.01 (0.00, 0.56)
 
 
-2.00
 
 
0.9773
 
 
1.0000
 
 
0.01 (0.00, 0.84)
 
 
FALSE
 
 
 
 
 APNP 
 
 
skull
 
 
angle
 
 
0.76 (0.73, 0.79)
 
 
ADE
 
 
0.54 (0.00, 0.66)
 
 
0.53
 
 
0.1120
 
 
1.0000
 
 
0.71 (0.00, 0.79)
 
 
TRUE
 
 
 
 
 L3EA 
 
 
larynx
 
 
distance
 
 
0.76 (0.72, 0.79)
 
 
ADE
 
 
0.39 (0.00, 0.52)
 
 
-1.31
 
 
0.4056
 
 
1.0000
 
 
0.52 (0.00, 0.65)
 
 
FALSE
 
 
 
 
 CS2H 
 
 
cervical
 
 
distance
 
 
0.59 (0.53, 0.64)
 
 
ADE
 
 
0.41 (0.00, 0.50)
 
 
0.00
 
 
0.1573
 
 
1.0000
 
 
0.88 (0.00, 0.94)
 
 
TRUE
 
 
 
 
 MMPG 
 
 
mandible
 
 
distance
 
 
0.32 (0.24, 0.39)
 
 
ADE
 
 
0.24 (0.00, 0.32)
 
 
-0.54
 
 
0.2264
 
 
1.0000
 
 
0.84 (0.00, 1.00)
 
 
TRUE
 
 
 
 
 GRPD 
 
 
general
 
 
Procrustes dist.
 
 
0.30 (0.22, 0.36)
 
 
ADE
 
 
0.19 (0.00, 0.26)
 
 
-1.68
 
 
0.5730
 
 
1.0000
 
 
0.83 (0.00, 1.00)
 
 
TRUE
 
 
 
 
 PNNP 
 
 
pharynx
 
 
distance
 
 
0.74 (0.70, 0.77)
 
 
ADE
 
 
0.59 (0.00, 0.72)
 
 
0.55
 
 
0.1100
 
 
1.0000
 
 
0.81 (0.00, 0.88)
 
 
TRUE
 
 
 
 
 HPTH 
 
 
hard palate
 
 
distance
 
 
0.52 (0.46, 0.57)
 
 
ADE
 
 
0.37 (0.00, 0.47)
 
 
-0.79
 
 
0.2707
 
 
1.0000
 
 
0.76 (0.62, 0.76)
 
 
TRUE
 
 
 
 
 PPW2 
 
 
pharynx
 
 
curvature
 
 
0.09 (0.01, 0.17)
 
 
ADE
 
 
0.07 (0.00, 0.15)
 
 
-1.74
 
 
0.6124
 
 
1.0000
 
 
0.73 (0.00, 1.00)
 
 
TRUE
 
 
 
 
 DAIM 
 
 
dentition
 
 
angle
 
 
0.63 (0.58, 0.67)
 
 
ADE
 
 
0.40 (0.31, 0.51)
 
 
1.53
 
 
0.0604
 
 
1.0000
 
 
0.70 (0.58, 0.81)
 
 
TRUE
 
 
 
 
 DAIP 
 
 
dentition
 
 
angle
 
 
0.62 (0.57, 0.67)
 
 
ADE
 
 
0.40 (0.00, 0.50)
 
 
1.33
 
 
0.0680
 
 
1.0000
 
 
0.70 (0.00, 0.80)
 
 
TRUE
 
 
 
 
 DDAP 
 
 
dentition
 
 
Procrustes dist.
 
 
0.24 (0.17, 0.32)
 
 
ADE
 
 
0.16 (0.00, 0.23)
 
 
-1.61
 
 
0.5314
 
 
1.0000
 
 
0.69 (0.00, 1.00)
 
 
TRUE
 
 
 
 
 HCPP 
 
 
hard palate
 
 
Procrustes dist.
 
 
0.37 (0.30, 0.43)
 
 
ADE
 
 
0.18 (0.00, 0.27)
 
 
-1.36
 
 
0.4237
 
 
1.0000
 
 
0.55 (0.00, 0.78)
 
 
TRUE
 
 
 
 
 HIPL 
 
 
hard palate
 
 
distance
 
 
0.60 (0.55, 0.65)
 
 
ADE
 
 
0.28 (0.00, 0.40)
 
 
-1.47
 
 
0.4678
 
 
1.0000
 
 
0.53 (0.00, 0.69)
 
 
TRUE
 
 
 
 
 HCMP 
 
 
hard palate
 
 
Procrustes dist.
 
 
0.23 (0.15, 0.30)
 
 
ADE
 
 
0.13 (0.00, 0.22)
 
 
-0.77
 
 
0.2665
 
 
1.0000
 
 
0.53 (0.00, 0.87)
 
 
TRUE
 
 
 
 
 HMTH 
 
 
hard palate
 
 
distance
 
 
0.46 (0.40, 0.52)
 
 
ADE
 
 
0.21 (0.00, 0.36)
 
 
-1.63
 
 
0.5438
 
 
1.0000
 
 
0.49 (0.00, 0.79)
 
 
FALSE
 
 
 
 
 AANP 
 
 
skull
 
 
angle
 
 
0.40 (0.33, 0.47)
 
 
ADE
 
 
0.17 (0.00, 0.26)
 
 
-1.12
 
 
0.3489
 
 
1.0000
 
 
0.47 (0.00, 0.67)
 
 
TRUE
 
 
 
 
 LEPL 
 
 
larynx
 
 
distance
 
 
0.40 (0.34, 0.47)
 
 
ADE
 
 
0.15 (0.00, 0.24)
 
 
-1.14
 
 
0.3527
 
 
1.0000
 
 
0.46 (0.00, 0.68)
 
 
TRUE
 
 
 
 
 SIOD 
 
 
skull
 
 
distance
 
 
0.71 (0.67, 0.74)
 
 
ADE
 
 
0.17 (0.00, 0.50)
 
 
-1.55
 
 
0.5020
 
 
1.0000
 
 
0.38 (0.00, 1.00)
 
 
TRUE
 
 
 
 
 HMAC 
 
 
hard palate
 
 
curvature
 
 
0.08 (0.01, 0.16)
 
 
ADE
 
 
0.04 (0.00, 0.11)
 
 
-1.70
 
 
0.5818
 
 
1.0000
 
 
0.37 (0.00, 1.00)
 
 
TRUE
 
 
 
 
 PPW3 
 
 
pharynx
 
 
curvature
 
 
0.19 (0.12, 0.27)
 
 
ADE
 
 
0.07 (0.00, 0.14)
 
 
-1.85
 
 
0.6998
 
 
1.0000
 
 
0.36 (0.00, 0.76)
 
 
TRUE
 
 
 
 
 L4CT 
 
 
larynx
 
 
distance
 
 
0.46 (0.39, 0.52)
 
 
ADE
 
 
0.16 (0.00, 0.27)
 
 
-0.88
 
 
0.2898
 
 
1.0000
 
 
0.36 (0.00, 0.55)
 
 
TRUE
 
 
 
 
 HP2C 
 
 
hard palate
 
 
curvature
 
 
0.26 (0.19, 0.34)
 
 
ADE
 
 
0.09 (0.00, 0.17)
 
 
-1.63
 
 
0.5417
 
 
1.0000
 
 
0.34 (0.00, 0.66)
 
 
FALSE
 
 
 
 
 DICD 
 
 
dentition
 
 
distance
 
 
0.71 (0.67, 0.75)
 
 
ADE
 
 
0.21 (0.00, 0.51)
 
 
-1.74
 
 
0.6118
 
 
1.0000
 
 
0.34 (0.00, 0.75)
 
 
TRUE
 
 
 
 
 LHEP 
 
 
larynx
 
 
distance
 
 
0.47 (0.41, 0.53)
 
 
ADE
 
 
0.13 (0.00, 0.21)
 
 
-1.86
 
 
0.7061
 
 
1.0000
 
 
0.32 (0.00, 0.51)
 
 
TRUE
 
 
 
 
 APSN 
 
 
skull
 
 
angle
 
 
0.73 (0.69, 0.76)
 
 
ADE
 
 
0.22 (0.00, 0.71)
 
 
-1.67
 
 
0.5663
 
 
1.0000
 
 
0.32 (0.00, 0.91)
 
 
TRUE
 
 
 
 
 HM2C 
 
 
hard palate
 
 
curvature
 
 
0.23 (0.15, 0.30)
 
 
ADE
 
 
0.06 (0.00, 0.19)
 
 
-1.96
 
 
0.8386
 
 
1.0000
 
 
0.30 (0.00, 0.88)
 
 
FALSE
 
 
 
 
 LNEA 
 
 
larynx
 
 
distance
 
 
0.75 (0.71, 0.78)
 
 
ADE
 
 
0.15 (0.00, 0.38)
 
 
-1.82
 
 
0.6680
 
 
1.0000
 
 
0.27 (0.00, 0.63)
 
 
TRUE
 
 
 
 
 HDMP 
 
 
hard palate
 
 
angle
 
 
0.56 (0.50, 0.61)
 
 
ADE
 
 
0.12 (0.00, 0.21)
 
 
-1.78
 
 
0.6386
 
 
1.0000
 
 
0.27 (0.00, 0.44)
 
 
TRUE
 
 
 
 
 OCPR 
 
 
oral
 
 
ratio
 
 
0.52 (0.46, 0.57)
 
 
ADE
 
 
0.07 (0.00, 0.31)
 
 
-1.97
 
 
0.8743
 
 
1.0000
 
 
0.14 (0.00, 0.57)
 
 
FALSE
 
 
 
 
 DIIL 
 
 
dentition
 
 
distance
 
 
0.23 (0.15, 0.30)
 
 
ADE
 
 
0.03 (0.00, 0.24)
 
 
-1.99
 
 
0.9356
 
 
1.0000
 
 
0.12 (0.00, 1.00)
 
 
TRUE
 
 
 
 
 L2EA 
 
 
larynx
 
 
distance
 
 
0.65 (0.61, 0.70)
 
 
ADE
 
 
0.06 (0.00, 0.35)
 
 
-1.98
 
 
0.8895
 
 
1.0000
 
 
0.11 (0.00, 0.63)
 
 
TRUE
 
 
 
 
 OPMR 
 
 
oral
 
 
ratio
 
 
0.57 (0.52, 0.62)
 
 
ADE
 
 
0.05 (0.00, 0.40)
 
 
-1.98
 
 
0.8991
 
 
1.0000
 
 
0.10 (0.00, 0.69)
 
 
TRUE
 
 
 
 
 L5EP 
 
 
larynx
 
 
distance
 
 
0.66 (0.61, 0.70)
 
 
ADE
 
 
0.06 (0.00, 0.46)
 
 
-1.98
 
 
0.8907
 
 
1.0000
 
 
0.09 (0.00, 0.67)
 
 
TRUE
 
 
 
 
 HICL 
 
 
hard palate
 
 
distance
 
 
0.40 (0.33, 0.46)
 
 
ADE
 
 
0.03 (0.00, 0.34)
 
 
-1.99
 
 
0.9314
 
 
1.0000
 
 
0.09 (0.00, 0.87)
 
 
FALSE
 
 
 
 
 SNOR 
 
 
skull
 
 
distance
 
 
0.53 (0.47, 0.58)
 
 
ADE
 
 
0.03 (0.00, 0.42)
 
 
-1.99
 
 
0.9111
 
 
1.0000
 
 
0.09 (0.00, 1.00)
 
 
TRUE
 
 
 
 
 L4EP 
 
 
larynx
 
 
distance
 
 
0.44 (0.38, 0.50)
 
 
ADE
 
 
0.02 (0.00, 0.31)
 
 
-2.00
 
 
0.9510
 
 
1.0000
 
 
0.06 (0.00, 0.72)
 
 
FALSE
 
 
 
 
 ANNF 
 
 
skull
 
 
angle
 
 
0.74 (0.71, 0.78)
 
 
ADE
 
 
0.04 (0.00, 0.61)
 
 
-1.99
 
 
0.9271
 
 
1.0000
 
 
0.06 (0.00, 0.79)
 
 
FALSE
 
 
 
 
 HIMH 
 
 
hard palate
 
 
distance
 
 
0.47 (0.41, 0.53)
 
 
ADE
 
 
0.01 (0.00, 0.29)
 
 
-2.00
 
 
0.9754
 
 
1.0000
 
 
0.03 (0.00, 0.68)
 
 
FALSE
 
 
 
 
 CS6H 
 
 
cervical
 
 
distance
 
 
0.56 (0.50, 0.61)
 
 
ADE
 
 
0.00 (0.00, 0.39)
 
 
-2.00
 
 
0.9959
 
 
1.0000
 
 
0.00 (0.00, 0.78)
 
 
FALSE
 
 
 
 
 
 
  E  and  e  2  
 Relationship between the significance of  E  judged by AIC and
LRT is perfect: 
 
 
 
 
 
 
 
 
   
 FALSE 
 TRUE 
 
 
 
 
  FALSE  
 13 
 0 
 
 
  TRUE  
 0 
 133 
 
 
 
 but that between AIC( (or, equivalently, LRT) and the inclusion of 0
within the 95%CI of  d  2  is not, with the latter being
more liberal (i.e., finding more measures with a significant
 d  2 ): 
 
 
 
 
 
 
 
 
   
 FALSE 
 TRUE 
 
 
 
 
  FALSE  
 6 
 7 
 
 
  TRUE  
 0 
 133 
 
 
 
 
 
Measures with a significant  E  component, ordered by the
decreasing strength of evidence for  E , inter-rater agreement
and estimate of  d  2 . In  dark blue  measures
with a significant  E  component after Holm’s correction (122),
while in  blue  those only nominally significant (11); in
 bold  measures with  ICC(C,1)  > 0.75 (44), in
 bold italic  mesures with the lower 95%CI of  ICC(C,1) 
> 0.75 (37); in gray those with  d  2  < 0.20
(38). Column  c  2  > 0 is TRUE if and only if 0 is
not included in the 95% CI of  d  2  (this is a much
less strict test than ΔAIC and LTR).
 
 
 
 
measure
 
 
domain
 
 
type
 
 
 ICC ( C ,1)
 
 
genetic model
 
 
 C 
 
 
ΔAIC
 
 
 p 
 
 
 p (Holm)
 
 
 c  2 
 
 
 c  2 >0
 
 
 
 
 
 
 MICD 
 
 
mandible
 
 
distance
 
 
0.98 (0.98, 0.99)
 
 
ACE
 
 
0.00 (0.05, 0.08)
 
 
470.88
 
 
0.0000
 
 
0.0000
 
 
0.13 (0.10, 0.16)
 
 
TRUE
 
 
 
 
 SVTh* 
 
 
general
 
 
distance
 
 
0.93 (0.91, 0.94)
 
 
ACE
 
 
0.42 (0.12, 0.18)
 
 
251.94
 
 
0.0000
 
 
0.0000
 
 
0.19 (0.15, 0.24)
 
 
TRUE
 
 
 
 
 SVTh 
 
 
general
 
 
distance
 
 
0.90 (0.88, 0.91)
 
 
ACE
 
 
0.45 (0.14, 0.22)
 
 
219.87
 
 
0.0000
 
 
0.0000
 
 
0.24 (0.18, 0.30)
 
 
TRUE
 
 
 
 
 SSEG 
 
 
skull
 
 
distance
 
 
0.90 (0.88, 0.91)
 
 
ACE
 
 
0.01 (0.13, 0.22)
 
 
211.03
 
 
0.0000
 
 
0.0000
 
 
0.22 (0.17, 0.29)
 
 
TRUE
 
 
 
 
 SHBN 
 
 
skull
 
 
distance
 
 
0.96 (0.95, 0.96)
 
 
ACE
 
 
0.20 (0.05, 0.08)
 
 
192.00
 
 
0.0000
 
 
0.0000
 
 
0.10 (0.08, 0.13)
 
 
TRUE
 
 
 
 
 HBPG 
 
 
hyoid
 
 
distance
 
 
0.81 (0.78, 0.83)
 
 
ACE
 
 
0.16 (0.23, 0.37)
 
 
191.38
 
 
0.0000
 
 
0.0000
 
 
0.40 (0.31, 0.51)
 
 
TRUE
 
 
 
 
 ASCG* 
 
 
skull
 
 
angle
 
 
0.87 (0.85, 0.89)
 
 
ACE
 
 
0.12 (0.13, 0.21)
 
 
179.85
 
 
0.0000
 
 
0.0000
 
 
0.20 (0.15, 0.26)
 
 
TRUE
 
 
 
 
 MCGM 
 
 
mandible
 
 
angle
 
 
0.89 (0.87, 0.90)
 
 
ACE
 
 
0.12 (0.13, 0.21)
 
 
179.85
 
 
0.0000
 
 
0.0000
 
 
0.20 (0.15, 0.26)
 
 
TRUE
 
 
 
 
 L3EA 
 
 
larynx
 
 
distance
 
 
0.76 (0.72, 0.79)
 
 
ADE
 
 
0.39 (0.26, 0.43)
 
 
172.03
 
 
0.0000
 
 
0.0000
 
 
0.45 (0.35, 0.58)
 
 
TRUE
 
 
 
 
 HBC4 
 
 
hyoid
 
 
distance
 
 
0.79 (0.76, 0.82)
 
 
ACE
 
 
0.09 (0.21, 0.35)
 
 
165.72
 
 
0.0000
 
 
0.0000
 
 
0.36 (0.28, 0.46)
 
 
TRUE
 
 
 
 
 SHSW 
 
 
skull
 
 
distance
 
 
0.94 (0.93, 0.95)
 
 
ACE
 
 
0.08 (0.06, 0.10)
 
 
161.11
 
 
0.0000
 
 
0.0000
 
 
0.18 (0.14, 0.24)
 
 
TRUE
 
 
 
 
 SSEN* 
 
 
skull
 
 
distance
 
 
0.90 (0.88, 0.91)
 
 
ADE
 
 
0.20 (0.10, 0.17)
 
 
158.96
 
 
0.0000
 
 
0.0000
 
 
0.22 (0.16, 0.28)
 
 
TRUE
 
 
 
 
 DIPD 
 
 
dentition
 
 
distance
 
 
0.80 (0.77, 0.82)
 
 
ADE
 
 
0.23 (0.19, 0.32)
 
 
153.77
 
 
0.0000
 
 
0.0000
 
 
0.35 (0.27, 0.46)
 
 
TRUE
 
 
 
 
 ACSN 
 
 
skull
 
 
angle
 
 
0.87 (0.85, 0.89)
 
 
ADE
 
 
0.41 (0.13, 0.21)
 
 
153.53
 
 
0.0000
 
 
0.0000
 
 
0.20 (0.15, 0.26)
 
 
TRUE
 
 
 
 
 LAEA 
 
 
larynx
 
 
distance
 
 
0.77 (0.73, 0.80)
 
 
ACE
 
 
0.13 (0.22, 0.37)
 
 
151.21
 
 
0.0000
 
 
0.0000
 
 
0.49 (0.38, 0.62)
 
 
TRUE
 
 
 
 
 HBPL 
 
 
hard palate
 
 
distance
 
 
0.91 (0.89, 0.92)
 
 
ACE
 
 
0.32 (0.09, 0.15)
 
 
150.85
 
 
0.0000
 
 
0.0000
 
 
0.16 (0.12, 0.21)
 
 
TRUE
 
 
 
 
 L4EA 
 
 
larynx
 
 
distance
 
 
0.76 (0.72, 0.79)
 
 
ACE
 
 
0.12 (0.22, 0.37)
 
 
146.42
 
 
0.0000
 
 
0.0000
 
 
0.40 (0.31, 0.51)
 
 
TRUE
 
 
 
 
 MCPD 
 
 
mandible
 
 
distance
 
 
0.92 (0.90, 0.93)
 
 
ADE
 
 
0.31 (0.07, 0.12)
 
 
140.64
 
 
0.0000
 
 
0.0000
 
 
0.18 (0.13, 0.23)
 
 
TRUE
 
 
 
 
 APNS 
 
 
skull
 
 
angle
 
 
0.83 (0.80, 0.85)
 
 
ACE
 
 
0.01 (0.15, 0.25)
 
 
133.73
 
 
0.0000
 
 
0.0000
 
 
0.25 (0.19, 0.33)
 
 
TRUE
 
 
 
 
 MCGP 
 
 
mandible
 
 
angle
 
 
0.87 (0.84, 0.88)
 
 
ACE
 
 
0.06 (0.11, 0.19)
 
 
130.56
 
 
0.0000
 
 
0.0000
 
 
0.18 (0.14, 0.24)
 
 
TRUE
 
 
 
 
 DAIS 
 
 
dentition
 
 
angle
 
 
0.65 (0.60, 0.69)
 
 
ACE
 
 
0.11 (0.28, 0.47)
 
 
130.38
 
 
0.0000
 
 
0.0000
 
 
0.59 (0.46, 0.72)
 
 
TRUE
 
 
 
 
 SBNA 
 
 
skull
 
 
distance
 
 
0.90 (0.88, 0.91)
 
 
ACE
 
 
0.11 (0.08, 0.14)
 
 
126.66
 
 
0.0000
 
 
0.0000
 
 
0.17 (0.12, 0.22)
 
 
TRUE
 
 
 
 
 MIGD 
 
 
mandible
 
 
distance
 
 
0.95 (0.94, 0.95)
 
 
ACE
 
 
0.09 (0.04, 0.07)
 
 
121.01
 
 
0.0000
 
 
0.0000
 
 
0.12 (0.09, 0.17)
 
 
TRUE
 
 
 
 
 LNEA 
 
 
larynx
 
 
distance
 
 
0.75 (0.71, 0.78)
 
 
ADE
 
 
0.15 (0.20, 0.35)
 
 
120.70
 
 
0.0000
 
 
0.0000
 
 
0.48 (0.37, 0.62)
 
 
TRUE
 
 
 
 
 L5EA 
 
 
larynx
 
 
distance
 
 
0.74 (0.70, 0.77)
 
 
ACE
 
 
0.12 (0.20, 0.35)
 
 
120.69
 
 
0.0000
 
 
0.0000
 
 
0.39 (0.30, 0.51)
 
 
TRUE
 
 
 
 
 SNAM 
 
 
skull
 
 
distance
 
 
0.90 (0.89, 0.92)
 
 
ACE
 
 
0.03 (0.08, 0.13)
 
 
120.26
 
 
0.0000
 
 
0.0000
 
 
0.17 (0.12, 0.22)
 
 
TRUE
 
 
 
 
 MCMD 
 
 
mandible
 
 
distance
 
 
0.92 (0.90, 0.93)
 
 
ADE
 
 
0.41 (0.07, 0.11)
 
 
119.89
 
 
0.0000
 
 
0.0000
 
 
0.16 (0.12, 0.21)
 
 
TRUE
 
 
 
 
 GAPD 
 
 
general
 
 
Procrustes dist.
 
 
0.80 (0.77, 0.82)
 
 
ACE
 
 
0.47 (0.16, 0.26)
 
 
118.53
 
 
0.0000
 
 
0.0000
 
 
0.30 (0.23, 0.39)
 
 
TRUE
 
 
 
 
 LACT 
 
 
larynx
 
 
distance
 
 
0.81 (0.78, 0.83)
 
 
ACE
 
 
0.25 (0.14, 0.25)
 
 
113.85
 
 
0.0000
 
 
0.0000
 
 
0.36 (0.27, 0.46)
 
 
TRUE
 
 
 
 
 ABSN 
 
 
skull
 
 
angle
 
 
0.82 (0.79, 0.84)
 
 
ADE
 
 
0.34 (0.13, 0.23)
 
 
108.28
 
 
0.0000
 
 
0.0000
 
 
0.21 (0.16, 0.29)
 
 
TRUE
 
 
 
 
 APNP 
 
 
skull
 
 
angle
 
 
0.76 (0.73, 0.79)
 
 
ADE
 
 
0.54 (0.16, 0.28)
 
 
106.96
 
 
0.0000
 
 
0.0000
 
 
0.29 (0.21, 0.37)
 
 
TRUE
 
 
 
 
 L5CT 
 
 
larynx
 
 
distance
 
 
0.71 (0.67, 0.75)
 
 
ACE
 
 
0.27 (0.20, 0.35)
 
 
105.61
 
 
0.0000
 
 
0.0000
 
 
0.43 (0.32, 0.56)
 
 
TRUE
 
 
 
 
 SSEN 
 
 
skull
 
 
distance
 
 
0.90 (0.89, 0.92)
 
 
ACE
 
 
0.27 (0.06, 0.11)
 
 
103.07
 
 
0.0000
 
 
0.0000
 
 
0.13 (0.09, 0.17)
 
 
TRUE
 
 
 
 
 DIMD 
 
 
dentition
 
 
distance
 
 
0.77 (0.73, 0.80)
 
 
ACE
 
 
0.25 (0.15, 0.26)
 
 
94.10
 
 
0.0000
 
 
0.0000
 
 
0.32 (0.23, 0.42)
 
 
TRUE
 
 
 
 
 LAEP 
 
 
larynx
 
 
distance
 
 
0.74 (0.70, 0.77)
 
 
ACE
 
 
0.25 (0.16, 0.28)
 
 
88.69
 
 
0.0000
 
 
0.0000
 
 
0.43 (0.32, 0.55)
 
 
TRUE
 
 
 
 
 L3CT 
 
 
larynx
 
 
distance
 
 
0.69 (0.64, 0.73)
 
 
ACE
 
 
0.34 (0.19, 0.33)
 
 
88.34
 
 
0.0000
 
 
0.0000
 
 
0.42 (0.32, 0.54)
 
 
TRUE
 
 
 
 
 L5EP 
 
 
larynx
 
 
distance
 
 
0.66 (0.61, 0.70)
 
 
ADE
 
 
0.06 (0.20, 0.36)
 
 
87.98
 
 
0.0000
 
 
0.0000
 
 
0.44 (0.32, 0.57)
 
 
TRUE
 
 
 
 
 L2CT 
 
 
larynx
 
 
distance
 
 
0.73 (0.69, 0.76)
 
 
ACE
 
 
0.34 (0.15, 0.28)
 
 
84.48
 
 
0.0000
 
 
0.0000
 
 
0.37 (0.27, 0.48)
 
 
TRUE
 
 
 
 
 SNAP 
 
 
skull
 
 
distance
 
 
0.88 (0.86, 0.90)
 
 
ACE
 
 
0.03 (0.07, 0.13)
 
 
83.63
 
 
0.0000
 
 
0.0000
 
 
0.16 (0.12, 0.22)
 
 
TRUE
 
 
 
 
 HBNP 
 
 
hyoid
 
 
distance
 
 
0.84 (0.81, 0.86)
 
 
ACE
 
 
0.06 (0.09, 0.17)
 
 
82.80
 
 
0.0000
 
 
0.0000
 
 
0.30 (0.22, 0.40)
 
 
TRUE
 
 
 
 
 MPGL 
 
 
mandible
 
 
distance
 
 
0.84 (0.82, 0.86)
 
 
ADE
 
 
0.33 (0.09, 0.16)
 
 
82.77
 
 
0.0000
 
 
0.0000
 
 
0.22 (0.16, 0.30)
 
 
TRUE
 
 
 
 
 ANNF 
 
 
skull
 
 
angle
 
 
0.74 (0.71, 0.78)
 
 
ADE
 
 
0.04 (0.14, 0.26)
 
 
82.61
 
 
0.0000
 
 
0.0000
 
 
0.28 (0.20, 0.37)
 
 
TRUE
 
 
 
 
 L2EA 
 
 
larynx
 
 
distance
 
 
0.65 (0.61, 0.70)
 
 
ADE
 
 
0.06 (0.18, 0.33)
 
 
82.35
 
 
0.0000
 
 
0.0000
 
 
0.49 (0.37, 0.63)
 
 
TRUE
 
 
 
 
 ANSF 
 
 
skull
 
 
angle
 
 
0.79 (0.75, 0.81)
 
 
ADE
 
 
0.04 (0.12, 0.21)
 
 
78.46
 
 
0.0000
 
 
0.0000
 
 
0.22 (0.16, 0.30)
 
 
TRUE
 
 
 
 
 DICD 
 
 
dentition
 
 
distance
 
 
0.71 (0.67, 0.75)
 
 
ADE
 
 
0.21 (0.16, 0.30)
 
 
78.02
 
 
0.0000
 
 
0.0000
 
 
0.35 (0.25, 0.47)
 
 
TRUE
 
 
 
 
 SBNP 
 
 
skull
 
 
distance
 
 
0.80 (0.77, 0.83)
 
 
ADE
 
 
0.11 (0.10, 0.19)
 
 
74.80
 
 
0.0000
 
 
0.0000
 
 
0.20 (0.14, 0.27)
 
 
TRUE
 
 
 
 
 SVTv* 
 
 
general
 
 
distance
 
 
0.80 (0.77, 0.83)
 
 
ACE
 
 
0.19 (0.10, 0.19)
 
 
74.74
 
 
0.0000
 
 
0.0000
 
 
0.32 (0.23, 0.43)
 
 
TRUE
 
 
 
 
 DAII 
 
 
dentition
 
 
angle
 
 
0.66 (0.61, 0.70)
 
 
ADE
 
 
0.40 (0.17, 0.32)
 
 
74.31
 
 
0.0000
 
 
0.0000
 
 
0.37 (0.26, 0.49)
 
 
TRUE
 
 
 
 
 HDMP 
 
 
hard palate
 
 
angle
 
 
0.56 (0.50, 0.61)
 
 
ADE
 
 
0.12 (0.24, 0.44)
 
 
70.65
 
 
0.0000
 
 
0.0000
 
 
0.73 (0.56, 0.90)
 
 
TRUE
 
 
 
 
 HBC2 
 
 
hyoid
 
 
distance
 
 
0.78 (0.74, 0.81)
 
 
ACE
 
 
0.12 (0.11, 0.21)
 
 
69.78
 
 
0.0000
 
 
0.0000
 
 
0.37 (0.26, 0.49)
 
 
TRUE
 
 
 
 
 LHEA 
 
 
larynx
 
 
distance
 
 
0.61 (0.56, 0.66)
 
 
ACE
 
 
0.05 (0.18, 0.34)
 
 
69.53
 
 
0.0000
 
 
0.0000
 
 
0.63 (0.48, 0.80)
 
 
TRUE
 
 
 
 
 CS7H 
 
 
cervical
 
 
distance
 
 
0.69 (0.64, 0.72)
 
 
ACE
 
 
0.24 (0.15, 0.29)
 
 
66.27
 
 
0.0000
 
 
0.0000
 
 
0.35 (0.25, 0.47)
 
 
TRUE
 
 
 
 
 HIPL* 
 
 
hard palate
 
 
distance
 
 
0.63 (0.58, 0.67)
 
 
ACE
 
 
0.23 (0.17, 0.33)
 
 
64.62
 
 
0.0000
 
 
0.0000
 
 
0.43 (0.31, 0.57)
 
 
TRUE
 
 
 
 
 OMLW 
 
 
oral
 
 
ratio
 
 
0.70 (0.65, 0.73)
 
 
ACE
 
 
0.27 (0.14, 0.26)
 
 
64.39
 
 
0.0000
 
 
0.0000
 
 
0.38 (0.27, 0.51)
 
 
TRUE
 
 
 
 
 MGPD 
 
 
mandible
 
 
distance
 
 
0.83 (0.80, 0.85)
 
 
ADE
 
 
0.50 (0.08, 0.15)
 
 
63.75
 
 
0.0000
 
 
0.0000
 
 
0.17 (0.12, 0.23)
 
 
TRUE
 
 
 
 
 HACP 
 
 
hard palate
 
 
angle
 
 
0.47 (0.41, 0.53)
 
 
ACE
 
 
0.11 (0.23, 0.43)
 
 
62.95
 
 
0.0000
 
 
0.0000
 
 
0.75 (0.57, 0.93)
 
 
TRUE
 
 
 
 
 SVTv 
 
 
general
 
 
distance
 
 
0.73 (0.70, 0.77)
 
 
ACE
 
 
0.25 (0.11, 0.21)
 
 
61.86
 
 
0.0000
 
 
0.0000
 
 
0.38 (0.28, 0.50)
 
 
TRUE
 
 
 
 
 OCPR 
 
 
oral
 
 
ratio
 
 
0.52 (0.46, 0.57)
 
 
ADE
 
 
0.07 (0.21, 0.41)
 
 
61.84
 
 
0.0000
 
 
0.0000
 
 
0.59 (0.43, 0.76)
 
 
TRUE
 
 
 
 
 SBAN 
 
 
skull
 
 
distance
 
 
0.89 (0.88, 0.91)
 
 
ACE
 
 
0.13 (0.05, 0.09)
 
 
61.03
 
 
0.0000
 
 
0.0000
 
 
0.11 (0.08, 0.16)
 
 
TRUE
 
 
 
 
 SBAS 
 
 
skull
 
 
distance
 
 
0.79 (0.76, 0.82)
 
 
ADE
 
 
0.01 (0.09, 0.18)
 
 
60.71
 
 
0.0000
 
 
0.0000
 
 
0.20 (0.14, 0.28)
 
 
TRUE
 
 
 
 
 L2EP 
 
 
larynx
 
 
distance
 
 
0.67 (0.62, 0.71)
 
 
ACE
 
 
0.17 (0.14, 0.28)
 
 
60.55
 
 
0.0000
 
 
0.0000
 
 
0.39 (0.28, 0.52)
 
 
TRUE
 
 
 
 
 HIPL 
 
 
hard palate
 
 
distance
 
 
0.60 (0.55, 0.65)
 
 
ADE
 
 
0.28 (0.16, 0.31)
 
 
56.05
 
 
0.0000
 
 
0.0000
 
 
0.43 (0.31, 0.58)
 
 
TRUE
 
 
 
 
 HBC3 
 
 
hyoid
 
 
distance
 
 
0.71 (0.67, 0.74)
 
 
ACE
 
 
0.06 (0.12, 0.24)
 
 
54.78
 
 
0.0000
 
 
0.0000
 
 
0.34 (0.24, 0.47)
 
 
TRUE
 
 
 
 
 OPMR 
 
 
oral
 
 
ratio
 
 
0.57 (0.52, 0.62)
 
 
ADE
 
 
0.05 (0.16, 0.33)
 
 
52.99
 
 
0.0000
 
 
0.0000
 
 
0.44 (0.31, 0.58)
 
 
TRUE
 
 
 
 
 MPGR 
 
 
mandible
 
 
distance
 
 
0.84 (0.81, 0.86)
 
 
ADE
 
 
0.33 (0.00, 0.12)
 
 
51.95
 
 
0.0000
 
 
0.0000
 
 
0.16 (0.11, 0.23)
 
 
TRUE
 
 
 
 
 HNSL 
 
 
hard palate
 
 
distance
 
 
0.77 (0.74, 0.80)
 
 
ACE
 
 
0.30 (0.00, 0.17)
 
 
49.35
 
 
0.0000
 
 
0.0000
 
 
0.20 (0.14, 0.28)
 
 
TRUE
 
 
 
 
 HAPL 
 
 
hard palate
 
 
distance
 
 
0.52 (0.46, 0.57)
 
 
ACE
 
 
0.16 (0.17, 0.35)
 
 
49.11
 
 
0.0000
 
 
0.0000
 
 
0.54 (0.38, 0.70)
 
 
TRUE
 
 
 
 
 LHCT 
 
 
larynx
 
 
distance
 
 
0.66 (0.62, 0.70)
 
 
ACE
 
 
0.04 (0.15, 0.25)
 
 
48.05
 
 
0.0000
 
 
0.0000
 
 
0.38 (0.26, 0.52)
 
 
TRUE
 
 
 
 
 SPUN 
 
 
soft palate
 
 
distance
 
 
0.63 (0.58, 0.67)
 
 
ACE
 
 
0.12 (0.00, 0.27)
 
 
46.72
 
 
0.0000
 
 
0.0000
 
 
0.34 (0.23, 0.47)
 
 
TRUE
 
 
 
 
 L4CT 
 
 
larynx
 
 
distance
 
 
0.46 (0.39, 0.52)
 
 
ADE
 
 
0.16 (0.20, 0.42)
 
 
46.67
 
 
0.0000
 
 
0.0000
 
 
0.64 (0.45, 0.86)
 
 
TRUE
 
 
 
 
 LHEP 
 
 
larynx
 
 
distance
 
 
0.47 (0.41, 0.53)
 
 
ADE
 
 
0.13 (0.18, 0.37)
 
 
45.52
 
 
0.0000
 
 
0.0000
 
 
0.68 (0.49, 0.87)
 
 
TRUE
 
 
 
 
 OPLW 
 
 
oral
 
 
ratio
 
 
0.50 (0.44, 0.56)
 
 
ACE
 
 
0.13 (0.17, 0.35)
 
 
44.75
 
 
0.0000
 
 
0.0000
 
 
0.56 (0.39, 0.74)
 
 
TRUE
 
 
 
 
 PNNP 
 
 
pharynx
 
 
distance
 
 
0.74 (0.70, 0.77)
 
 
ADE
 
 
0.59 (0.00, 0.18)
 
 
44.41
 
 
0.0000
 
 
0.0000
 
 
0.18 (0.12, 0.25)
 
 
TRUE
 
 
 
 
 PPW7 
 
 
pharynx
 
 
curvature
 
 
0.71 (0.67, 0.74)
 
 
ACE
 
 
0.22 (0.00, 0.20)
 
 
43.67
 
 
0.0000
 
 
0.0000
 
 
0.28 (0.19, 0.39)
 
 
TRUE
 
 
 
 
 OMHW 
 
 
oral
 
 
ratio
 
 
0.48 (0.42, 0.54)
 
 
ACE
 
 
0.08 (0.00, 0.35)
 
 
43.24
 
 
0.0000
 
 
0.0000
 
 
0.53 (0.37, 0.70)
 
 
TRUE
 
 
 
 
 L3EP 
 
 
larynx
 
 
distance
 
 
0.57 (0.52, 0.62)
 
 
ACE
 
 
0.15 (0.00, 0.29)
 
 
41.79
 
 
0.0000
 
 
0.0000
 
 
0.41 (0.28, 0.56)
 
 
TRUE
 
 
 
 
 HHPL 
 
 
hard palate
 
 
distance
 
 
0.50 (0.44, 0.56)
 
 
ACE
 
 
0.24 (0.00, 0.31)
 
 
40.28
 
 
0.0000
 
 
0.0000
 
 
0.49 (0.34, 0.65)
 
 
TRUE
 
 
 
 
 OPHW 
 
 
oral
 
 
ratio
 
 
0.50 (0.43, 0.55)
 
 
ACE
 
 
0.04 (0.00, 0.33)
 
 
38.90
 
 
0.0000
 
 
0.0000
 
 
0.49 (0.34, 0.67)
 
 
TRUE
 
 
 
 
 MGMD 
 
 
mandible
 
 
distance
 
 
0.80 (0.77, 0.83)
 
 
ADE
 
 
0.53 (0.00, 0.13)
 
 
38.79
 
 
0.0000
 
 
0.0000
 
 
0.15 (0.10, 0.22)
 
 
TRUE
 
 
 
 
 DAIP 
 
 
dentition
 
 
angle
 
 
0.62 (0.57, 0.67)
 
 
ADE
 
 
0.40 (0.00, 0.24)
 
 
38.58
 
 
0.0000
 
 
0.0000
 
 
0.30 (0.20, 0.42)
 
 
TRUE
 
 
 
 
 MCGL 
 
 
mandible
 
 
distance
 
 
0.68 (0.64, 0.72)
 
 
ACE
 
 
0.08 (0.00, 0.22)
 
 
38.31
 
 
0.0000
 
 
0.0000
 
 
0.34 (0.22, 0.48)
 
 
TRUE
 
 
 
 
 DAIM 
 
 
dentition
 
 
angle
 
 
0.63 (0.58, 0.67)
 
 
ADE
 
 
0.40 (0.00, 0.24)
 
 
38.25
 
 
0.0000
 
 
0.0000
 
 
0.30 (0.19, 0.42)
 
 
TRUE
 
 
 
 
 HIML* 
 
 
hard palate
 
 
distance
 
 
0.65 (0.60, 0.69)
 
 
ACE
 
 
0.25 (0.00, 0.23)
 
 
36.95
 
 
0.0000
 
 
0.0000
 
 
0.29 (0.19, 0.41)
 
 
TRUE
 
 
 
 
 OMHL 
 
 
oral
 
 
ratio
 
 
0.44 (0.37, 0.50)
 
 
ACE
 
 
0.04 (0.00, 0.34)
 
 
34.14
 
 
0.0000
 
 
0.0000
 
 
0.59 (0.40, 0.79)
 
 
TRUE
 
 
 
 
 ALNS 
 
 
skull
 
 
angle
 
 
0.60 (0.55, 0.65)
 
 
ACE
 
 
0.27 (0.00, 0.24)
 
 
33.92
 
 
0.0000
 
 
0.0000
 
 
0.29 (0.18, 0.41)
 
 
TRUE
 
 
 
 
 CS4H 
 
 
cervical
 
 
distance
 
 
0.59 (0.53, 0.64)
 
 
ACE
 
 
0.21 (0.00, 0.24)
 
 
33.70
 
 
0.0000
 
 
0.0000
 
 
0.44 (0.30, 0.60)
 
 
TRUE
 
 
 
 
 OCMR 
 
 
oral
 
 
ratio
 
 
0.47 (0.40, 0.53)
 
 
ACE
 
 
0.11 (0.00, 0.31)
 
 
29.54
 
 
0.0000
 
 
0.0000
 
 
0.47 (0.30, 0.66)
 
 
TRUE
 
 
 
 
 MCGR 
 
 
mandible
 
 
distance
 
 
0.68 (0.64, 0.72)
 
 
ACE
 
 
0.05 (0.00, 0.18)
 
 
29.43
 
 
0.0000
 
 
0.0000
 
 
0.29 (0.18, 0.42)
 
 
TRUE
 
 
 
 
 HIML 
 
 
hard palate
 
 
distance
 
 
0.63 (0.58, 0.67)
 
 
ACE
 
 
0.13 (0.00, 0.21)
 
 
28.83
 
 
0.0000
 
 
0.0000
 
 
0.29 (0.18, 0.43)
 
 
TRUE
 
 
 
 
 HIMH 
 
 
hard palate
 
 
distance
 
 
0.47 (0.41, 0.53)
 
 
ADE
 
 
0.01 (0.00, 0.29)
 
 
28.14
 
 
0.0000
 
 
0.0000
 
 
0.50 (0.32, 0.69)
 
 
TRUE
 
 
 
 
 ASNP 
 
 
skull
 
 
angle
 
 
0.72 (0.68, 0.76)
 
 
ACE
 
 
0.08 (0.00, 0.16)
 
 
28.15
 
 
0.0000
 
 
0.0000
 
 
0.16 (0.09, 0.23)
 
 
TRUE
 
 
 
 
 APSN 
 
 
skull
 
 
angle
 
 
0.73 (0.69, 0.76)
 
 
ADE
 
 
0.22 (0.00, 0.15)
 
 
27.04
 
 
0.0000
 
 
0.0000
 
 
0.14 (0.09, 0.21)
 
 
TRUE
 
 
 
 
 CS6H 
 
 
cervical
 
 
distance
 
 
0.56 (0.50, 0.61)
 
 
ADE
 
 
0.00 (0.00, 0.24)
 
 
26.29
 
 
0.0000
 
 
0.0000
 
 
0.35 (0.21, 0.50)
 
 
TRUE
 
 
 
 
 HARC 
 
 
hard palate
 
 
angle
 
 
0.36 (0.29, 0.42)
 
 
ACE
 
 
0.03 (0.00, 0.33)
 
 
23.53
 
 
0.0000
 
 
0.0000
 
 
0.66 (0.41, 0.90)
 
 
TRUE
 
 
 
 
 HAML 
 
 
hard palate
 
 
distance
 
 
0.56 (0.50, 0.61)
 
 
ACE
 
 
0.28 (0.12, 0.22)
 
 
23.52
 
 
0.0000
 
 
0.0000
 
 
0.34 (0.20, 0.48)
 
 
TRUE
 
 
 
 
 OPHL 
 
 
oral
 
 
ratio
 
 
0.38 (0.31, 0.44)
 
 
ACE
 
 
0.09 (0.00, 0.30)
 
 
22.14
 
 
0.0000
 
 
0.0000
 
 
0.53 (0.33, 0.74)
 
 
TRUE
 
 
 
 
 L4EP 
 
 
larynx
 
 
distance
 
 
0.44 (0.38, 0.50)
 
 
ADE
 
 
0.02 (0.00, 0.28)
 
 
22.06
 
 
0.0000
 
 
0.0000
 
 
0.46 (0.28, 0.65)
 
 
TRUE
 
 
 
 
 AANP 
 
 
skull
 
 
angle
 
 
0.40 (0.33, 0.47)
 
 
ADE
 
 
0.17 (0.00, 0.29)
 
 
21.92
 
 
0.0000
 
 
0.0000
 
 
0.53 (0.33, 0.75)
 
 
TRUE
 
 
 
 
 HIPH 
 
 
hard palate
 
 
distance
 
 
0.43 (0.36, 0.49)
 
 
ACE
 
 
0.11 (0.00, 0.28)
 
 
21.88
 
 
0.0000
 
 
0.0000
 
 
0.46 (0.28, 0.65)
 
 
TRUE
 
 
 
 
 HICH 
 
 
hard palate
 
 
distance
 
 
0.39 (0.32, 0.46)
 
 
ACE
 
 
0.11 (0.00, 0.30)
 
 
21.34
 
 
0.0000
 
 
0.0001
 
 
0.53 (0.32, 0.75)
 
 
TRUE
 
 
 
 
 PWTP 
 
 
pharynx
 
 
Procrustes dist.
 
 
0.48 (0.42, 0.54)
 
 
ACE
 
 
0.20 (0.00, 0.25)
 
 
21.26
 
 
0.0000
 
 
0.0001
 
 
0.46 (0.29, 0.64)
 
 
TRUE
 
 
 
 
 OCLW 
 
 
oral
 
 
ratio
 
 
0.25 (0.17, 0.32)
 
 
ACE
 
 
0.00 (0.00, 0.33)
 
 
20.68
 
 
0.0000
 
 
0.0001
 
 
0.78 (0.50, 1.00)
 
 
TRUE
 
 
 
 
 OCHW 
 
 
oral
 
 
ratio
 
 
0.41 (0.34, 0.47)
 
 
ACE
 
 
0.13 (0.00, 0.28)
 
 
18.82
 
 
0.0000
 
 
0.0002
 
 
0.47 (0.27, 0.68)
 
 
TRUE
 
 
 
 
 LEPL 
 
 
larynx
 
 
distance
 
 
0.40 (0.34, 0.47)
 
 
ADE
 
 
0.15 (0.00, 0.28)
 
 
18.17
 
 
0.0000
 
 
0.0003
 
 
0.54 (0.32, 0.78)
 
 
TRUE
 
 
 
 
 HMTH 
 
 
hard palate
 
 
distance
 
 
0.46 (0.40, 0.52)
 
 
ADE
 
 
0.21 (0.00, 0.25)
 
 
18.01
 
 
0.0000
 
 
0.0003
 
 
0.37 (0.21, 0.56)
 
 
TRUE
 
 
 
 
 HCTH 
 
 
hard palate
 
 
distance
 
 
0.44 (0.37, 0.50)
 
 
ACE
 
 
0.12 (0.00, 0.25)
 
 
17.27
 
 
0.0000
 
 
0.0005
 
 
0.47 (0.27, 0.67)
 
 
TRUE
 
 
 
 
 CS5H 
 
 
cervical
 
 
distance
 
 
0.43 (0.37, 0.50)
 
 
ACE
 
 
0.02 (0.00, 0.23)
 
 
14.94
 
 
0.0000
 
 
0.0015
 
 
0.40 (0.22, 0.59)
 
 
TRUE
 
 
 
 
 MCGD 
 
 
mandible
 
 
distance
 
 
0.71 (0.67, 0.75)
 
 
ACE
 
 
0.01 (0.00, 0.12)
 
 
14.72
 
 
0.0000
 
 
0.0017
 
 
0.18 (0.09, 0.29)
 
 
TRUE
 
 
 
 
 AASN 
 
 
skull
 
 
angle
 
 
0.63 (0.58, 0.67)
 
 
ACE
 
 
0.10 (0.00, 0.16)
 
 
14.05
 
 
0.0001
 
 
0.0023
 
 
0.17 (0.08, 0.26)
 
 
TRUE
 
 
 
 
 SNNP 
 
 
skull
 
 
distance
 
 
0.82 (0.80, 0.85)
 
 
ACE
 
 
0.16 (0.00, 0.07)
 
 
13.74
 
 
0.0001
 
 
0.0027
 
 
0.08 (0.04, 0.13)
 
 
TRUE
 
 
 
 
 DSIL 
 
 
dentition
 
 
distance
 
 
0.45 (0.39, 0.51)
 
 
ACE
 
 
0.13 (0.00, 0.22)
 
 
12.99
 
 
0.0001
 
 
0.0039
 
 
0.35 (0.18, 0.53)
 
 
TRUE
 
 
 
 
 PPW5 
 
 
pharynx
 
 
curvature
 
 
0.41 (0.35, 0.48)
 
 
ACE
 
 
0.14 (0.00, 0.21)
 
 
12.59
 
 
0.0001
 
 
0.0047
 
 
0.47 (0.24, 0.69)
 
 
TRUE
 
 
 
 
 HPTH 
 
 
hard palate
 
 
distance
 
 
0.52 (0.46, 0.57)
 
 
ADE
 
 
0.37 (0.00, 0.19)
 
 
12.05
 
 
0.0002
 
 
0.0061
 
 
0.24 (0.14, 0.38)
 
 
TRUE
 
 
 
 
 HCPP 
 
 
hard palate
 
 
Procrustes dist.
 
 
0.37 (0.30, 0.43)
 
 
ADE
 
 
0.18 (0.00, 0.24)
 
 
11.75
 
 
0.0002
 
 
0.0069
 
 
0.45 (0.22, 0.69)
 
 
TRUE
 
 
 
 
 PPW4 
 
 
pharynx
 
 
curvature
 
 
0.31 (0.24, 0.38)
 
 
ACE
 
 
0.09 (0.00, 0.23)
 
 
11.49
 
 
0.0002
 
 
0.0077
 
 
0.54 (0.28, 0.79)
 
 
TRUE
 
 
 
 
 HP2C 
 
 
hard palate
 
 
curvature
 
 
0.26 (0.19, 0.34)
 
 
ADE
 
 
0.09 (0.00, 0.28)
 
 
11.10
 
 
0.0003
 
 
0.0092
 
 
0.66 (0.34, 0.97)
 
 
TRUE
 
 
 
 
 OCHL 
 
 
oral
 
 
ratio
 
 
0.35 (0.28, 0.42)
 
 
ACE
 
 
0.17 (0.00, 0.24)
 
 
10.23
 
 
0.0005
 
 
0.0141
 
 
0.46 (0.23, 0.69)
 
 
TRUE
 
 
 
 
 HARF 
 
 
hard palate
 
 
angle
 
 
0.16 (0.08, 0.23)
 
 
ACE
 
 
0.02 (0.00, 0.26)
 
 
10.08
 
 
0.0005
 
 
0.0148
 
 
0.89 (0.50, 1.00)
 
 
TRUE
 
 
 
 
 HACL 
 
 
hard palate
 
 
distance
 
 
0.26 (0.19, 0.33)
 
 
ACE
 
 
0.08 (0.00, 0.25)
 
 
9.79
 
 
0.0006
 
 
0.0167
 
 
0.65 (0.36, 0.93)
 
 
TRUE
 
 
 
 
 HICL* 
 
 
hard palate
 
 
distance
 
 
0.39 (0.32, 0.45)
 
 
ACE
 
 
0.14 (0.00, 0.22)
 
 
9.79
 
 
0.0006
 
 
0.0167
 
 
0.42 (0.20, 0.64)
 
 
TRUE
 
 
 
 
 HNAP 
 
 
hard palate
 
 
distance
 
 
0.37 (0.30, 0.44)
 
 
ACE
 
 
0.16 (0.00, 0.21)
 
 
8.53
 
 
0.0012
 
 
0.0306
 
 
0.36 (0.15, 0.57)
 
 
TRUE
 
 
 
 
 HICL 
 
 
hard palate
 
 
distance
 
 
0.40 (0.33, 0.46)
 
 
ADE
 
 
0.03 (0.00, 0.20)
 
 
7.90
 
 
0.0017
 
 
0.0413
 
 
0.31 (0.12, 0.50)
 
 
TRUE
 
 
 
 
 CS3H 
 
 
cervical
 
 
distance
 
 
0.60 (0.54, 0.64)
 
 
ACE
 
 
0.22 (0.00, 0.13)
 
 
6.77
 
 
0.0031
 
 
0.0735
 
 
0.20 (0.07, 0.34)
 
 
TRUE
 
 
 
 
 HCCC 
 
 
hard palate
 
 
curvature
 
 
0.31 (0.24, 0.38)
 
 
ACE
 
 
0.15 (0.00, 0.20)
 
 
5.96
 
 
0.0048
 
 
0.1102
 
 
0.43 (0.15, 0.69)
 
 
TRUE
 
 
 
 
 SNNA 
 
 
skull
 
 
distance
 
 
0.66 (0.61, 0.70)
 
 
ACE
 
 
0.24 (0.00, 0.10)
 
 
5.77
 
 
0.0053
 
 
0.1171
 
 
0.13 (0.04, 0.24)
 
 
TRUE
 
 
 
 
 PPW3 
 
 
pharynx
 
 
curvature
 
 
0.19 (0.12, 0.27)
 
 
ADE
 
 
0.07 (0.00, 0.22)
 
 
5.02
 
 
0.0081
 
 
0.1618
 
 
0.64 (0.25, 1.00)
 
 
TRUE
 
 
 
 
 PPW6 
 
 
pharynx
 
 
curvature
 
 
0.43 (0.37, 0.50)
 
 
ACE
 
 
0.05 (0.00, 0.16)
 
 
5.10
 
 
0.0077
 
 
0.1618
 
 
0.28 (0.08, 0.49)
 
 
TRUE
 
 
 
 
 HMDC 
 
 
hard palate
 
 
curvature
 
 
0.21 (0.13, 0.28)
 
 
ACE
 
 
0.11 (0.00, 0.21)
 
 
4.47
 
 
0.0110
 
 
0.2089
 
 
0.51 (0.16, 0.83)
 
 
TRUE
 
 
 
 
 HCMP 
 
 
hard palate
 
 
Procrustes dist.
 
 
0.23 (0.15, 0.30)
 
 
ADE
 
 
0.13 (0.00, 0.22)
 
 
4.45
 
 
0.0111
 
 
0.2089
 
 
0.47 (0.13, 0.79)
 
 
TRUE
 
 
 
 
 PPWC 
 
 
pharynx
 
 
curvature
 
 
0.27 (0.19, 0.34)
 
 
ACE
 
 
0.06 (0.00, 0.19)
 
 
4.29
 
 
0.0122
 
 
0.2089
 
 
0.46 (0.12, 0.75)
 
 
TRUE
 
 
 
 
 DMAC 
 
 
dentition
 
 
curvature
 
 
0.21 (0.13, 0.28)
 
 
ACE
 
 
0.08 (0.00, 0.21)
 
 
3.94
 
 
0.0148
 
 
0.2369
 
 
0.57 (0.17, 0.91)
 
 
TRUE
 
 
 
 
 CS2H 
 
 
cervical
 
 
distance
 
 
0.59 (0.53, 0.64)
 
 
ADE
 
 
0.41 (0.00, 0.11)
 
 
3.65
 
 
0.0175
 
 
0.2622
 
 
0.12 (0.05, 0.24)
 
 
TRUE
 
 
 
 
 HM2C 
 
 
hard palate
 
 
curvature
 
 
0.23 (0.15, 0.30)
 
 
ADE
 
 
0.06 (0.00, 0.20)
 
 
2.85
 
 
0.0276
 
 
0.3868
 
 
0.48 (0.07, 0.84)
 
 
TRUE
 
 
 
 
 HMAC 
 
 
hard palate
 
 
curvature
 
 
0.08 (0.01, 0.16)
 
 
ADE
 
 
0.04 (0.00, 0.17)
 
 
-0.51
 
 
0.2226
 
 
1.0000
 
 
0.63 (0.00, 1.00)
 
 
TRUE
 
 
 
 
 DDAP 
 
 
dentition
 
 
Procrustes dist.
 
 
0.24 (0.17, 0.32)
 
 
ADE
 
 
0.16 (0.00, 0.16)
 
 
0.94
 
 
0.0867
 
 
1.0000
 
 
0.31 (0.00, 0.61)
 
 
TRUE
 
 
 
 
 DIIL 
 
 
dentition
 
 
distance
 
 
0.23 (0.15, 0.30)
 
 
ADE
 
 
0.03 (0.00, 0.16)
 
 
0.38
 
 
0.1231
 
 
1.0000
 
 
0.31 (0.00, 0.65)
 
 
TRUE
 
 
 
 
 PPW2 
 
 
pharynx
 
 
curvature
 
 
0.09 (0.01, 0.17)
 
 
ADE
 
 
0.07 (0.00, 0.13)
 
 
-1.68
 
 
0.5730
 
 
1.0000
 
 
0.27 (0.00, 1.00)
 
 
TRUE
 
 
 
 
 HMSP 
 
 
hard palate
 
 
Procrustes dist.
 
 
0.11 (0.03, 0.19)
 
 
ACE
 
 
0.08 (0.00, 0.11)
 
 
-1.86
 
 
0.7128
 
 
1.0000
 
 
0.17 (0.00, 0.79)
 
 
TRUE
 
 
 
 
 GRPD 
 
 
general
 
 
Procrustes dist.
 
 
0.30 (0.22, 0.36)
 
 
ADE
 
 
0.19 (0.00, 0.12)
 
 
-0.98
 
 
0.3132
 
 
1.0000
 
 
0.17 (0.00, 0.46)
 
 
FALSE
 
 
 
 
 MMPG 
 
 
mandible
 
 
distance
 
 
0.32 (0.24, 0.39)
 
 
ADE
 
 
0.24 (0.00, 0.13)
 
 
-0.61
 
 
0.2378
 
 
1.0000
 
 
0.16 (0.00, 0.40)
 
 
TRUE
 
 
 
 
 HCCP 
 
 
hard palate
 
 
Procrustes dist.
 
 
0.11 (0.03, 0.19)
 
 
ACE
 
 
0.09 (0.00, 0.11)
 
 
-1.93
 
 
0.7959
 
 
1.0000
 
 
0.12 (0.00, 0.67)
 
 
FALSE
 
 
 
 
 HMTC 
 
 
hard palate
 
 
distance
 
 
0.07 (-0.01, 0.15)
 
 
ACE
 
 
0.08 (0.00, 0.10)
 
 
-1.96
 
 
0.8350
 
 
1.0000
 
 
0.11 (0.00, 0.82)
 
 
FALSE
 
 
 
 
 SIOD 
 
 
skull
 
 
distance
 
 
0.71 (0.67, 0.74)
 
 
ADE
 
 
0.17 (0.00, 0.05)
 
 
-0.99
 
 
0.3149
 
 
1.0000
 
 
0.04 (0.00, 0.11)
 
 
TRUE
 
 
 
 
 SNOR 
 
 
skull
 
 
distance
 
 
0.53 (0.47, 0.58)
 
 
ADE
 
 
0.03 (0.00, 0.05)
 
 
-2.00
 
 
1.0000
 
 
1.0000
 
 
0.00 (0.00, 0.13)
 
 
FALSE
 
 
 
 
 SNOL 
 
 
skull
 
 
distance
 
 
0.54 (0.48, 0.59)
 
 
ACE
 
 
0.11 (0.00, 0.04)
 
 
-2.00
 
 
1.0000
 
 
1.0000
 
 
0.00 (0.00, 0.11)
 
 
FALSE
 
 
 
 
 CS2A 
 
 
cervical
 
 
distance
 
 
0.60 (0.55, 0.65)
 
 
ACE
 
 
0.05 (0.00, 0.03)
 
 
-2.00
 
 
1.0000
 
 
1.0000
 
 
0.00 (0.00, 0.08)
 
 
FALSE
 
 
 
 
 
 
 
 
 
 
 Estimates of interest 
 Here we focus on the estimates of interest from a genetic point of
view, namely the  narrow-sense heritability 
 h  2 , the  non-shared environment 
 e  2 , and the  shared environment 
 c  2  and  familiality   F  2 
(for ACE) or the  dominance   d  2  and
 broad-sense heritability   H  2  (for ADE), also
taking the inter-rater agreement into account. When it comes to
statistical significance, we used the following: 
 
 for the “simple” estimates  h  2 ,
 c  2 ,  d  2  and
 e  2 , we use the corresponding  p -values
derived from model comparison because they are more conservative and
allow multiple testing corrections, 
 however, the familiality  familiality   F  2 
and the  broad-sense heritability   H  2  are
composed from two “atomic” components ( A  and  C , and
 A  and  D , respectively), which makes model comparison
difficult; in these cases, we check if 0 is included in the 95%CI of the
composed estimate (but this method might be too liberal). 
 
 So, we do not include here the non-standardized components
 A ,  C  or  D , and  E  (please see above
for the results concerning them). 
 Moreover, we exclude here the 15 measures with warning flag values ≥
5. 
 
 Relationships between these estimates 
 
 
  Table 21.    Correlations between the
estimates of interest, including a Bonferroni correction and
significance stars. 
 
 
 
 
measure1
 
 
measure2
 
 
Pearson’s  r  ( p ; adjusted  p )
 
 
Spearman’s  ρ  ( p ; adjusted  p )
 
 
 
 
 
 
h2
 
 
c2
 
 
-0.75 (7.76e-17; 6.99e-16***)
 
 
-0.75 (0; 0***)
 
 
 
 
h2
 
 
d2
 
 
-0.80 (3.28e-11; 2.95e-10***)
 
 
-0.80 (0; 0***)
 
 
 
 
h2
 
 
e2
 
 
-0.54 (2.05e-11; 1.85e-10***)
 
 
-0.50 (1.84e-09; 1.65e-08***)
 
 
 
 
c2
 
 
e2
 
 
0.06 (0.599; 1 )
 
 
0.11 (0.32; 1 )
 
 
 
 
d2
 
 
e2
 
 
-0.31 (0.0339; 0.305 )
 
 
-0.34 (0.0195; 0.176 )
 
 
 
 
ICC
 
 
h2
 
 
0.42 (7.07e-07; 6.36e-06***)
 
 
0.42 (8.76e-07; 7.89e-06***)
 
 
 
 
ICC
 
 
c2
 
 
-0.16 (0.152; 1 )
 
 
-0.17 (0.128; 1 )
 
 
 
 
ICC
 
 
d2
 
 
0.20 (0.174; 1 )
 
 
0.22 (0.14; 1 )
 
 
 
 
ICC
 
 
e2
 
 
-0.64 (1.72e-16; 1.55e-15***)
 
 
-0.65 (0; 0***)
 
 
 
 
 
 
 
  Figure 40.    Pairwise correlations between
the estimates of interest. 
 
 
 It is important to note the following: 
 
 narrow-sense heritability  h  2  is very strongly
and negatively correlated with the shared environment
( c  2 ) and the dominance ( d  2 )
effects, and less strongly and negatively with the error
( e  2 ); 
  c  2  and  d  2  are not correlated
with  e  2 ; 
 the inter-rater agreement ( ICC ( C ,1)) is strongly
and positively correlated with  h  2 , strongly and
negatively with  e  2 , but not with
 d  2  and  c  2 , suggesting that
measures where the raters disagree might result in artificially lowered
heritability estimates as this disagreement goes into the error
term. 
 
 
 
 By domain and type 
 
 
 
  Figure 41.    By row, from top to bottom the
estimates of interest (excluding
 H  2 / F  2 ). Left column: by domain;
right column: by type. Dashed black lines represent the important values
of 0.0, 0.2, and 1.0. 
 
 
 
  h  2  
 ANOVA by domain and type (N.B. strictly speaking, we should use a
 Beta  distribution, but for our purposes here a linear model
should suffice): 
 
 by  domain : 
 
 
 Analysis of Variance Model 
 
 
 
 
 
 
 
 
 
 
   
 Df 
 Sum Sq 
 Mean Sq 
 F value 
 Pr(>F) 
 
 
 
 
  domain  
 10 
 3.099 
 0.3099 
 6.641 
 3.851e-08 
 
 
  Residuals  
 120 
 5.6 
 0.04667 
 NA 
 NA 
 
 
 
 
 by  type : 
 
 
 Analysis of Variance Model 
 
 
 
 
 
 
 
 
 
 
   
 Df 
 Sum Sq 
 Mean Sq 
 F value 
 Pr(>F) 
 
 
 
 
  type  
 4 
 0.556 
 0.139 
 2.151 
 0.07839 
 
 
  Residuals  
 126 
 8.143 
 0.06463 
 NA 
 NA 
 
 
 
 The pairwise significant differences after Tukey’s HSD: 
 
 by  domain  there are 6 significantly different
pairs:
 
 
  Table 22.    Pairwise significant comparisons
(Tukey’s HSD) 
 
 
 
 
 
 
diff
 
 
lwr
 
 
upr
 
 
p.adj
 
 
 
 
 
 
skull-general
 
 
0.40
 
 
0.02
 
 
0.78
 
 
0.0292
 
 
 
 
skull-hard palate
 
 
0.40
 
 
0.20
 
 
0.59
 
 
4.62e-08
 
 
 
 
skull-pharynx
 
 
0.40
 
 
0.05
 
 
0.74
 
 
0.0112
 
 
 
 
skull-dentition
 
 
0.37
 
 
0.10
 
 
0.63
 
 
0.000549
 
 
 
 
skull-larynx
 
 
0.35
 
 
0.14
 
 
0.55
 
 
1.37e-05
 
 
 
 
skull-oral
 
 
0.33
 
 
0.08
 
 
0.57
 
 
0.00117
 
 
 
  
 by  type  there are 0 significantly different
pairs: 
 
 
 
  c  2  
 
 by  domain : 
 
 
 Analysis of Variance Model 
 
 
 
 
 
 
 
 
 
 
   
 Df 
 Sum Sq 
 Mean Sq 
 F value 
 Pr(>F) 
 
 
 
 
  domain  
 10 
 0.9451 
 0.09451 
 3.985 
 0.0002225 
 
 
  Residuals  
 74 
 1.755 
 0.02371 
 NA 
 NA 
 
 
 
 
 by  type : 
 
 
 Analysis of Variance Model 
 
 
 
 
 
 
 
 
 
 
   
 Df 
 Sum Sq 
 Mean Sq 
 F value 
 Pr(>F) 
 
 
 
 
  type  
 4 
 0.3596 
 0.08991 
 3.074 
 0.02079 
 
 
  Residuals  
 80 
 2.34 
 0.02925 
 NA 
 NA 
 
 
 
 The pairwise significant differences after Tukey’s HSD: 
 
 by  domain  there are 4 significantly different
pairs:
 
 
  Table 23.    Pairwise significant comparisons
(Tukey’s HSD) 
 
 
 
 
 
 
diff
 
 
lwr
 
 
upr
 
 
p.adj
 
 
 
 
 
 
mandible-general
 
 
-0.44
 
 
-0.76
 
 
-0.12
 
 
0.000823
 
 
 
 
hyoid-general
 
 
-0.37
 
 
-0.71
 
 
-0.03
 
 
0.0236
 
 
 
 
skull-general
 
 
-0.33
 
 
-0.61
 
 
-0.04
 
 
0.0117
 
 
 
 
mandible-hard palate
 
 
-0.29
 
 
-0.52
 
 
-0.06
 
 
0.00388
 
 
 
  
 by  type  there are 0 significantly different
pairs: 
 
 
 
  d  2  
 
 by  domain : 
 
 
 Analysis of Variance Model 
 
 
 
 
 
 
 
 
 
 
   
 Df 
 Sum Sq 
 Mean Sq 
 F value 
 Pr(>F) 
 
 
 
 
  domain  
 7 
 1.327 
 0.1896 
 3.79 
 0.003267 
 
 
  Residuals  
 38 
 1.901 
 0.05002 
 NA 
 NA 
 
 
 
 
 by  type : 
 
 
 Analysis of Variance Model 
 
 
 
 
 
 
 
 
 
 
   
 Df 
 Sum Sq 
 Mean Sq 
 F value 
 Pr(>F) 
 
 
 
 
  type  
 3 
 0.1978 
 0.06595 
 0.9141 
 0.4424 
 
 
  Residuals  
 42 
 3.03 
 0.07215 
 NA 
 NA 
 
 
 
 The pairwise significant differences after Tukey’s HSD: 
 
 by  domain  there are 3 significantly different
pairs:
 
 
  Table 24.    Pairwise significant comparisons
(Tukey’s HSD) 
 
 
 
 
 
 
diff
 
 
lwr
 
 
upr
 
 
p.adj
 
 
 
 
 
 
oral-mandible
 
 
-0.59
 
 
-1.17
 
 
-0.02
 
 
0.0386
 
 
 
 
mandible-larynx
 
 
0.44
 
 
0.07
 
 
0.81
 
 
0.0105
 
 
 
 
skull-mandible
 
 
-0.42
 
 
-0.77
 
 
-0.08
 
 
0.00635
 
 
 
  
 by  type  there are 0 significantly different
pairs: 
 
 
 
  e  2  
 
 by  domain : 
 
 
 Analysis of Variance Model 
 
 
 
 
 
 
 
 
 
 
   
 Df 
 Sum Sq 
 Mean Sq 
 F value 
 Pr(>F) 
 
 
 
 
  domain  
 10 
 2.091 
 0.2091 
 16.62 
 1.696e-18 
 
 
  Residuals  
 120 
 1.509 
 0.01258 
 NA 
 NA 
 
 
 
 
 by  type : 
 
 
 Analysis of Variance Model 
 
 
 
 
 
 
 
 
 
 
   
 Df 
 Sum Sq 
 Mean Sq 
 F value 
 Pr(>F) 
 
 
 
 
  type  
 4 
 0.6488 
 0.1622 
 6.926 
 4.512e-05 
 
 
  Residuals  
 126 
 2.951 
 0.02342 
 NA 
 NA 
 
 
 
 The pairwise significant differences after Tukey’s HSD: 
 
 by  domain  there are 12 significantly different
pairs:
 
 
  Table 25.    Pairwise significant comparisons
(Tukey’s HSD) 
 
 
 
 
 
 
diff
 
 
lwr
 
 
upr
 
 
p.adj
 
 
 
 
 
 
skull-oral
 
 
-0.34
 
 
-0.47
 
 
-0.22
 
 
5.31e-13
 
 
 
 
oral-mandible
 
 
0.34
 
 
0.19
 
 
0.48
 
 
3.8e-10
 
 
 
 
skull-larynx
 
 
-0.28
 
 
-0.39
 
 
-0.18
 
 
1.55e-12
 
 
 
 
mandible-larynx
 
 
-0.28
 
 
-0.40
 
 
-0.15
 
 
6.4e-09
 
 
 
 
skull-hard palate
 
 
-0.26
 
 
-0.36
 
 
-0.16
 
 
2.95e-12
 
 
 
 
oral-cervical
 
 
0.26
 
 
0.08
 
 
0.43
 
 
0.000184
 
 
 
 
mandible-hard palate
 
 
-0.25
 
 
-0.38
 
 
-0.13
 
 
2.28e-08
 
 
 
 
oral-general
 
 
0.24
 
 
0.03
 
 
0.45
 
 
0.0123
 
 
 
 
skull-dentition
 
 
-0.20
 
 
-0.34
 
 
-0.06
 
 
0.000197
 
 
 
 
larynx-cervical
 
 
0.20
 
 
0.04
 
 
0.36
 
 
0.00422
 
 
 
 
mandible-dentition
 
 
-0.19
 
 
-0.34
 
 
-0.04
 
 
0.0032
 
 
 
 
hard palate-cervical
 
 
0.18
 
 
0.02
 
 
0.34
 
 
0.0131
 
 
 
  
 by  type  there are 3 significantly different pairs:
 
 
  Table 26.    Pairwise significant comparisons
(Tukey’s HSD) 
 
 
 
 
 
 
diff
 
 
lwr
 
 
upr
 
 
p.adj
 
 
 
 
 
 
ratio-distance
 
 
0.21
 
 
0.08
 
 
0.34
 
 
0.000142
 
 
 
 
ratio-angle
 
 
0.21
 
 
0.06
 
 
0.37
 
 
0.0023
 
 
 
 
distance-curvature
 
 
-0.16
 
 
-0.32
 
 
-0.01
 
 
0.0373
 
 
 
  
 
 
 
 
 Combined results 
 While our GSEM does model the inter-rater agreement (as
 agr (GSEM)), it cannot do so perfectly, in the sense that a low
inter-rater agreement fundamentally induces noise in the estimates
(moreover, the residual correlation between  ICC ( C ,1)
and the GSEM estimates strongly suggests that this statistical control
is not perfect). Therefore, we decided to weight the interpretation of
the GSEM estimates by the generic inter-rater agreement
 ICC ( C ,1). 
 With these, we have several ways of ranking the PMs (not necessarily
independent), given below with the notation we’ll use throughout: 
 
 is the GSEM estimate statistically significantly greater than 0?
 
 is this  nominally  so at  α -level 0.05? (denoted as
“ ★ ”) 
 if so, does this survive  Holm’s multiple testing correction 
at the same  α -level 0.05? (denoted as “ ★c ”) 
  
 is the GSEM estimate greater than a given threshold (here, 20%)?
 
 is the  point estimate  grater than this threshold? (denoted
as “ > ”) 
 is the  lower limit of the 95%CI  grater than this threshold?
(denoted as “ ≫ ”) 
  
 is the inter-rater agreement greater than a given threshold (here,
75%)?
 
 is the  point estimate  grater than this threshold? (denoted
as “ + ”) 
 is the  lower limit of the 95%CI  grater than this threshold?
(denoted as “ ++ ”) 
  
 
 Please note that there are logical relationships between some of
these criteria: 
 
 “ ★c ” ⇒ “ ★ ” 
 “ ≫ ” ⇒ “ > ” 
 “ ++ ” ⇒ “ + ” 
 
 For each component ( h  2 ,  c  2  or
 d  2 , and  e  2 ), we combine these
criteria to obtain a  ranking  of the measures from
 0  (= best) to a maximum (= worst), which we then
combine into  classes  from  I  (= strongest
evidence) to  V  (= basically no evidence
whatsoever). 
 
 Narrow-sense heritability,  h  2  
 Because there is a large positive correlation between
 h  2  and the inter-rater agreement
 ICC ( C ,1) (Pearson’s  r =0.42,
 p =7.07e-07; Spearman’s  ρ =0.42,  p =8.76e-07),
we also consider this agreement in our ranking of the measures. 
 
 
  Table 27.    Ranking of measures ordered
decreassingly by the strength of evidence they provide for high
narrow-sense heritability  h  2 , with the number of
measures in each ranking (see below for the defintion of classes). 
 
 
 
 
ranking
 
 
class
 
 
count
 
 
 h  2★c 
 
 
 h  2★ 
 
 
 h  2≫ 
 
 
 h  2> 
 
 
ICC ++ 
 
 
ICC + 
 
 
 
 
 
 
 0 
 
 
I
 
 
13
 
 
Yes
 
 
Yes
 
 
Yes
 
 
Yes
 
 
Yes
 
 
Yes
 
 
 
 
 1 
 
 
I
 
 
1
 
 
Yes
 
 
Yes
 
 
No
 
 
Yes
 
 
Yes
 
 
Yes
 
 
 
 
 2 
 
 
II
 
 
1
 
 
No
 
 
Yes
 
 
Yes
 
 
Yes
 
 
Yes
 
 
Yes
 
 
 
 
 3 
 
 
III
 
 
3
 
 
No
 
 
Yes
 
 
Yes
 
 
Yes
 
 
No
 
 
No
 
 
 
 
 4 
 
 
II
 
 
4
 
 
No
 
 
Yes
 
 
No
 
 
Yes
 
 
Yes
 
 
Yes
 
 
 
 
 5 
 
 
III
 
 
3
 
 
No
 
 
Yes
 
 
No
 
 
Yes
 
 
No
 
 
No
 
 
 
 
 6 
 
 
IV
 
 
11
 
 
No
 
 
No
 
 
No
 
 
Yes
 
 
Yes
 
 
Yes
 
 
 
 
 7 
 
 
IV
 
 
5
 
 
No
 
 
No
 
 
No
 
 
Yes
 
 
No
 
 
Yes
 
 
 
 
 8 
 
 
V
 
 
43
 
 
No
 
 
No
 
 
No
 
 
Yes
 
 
No
 
 
No
 
 
 
 
 9 
 
 
V
 
 
6
 
 
No
 
 
No
 
 
No
 
 
No
 
 
Yes
 
 
Yes
 
 
 
 
 10 
 
 
V
 
 
2
 
 
No
 
 
No
 
 
No
 
 
No
 
 
No
 
 
Yes
 
 
 
 
 11 
 
 
V
 
 
39
 
 
No
 
 
No
 
 
No
 
 
No
 
 
No
 
 
No
 
 
 
 
 Ranking  0  gives the strongest evidence of high
heritability of a reliably-measured phenotype, followed by ranking
 1  → class  I . 
 Rankings  2  and  4  give evidence of
relatively high heritability, coupled with good inter-rater agreement →
class  II . 
 Rankings  3  and  5  give evidence of
relatively high heritability, but lack this inter-rater agreement→ class
 III . 
 Rankings  6  and  7  give some
circumstantial evidence of heritability, coupled or not with inter-rater
agreement → class  IV . 
 Rankings  8 ,  9 ,  10 
and  11  basically give no evidence of heritability →
class  V . 
 
 
  Table 28.    The 41 measures with at least
some evidence of heritability (class < V) ordered by class and
 h  2 , also showing the nominal  p -value (and
the Holm-corrected  p -value). The precise meaning of
 c  2  or  d  2  is disambiguated by the
genetic model. 
 
 
 
 
measure
 
 
domain
 
 
type
 
 
genetic model
 
 
ranking
 
 
class
 
 
 h  2 
 
 
 p 
 
 
 c  2  or  d  2 
 
 
 e  2 
 
 
ICC(C,1)
 
 
r MZ 
 
 
r DZ 
 
 
 
 
 
 
 MICD 
 
 
mandible
 
 
distance
 
 
ACE
 
 
0
 
 
I
 
 
0.87 (0.51, 0.90)
 
 
2.58e-13 (3.76e-11)
 
 
0.01 (0.00, 0.36)
 
 
0.13 (0.10, 0.16)
 
 
0.98 (0.98, 0.99)
 
 
0.92
 
 
0.19
 
 
 
 
 SNAP 
 
 
skull
 
 
distance
 
 
ACE
 
 
0
 
 
I
 
 
0.79 (0.40, 0.88)
 
 
9.46e-07 (0.000133)
 
 
0.05 (0.00, 0.43)
 
 
0.16 (0.12, 0.22)
 
 
0.88 (0.86, 0.90)
 
 
0.79
 
 
0.38
 
 
 
 
 SNAM 
 
 
skull
 
 
distance
 
 
ACE
 
 
0
 
 
I
 
 
0.78 (0.40, 0.88)
 
 
7.92e-07 (0.000112)
 
 
0.06 (0.00, 0.43)
 
 
0.17 (0.12, 0.22)
 
 
0.90 (0.89, 0.92)
 
 
0.81
 
 
0.35
 
 
 
 
 SSEG 
 
 
skull
 
 
distance
 
 
ACE
 
 
0
 
 
I
 
 
0.77 (0.38, 0.83)
 
 
5.21e-06 (0.000724)
 
 
0.01 (0.00, 0.39)
 
 
0.22 (0.17, 0.29)
 
 
0.90 (0.88, 0.91)
 
 
0.72
 
 
0.38
 
 
 
 
 MCGP 
 
 
mandible
 
 
angle
 
 
ACE
 
 
0
 
 
I
 
 
0.74 (0.38, 0.86)
 
 
3.07e-06 (0.000429)
 
 
0.07 (0.00, 0.44)
 
 
0.18 (0.14, 0.24)
 
 
0.87 (0.84, 0.88)
 
 
0.73
 
 
0.44
 
 
 
 
 MIGD 
 
 
mandible
 
 
distance
 
 
ACE
 
 
0
 
 
I
 
 
0.69 (0.39, 0.91)
 
 
8.41e-09 (1.21e-06)
 
 
0.19 (0.00, 0.49)
 
 
0.12 (0.09, 0.17)
 
 
0.95 (0.94, 0.95)
 
 
0.91
 
 
0.41
 
 
 
 
 MCGM 
 
 
mandible
 
 
angle
 
 
ACE
 
 
0
 
 
I
 
 
0.67 (0.43, 0.85)
 
 
1.74e-05 (0.00238)
 
 
0.14 (0.00, 0.48)
 
 
0.20 (0.15, 0.26)
 
 
0.89 (0.87, 0.90)
 
 
0.73
 
 
0.45
 
 
 
 
 ASCG* 
 
 
skull
 
 
angle
 
 
ACE
 
 
0
 
 
I
 
 
0.67 (0.41, 0.85)
 
 
1.74e-05 (0.00238)
 
 
0.14 (0.00, 0.48)
 
 
0.20 (0.15, 0.26)
 
 
0.87 (0.85, 0.89)
 
 
0.73
 
 
0.45
 
 
 
 
 SBNA 
 
 
skull
 
 
distance
 
 
ACE
 
 
0
 
 
I
 
 
0.67 (0.32, 0.87)
 
 
5.76e-06 (0.000794)
 
 
0.17 (0.00, 0.51)
 
 
0.17 (0.12, 0.22)
 
 
0.90 (0.88, 0.91)
 
 
0.79
 
 
0.37
 
 
 
 
 SBAN 
 
 
skull
 
 
distance
 
 
ACE
 
 
0
 
 
I
 
 
0.66 (0.36, 0.92)
 
 
1.69e-07 (2.41e-05)
 
 
0.22 (0.00, 0.52)
 
 
0.11 (0.08, 0.16)
 
 
0.89 (0.88, 0.91)
 
 
0.83
 
 
0.44
 
 
 
 
 SHSW 
 
 
skull
 
 
distance
 
 
ACE
 
 
0
 
 
I
 
 
0.63 (0.28, 0.86)
 
 
3.15e-05 (0.00422)
 
 
0.19 (0.00, 0.53)
 
 
0.18 (0.14, 0.24)
 
 
0.94 (0.93, 0.95)
 
 
0.88
 
 
0.21
 
 
 
 
 SNNP 
 
 
skull
 
 
distance
 
 
ACE
 
 
0
 
 
I
 
 
0.62 (0.28, 0.95)
 
 
2.2e-05 (0.00297)
 
 
0.29 (0.00, 0.63)
 
 
0.08 (0.04, 0.13)
 
 
0.82 (0.80, 0.85)
 
 
0.80
 
 
0.46
 
 
 
 
 SHBN 
 
 
skull
 
 
distance
 
 
ACE
 
 
0
 
 
I
 
 
0.56 (0.31, 0.91)
 
 
1.99e-09 (2.88e-07)
 
 
0.34 (0.00, 0.58)
 
 
0.10 (0.08, 0.13)
 
 
0.96 (0.95, 0.96)
 
 
0.89
 
 
0.52
 
 
 
 
 SSEN 
 
 
skull
 
 
distance
 
 
ACE
 
 
1
 
 
I
 
 
0.47 (0.19, 0.87)
 
 
9.46e-05 (0.0126)
 
 
0.40 (0.00, 0.67)
 
 
0.13 (0.09, 0.17)
 
 
0.90 (0.89, 0.92)
 
 
0.83
 
 
0.53
 
 
 
 
 APNS 
 
 
skull
 
 
angle
 
 
ACE
 
 
2
 
 
II
 
 
0.73 (0.25, 0.81)
 
 
0.000716 (0.0934)
 
 
0.02 (0.00, 0.48)
 
 
0.25 (0.19, 0.33)
 
 
0.83 (0.80, 0.85)
 
 
0.65
 
 
0.30
 
 
 
 
 HBNP 
 
 
hyoid
 
 
distance
 
 
ACE
 
 
4
 
 
II
 
 
0.57 (0.11, 0.78)
 
 
0.0128 (1)
 
 
0.13 (0.00, 0.56)
 
 
0.30 (0.22, 0.40)
 
 
0.84 (0.81, 0.86)
 
 
0.70
 
 
0.29
 
 
 
 
 HBC4 
 
 
hyoid
 
 
distance
 
 
ACE
 
 
4
 
 
II
 
 
0.52 (0.04, 0.72)
 
 
0.0319 (1)
 
 
0.12 (0.00, 0.57)
 
 
0.36 (0.28, 0.46)
 
 
0.79 (0.76, 0.82)
 
 
0.52
 
 
0.32
 
 
 
 
 HBPL 
 
 
hard palate
 
 
distance
 
 
ACE
 
 
4
 
 
II
 
 
0.38 (0.12, 0.78)
 
 
0.00208 (0.267)
 
 
0.45 (0.08, 0.71)
 
 
0.16 (0.12, 0.21)
 
 
0.91 (0.89, 0.92)
 
 
0.80
 
 
0.48
 
 
 
 
 SVTh* 
 
 
general
 
 
distance
 
 
ACE
 
 
4
 
 
II
 
 
0.26 (0.03, 0.62)
 
 
0.0278 (1)
 
 
0.55 (0.20, 0.77)
 
 
0.19 (0.15, 0.24)
 
 
0.93 (0.91, 0.94)
 
 
0.78
 
 
0.55
 
 
 
 
 CS2A 
 
 
cervical
 
 
distance
 
 
ACE
 
 
3
 
 
III
 
 
0.88 (0.36, 1.00)
 
 
0.000713 (0.0934)
 
 
0.12 (0.00, 0.66)
 
 
0.00 (0.00, 0.08)
 
 
0.60 (0.55, 0.65)
 
 
0.70
 
 
0.24
 
 
 
 
 MCGD 
 
 
mandible
 
 
distance
 
 
ACE
 
 
3
 
 
III
 
 
0.80 (0.31, 0.91)
 
 
0.000737 (0.0951)
 
 
0.02 (0.00, 0.47)
 
 
0.18 (0.09, 0.29)
 
 
0.71 (0.67, 0.75)
 
 
0.66
 
 
0.36
 
 
 
 
 ASNP 
 
 
skull
 
 
angle
 
 
ACE
 
 
3
 
 
III
 
 
0.73 (0.26, 0.91)
 
 
0.000667 (0.0881)
 
 
0.12 (0.00, 0.58)
 
 
0.16 (0.09, 0.23)
 
 
0.72 (0.68, 0.76)
 
 
0.63
 
 
0.30
 
 
 
 
 SNOL 
 
 
skull
 
 
distance
 
 
ACE
 
 
5
 
 
III
 
 
0.69 (0.09, 1.00)
 
 
0.0226 (1)
 
 
0.31 (0.00, 0.90)
 
 
0.00 (0.00, 0.11)
 
 
0.54 (0.48, 0.59)
 
 
0.60
 
 
0.29
 
 
 
 
 AASN 
 
 
skull
 
 
angle
 
 
ACE
 
 
5
 
 
III
 
 
0.66 (0.17, 0.92)
 
 
0.00705 (0.896)
 
 
0.18 (0.00, 0.65)
 
 
0.17 (0.08, 0.26)
 
 
0.63 (0.58, 0.67)
 
 
0.53
 
 
0.36
 
 
 
 
 MCGR 
 
 
mandible
 
 
distance
 
 
ACE
 
 
5
 
 
III
 
 
0.59 (0.05, 0.82)
 
 
0.0323 (1)
 
 
0.12 (0.00, 0.61)
 
 
0.29 (0.18, 0.42)
 
 
0.68 (0.64, 0.72)
 
 
0.57
 
 
0.28
 
 
 
 
 SBAS 
 
 
skull
 
 
distance
 
 
ADE
 
 
6
 
 
IV
 
 
0.78 (0.00, 0.86)
 
 
0.112 (1)
 
 
0.01 (0.00, 0.84)
 
 
0.20 (0.14, 0.28)
 
 
0.79 (0.76, 0.82)
 
 
0.66
 
 
0.36
 
 
 
 
 ANSF 
 
 
skull
 
 
angle
 
 
ADE
 
 
6
 
 
IV
 
 
0.73 (0.00, 0.84)
 
 
0.219 (1)
 
 
0.05 (0.00, 0.83)
 
 
0.22 (0.16, 0.30)
 
 
0.79 (0.75, 0.81)
 
 
0.64
 
 
0.33
 
 
 
 
 SBNP 
 
 
skull
 
 
distance
 
 
ADE
 
 
6
 
 
IV
 
 
0.65 (0.00, 0.86)
 
 
0.257 (1)
 
 
0.16 (0.00, 0.85)
 
 
0.20 (0.14, 0.27)
 
 
0.80 (0.77, 0.83)
 
 
0.70
 
 
0.24
 
 
 
 
 SSEN* 
 
 
skull
 
 
distance
 
 
ADE
 
 
6
 
 
IV
 
 
0.46 (0.00, 0.83)
 
 
0.393 (1)
 
 
0.32 (0.00, 0.83)
 
 
0.22 (0.16, 0.28)
 
 
0.90 (0.88, 0.91)
 
 
0.77
 
 
0.26
 
 
 
 
 HBPG 
 
 
hyoid
 
 
distance
 
 
ACE
 
 
6
 
 
IV
 
 
0.37 (0.00, 0.68)
 
 
0.147 (1)
 
 
0.22 (0.00, 0.62)
 
 
0.40 (0.31, 0.51)
 
 
0.81 (0.78, 0.83)
 
 
0.51
 
 
0.26
 
 
 
 
 ABSN 
 
 
skull
 
 
angle
 
 
ADE
 
 
6
 
 
IV
 
 
0.36 (0.00, 0.84)
 
 
0.495 (1)
 
 
0.42 (0.00, 0.84)
 
 
0.21 (0.16, 0.29)
 
 
0.82 (0.79, 0.84)
 
 
0.66
 
 
0.26
 
 
 
 
 DIPD 
 
 
dentition
 
 
distance
 
 
ADE
 
 
6
 
 
IV
 
 
0.33 (0.00, 0.72)
 
 
0.568 (1)
 
 
0.32 (0.00, 0.73)
 
 
0.35 (0.27, 0.46)
 
 
0.80 (0.77, 0.82)
 
 
0.55
 
 
0.23
 
 
 
 
 ACSN 
 
 
skull
 
 
angle
 
 
ADE
 
 
6
 
 
IV
 
 
0.30 (0.00, 0.84)
 
 
0.574 (1)
 
 
0.50 (0.00, 0.85)
 
 
0.20 (0.15, 0.26)
 
 
0.87 (0.85, 0.89)
 
 
0.71
 
 
0.25
 
 
 
 
 MCPD 
 
 
mandible
 
 
distance
 
 
ADE
 
 
6
 
 
IV
 
 
0.26 (0.00, 0.86)
 
 
0.615 (1)
 
 
0.57 (0.00, 0.87)
 
 
0.18 (0.13, 0.23)
 
 
0.92 (0.90, 0.93)
 
 
0.82
 
 
0.19
 
 
 
 
 MPGR 
 
 
mandible
 
 
distance
 
 
ADE
 
 
6
 
 
IV
 
 
0.24 (0.00, 0.88)
 
 
0.668 (1)
 
 
0.60 (0.00, 0.89)
 
 
0.16 (0.11, 0.23)
 
 
0.84 (0.81, 0.86)
 
 
0.76
 
 
0.12
 
 
 
 
 SVTv* 
 
 
general
 
 
distance
 
 
ACE
 
 
6
 
 
IV
 
 
0.24 (0.00, 0.74)
 
 
0.307 (1)
 
 
0.43 (0.00, 0.73)
 
 
0.32 (0.23, 0.43)
 
 
0.80 (0.77, 0.83)
 
 
0.68
 
 
0.32
 
 
 
 
 L4EA 
 
 
larynx
 
 
distance
 
 
ACE
 
 
7
 
 
IV
 
 
0.43 (0.00, 0.69)
 
 
0.0994 (1)
 
 
0.17 (0.00, 0.61)
 
 
0.40 (0.31, 0.51)
 
 
0.76 (0.72, 0.79)
 
 
0.47
 
 
0.28
 
 
 
 
 HBC2 
 
 
hyoid
 
 
distance
 
 
ACE
 
 
7
 
 
IV
 
 
0.35 (0.00, 0.73)
 
 
0.196 (1)
 
 
0.29 (0.00, 0.67)
 
 
0.37 (0.26, 0.49)
 
 
0.78 (0.74, 0.81)
 
 
0.64
 
 
0.28
 
 
 
 
 HNSL 
 
 
hard palate
 
 
distance
 
 
ACE
 
 
7
 
 
IV
 
 
0.31 (0.00, 0.78)
 
 
0.0848 (1)
 
 
0.49 (0.02, 0.80)
 
 
0.20 (0.14, 0.28)
 
 
0.77 (0.74, 0.80)
 
 
0.66
 
 
0.51
 
 
 
 
 DIMD 
 
 
dentition
 
 
distance
 
 
ACE
 
 
7
 
 
IV
 
 
0.29 (0.00, 0.75)
 
 
0.197 (1)
 
 
0.40 (0.00, 0.72)
 
 
0.32 (0.23, 0.42)
 
 
0.77 (0.73, 0.80)
 
 
0.58
 
 
0.41
 
 
 
 
 LAEA 
 
 
larynx
 
 
distance
 
 
ACE
 
 
7
 
 
IV
 
 
0.27 (0.00, 0.61)
 
 
0.394 (1)
 
 
0.23 (0.00, 0.58)
 
 
0.49 (0.38, 0.62)
 
 
0.77 (0.73, 0.80)
 
 
0.48
 
 
0.20
 
 
 
 
 
 
 Shared environment,  c  2  
 Because there is no correlation between  c  2  and
the inter-rater agreement  ICC ( C ,1) (Pearson’s
 r =-0.16,  p =0.152; Spearman’s  ρ =-0.17,
 p =0.128), we will not consider agreement in our ranking of the
measures. 
 
 
  Table 29.    Rankings of measures ordered
decreassingly by the strength of evidence they provide for high shared
environmental effects  c  2 , with the number of
measures in each ranking. 
 
 
 
 
ranking
 
 
class
 
 
count
 
 
 c  2★c 
 
 
 c  2★ 
 
 
 c  2≫ 
 
 
 c  2> 
 
 
 
 
 
 
 1 
 
 
II
 
 
3
 
 
No
 
 
Yes
 
 
Yes
 
 
Yes
 
 
 
 
 2 
 
 
II
 
 
6
 
 
No
 
 
Yes
 
 
No
 
 
Yes
 
 
 
 
 3 
 
 
IV
 
 
44
 
 
No
 
 
No
 
 
No
 
 
Yes
 
 
 
 
 4 
 
 
V
 
 
32
 
 
No
 
 
No
 
 
No
 
 
No
 
 
 
 
 There is no equivalent of  h  2 ’s ranking
 0 . 
 Rankings  1  and  2  give evidence of a
shared environmental effect → class  II . 
 Ranking  3  gives marginal evidence of a shared
environmental effect → class  IV . 
 Ranking  4  basically gives no evidence of a shared
environmental effect → class  V . 
 
 
  Table 30.    The 53 measures with at least
some evidence of shared environmental effects (class < V) ordered by
ranking and  c  2 , also showing the nominal
 p -value (and the Holm-corrected  p -value). 
 
 
 
 
measure
 
 
domain
 
 
type
 
 
genetic model
 
 
ranking
 
 
class
 
 
 h  2 
 
 
 c  2 
 
 
 p 
 
 
 e  2 
 
 
ICC(C,1)
 
 
r MZ 
 
 
r DZ 
 
 
 
 
 
 
 SVTh 
 
 
general
 
 
distance
 
 
ACE
 
 
1
 
 
II
 
 
0.17 (0.00, 0.53)
 
 
0.59 (0.22, 0.79)
 
 
0.00485 (0.703)
 
 
0.24 (0.18, 0.30)
 
 
0.90 (0.88, 0.91)
 
 
0.73
 
 
0.55
 
 
 
 
 SVTv 
 
 
general
 
 
distance
 
 
ACE
 
 
1
 
 
II
 
 
0.00 (0.00, 0.36)
 
 
0.62 (0.25, 0.72)
 
 
0.0066 (0.944)
 
 
0.38 (0.28, 0.50)
 
 
0.73 (0.70, 0.77)
 
 
0.57
 
 
0.40
 
 
 
 
 CS4H 
 
 
cervical
 
 
distance
 
 
ACE
 
 
1
 
 
II
 
 
0.00 (0.00, 0.32)
 
 
0.56 (0.40, 0.70)
 
 
0.00801 (1)
 
 
0.44 (0.30, 0.60)
 
 
0.59 (0.53, 0.64)
 
 
0.43
 
 
0.34
 
 
 
 
 SVTh* 
 
 
general
 
 
distance
 
 
ACE
 
 
2
 
 
II
 
 
0.26 (0.03, 0.62)
 
 
0.55 (0.20, 0.77)
 
 
0.00538 (0.774)
 
 
0.19 (0.15, 0.24)
 
 
0.93 (0.91, 0.94)
 
 
0.78
 
 
0.55
 
 
 
 
 HBPL 
 
 
hard palate
 
 
distance
 
 
ACE
 
 
2
 
 
II
 
 
0.38 (0.12, 0.78)
 
 
0.45 (0.08, 0.71)
 
 
0.0274 (1)
 
 
0.16 (0.12, 0.21)
 
 
0.91 (0.89, 0.92)
 
 
0.80
 
 
0.48
 
 
 
 
 SSEN 
 
 
skull
 
 
distance
 
 
ACE
 
 
2
 
 
II
 
 
0.47 (0.19, 0.87)
 
 
0.40 (0.00, 0.67)
 
 
0.0494 (1)
 
 
0.13 (0.09, 0.17)
 
 
0.90 (0.89, 0.92)
 
 
0.83
 
 
0.53
 
 
 
 
 HNSL 
 
 
hard palate
 
 
distance
 
 
ACE
 
 
2
 
 
II
 
 
0.31 (0.00, 0.78)
 
 
0.49 (0.02, 0.80)
 
 
0.0431 (1)
 
 
0.20 (0.14, 0.28)
 
 
0.77 (0.74, 0.80)
 
 
0.66
 
 
0.51
 
 
 
 
 L2CT 
 
 
larynx
 
 
distance
 
 
ACE
 
 
2
 
 
II
 
 
0.03 (0.00, 0.61)
 
 
0.60 (0.04, 0.72)
 
 
0.0377 (1)
 
 
0.37 (0.27, 0.48)
 
 
0.73 (0.69, 0.76)
 
 
0.55
 
 
0.42
 
 
 
 
 L3CT 
 
 
larynx
 
 
distance
 
 
ACE
 
 
2
 
 
II
 
 
0.00 (0.00, 0.52)
 
 
0.58 (0.06, 0.68)
 
 
0.0312 (1)
 
 
0.42 (0.32, 0.54)
 
 
0.69 (0.64, 0.73)
 
 
0.46
 
 
0.44
 
 
 
 
 LACT 
 
 
larynx
 
 
distance
 
 
ACE
 
 
3
 
 
IV
 
 
0.17 (0.00, 0.69)
 
 
0.47 (0.00, 0.71)
 
 
0.0778 (1)
 
 
0.36 (0.27, 0.46)
 
 
0.81 (0.78, 0.83)
 
 
0.64
 
 
0.37
 
 
 
 
 SVTv* 
 
 
general
 
 
distance
 
 
ACE
 
 
3
 
 
IV
 
 
0.24 (0.00, 0.74)
 
 
0.43 (0.00, 0.73)
 
 
0.118 (1)
 
 
0.32 (0.23, 0.43)
 
 
0.80 (0.77, 0.83)
 
 
0.68
 
 
0.32
 
 
 
 
 SHBN 
 
 
skull
 
 
distance
 
 
ACE
 
 
3
 
 
IV
 
 
0.56 (0.31, 0.91)
 
 
0.34 (0.00, 0.58)
 
 
0.0685 (1)
 
 
0.10 (0.08, 0.13)
 
 
0.96 (0.95, 0.96)
 
 
0.89
 
 
0.52
 
 
 
 
 SNNP 
 
 
skull
 
 
distance
 
 
ACE
 
 
3
 
 
IV
 
 
0.62 (0.28, 0.95)
 
 
0.29 (0.00, 0.63)
 
 
0.219 (1)
 
 
0.08 (0.04, 0.13)
 
 
0.82 (0.80, 0.85)
 
 
0.80
 
 
0.46
 
 
 
 
 SBAN 
 
 
skull
 
 
distance
 
 
ACE
 
 
3
 
 
IV
 
 
0.66 (0.36, 0.92)
 
 
0.22 (0.00, 0.52)
 
 
0.256 (1)
 
 
0.11 (0.08, 0.16)
 
 
0.89 (0.88, 0.91)
 
 
0.83
 
 
0.44
 
 
 
 
 HBPG 
 
 
hyoid
 
 
distance
 
 
ACE
 
 
3
 
 
IV
 
 
0.37 (0.00, 0.68)
 
 
0.22 (0.00, 0.62)
 
 
0.45 (1)
 
 
0.40 (0.31, 0.51)
 
 
0.81 (0.78, 0.83)
 
 
0.51
 
 
0.26
 
 
 
 
 DIMD 
 
 
dentition
 
 
distance
 
 
ACE
 
 
3
 
 
IV
 
 
0.29 (0.00, 0.75)
 
 
0.40 (0.00, 0.72)
 
 
0.133 (1)
 
 
0.32 (0.23, 0.42)
 
 
0.77 (0.73, 0.80)
 
 
0.58
 
 
0.41
 
 
 
 
 HBC2 
 
 
hyoid
 
 
distance
 
 
ACE
 
 
3
 
 
IV
 
 
0.35 (0.00, 0.73)
 
 
0.29 (0.00, 0.67)
 
 
0.325 (1)
 
 
0.37 (0.26, 0.49)
 
 
0.78 (0.74, 0.81)
 
 
0.64
 
 
0.28
 
 
 
 
 LAEA 
 
 
larynx
 
 
distance
 
 
ACE
 
 
3
 
 
IV
 
 
0.27 (0.00, 0.61)
 
 
0.23 (0.00, 0.58)
 
 
0.509 (1)
 
 
0.49 (0.38, 0.62)
 
 
0.77 (0.73, 0.80)
 
 
0.48
 
 
0.20
 
 
 
 
 HAML 
 
 
hard palate
 
 
distance
 
 
ACE
 
 
3
 
 
IV
 
 
0.04 (0.00, 0.75)
 
 
0.62 (0.00, 0.78)
 
 
0.0852 (1)
 
 
0.34 (0.20, 0.48)
 
 
0.56 (0.50, 0.61)
 
 
0.43
 
 
0.24
 
 
 
 
 CS3H 
 
 
cervical
 
 
distance
 
 
ACE
 
 
3
 
 
IV
 
 
0.20 (0.00, 0.89)
 
 
0.60 (0.00, 0.90)
 
 
0.117 (1)
 
 
0.20 (0.07, 0.34)
 
 
0.60 (0.54, 0.64)
 
 
0.52
 
 
0.32
 
 
 
 
 HCCC 
 
 
hard palate
 
 
curvature
 
 
ACE
 
 
3
 
 
IV
 
 
0.00 (0.00, 0.79)
 
 
0.57 (0.00, 0.85)
 
 
0.216 (1)
 
 
0.43 (0.15, 0.69)
 
 
0.31 (0.24, 0.38)
 
 
0.22
 
 
0.23
 
 
 
 
 SNNA 
 
 
skull
 
 
distance
 
 
ACE
 
 
3
 
 
IV
 
 
0.31 (0.00, 0.92)
 
 
0.56 (0.00, 0.91)
 
 
0.0904 (1)
 
 
0.13 (0.04, 0.24)
 
 
0.66 (0.61, 0.70)
 
 
0.63
 
 
0.26
 
 
 
 
 PWTP 
 
 
pharynx
 
 
Procrustes dist.
 
 
ACE
 
 
3
 
 
IV
 
 
0.00 (0.00, 0.70)
 
 
0.54 (0.00, 0.71)
 
 
0.238 (1)
 
 
0.46 (0.29, 0.64)
 
 
0.48 (0.42, 0.54)
 
 
0.31
 
 
0.23
 
 
 
 
 OCHL 
 
 
oral
 
 
ratio
 
 
ACE
 
 
3
 
 
IV
 
 
0.00 (0.00, 0.76)
 
 
0.54 (0.00, 0.77)
 
 
0.252 (1)
 
 
0.46 (0.23, 0.69)
 
 
0.35 (0.28, 0.42)
 
 
0.17
 
 
0.10
 
 
 
 
 OMLW 
 
 
oral
 
 
ratio
 
 
ACE
 
 
3
 
 
IV
 
 
0.09 (0.00, 0.68)
 
 
0.52 (0.00, 0.71)
 
 
0.0744 (1)
 
 
0.38 (0.27, 0.51)
 
 
0.70 (0.65, 0.73)
 
 
0.54
 
 
0.34
 
 
 
 
 HHPL 
 
 
hard palate
 
 
distance
 
 
ACE
 
 
3
 
 
IV
 
 
0.00 (0.00, 0.58)
 
 
0.51 (0.00, 0.66)
 
 
0.0698 (1)
 
 
0.49 (0.34, 0.65)
 
 
0.50 (0.44, 0.56)
 
 
0.30
 
 
0.31
 
 
 
 
 PPW5 
 
 
pharynx
 
 
curvature
 
 
ACE
 
 
3
 
 
IV
 
 
0.02 (0.00, 0.74)
 
 
0.51 (0.00, 0.73)
 
 
0.286 (1)
 
 
0.47 (0.24, 0.69)
 
 
0.41 (0.35, 0.48)
 
 
0.41
 
 
0.24
 
 
 
 
 LAEP 
 
 
larynx
 
 
distance
 
 
ACE
 
 
3
 
 
IV
 
 
0.07 (0.00, 0.65)
 
 
0.50 (0.00, 0.67)
 
 
0.106 (1)
 
 
0.43 (0.32, 0.55)
 
 
0.74 (0.70, 0.77)
 
 
0.55
 
 
0.33
 
 
 
 
 HNAP 
 
 
hard palate
 
 
distance
 
 
ACE
 
 
3
 
 
IV
 
 
0.17 (0.00, 0.39)
 
 
0.48 (0.00, 0.82)
 
 
0.363 (1)
 
 
0.36 (0.15, 0.57)
 
 
0.37 (0.30, 0.44)
 
 
0.28
 
 
0.11
 
 
 
 
 HIML* 
 
 
hard palate
 
 
distance
 
 
ACE
 
 
3
 
 
IV
 
 
0.24 (0.00, 0.78)
 
 
0.47 (0.00, 0.77)
 
 
0.125 (1)
 
 
0.29 (0.19, 0.41)
 
 
0.65 (0.60, 0.69)
 
 
0.51
 
 
0.30
 
 
 
 
 ALNS 
 
 
skull
 
 
angle
 
 
ACE
 
 
3
 
 
IV
 
 
0.26 (0.00, 0.80)
 
 
0.46 (0.00, 0.78)
 
 
0.221 (1)
 
 
0.29 (0.18, 0.41)
 
 
0.60 (0.55, 0.65)
 
 
0.46
 
 
0.15
 
 
 
 
 HICL* 
 
 
hard palate
 
 
distance
 
 
ACE
 
 
3
 
 
IV
 
 
0.14 (0.00, 0.80)
 
 
0.44 (0.00, 0.78)
 
 
0.443 (1)
 
 
0.42 (0.20, 0.64)
 
 
0.39 (0.32, 0.45)
 
 
0.29
 
 
0.10
 
 
 
 
 L5CT 
 
 
larynx
 
 
distance
 
 
ACE
 
 
3
 
 
IV
 
 
0.13 (0.00, 0.65)
 
 
0.44 (0.00, 0.65)
 
 
0.108 (1)
 
 
0.43 (0.32, 0.56)
 
 
0.71 (0.67, 0.75)
 
 
0.44
 
 
0.40
 
 
 
 
 DMAC 
 
 
dentition
 
 
curvature
 
 
ACE
 
 
3
 
 
IV
 
 
0.00 (0.00, 0.85)
 
 
0.43 (0.00, 0.82)
 
 
0.587 (1)
 
 
0.57 (0.17, 0.91)
 
 
0.21 (0.13, 0.28)
 
 
0.10
 
 
0.14
 
 
 
 
 PPW7 
 
 
pharynx
 
 
curvature
 
 
ACE
 
 
3
 
 
IV
 
 
0.30 (0.00, 0.80)
 
 
0.43 (0.00, 0.77)
 
 
0.17 (1)
 
 
0.28 (0.19, 0.39)
 
 
0.71 (0.67, 0.74)
 
 
0.59
 
 
0.25
 
 
 
 
 HIPL* 
 
 
hard palate
 
 
distance
 
 
ACE
 
 
3
 
 
IV
 
 
0.15 (0.00, 0.68)
 
 
0.41 (0.00, 0.67)
 
 
0.241 (1)
 
 
0.43 (0.31, 0.57)
 
 
0.63 (0.58, 0.67)
 
 
0.39
 
 
0.27
 
 
 
 
 CS7H 
 
 
cervical
 
 
distance
 
 
ACE
 
 
3
 
 
IV
 
 
0.25 (0.00, 0.74)
 
 
0.40 (0.00, 0.72)
 
 
0.229 (1)
 
 
0.35 (0.25, 0.47)
 
 
0.69 (0.64, 0.72)
 
 
0.52
 
 
0.33
 
 
 
 
 HACL 
 
 
hard palate
 
 
distance
 
 
ACE
 
 
3
 
 
IV
 
 
0.00 (0.00, 0.67)
 
 
0.35 (0.00, 0.55)
 
 
0.593 (1)
 
 
0.65 (0.36, 0.93)
 
 
0.26 (0.19, 0.33)
 
 
0.11
 
 
0.15
 
 
 
 
 DSIL 
 
 
dentition
 
 
distance
 
 
ACE
 
 
3
 
 
IV
 
 
0.30 (0.00, 0.82)
 
 
0.35 (0.00, 0.78)
 
 
0.419 (1)
 
 
0.35 (0.18, 0.53)
 
 
0.45 (0.39, 0.51)
 
 
0.35
 
 
0.19
 
 
 
 
 HCTH 
 
 
hard palate
 
 
distance
 
 
ACE
 
 
3
 
 
IV
 
 
0.19 (0.00, 0.73)
 
 
0.34 (0.00, 0.70)
 
 
0.507 (1)
 
 
0.47 (0.27, 0.67)
 
 
0.44 (0.37, 0.50)
 
 
0.31
 
 
0.18
 
 
 
 
 HAPL 
 
 
hard palate
 
 
distance
 
 
ACE
 
 
3
 
 
IV
 
 
0.12 (0.00, 0.61)
 
 
0.34 (0.00, 0.60)
 
 
0.442 (1)
 
 
0.54 (0.38, 0.70)
 
 
0.52 (0.46, 0.57)
 
 
0.27
 
 
0.23
 
 
 
 
 OCHW 
 
 
oral
 
 
ratio
 
 
ACE
 
 
3
 
 
IV
 
 
0.20 (0.00, 0.72)
 
 
0.33 (0.00, 0.68)
 
 
0.417 (1)
 
 
0.47 (0.27, 0.68)
 
 
0.41 (0.34, 0.47)
 
 
0.22
 
 
0.15
 
 
 
 
 L2EP 
 
 
larynx
 
 
distance
 
 
ACE
 
 
3
 
 
IV
 
 
0.28 (0.00, 0.71)
 
 
0.33 (0.00, 0.68)
 
 
0.326 (1)
 
 
0.39 (0.28, 0.52)
 
 
0.67 (0.62, 0.71)
 
 
0.47
 
 
0.29
 
 
 
 
 SNOL 
 
 
skull
 
 
distance
 
 
ACE
 
 
3
 
 
IV
 
 
0.69 (0.09, 1.00)
 
 
0.31 (0.00, 0.90)
 
 
0.43 (1)
 
 
0.00 (0.00, 0.11)
 
 
0.54 (0.48, 0.59)
 
 
0.60
 
 
0.29
 
 
 
 
 L3EP 
 
 
larynx
 
 
distance
 
 
ACE
 
 
3
 
 
IV
 
 
0.29 (0.00, 0.72)
 
 
0.31 (0.00, 0.68)
 
 
0.391 (1)
 
 
0.41 (0.28, 0.56)
 
 
0.57 (0.52, 0.62)
 
 
0.36
 
 
0.27
 
 
 
 
 HICH 
 
 
hard palate
 
 
distance
 
 
ACE
 
 
3
 
 
IV
 
 
0.17 (0.00, 0.68)
 
 
0.30 (0.00, 0.63)
 
 
0.479 (1)
 
 
0.53 (0.32, 0.75)
 
 
0.39 (0.32, 0.46)
 
 
0.17
 
 
0.12
 
 
 
 
 OPLW 
 
 
oral
 
 
ratio
 
 
ACE
 
 
3
 
 
IV
 
 
0.15 (0.00, 0.61)
 
 
0.29 (0.00, 0.58)
 
 
0.477 (1)
 
 
0.56 (0.39, 0.74)
 
 
0.50 (0.44, 0.56)
 
 
0.29
 
 
0.25
 
 
 
 
 HIPH 
 
 
hard palate
 
 
distance
 
 
ACE
 
 
3
 
 
IV
 
 
0.26 (0.00, 0.72)
 
 
0.27 (0.00, 0.68)
 
 
0.565 (1)
 
 
0.46 (0.28, 0.65)
 
 
0.43 (0.36, 0.49)
 
 
0.27
 
 
0.14
 
 
 
 
 HIML 
 
 
hard palate
 
 
distance
 
 
ACE
 
 
3
 
 
IV
 
 
0.44 (0.00, 0.81)
 
 
0.27 (0.00, 0.73)
 
 
0.413 (1)
 
 
0.29 (0.18, 0.43)
 
 
0.63 (0.58, 0.67)
 
 
0.51
 
 
0.24
 
 
 
 
 HACP 
 
 
hard palate
 
 
angle
 
 
ACE
 
 
3
 
 
IV
 
 
0.00 (0.00, 0.42)
 
 
0.25 (0.00, 0.43)
 
 
0.479 (1)
 
 
0.75 (0.57, 0.93)
 
 
0.47 (0.41, 0.53)
 
 
0.13
 
 
0.10
 
 
 
 
 OCMR 
 
 
oral
 
 
ratio
 
 
ACE
 
 
3
 
 
IV
 
 
0.29 (0.00, 0.70)
 
 
0.25 (0.00, 0.65)
 
 
0.55 (1)
 
 
0.47 (0.30, 0.66)
 
 
0.47 (0.40, 0.53)
 
 
0.26
 
 
0.10
 
 
 
 
 OPHL 
 
 
oral
 
 
ratio
 
 
ACE
 
 
3
 
 
IV
 
 
0.22 (0.00, 0.67)
 
 
0.25 (0.00, 0.64)
 
 
0.663 (1)
 
 
0.53 (0.33, 0.74)
 
 
0.38 (0.31, 0.44)
 
 
0.21
 
 
0.09
 
 
 
 
 SPUN 
 
 
soft palate
 
 
distance
 
 
ACE
 
 
3
 
 
IV
 
 
0.45 (0.00, 0.77)
 
 
0.21 (0.00, 0.69)
 
 
0.52 (1)
 
 
0.34 (0.23, 0.47)
 
 
0.63 (0.58, 0.67)
 
 
0.47
 
 
0.33
 
 
 
 
 
 
 Dominance,  d  2  
 Because there is no correlation between  d  2  and
the inter-rater agreement  ICC ( C ,1) (Pearson’s
 r =0.20,  p =0.174; Spearman’s  ρ =0.22,
 p =0.14), we will not consider agreement in our ranking of the
measures. 
 
 
  Table 31.    Rankings of measures ordered
decreassingly by the strength of evidence they provide for high
dominance effects  d  2 , with the number of measures in
each ranking. 
 
 
 
 
ranking
 
 
class
 
 
count
 
 
 d  2★c 
 
 
 d  2★ 
 
 
 d  2≫ 
 
 
 d  2> 
 
 
 
 
 
 
 1 
 
 
II
 
 
2
 
 
No
 
 
Yes
 
 
No
 
 
Yes
 
 
 
 
 2 
 
 
IV
 
 
2
 
 
No
 
 
No
 
 
Yes
 
 
Yes
 
 
 
 
 3 
 
 
IV
 
 
28
 
 
No
 
 
No
 
 
No
 
 
Yes
 
 
 
 
 4 
 
 
V
 
 
14
 
 
No
 
 
No
 
 
No
 
 
No
 
 
 
 
 There is no equivalent of  h  2 ’s ranking
 0 . 
 Ranking  1  gives evidence of a dimonance effect →
class  II . 
 Rankings  2  and  3  give marginal
evidence of a dominance effect → class  IV . 
 Ranking  4  basically gives no evidence of a dominance
effect → class  V . 
 
 
  Table 32.    The 32 measures with at least
some evidence of dominance effects (class < V) ordered by ranking and
 d  2 , also showing the nominal  p -value (and
the Holm-corrected  p -value). 
 
 
 
 
measure
 
 
domain
 
 
type
 
 
genetic model
 
 
ranking
 
 
class
 
 
 h  2 
 
 
 d  2 
 
 
 p 
 
 
 e  2 
 
 
ICC(C,1)
 
 
r MZ 
 
 
r DZ 
 
 
 
 
 
 
 MGMD 
 
 
mandible
 
 
distance
 
 
ADE
 
 
1
 
 
II
 
 
0.00 (0.00, 0.65)
 
 
0.85 (0.18, 0.90)
 
 
0.018 (1)
 
 
0.15 (0.10, 0.22)
 
 
0.80 (0.77, 0.83)
 
 
0.71
 
 
0.04
 
 
 
 
 DAII 
 
 
dentition
 
 
angle
 
 
ADE
 
 
1
 
 
II
 
 
0.00 (0.00, 0.37)
 
 
0.63 (0.19, 0.74)
 
 
0.0142 (1)
 
 
0.37 (0.26, 0.49)
 
 
0.66 (0.61, 0.70)
 
 
0.45
 
 
-0.12
 
 
 
 
 HPTH 
 
 
hard palate
 
 
distance
 
 
ADE
 
 
2
 
 
IV
 
 
0.00 (0.00, 0.85)
 
 
0.76 (0.62, 0.76)
 
 
0.271 (1)
 
 
0.24 (0.14, 0.38)
 
 
0.52 (0.46, 0.57)
 
 
0.44
 
 
0.05
 
 
 
 
 DAIM 
 
 
dentition
 
 
angle
 
 
ADE
 
 
2
 
 
IV
 
 
0.00 (0.00, 0.72)
 
 
0.70 (0.58, 0.81)
 
 
0.0604 (1)
 
 
0.30 (0.19, 0.42)
 
 
0.63 (0.58, 0.67)
 
 
0.50
 
 
-0.01
 
 
 
 
 MGPD 
 
 
mandible
 
 
distance
 
 
ADE
 
 
3
 
 
IV
 
 
0.05 (0.00, 0.87)
 
 
0.78 (0.00, 0.88)
 
 
0.141 (1)
 
 
0.17 (0.12, 0.23)
 
 
0.83 (0.80, 0.85)
 
 
0.74
 
 
0.12
 
 
 
 
 MCMD 
 
 
mandible
 
 
distance
 
 
ADE
 
 
3
 
 
IV
 
 
0.09 (0.00, 0.86)
 
 
0.75 (0.00, 0.88)
 
 
0.105 (1)
 
 
0.16 (0.12, 0.21)
 
 
0.92 (0.90, 0.93)
 
 
0.83
 
 
0.19
 
 
 
 
 MPGL 
 
 
mandible
 
 
distance
 
 
ADE
 
 
3
 
 
IV
 
 
0.15 (0.00, 0.83)
 
 
0.62 (0.00, 0.84)
 
 
0.261 (1)
 
 
0.22 (0.16, 0.30)
 
 
0.84 (0.82, 0.86)
 
 
0.74
 
 
0.15
 
 
 
 
 MPGR 
 
 
mandible
 
 
distance
 
 
ADE
 
 
3
 
 
IV
 
 
0.24 (0.00, 0.88)
 
 
0.60 (0.00, 0.89)
 
 
0.251 (1)
 
 
0.16 (0.11, 0.23)
 
 
0.84 (0.81, 0.86)
 
 
0.76
 
 
0.12
 
 
 
 
 MCPD 
 
 
mandible
 
 
distance
 
 
ADE
 
 
3
 
 
IV
 
 
0.26 (0.00, 0.86)
 
 
0.57 (0.00, 0.87)
 
 
0.219 (1)
 
 
0.18 (0.13, 0.23)
 
 
0.92 (0.90, 0.93)
 
 
0.82
 
 
0.19
 
 
 
 
 ACSN 
 
 
skull
 
 
angle
 
 
ADE
 
 
3
 
 
IV
 
 
0.30 (0.00, 0.84)
 
 
0.50 (0.00, 0.85)
 
 
0.31 (1)
 
 
0.20 (0.15, 0.26)
 
 
0.87 (0.85, 0.89)
 
 
0.71
 
 
0.25
 
 
 
 
 ABSN 
 
 
skull
 
 
angle
 
 
ADE
 
 
3
 
 
IV
 
 
0.36 (0.00, 0.84)
 
 
0.42 (0.00, 0.84)
 
 
0.386 (1)
 
 
0.21 (0.16, 0.29)
 
 
0.82 (0.79, 0.84)
 
 
0.66
 
 
0.26
 
 
 
 
 SSEN* 
 
 
skull
 
 
distance
 
 
ADE
 
 
3
 
 
IV
 
 
0.46 (0.00, 0.83)
 
 
0.32 (0.00, 0.83)
 
 
0.515 (1)
 
 
0.22 (0.16, 0.28)
 
 
0.90 (0.88, 0.91)
 
 
0.77
 
 
0.26
 
 
 
 
 DIPD 
 
 
dentition
 
 
distance
 
 
ADE
 
 
3
 
 
IV
 
 
0.33 (0.00, 0.72)
 
 
0.32 (0.00, 0.73)
 
 
0.56 (1)
 
 
0.35 (0.27, 0.46)
 
 
0.80 (0.77, 0.82)
 
 
0.55
 
 
0.23
 
 
 
 
 APNP 
 
 
skull
 
 
angle
 
 
ADE
 
 
3
 
 
IV
 
 
0.00 (0.00, 0.75)
 
 
0.71 (0.00, 0.79)
 
 
0.112 (1)
 
 
0.29 (0.21, 0.37)
 
 
0.76 (0.73, 0.79)
 
 
0.57
 
 
0.07
 
 
 
 
 L3EA 
 
 
larynx
 
 
distance
 
 
ADE
 
 
3
 
 
IV
 
 
0.02 (0.00, 0.63)
 
 
0.52 (0.00, 0.65)
 
 
0.406 (1)
 
 
0.45 (0.35, 0.58)
 
 
0.76 (0.72, 0.79)
 
 
0.44
 
 
0.11
 
 
 
 
 CS2H 
 
 
cervical
 
 
distance
 
 
ADE
 
 
3
 
 
IV
 
 
0.00 (0.00, 0.94)
 
 
0.88 (0.00, 0.94)
 
 
0.157 (1)
 
 
0.12 (0.05, 0.24)
 
 
0.59 (0.53, 0.64)
 
 
0.60
 
 
0.08
 
 
 
 
 MMPG 
 
 
mandible
 
 
distance
 
 
ADE
 
 
3
 
 
IV
 
 
0.00 (0.00, 1.00)
 
 
0.84 (0.00, 1.00)
 
 
0.226 (1)
 
 
0.16 (0.00, 0.40)
 
 
0.32 (0.24, 0.39)
 
 
0.29
 
 
0.02
 
 
 
 
 PNNP 
 
 
pharynx
 
 
distance
 
 
ADE
 
 
3
 
 
IV
 
 
0.02 (0.00, 0.85)
 
 
0.81 (0.00, 0.88)
 
 
0.11 (1)
 
 
0.18 (0.12, 0.25)
 
 
0.74 (0.70, 0.77)
 
 
0.64
 
 
0.20
 
 
 
 
 DAIP 
 
 
dentition
 
 
angle
 
 
ADE
 
 
3
 
 
IV
 
 
0.00 (0.00, 0.73)
 
 
0.70 (0.00, 0.80)
 
 
0.068 (1)
 
 
0.30 (0.20, 0.42)
 
 
0.62 (0.57, 0.67)
 
 
0.49
 
 
0.00
 
 
 
 
 HIPL 
 
 
hard palate
 
 
distance
 
 
ADE
 
 
3
 
 
IV
 
 
0.03 (0.00, 0.68)
 
 
0.53 (0.00, 0.69)
 
 
0.468 (1)
 
 
0.43 (0.31, 0.58)
 
 
0.60 (0.55, 0.65)
 
 
0.36
 
 
0.10
 
 
 
 
 HMTH 
 
 
hard palate
 
 
distance
 
 
ADE
 
 
3
 
 
IV
 
 
0.14 (0.00, 0.77)
 
 
0.49 (0.00, 0.79)
 
 
0.544 (1)
 
 
0.37 (0.21, 0.56)
 
 
0.46 (0.40, 0.52)
 
 
0.35
 
 
0.08
 
 
 
 
 AANP 
 
 
skull
 
 
angle
 
 
ADE
 
 
3
 
 
IV
 
 
0.00 (0.00, 0.63)
 
 
0.47 (0.00, 0.67)
 
 
0.349 (1)
 
 
0.53 (0.33, 0.75)
 
 
0.40 (0.33, 0.47)
 
 
0.23
 
 
0.06
 
 
 
 
 LEPL 
 
 
larynx
 
 
distance
 
 
ADE
 
 
3
 
 
IV
 
 
0.00 (0.00, 0.63)
 
 
0.46 (0.00, 0.68)
 
 
0.353 (1)
 
 
0.54 (0.32, 0.78)
 
 
0.40 (0.34, 0.47)
 
 
0.23
 
 
-0.10
 
 
 
 
 SIOD 
 
 
skull
 
 
distance
 
 
ADE
 
 
3
 
 
IV
 
 
0.58 (0.00, 1.00)
 
 
0.38 (0.00, 1.00)
 
 
0.502 (1)
 
 
0.04 (0.00, 0.11)
 
 
0.71 (0.67, 0.74)
 
 
0.74
 
 
0.30
 
 
 
 
 HMAC 
 
 
hard palate
 
 
curvature
 
 
ADE
 
 
3
 
 
IV
 
 
0.00 (0.00, 1.00)
 
 
0.37 (0.00, 1.00)
 
 
0.582 (1)
 
 
0.63 (0.00, 1.00)
 
 
0.08 (0.01, 0.16)
 
 
0.08
 
 
0.03
 
 
 
 
 L4CT 
 
 
larynx
 
 
distance
 
 
ADE
 
 
3
 
 
IV
 
 
0.00 (0.00, 0.49)
 
 
0.36 (0.00, 0.55)
 
 
0.29 (1)
 
 
0.64 (0.45, 0.86)
 
 
0.46 (0.39, 0.52)
 
 
0.17
 
 
-0.04
 
 
 
 
 HP2C 
 
 
hard palate
 
 
curvature
 
 
ADE
 
 
3
 
 
IV
 
 
0.00 (0.00, 0.62)
 
 
0.34 (0.00, 0.66)
 
 
0.542 (1)
 
 
0.66 (0.34, 0.97)
 
 
0.26 (0.19, 0.34)
 
 
0.13
 
 
0.06
 
 
 
 
 DICD 
 
 
dentition
 
 
distance
 
 
ADE
 
 
3
 
 
IV
 
 
0.31 (0.00, 0.74)
 
 
0.34 (0.00, 0.75)
 
 
0.612 (1)
 
 
0.35 (0.25, 0.47)
 
 
0.71 (0.67, 0.75)
 
 
0.50
 
 
0.23
 
 
 
 
 LHEP 
 
 
larynx
 
 
distance
 
 
ADE
 
 
3
 
 
IV
 
 
0.00 (0.00, 0.50)
 
 
0.32 (0.00, 0.51)
 
 
0.706 (1)
 
 
0.68 (0.49, 0.87)
 
 
0.47 (0.41, 0.53)
 
 
0.22
 
 
0.16
 
 
 
 
 APSN 
 
 
skull
 
 
angle
 
 
ADE
 
 
3
 
 
IV
 
 
0.54 (0.00, 0.91)
 
 
0.32 (0.00, 0.91)
 
 
0.566 (1)
 
 
0.14 (0.09, 0.21)
 
 
0.73 (0.69, 0.76)
 
 
0.67
 
 
0.29
 
 
 
 
 HM2C 
 
 
hard palate
 
 
curvature
 
 
ADE
 
 
3
 
 
IV
 
 
0.22 (0.00, 0.91)
 
 
0.30 (0.00, 0.88)
 
 
0.839 (1)
 
 
0.48 (0.07, 0.84)
 
 
0.23 (0.15, 0.30)
 
 
0.10
 
 
0.09
 
 
 
 
 LNEA 
 
 
larynx
 
 
distance
 
 
ADE
 
 
3
 
 
IV
 
 
0.25 (0.00, 0.63)
 
 
0.27 (0.00, 0.63)
 
 
0.668 (1)
 
 
0.48 (0.37, 0.62)
 
 
0.75 (0.71, 0.78)
 
 
0.46
 
 
0.08
 
 
 
 
 
 
 
 
 Appendices 
 
 Appendix I: Checking with  lavaan  
 As an extra check, we also implemented the genetic SEM model is
 lavaan . However, this re-implementation differs from the
main one in  OpenMX  due to some limitations of
 lavaan , as described below (our implementation is based on
the suggestions given in  Michel
G Nivard’s “Twin Strutural Equation Models in R & Lavaan”
(6/21/2021)  and  A.
Alexander Beaujean’s “Latent Variable Models in Education” slides (Fall
2012) ). 
 First, some notations: for a given PM, the data was organized as a
matrix with columns:  Zygosity  (“MZ” or “DZ”),  T1R1 ,
 T1R2 ,  T2R1 ,  T2R2  (the z-scored measure in
twin 1 by rater 1, in twin 1 by rater 2, etc.)and the covariates of
interest  “T1sex ,  T1age ,  T1age2 
(age 2 ),  T1icv ,  T2sex ,  T2age ,
 T2age2  and  T2icv  (all z-scored and arranged by
twin). 
 Second, the covariates could not be included directly in the model,
but were regressed out from the measure previous to fitting the SEM
model. More precisely, for each of  T1R1 ,  T1R2 ,
 T2R1 ,  T2R2 , we performed an independent multiple
regression  TiRj  ~  “Tisex  +  Tiage  +
 Tiage2  +  Tiicv  (where  i  ∈ {1,2} is the twin,
and  j  ∈ {1,2} is the rater), and we stored the residuals of
this regression in the variables  T1R1rez ,  T1R2rez ,
 T2R1rez ,  T2R2rez  – which we used in the SEM model. 
 Third, we implemented both the ACE and the ADE models, and we chose
between them using Akaike’s Information Criterion, AIC (i.e., we picked
the one with the smaller AIC). These models were implemented as
 grouped  models (the groups being the MZ and DZ twins) and we
tried to be as similar to the  OpenMX  implementation as
possible. The  lavaan  code is given below (please note that
we used the non-default optimizer  BFGS  because the default
one had convergence issues for a few PMs; also, we used a robust method
for estimating the standard errors – please see the  lavaan 
help for more info). 
   ACE:   
  ace.model  <- " # ACE model (using reziduals after controllng for covariates):
    
                # Raters:
                T1 =~ c(r1,r1)*T1R1rez + c(r2,r2)*T1R2rez # twin 1 is measured by both raters
                T2 =~ c(r1,r1)*T2R1rez + c(r2,r2)*T2R2rez # twin 2 is measured by both raters

                # Latents:
                A1 =~ NA*T1 + c(a,a)*T1 # A for twin 1
                A2 =~ NA*T2 + c(a,a)*T2 # A for twin 2 
                C1 =~ NA*T1 + c(c,c)*T1 # C for twin 1 
                C2 =~ NA*T2 + c(c,c)*T2 # C for twin 2 

                # Variances:
                A1 ~~ 1*A1 # A has variance 1
                A2 ~~ 1*A2 # A has variance 1
                C1 ~~ 1*C1 # C has variance 1
                C2 ~~ 1*C2 # C has variance 1
                    
                T1 ~~ c(e2,e2)*T1 # E is the residual variance of the phenotype
                T2 ~~ c(e2,e2)*T2 # E is the residual variance of the phenotype
                    
                # Covariances
                A1 ~~ c(1,.5)*A2 # for A, the correlation is 1 for MZ twins and 0.5 for DZ twins
                A1 ~~ 0*C1 + 0*C2 # A and C are uncorrelated
                A2 ~~ 0*C1 + 0*C2 # A and C are uncorrelated 
                C1 ~~ c(1,1)*C2 # C is correlated 1 regardless of twin status
                                # by lavaan default, E is uncorrelated with A and C, so no need to model it
              ";
ace.fit <- sem(ace.model, data=d, group="zyg", se="robust", optim.method="BFGS");  
   ADE:   
  ade.model  <- " # ADE model (using reziduals after controllng for covariates):
    
                # Raters:
                T1 =~ c(r1,r1)*T1R1rez + c(r2,r2)*T1R2rez # twin 1 is measured by both raters
                T2 =~ c(r1,r1)*T2R1rez + c(r2,r2)*T2R2rez # twin 2 is measured by both raters

                # Latents:
                A1 =~ NA*T1 + c(a,a)*T1 # A for twin 1
                A2 =~ NA*T2 + c(a,a)*T2 # A for twin 2 
                D1 =~ NA*T1 + c(d,d)*T1 # D for twin 1 
                D2 =~ NA*T2 + c(d,d)*T2 # D for twin 2 

                # Variances:
                A1 ~~ 1*A1 # A has variance 1
                A2 ~~ 1*A2 # A has variance 1
                D1 ~~ 1*D1 # D has variance 1
                D2 ~~ 1*D2 # D has variance 1
                    
                T1 ~~ c(e2,e2)*T1 # E is the residual variance of the phenotype
                T2 ~~ c(e2,e2)*T2 # E is the residual variance of the phenotype
                    
                # Covariances
                A1 ~~ c(1,.5)*A2 # for A, the correlation is 1 for MZ twins and 0.5 for DZ twins
                A1 ~~ 0*D1 + 0*D2 # A and D are uncorrelated
                A2 ~~ 0*D1 + 0*D2 # A and D are uncorrelated 
                D1 ~~ c(1,.25)*D2 # non-additive genetic effects D are correlated 1 for MZ twins and .25 for DZ
                                  # by lavaan default, E is uncorrelated with A and D, so no need to model it
              ";
ade.fit <- sem(ade.model, data=d, group="zyg", se="robust", optim.method="BFGS");  
  lavaan  returns the standardized estimates with standard
errors, 95%CIs and  p -values, but for the important components
( A ,  C  and  D ) we also performed model
comparison using AIC and the likelihood ratio test for nested models (as
implemented by  lavTestLRT() ) between the “full” model (ACE
or ADE, respectively) and the “reduced” model without the component of
interest (AE and CE, or AE and DE, respectively). With these, we
obtained a list of all PMs with the preferred model (ACE or ADE), their
standardized point estimates, standard errors, 95%CIs,  p -values
and (for  A ,  C  and  D ) the ΔAIC and
χ 2  LRT test, which we compared with their corresponding main
 OpenMX  estimates. 
 
 Genetic model 
 The choice between the ACE and DE genetic models is based on
different approaches: the “ r  MZ  vs
2 r  DZ ” heuristic for  OpenMX , and formal
ΔAIC model comparison for  lavaan , but it turns out that
they agree pretty well: 
 
 
 
 
 
 
 
 
   
 ACE 
 ADE 
 
 
 
 
  ACE  
 81 
 7 
 
 
  ADE  
 12 
 46 
 
 
 
 For 127 (87.0%) the chosen genetic model is the same, but for the
remaining 19 (13.0%) it isn’t: 
 
 
  Table 33.    The phenotypes where the genetic
model chosen differs between  OpenMX  and
 lavaan , also showing the difference between
 r  MZ  and 2 r ~DZ* (for  OpenMX )
and the ΔAIC between ACE and ADE (for  lavaan ). 
 
 
 
 
measure
 
 
genetic model ( OpenMX )
 
 
 r  MZ  - 2 r  DZ 
 
 
genetic model ( lavaan )
 
 
ΔAIC(ACE, ADE)
 
 
 
 
 
 
APNS
 
 
ACE
 
 
-0.02
 
 
ADE
 
 
0.15
 
 
 
 
ASNP
 
 
ACE
 
 
-0.12
 
 
ADE
 
 
0.00
 
 
 
 
CS2A
 
 
ACE
 
 
-0.10
 
 
ADE
 
 
0.10
 
 
 
 
CS5H
 
 
ACE
 
 
-0.06
 
 
ADE
 
 
0.01
 
 
 
 
HARF
 
 
ACE
 
 
-0.67
 
 
ADE
 
 
0.01
 
 
 
 
L4EA
 
 
ACE
 
 
-0.17
 
 
ADE
 
 
0.02
 
 
 
 
LHCT
 
 
ACE
 
 
-0.08
 
 
ADE
 
 
0.01
 
 
 
 
LHEA
 
 
ACE
 
 
-0.14
 
 
ADE
 
 
0.03
 
 
 
 
MICD
 
 
ACE
 
 
-0.01
 
 
ADE
 
 
0.08
 
 
 
 
OMHL
 
 
ACE
 
 
-0.11
 
 
ADE
 
 
0.20
 
 
 
 
OPHL
 
 
ACE
 
 
-0.25
 
 
ADE
 
 
0.00
 
 
 
 
PPW7
 
 
ACE
 
 
-0.43
 
 
ADE
 
 
0.17
 
 
 
 
CS6H
 
 
ADE
 
 
0.00
 
 
ACE
 
 
-0.04
 
 
 
 
DIIL
 
 
ADE
 
 
0.06
 
 
ACE
 
 
0.00
 
 
 
 
HIMH
 
 
ADE
 
 
0.01
 
 
ACE
 
 
-0.01
 
 
 
 
HMAC
 
 
ADE
 
 
1.89
 
 
ACE
 
 
-0.51
 
 
 
 
PPW2
 
 
ADE
 
 
0.72
 
 
ACE
 
 
-0.92
 
 
 
 
SBNP
 
 
ADE
 
 
0.08
 
 
ACE
 
 
0.00
 
 
 
 
SSEN*
 
 
ADE
 
 
0.16
 
 
ACE
 
 
-0.03
 
 
 
 
 It can be seen that, in all cases, the  lavaan  ACE and
ADE models are virtually indistinguishable (the ΔAIC(ACE, ADE) faisl to
reach 2 AIC points), and that in some of these, the  OpenMX 
latent  r  MZ  and 2 r  DZ  are also
very close, suggesting that these differences in the genetic model are
rather superficial. 
 
 
 Narrow-sense heritability estimates,  a  2  
 There is a pretty good correlation between the narrow-sense
heritability estimates from  OpenMX  ( h  2 )
and  lavaan  ( a  2 ), and the measures where
the two pick different genetic models (ACE vs ADE) do not seem to be
especially divergent (see also figure below): Pearson’s  r  =
0.82 ( p  = 2.08e-37) and Spearman’s  ρ  = 0.82
( p  = 2.39e-36). 
 
 
 
  Figure 42.    Relationship between naroow
sense heritability estimates using  OpenMX  and
 lavaan , also highlighting the measures where the two fitted
the same genetic model (or not). 
 
 
 we linearly regressed the one on the other, as follows: 
 
  lavaan  ~  OpenMX  
 This linear regressions has an adjusted  R  2  =
67.7%, intercept  α  = 0.070 (±0.036,  p  = 0.000156) and
slope  β  = 0.803 (±0.092,  p  = 2.08e-37). 
 The most important outliers and influential points are: 
 
 
 
  Figure 43.    Identifyng outliers and
influential points. 
 
 
 
 
  Table 34.    The outlier phenotypes ordered
by decreasing difference between  lavaan  and
 OpenMX  estimates. 
 
 
 
 
measure
 
 
 OpenMX 
 
 
 p 
 
 
 lavaan 
 
 
 p 
 
 
 
 
 
 
SSEN*
 
 
0.46 (0.00, 0.83)
 
 
0.393
 
 
0.72 (0.31, 1.30)
 
 
0.000
 
 
 
 
PWTP
 
 
0.00 (NA, 0.70)
 
 
1.000
 
 
0.25 (0.06, 1.54)
 
 
0.186
 
 
 
 
L3EP
 
 
0.29 (0.00, 0.72)
 
 
0.401
 
 
0.50 (0.08, 1.28)
 
 
0.001
 
 
 
 
CS2H
 
 
0.00 (NA, 0.94)
 
 
1.000
 
 
0.00 (0.00, 0.00)
 
 
NA
 
 
 
 
 
 
  OpenMX  ~  lavaan  
 This linear regressions has an adjusted  R  2  =
67.7%, intercept  α  = 0.034 (±0.038,  p  = 0.0757) and
slope  β  = 0.847 (±0.097,  p  = 2.08e-37). 
 The most important outliers and influential points are: 
 
 
 
  Figure 44.    Identifyng outliers and
influential points. 
 
 
 
 
  Table 35.    The outlier phenotypes ordered
by decreasing difference between  lavaan  and
 OpenMX  estimates. 
 
 
 
 
measure
 
 
 OpenMX 
 
 
 p 
 
 
 lavaan 
 
 
 p 
 
 
 
 
 
 
SSEN*
 
 
0.46 (0.00, 0.83)
 
 
0.393
 
 
0.72 (0.31, 1.30)
 
 
0.000
 
 
 
 
PWTP
 
 
0.00 (NA, 0.70)
 
 
1.000
 
 
0.25 (0.06, 1.54)
 
 
0.186
 
 
 
 
L2EP
 
 
0.28 (0.00, 0.71)
 
 
0.359
 
 
0.46 (0.06, 1.23)
 
 
0.002
 
 
 
 
CS4H
 
 
0.00 (NA, 0.32)
 
 
1.000
 
 
0.00 (0.00, 0.00)
 
 
NA
 
 
 
 
CS2A
 
 
0.88 (0.36, 1.00)
 
 
0.001
 
 
0.73 (0.02, 2.42)
 
 
0.017
 
 
 
 
SNOR
 
 
0.91 (0.00, 1.00)
 
 
0.290
 
 
0.32 (0.93, 4.40)
 
 
0.469
 
 
 
 
 
 
 
 Conslusions  OpenMX  vs  lavaan  
 Thus, it is comforting that two such different implementations
support each other. However, given the limitations of our
 lavaan  implementation, we will use the  OpenMX 
results throughout. 
 
 
 
 Appendix II: Session information 
  CPU:  Apple M1 (8 threads) 
  RAM (memory):  17.2 GB 
  OS:   macOS  
  R version 4.1.3 (2022-03-10)  
  Platform:  aarch64-apple-darwin20 (64-bit) 
  locale: 
en_US.UTF-8||en_US.UTF-8||en_US.UTF-8||C||en_US.UTF-8||en_US.UTF-8 
  attached base packages:   parallel ,
 grid ,  stats ,  graphics ,  grDevices ,
 utils ,  datasets ,  methods  and
 base  
  other attached packages: 
 lavaanPlot(v.0.6.2) ,  lavaan(v.0.6-11) ,
 OpenMx(v.2.20.6) ,  psych(v.2.2.5) ,
 doParallel(v.1.0.17) ,  iterators(v.1.0.14) ,
 foreach(v.1.5.2) ,  stringr(v.1.4.0) ,
 MASS(v.7.3-57) ,  mets(v.1.2.9) ,
 lava(v.1.6.10) ,  timereg(v.2.0.2) ,
 corrplot(v.0.92) ,  effsize(v.0.8.1) ,
 lsr(v.0.5.2) ,  benchmarkme(v.1.0.7) ,
 gtools(v.3.9.2.1) ,  BlandAltmanLeh(v.0.3.1) ,
 DiagrammeR(v.1.0.9) ,  Hmisc(v.4.7-0) ,
 Formula(v.1.2-4) ,  survival(v.3.3-1) ,
 lattice(v.0.20-45) ,  tidyr(v.1.2.0) ,
 moments(v.0.14.1) ,  pander(v.0.6.5) ,
 kableExtra(v.1.3.4) ,  knitr(v.1.39) ,
 irr(v.0.84.1) ,  lpSolve(v.5.6.15) ,
 reshape2(v.1.4.4) ,  gridExtra(v.2.3) ,
 ggplot2(v.3.3.6) ,  dplyr(v.1.0.9) ,
 car(v.3.0-13)  and  carData(v.3.0-5)  
  loaded via a namespace (and not attached): 
 colorspace(v.2.0-3) ,  ellipsis(v.0.3.2) ,
 htmlTable(v.2.4.0) ,  base64enc(v.0.1-3) ,
 rstudioapi(v.0.13) ,  listenv(v.0.8.0) ,
 farver(v.2.1.0) ,  fansi(v.1.0.3) ,
 mvtnorm(v.1.1-3) ,  xml2(v.1.3.3) ,
 codetools(v.0.2-18) ,  splines(v.4.1.3) ,
 mnormt(v.2.0.2) ,  jsonlite(v.1.8.0) ,
 cluster(v.2.1.3) ,  png(v.0.1-7) ,
 compiler(v.4.1.3) ,  httr(v.1.4.3) ,
 backports(v.1.4.1) ,  assertthat(v.0.2.1) ,
 Matrix(v.1.4-1) ,  fastmap(v.1.1.0) ,
 cli(v.3.3.0) ,  visNetwork(v.2.1.0) ,
 htmltools(v.0.5.2) ,  tools(v.4.1.3) ,
 gtable(v.0.3.0) ,  glue(v.1.6.2) ,
 Rcpp(v.1.0.8.3) ,  jquerylib(v.0.1.4) ,
 vctrs(v.0.4.1) ,  svglite(v.2.1.0) ,
 nlme(v.3.1-157) ,  xfun(v.0.31) ,
 globals(v.0.15.0) ,  rvest(v.1.0.2) ,
 lifecycle(v.1.0.1) ,  future(v.1.25.0) ,
 scales(v.1.2.0) ,  RColorBrewer(v.1.1-3) ,
 yaml(v.2.3.5) ,  sass(v.0.4.1) ,
 rpart(v.4.1.16) ,  latticeExtra(v.0.6-29) ,
 stringi(v.1.7.6) ,  highr(v.0.9) ,
 checkmate(v.2.1.0) ,  benchmarkmeData(v.1.0.4) ,
 rlang(v.1.0.2) ,  pkgconfig(v.2.0.3) ,
 systemfonts(v.1.0.4) ,  evaluate(v.0.15) ,
 purrr(v.0.3.4) ,  htmlwidgets(v.1.5.4) ,
 labeling(v.0.4.2) ,  tidyselect(v.1.1.2) ,
 parallelly(v.1.31.1) ,  plyr(v.1.8.7) ,
 magrittr(v.2.0.3) ,  R6(v.2.5.1) ,
 generics(v.0.1.2) ,  DBI(v.1.1.2) ,
 pillar(v.1.7.0) ,  foreign(v.0.8-82) ,
 withr(v.2.5.0) ,  mgcv(v.1.8-40) ,
 abind(v.1.4-5) ,  nnet(v.7.3-17) ,
 tibble(v.3.1.7) ,  future.apply(v.1.9.0) ,
 crayon(v.1.5.1) ,  utf8(v.1.2.2) ,
 tmvnsim(v.1.0-2) ,  rmarkdown(v.2.14) ,
 jpeg(v.0.1-9) ,  pbivnorm(v.0.6.0) ,
 data.table(v.1.14.2) ,  digest(v.0.6.29) ,
 webshot(v.0.5.3) ,  numDeriv(v.2016.8-1.1) ,
 stats4(v.4.1.3) ,  RcppParallel(v.5.1.5) ,
 munsell(v.0.5.0) ,  viridisLite(v.0.4.0)  and
 bslib(v.0.3.1)  
 
 


 
 

 

 

 

 

 

 

 
 

 
 
